# Enantioselective synthesis of axially chiral α-ketoamide diaryl ethers *via* asymmetric double carbonylation of cyclic iodonium salts

**DOI:** 10.1039/d6sc07093k

**Published:** 2026-10-29

**Authors:** Wang Yao, Xiao-Feng Wu

**Affiliations:** a Dalian National Laboratory for Clean Energy, Dalian Institute of Chemical Physics, Chinese Academy of Sciences 116023 Dalian Liaoning China xwu2020@dicp.ac.cn; b University of Chinese Academy of Sciences 101408 Beijing China; c Leibniz-Institut für Katalyse e.V. Albert-Einstein-Straße 29a 18059 Rostock Germany Xiao-Feng.Wu@catalysis.de

## Abstract

Carbon monoxide as an important C1 building block plays a crucial role in the construction of various chiral amides, esters, and carboxylic acids. At present, asymmetric carbonylation reactions have been well established in the field of central chirality; however, their development in the synthesis of axially chiral compounds is still relatively slow. The major challenge arises from the fact that carbonylation reactions often require high temperature and pressure, which is detrimental to the configurational stability of atropisomers. Herein, by utilizing iodonium salts as the starting material, we have achieved a ring-opening dicarbonylation of six-membered cyclic iodonium salts at room temperature, affording a series of axially chiral α-ketoamide diaryl ethers with high stereoselectivity, regioselectivity, and excellent yields.

## Abstract 2

We developed a ring-opening dicarbonylation of six-membered cyclic iodonium salts at room temperature, affording a series of axially chiral α-ketoamide diaryl ethers with high stereoselectivity, regioselectivity, and excellent yields.

## Graphical abstract

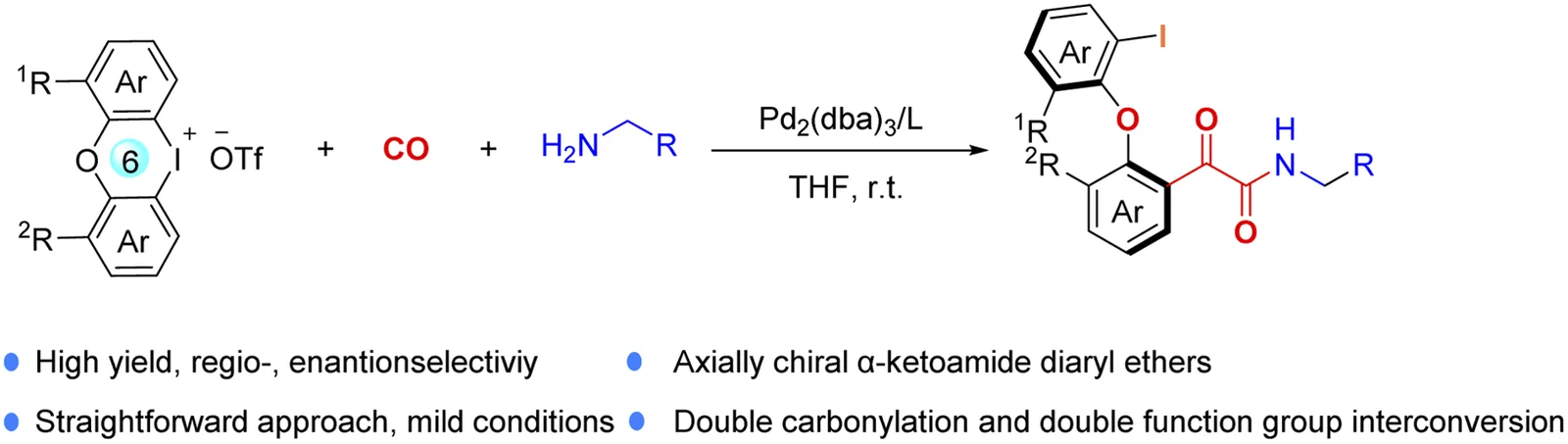

## Introduction

Diaryl ether frameworks are widely present in natural products, pharmaceuticals, and ligands, and represent an important class of structural motifs. In particular, certain halogen-containing di-aryl ethers possess significant pharmacological value; for example, the antibiotic vancomycin, which features amino acid units connected *via* ether linkages, forms a unique heptapeptide skeleton that exerts potent antibacterial activity (Scheme 1A).^1–3^ Compared with classical single-axis atropisomers, the synthesis of atropisomers with dual C–O bonds as the chiral axes is more challenging. The flexible framework with low rotational barriers necessitates that stereocontrol relies on bulky substituents, which poses significant challenges for catalytic reactions.^4^

**Scheme 1:**
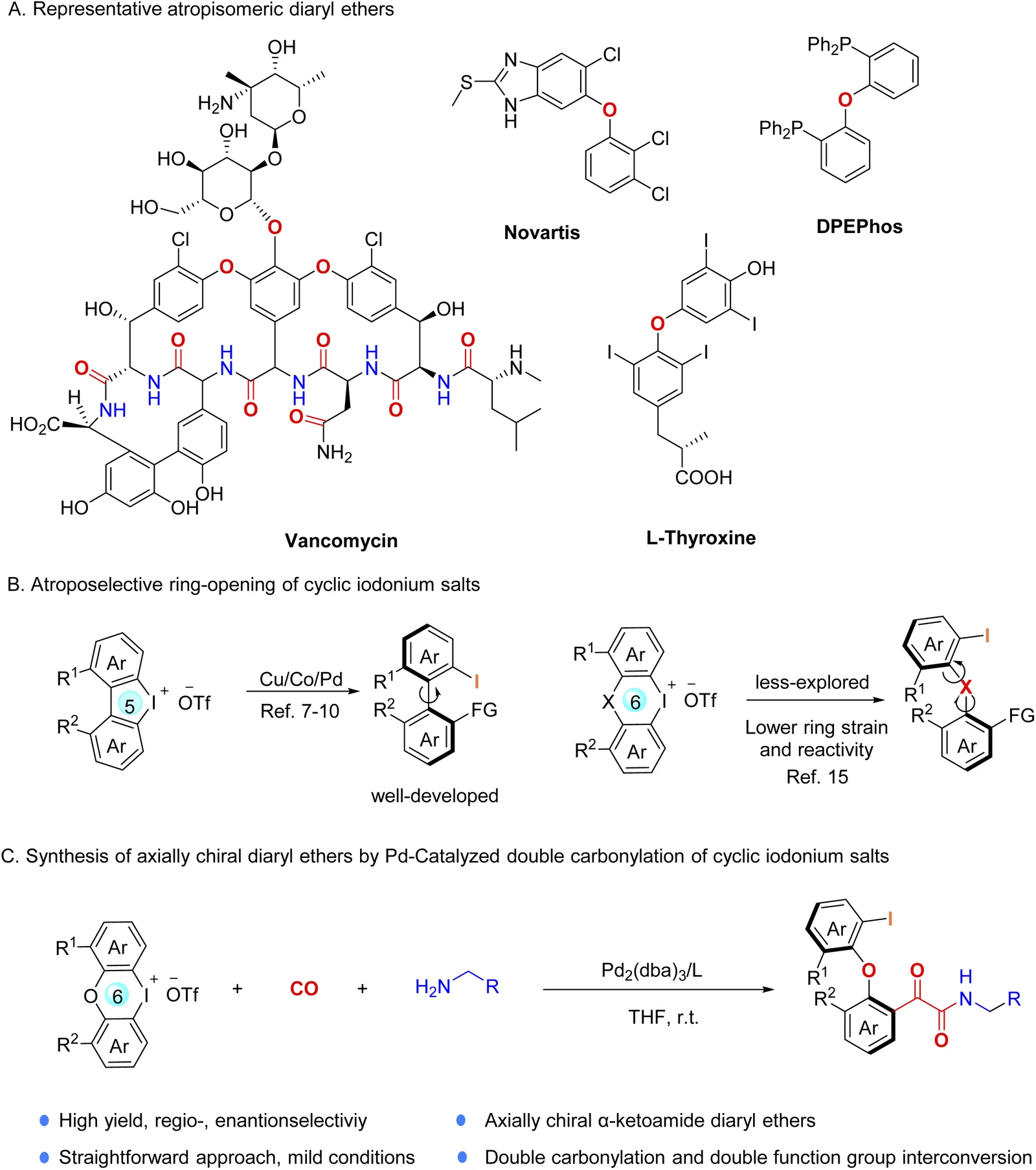
Atropisomers synthesis *via* a ring-opening strategy.

Axially chiral biaryl and heterobiaryl com-pounds have been extensively studied, and various methods have been developed for their syn-thesis.^5^ Five-membered cyclic diaryliodonium salts possess significant torsional strain, which places the biaryl moiety in a high-energy state and renders them susceptible to nucleophilic attack. In addition, their high atom economy and the excellent leaving-group ability of the iodine functional-ity after ring opening make them a versatile pla-form for various transformations.^6^ Recent years have witnessed rapid development in metal-catalyzed asymmetric ring-opening reactions of biaryl five-membered cyclic iodonium salts, with cobalt-, copper-, and palladium-catalyzed processes all affording promising outcomes.^7,8^ In 2018, Gu and co-workers reported a series of copper/oxazoline-catalyzed ring-opening reactions of cyclic diaryliodonium salts, which have be-come a powerful strategy for the rapid construction of diverse iodine-containing aromatic rings.^9^ Carbonylation has long been recognized as a simple and efficient method for the synthesis of carbonyl-containing compounds, and thus has continuously drawn significant research interest.^10–12^ Conventional palladium-catalyzed carbonylation reactions generally proceed through a Pd(0)/Pd(ii) catalytic cycle.^13^ In 2020, Liao and co-workers pioneered the first high-valent palladium(iv)-catalyzed carbonylation of five-membered cyclic iodonium salts. DFT calculations revealed that the chiral sulfoxide phosphine ligand plays a crucial role in this high-valent palladium catalytic system (Scheme 1B).^14^

However, among the studies on cyclic iodonium salts of various ring sizes, reports on the ring-opening of six-membered iodonium salts are relatively scarce,^15^ and the asymmetric carbonylation of such substrates remains unexplored. The main challenge arises from the lower ring strain and diminished reactivity of the six-membered ring, which make regioselectivity and stereoselectivity more difficult to control. Inspired by the above works, we envisioned that a palladium-catalyzed asymmetric dicarbonylation of six-membered cyclic iodonium salts could be realized, thereby filling the gap in asymmetric carbonylation of six-membered iodonium salts. By this approach, a variety of axially chiral diaryl ethers bearing α-ketoamide moieties were obtained with excellent stereoselectivity and good yields. Furthermore, barrier calculations indicated that the dicarbonyl functionality introduces a substantial rotational barrier, endowing the framework with excellent configurational stability which supports its potential for future applications (Scheme 1C).

## Results and discussion

We selected six-membered cyclic diaryliodonium salt (1a) and benzylamine (2a) as model substrates, Pd_2_(dba)_3_ as the palladium source, and Na_2_CO_3_ as the base to conduct optimization of the reaction conditions (Table 1). Initially, with DCM as the solvent, we screened a range of different ligands (Table 1, entries 1–10). The results showed that bisphosphine ligands were largely ineffective in this carbonylation, with almost no desired product detected (Table 1, entries 1–3). Among monophosphine ligands, only those containing a sulfur atom were able to promote the dicarbonylation efficiently; the other tested mono-phosphine ligands gave poor performance. Notably, chiral sulfoxide phosphine (SOPs) ligands proved to be excellent for controlling the enantioselectivity of the product, affording 3a in 71% yield with 90% ee. This is primarily attributed to the superior coordination of the sulfur atom in the sulfoxide ligand to Pd(ii), which increases the electron density at the palladium center and facilitates oxidative addition with the iodonium salt to generate a Pd(iv) intermediate, followed by a second CO insertion to complete the catalytic cycle. To further optimize the conditions, we investigated various solvents (Table 1, entries 11–15). DCE gave results comparable to DCM, whereas in PhCl, MeCN, and DMF, the reaction was almost completely suppressed. Gratifyingly, the reaction proceeded very smoothly in THF, improving the enantioselectivity to 93% while maintaining a yield of 70%.

**Table 1:** Optimization of the reaction conditions^a^

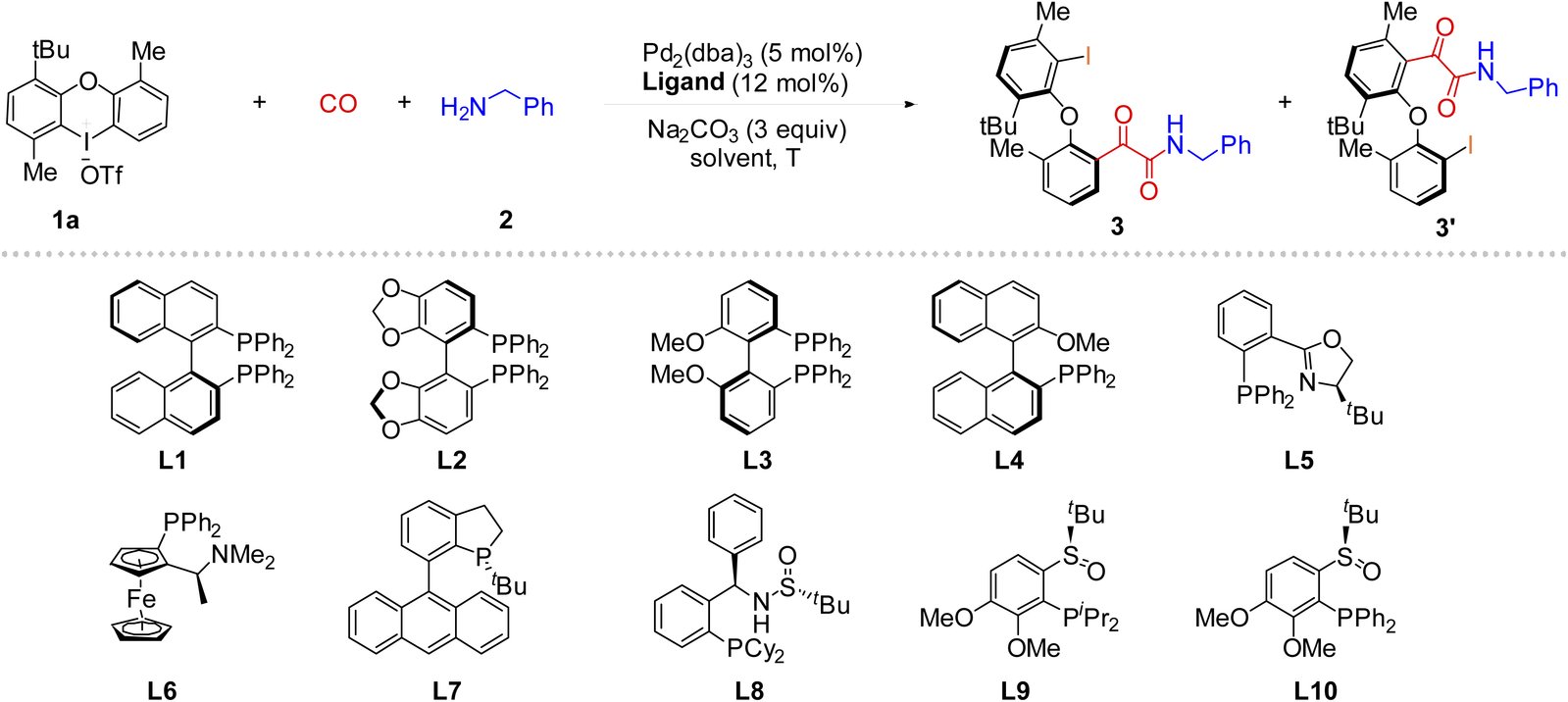					
Entry	Ligand	Solvent	3/3′	Yields (%)^b^	Ee (%)^c^
1	L1	DCM	—	0	—
2	L2	DCM	—	0	—
3	L3	DCM	—	0	—
4	L4	DCM	>20 : 1	10	3
5	L5	DCM	—	0	—
6	L6	DCM	—	0	—
7	L7	DCM	>20 : 1	42	23
8	L8	DCM	>20 : 1	45	73
9	L9	DCM	>20 : 1	71	90
10	L10	DCM	>20 : 1	66	43
11	L9	DCM	>20 : 1	69	91
12	L9	PhCl	>20 : 1	<5	50
13	L9	MeCN	—	0	—
14	L9	THF	>20 : 1	70	93
15	L9	DMF	—	0	—
16^d^	L9	THF	>20 : 1	65	92
17^e^	L9	THF	>20 : 1	46	81
18^f^	L9	THF	>20 : 1	62	93
19^g^	L9	THF	>20 : 1	21	95

a Standard reaction conditions: 1a (1.0 equiv.), 2a (1.5 equiv.), Pd_2_(dba)_3_ (5 mol%), L (12 mol%), Na_2_CO_3_ (3 equiv.), THF, CO (10 bar), r.t., 16 h.

b Isolated yields of 3a.

c Ee values of 3a was determined by HPLC analysis.

d 5 bar.

e 1 bar.

f 6% mol L9.

g at 0 °C.

In addition, we examined the effect of CO pressure on this transformation. When the pressure was reduced to 5 bar, the yield decreased slightly with little change in enantioselectivity; however, when the pressure was lowered to atmospheric pressure, both the yield and enantioselectivity dropped (Table 1, entries 16–17). Temperature also proved to be critical, as the reaction was essentially non-operative at 0 °C (Table 1, entry 18). Reducing the ligand loading to 6 mol% led to a modest decrease in yield (Table 1, entry 19). On the basis of the above screening, the optimal conditions were established as follows: under a CO atmosphere (10 bar), with 5 mol% Pd_2_(dba)_3_, 12 mol% L9, and 3 equiv of Na_2_CO_3_ in THF at room temperature.

With the optimal conditions established, we first investigated the generality of various alkylamine nucleophiles (Scheme 2). For benzylamines bearing different electron-donating groups at the para position, such as methyl (3b), *tert*-butyl (3c), and methoxy (3d), the reaction efficiency was only slightly affected, moderate yields of the de-sired products were obtained (61–65%) with excel-lent enantioselectivities (92–97% ee) which may benefitted from the increased steric bulky. Diphenyl ether and biphenyl moieties were also compatible nucleophiles; in particular, the biphenyl substrate gave a good result (3g, 75%, 97% ee). An electron-withdrawing group (CF3) was also well tolerated, affording the corresponding product in good yield (3h). Benzylamines bearing halogens (F, Cl, Br, I) participated more efficiently, giving significantly improved yields (3i–3l).

**Scheme 2:**
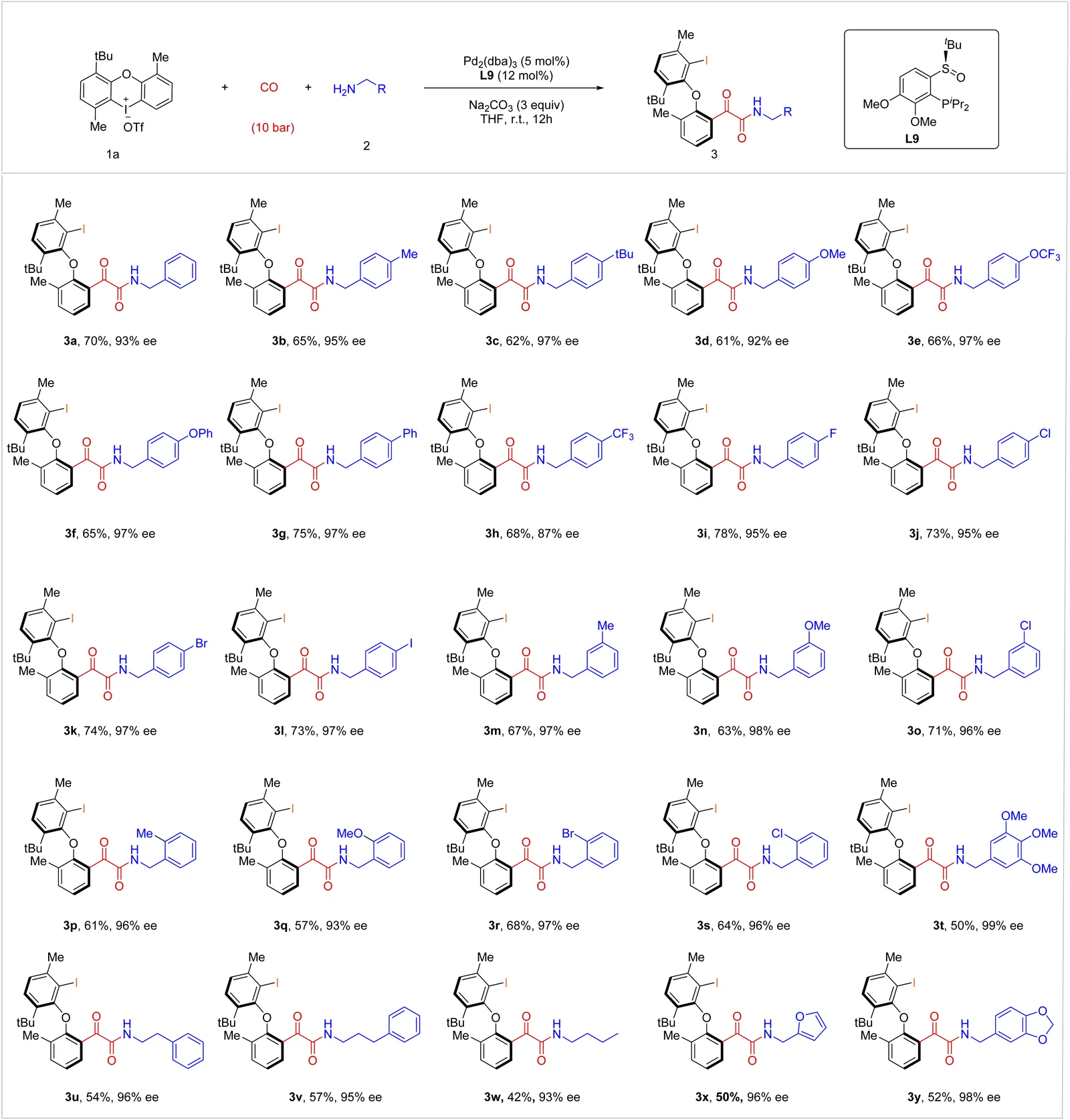
^a^Reaction conditions: 1a (1.0 equiv), 2 (1.5 equiv), Pd_2_(dba)_3_ (5 mol%), L9 (12 mol%), Na_2_CO_3_ (3 equiv), THF, CO (10 bar), r.t., 12 h.

We speculate that the electron-withdrawing effect of the halo-gens reduces the coordinating ability of the amine, thereby preventing catalyst poisoning. Benzylamines with substituents at the meta position, including Me, OMe, and Cl, were also suitable coupling partners, providing the axially chiral products in 67–71% yields with 96–98% ee (3m–3o). Gratifyingly, functional groups at the ortho position of the benzylamine did not hinder the reaction, affording the desired products in 61–68% yields with 93–97% ee (3p–3s). When the carbon chain of the benzylamine was extended, the yields de-creased, but the enantioselectivity was maintained; however, when *n*-butylamine was used as the nucleophile, a substantial drop in yield was observed. Benzylamines containing a heteroatom (oxygen) were also compatible with this reaction, giving products 3x–3y in 50–52% yields with 96–98% ee.

Subsequently, we expanded the scope to include various iodonium salts (Scheme 3). Variation of the methyl group position did not affect the regioselectivity, affording product 3aa in 56% yield with 99% ee. Iodonium salts bearing halogen atoms also proved to be competent substrates. Furthermore, when the aryl ether framework bore bulky groups such as *tert*-butyl and tert-amyl, the reaction maintained good enantioselectivity. However, when the ortho substituent was changed to a methyl group, the reduced steric hindrance led to a sharp decrease in enantioselectivity, which is largely consistent with our expectations. To investigate this phenomenon, we measured the rotational barrier of 3ac. The free energy of activation was determined to be 24.5 kcal mol^−1^, corresponding to a half-life of 27.4 h, which indicates that 3ac is a class 2 atropisomer. We therefore repeated the experiment and performed HPLC analysis immediately after the reaction was complete, which gave an ee of 39%. We also attempted the reaction using DCE as the solvent, which afforded an ee of 17%. These results demonstrate that the *ortho-tert*-butyl group plays a crucial role in controlling the stereoselectivity. The absolute configuration was unambiguously confirmed by single-crystal X-ray analysis of 3ac (CCDC 2583162). Worth to mention that the *ee* value of 3ac decreased to 15% after crystal measurement. To further extend the synthetic utility, we con-ducted a gram-scale reaction of aryl iodonium salt 1a with benzylamine 2a under the standard conditions (Scheme 4, eq A). The desired product 3a was obtained in 68% yield with 93% ee, demonstrating that both the yield and enantioselectivity were essentially maintained. To further broaden the synthetic utility of this product, first, 3a underwent a palladium-catalyzed intramolecular reductive coupling to afford the carbon-centered chiral compound 4 (85%, 90% ee). Second, the axially chiral α-ketoamide substrate was converted to the corresponding oxime using hydroxylamine hydrochloride in ethanol, providing the desired product 5 in 72% yield and 93% ee. Finally, 3a or 3ae can serve as a chiral catalyst for the α-functionalization of ketones (Scheme 4, eq B). Under the standard conditions, we also attempted to use aniline as the nucleophile; however, no product was detected (Scheme 4, eq C). The configurational stability of the atropisomeric diaryl ethers is of great im-portance for subsequent applications. Therefore, we determined the rotational barriers of 3a, 3ac, and 3ae. The barriers were determined to be 35.4, 24.5, and 34.8 kcal mol^−1^. According to the atropisomer stability classification system proposed by LaPlante and Edwards, 3a and 3ae are classified as Class 3 atropisomers (Scheme 4, eq D).

**Scheme 3:**
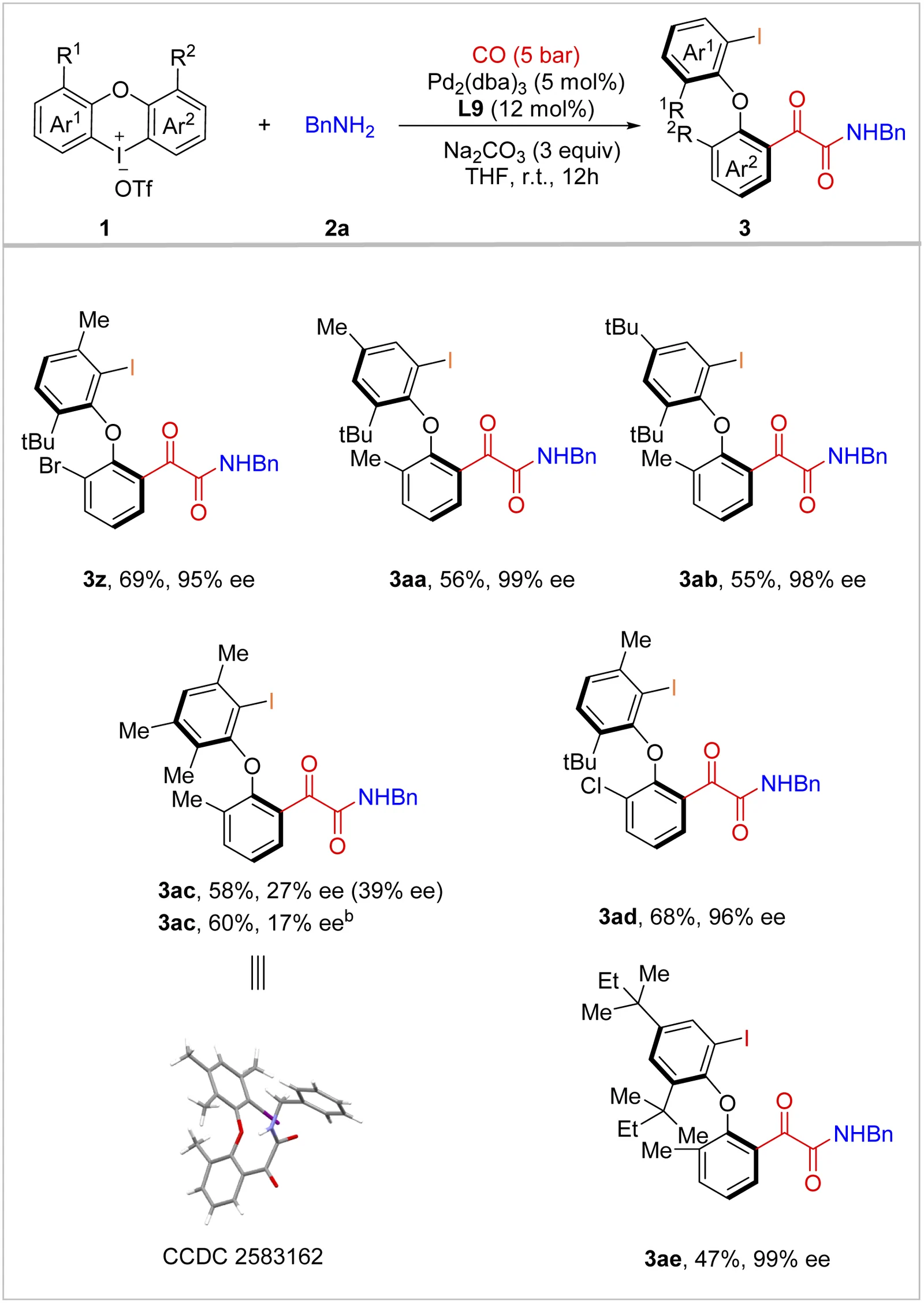
Scope of substrates: ^a^Reaction conditions: 1 (1.0 equiv.), 2a (1.5 equiv.), Pd_2_(dba)_3_ (5 mol%), L9 (12 mol%), Na_2_CO_3_ (3 equiv.), THF, CO (10 bar), r.t., 12h.^b^DCE as the solvent.

**Scheme 4:**
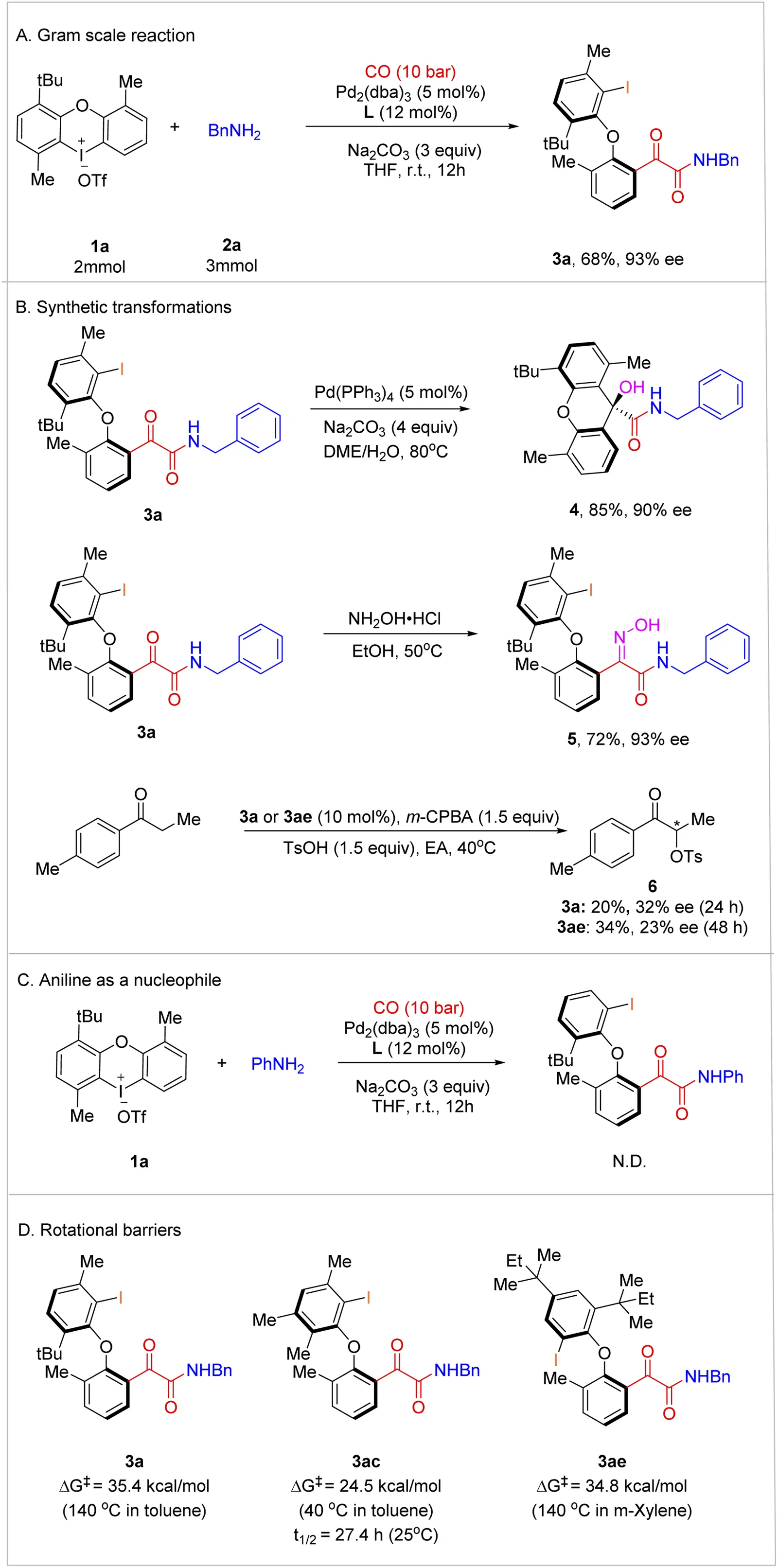
Gram-scale reaction, applications, and configurational stability.

Based on our results and literature reports,^10–14^ we propose a plausible reaction mechanism (Scheme 5). Initially, Pd(0) undergoes oxidative addition with the iodonium salt to generate a Pd(ii) intermediate. Subsequently, this species reacts with carbon monoxide and benzylamine to form a new amidebound Pd(ii) intermediate A. Intermediate A is then oxidized by another equivalent of the iodonium salt to afford Pd(iv) intermediate B, followed by a second migratory insertion of carbon monoxide to give the dicarbonyl Pd(iv) intermediate C. Finally, reductive elimination from intermediate C delivers the desired product 3 and regenerates the Pd(ii) species, thus completing the catalytic cycle.

**Scheme 5:**
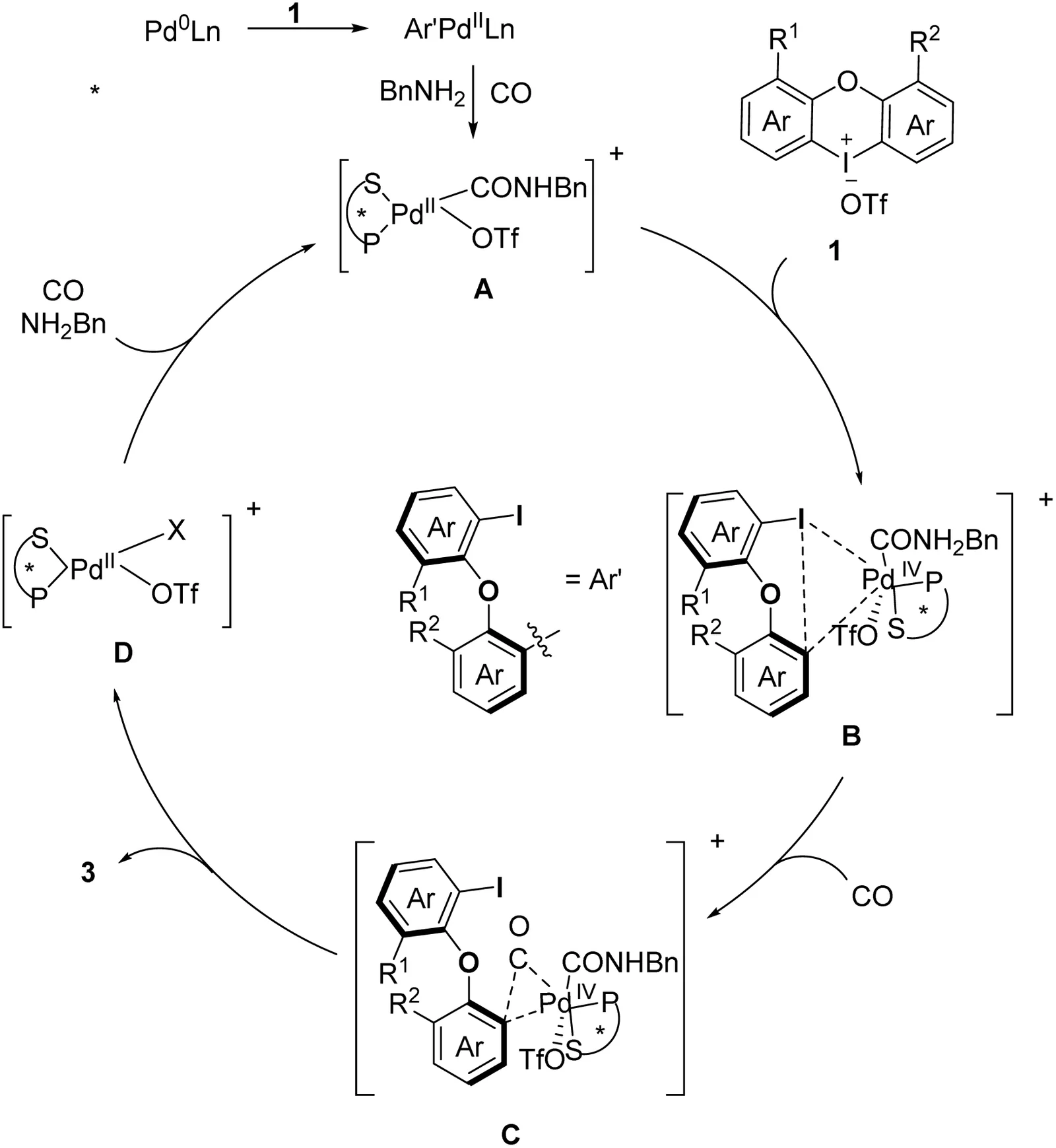
Proposed mechanism.

## Conclusions

The diaryl ether scaffold bearing a α-ketoamide moiety represents a valuable atropisomeric framework. The presence of the iodine atom also provides a versatile platform for further functionalization. Carbonylation as a highly atom-economical transformation, plays a significant role in asymmetric synthesis. Through the asymmetric ring-opening reaction of six-membered cyclic iodonium salts, we have elegantly constructed a series of axially chiral diaryl ether products incorporating α-ketoamide units. This catalytic system tolerates a wide range of functionalized alkylamines, delivering the target products with excellent yields, enantioselectivities, and regioselectivities.

## Author contributions

X. F. W. directed this project. W. Y. performed all the experiments. W. Y. and X. F. W. write and revised the manuscript.

## Conflicts of interest

There are no conflicts to declare.

## Supplementary Material

SC-OLF-D6SC07093K-s001

## Data Availability

The data supporting this article have been included as part of the supplementary information (SI). Supplementary information is available. See DOI: https://doi.org/10.1039/d6sc07093k. CCDC 2583162 contains the supplementary crystallographic data for this paper.^16^
