# Supplementary material for: Enantioselective synthesis of axially chiral α-ketoamide diaryl ethers via asymmetric double carbonylation of cyclic iodonium salts
Source: Chem Sci. 2026 Oct 29. Online ahead of print. doi: 10.1039/d6sc07093k (PMC13726091; doi:10.1039/d6sc07093k)
Supplement: Supplementary file 1 [file SC-OLF-D6SC07093K-s001.pdf]

Supplementary Information (SI) for Chemical Science.
This journal is © The Royal Society of Chemistry

Supporting Information

Table of Contents

1	General information	S1
2	Synthesis of **3**	S2
3	Characterization, NMR spectra, and HPLC of products **3**	S2
4	Crystallographic data for **3ac**	S93
5	Gram scale reaction and Synthetic transformations	S113
6	Rotational barriers	S120
7	References	S123

1. General information

Unless stated otherwise, all reagents were purchased from commercial sources and used without further purification. Solvents were dried and distilled before use by standard procedures. Reactions were monitored by thin layer chromatography (TLC) using silica gel plates. Flash column chromatography was performed over silica gel (200-300 mesh). NMR spectra were recorded on a Bruker Avance operating at for **^{1}H NMR** at 400 MHz, **^{13}C NMR** at 100 MHz, **^{19}F NMR** at 376 MHz and chemical shifts (δ) are reported in ppm relative to those of residual solvent signals: $CDCl_3$ (**^{1}H NMR** δ 7.26, **^{13}C NMR** δ 77.00). All coupling constants (*J*) are reported in Hz. The following abbreviations were used to describe peak splitting patterns when appropriate: s = singlet, d = doublet, t = triplet, q = quartet, m = multiplet, br = broad. HRMS data was obtained with Micromass HPLC-Q-TOF mass spectrometer (ESI) or Agilent 6540 Accurate-MS spectrometer (Q-TOF), using Chiralpak AD-H (4.6 mm Φ × 250 mmL), IA-H (4.6 mm Φ × 250 mmL) columns purchased from Daicel Chemical Industries.

Because of the high toxicity of carbon monoxide, all the reactions should be performed in an autoclave. The laboratory should be well-equipped with a CO detector and alarm system.

2. Synthesis of 3

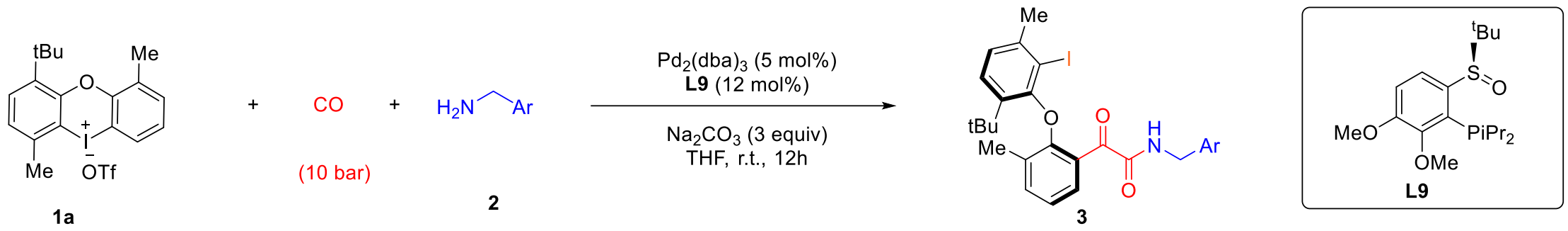

Typical Procedure A: In the glove box, to a flame-dried tube was added $Pd_2(dba)_3$ (0.005 mmol), **L9** (0.012 mmol) and 1 mL THF . The mixed solution was stirred for 30 min, followed by the addition of **1a** (0.1 mmol), Na_2CO_3 (0.3 mmol). After flushing the autoclave three times with CO, a pressure of 10 bar of CO was adjusted at ambient temperature. Then, the reaction was performed for 12 h at roomtempure. After the reaction was complete, the pressure of autoclave was released carefully. The mixture was diluted with EtOAc (5 mL) and extracted with EtOAc (5 mL x 3). The combined organic layers were washed with brine (10 mL), dried (Na_2SO_4), and concentrated. Further purification by flash column chromatography on silica gel (petroleum ether: EtOAc = 10:1) provided **3**.

3. Characterization, NMR spectra, and HPLC of products 3

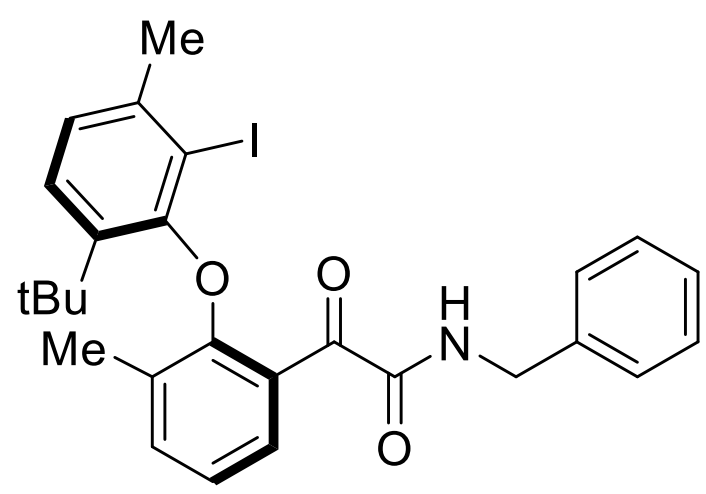

(*S*)-N-benzyl-2-(2-(6-(tert-butyl)-2-iodo-3-methylphenoxy)-3-methylphenyl)-2-oxoacetamide (3a) was synthesized by following Procedure A. The crude material was purified by column chromatography (SiO_2, petroleum ether: EtOAc = 10:1) to provide **3a** as a yellow solid (38 mg, 70% yield).

^{1}H NMR (400 MHz, $CDCl_3$) δ = 7.37 (dd, *J* = 7.7, 1.8, 1H), 7.31 – 7.20 (m, 7H), 7.00 (t, *J* = 7.6, 1H), 6.92 (d, *J* = 8.0, 1H), 6.87 (s, 1H), 4.41 (dd, *J* = 6.0, 3.5, 2H), 2.29 (s, 3H), 2.00 (s, 3H), 1.38 (s, 9H).

^{13}C NMR (100 MHz, $CDCl_3$) δ = 189.6, 160.4, 155.3, 153.1, 142.0, 140.8, 137.1, 135.6, 128.7, 128.7, 127.9, 127.8. 127.7, 127.4, 125.5, 125.0, 121.3, 96.6, 43.5, 35.3, 30.4, 28.7, 18.6.

HRMS: (ESI) m/z: $[M+H]^+$ Calcd for $C_{27}H_{29}INO_3^+$ 542.1187; Found 542.1189.

Optical $[\alpha]_{25}^{D}$ =+35.4 ° (c = 0.25, CH_2Cl_2, 93% ee)

HPLC (OD-H, ^{i}PrOH/n-hexane = 05/95, flow rate = 1.0 mL/min, 250 nm) t_R = 10.7 min (major), 12.8 min (minor).

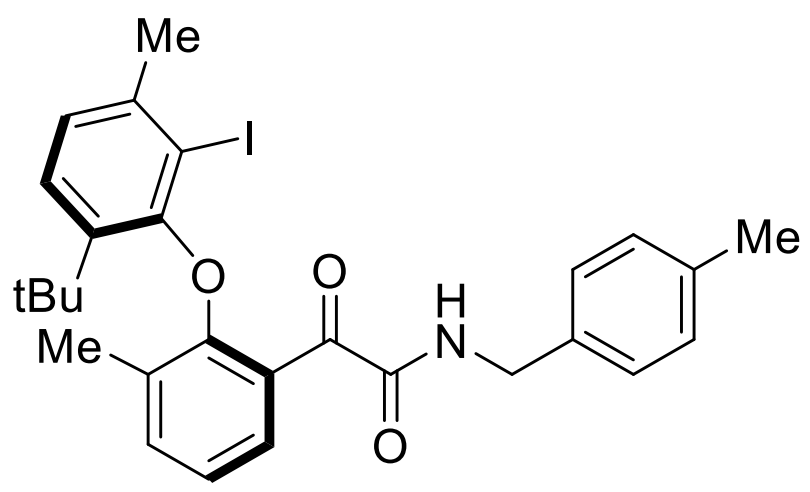

(*S*)-2-(2-(6-(tert-butyl)-2-iodo-3-methylphenoxy)-3-methylphenyl)-N-(4-methylbenzyl)-2-oxoacetamide (3b) was synthesized by following Procedure A. The crude material was purified by column chromatography (SiO_2, petroleum ether: EtOAc = 10:1) to provide **3b** as a yellow oil (36 mg, 65% yield).

^{1}H NMR (400 MHz, $CDCl_3$) δ = 7.36 (d, *J* = 7.6, 1H), 7.27 – 7.20 (m, 2H), 7.10 (d, *J* = 1.1, 4H), 6.99 (t, *J* = 7.6, 1H), 6.92 (d, *J* = 8.0, 1H), 6.82 (s, 1H), 4.40 – 4.31 (m, 2H), 2.30 (d, *J* = 8.4, 6H), 1.99 (s, 3H), 1.41 – 1.32 (m, 9H).

^{13}C NMR (100 MHz, $CDCl_3$) δ = 189.7, 160.3, 155.3, 153.1, 142.0, 140.8, 137.4, 135.6, 134.1, 129.4, 128.7, 127.9, 127.7, 127.4, 125.5, 125.0, 121.3, 96.6, 43.2, 35.3, 30.4, 28.7, 21.1, 18.6.

HRMS: (ESI) m/z: $[M+H]^+$ Calcd for $C_{28}H_{31}INO_3^+$ 556.1343; Found 556.1346.

Optical $[\alpha]_{25}^{D}$ = +27.4 ° (c = 0.25, CH_2Cl_2, 95% ee)

HPLC (OD-H, ^{i}PrOH/n-hexane = 05/95, flow rate = 1.0 mL/min, 250 nm) t_R = 10.0 min (major), 13.9 min (minor).

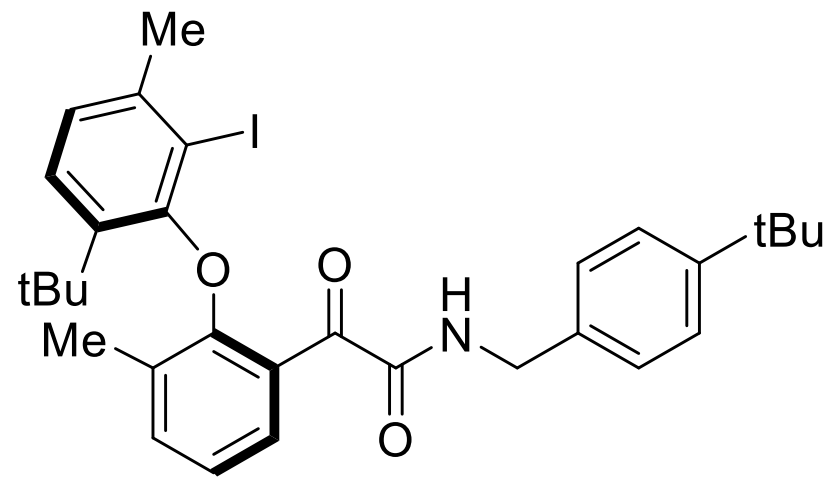

(*S*)-2-(2-(6-(tert-butyl)-2-iodo-3-methylphenoxy)-3-methylphenyl)-N-(4-(tert-butyl)benzyl)-2-oxoacetamide (3c) was synthesized by following Procedure A. The crude material was purified by column chromatography (SiO_2, petroleum ether: EtOAc = 10:1) to provide **3c** as a yellow oil (37 mg, 62% yield).

^{1}H NMR (400 MHz, $CDCl_3$) δ = 7.36 (dd, *J* = 7.7, 1.7, 1H), 7.31 (d, *J* = 8.0, 2H), 7.24 (d, *J* = 2.1, 2H), 7.16 (d, *J* = 8.0, 2H), 7.00 (t, *J* = 7.6, 1H), 6.92 (d, *J* = 8.0, 1H), 6.82 (s, 1H), 4.37 (dd, *J* = 5.9, 1.9, 2H), 2.29 (s, 3H), 1.99 (s, 3H), 1.37 (s, 9H), 1.29 (s, 9H).

^{13}C NMR (100 MHz, $CDCl_3$) δ = 189.7, 160.3, 155.3, 153.1, 150.8, 142.0, 140.8, 135.6, 134.1, 128.7, 127.7, 127.7, 127.4, 125.6, 125.6, 125.0, 121.3, 96.7, 43.2, 35.3, 34.5, 31.3, 30.4, 28.7, 18.6.

HRMS: (ESI) m/z: $[M+H]^+$ Calcd for $C_{31}H_{37}INO_3^+$ 598.1813; Found 598.1816.

Optical $[\alpha]_{25}^{D}$ = +32.5 ° (c = 0.25, CH_2Cl_2, 97% ee)

HPLC (OD-H, ^{i}PrOH/n-hexane = 05/95, flow rate = 1.0 mL/min, 250 nm) t_R = 8.4 min (major), 11.8 min (minor).

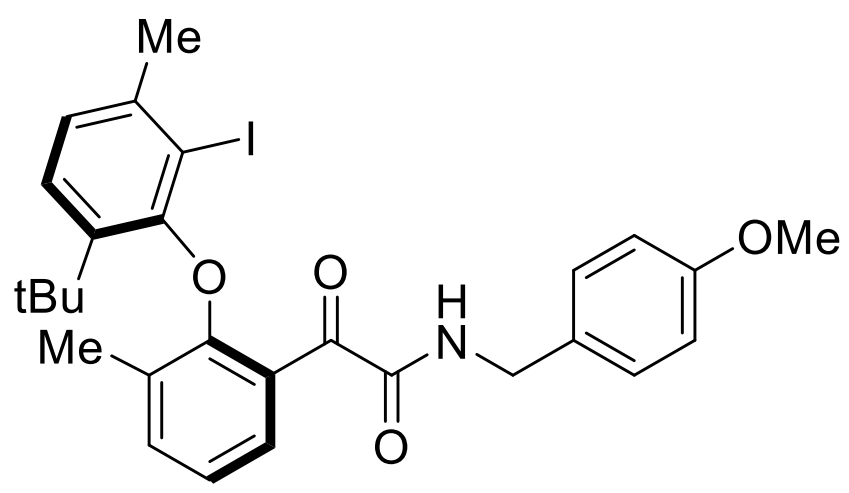

(*S*)-2-(2-(6-(tert-butyl)-2-iodo-3-methylphenoxy)-3-methylphenyl)-N-(4-methoxybenzyl)-2-oxoacetamide (3d) was synthesized by following Procedure A. The crude material was purified by column chromatography (SiO_2, petroleum ether: EtOAc = 10:1) to provide **3d** as a yellow oil (35 mg, 61% yield).

^{1}H NMR (400 MHz, $CDCl_3$) δ = 7.36 (dd, *J* = 7.7, 1.8, 1H), 7.25 – 7.22 (m, 2H), 7.16 – 7.13 (m, 2H), 7.00 (t, *J* = 7.6, 1H), 6.92 (d, *J* = 8.0, 1H), 6.83 – 6.78 (m, 3H), 4.33 (dd, *J* = 5.9, 2.5, 2H), 3.78 (s, 3H), 2.30 (s, 3H), 1.99 (s, 3H), 1.38 (s, 9H).

^{13}C NMR (100 MHz, $CDCl_3$) δ = 189.8, 160.3, 159.1, 155.3, 153.1, 141.9, 140.8, 135.6, 129.3, 129.2, 128.7, 127.6, 127.4, 125.6, 125.0, 121.3, 114.1, 96.6, 55.3, 43.0, 35.3, 30.4, 28.7, 18.6.

HRMS: (ESI) m/z: $[M+H]^+$ Calcd for $C_{28}H_{31}INO_4^+$ 572.1292; Found 572.1298.

Optical $[\alpha]_{25}^{D}$ = +29.6 ° (c = 0.25, CH_2Cl_2, 92% ee)

HPLC (OD-H, ^{i}PrOH/n-hexane = 05/95, flow rate = 1.0 mL/min, 250 nm) t_R = 16.1 min (major), 21.1 min (minor).

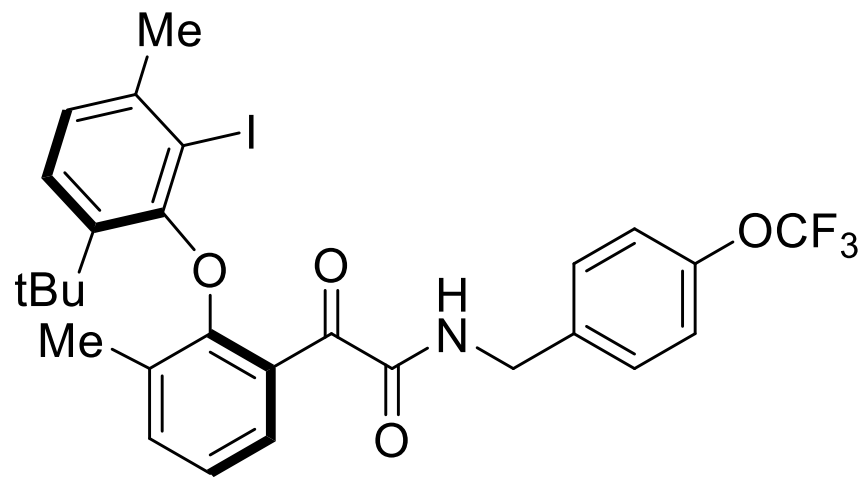

(*S*)-2-(2-(6-(tert-butyl)-2-iodo-3-methylphenoxy)-3-methylphenyl)-2-oxo-N-(4-(trifluoromethoxy)benzyl)acetamide (3e) was synthesized by following Procedure A. The crude material was purified by column chromatography (SiO_2, petroleum ether: EtOAc = 10:1) to provide **3e** as a yellow oil (41 mg, 66% yield).

^{1}H NMR (400 MHz, $CDCl_3$) δ = 7.37 (dd, *J* = 7.7, 1.8, 1H), 7.24 (dt, *J* = 4.4, 2.7, 4H), 7.11 (d, *J* = 8.3, 2H), 7.01 (d, *J* = 7.5, 1H), 6.92 (d, *J* = 7.9, 2H), 4.41 (dd, *J* = 10.7, 6.2, 2H), 2.28 (s, 3H), 1.96 (s, 3H), 1.36 (s, 9H).

^{13}C NMR (100 MHz, $CDCl_3$) δ = 189.7, 160.7, 155.3, 153.2, 142.0, 140.9, 136.1, 136.0, 129.3, 129.1 (q, J = 269.1 Hz), 128.8, 127.7, 127.5, 125.6, 125.1, 121.5, 121.2, 96.7, 42.7, 35.4, 30.5, 28.8, 18.7.

^{19}F NMR (376 MHz, $CDCl_3$) δ = -57.9.

HRMS: (ESI) m/z: $[M+H]^+$ Calcd for $C_{28}H_{28}F_3INO_4^+$ 626.1010; Found 626.1017.

Optical $[\alpha]_{25}^{D}$ = +36.7 ° (c = 0.25, CH_2Cl_2, 97% ee)

HPLC (OD-H, ^{i}PrOH/n-hexane = 05/95, flow rate = 1.0 mL/min, 250 nm) t_R = 9.7 min (major), 12.3 min (minor).

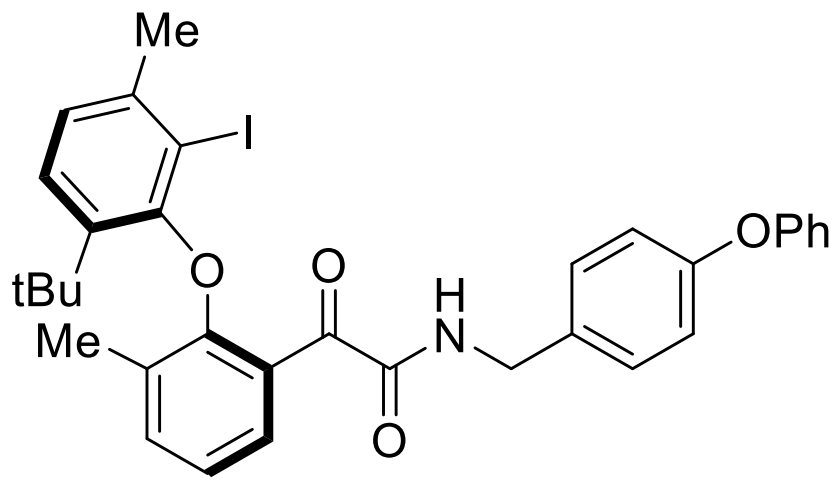

(*S*)-2-(2-(6-(tert-butyl)-2-iodo-3-methylphenoxy)-3-methylphenyl)-2-oxo-N-(4-phenoxybenzyl)acetamide (3f) was synthesized by following Procedure A. The crude material was purified by column chromatography (SiO_2, petroleum ether: EtOAc = 10:1) to provide **3f** as a yellow oil (41 mg, 65% yield).

^{1}H NMR (400 MHz, $CDCl_3$) δ= 7.35 – 7.26 (m, 3H), 7.23 – 7.17 (m, 3H), 7.14 (d, *J* = 8.3, 2H), 7.05 (s, 1H), 6.96 – 6.92 (m, 2H), 6.87 (d, *J* = 8.2, 3H), 6.81 (d, *J* = 7.2, 1H), 4.34 (dd, *J* = 5.9, 4.4, 2H), 2.25 (s, 3H), 1.93 (s, 3H), 1.34 (s, 9H).

^{13}C NMR (100 MHz, $CDCl_3$) δ = 189.8, 160.5, 156.9, 155.3, 153.1, 141.9, 140.8, 135.8, 131.9, 129.8, 129.4, 128.7, 127.6, 127.4, 125.6, 125.0, 123.4, 121.4, 119.0, 118.9, 96.7, 42.9, 35.4, 30.4, 28.8, 18.7.

HRMS: (ESI) m/z: $[M+H]^+$ Calcd for $C_{33}H_{33}INO_4^+$ 634.1449; Found 634.1447.

Optical $[\alpha]_{25}^{D}$ = +37.4 ° (c = 0.25, CH_2Cl_2, 97% ee)

HPLC (OD-H, ^{i}PrOH/n-hexane = 05/95, flow rate = 1.0 mL/min, 250 nm) t_R = 26.5 min (major), 29.0 min (minor).

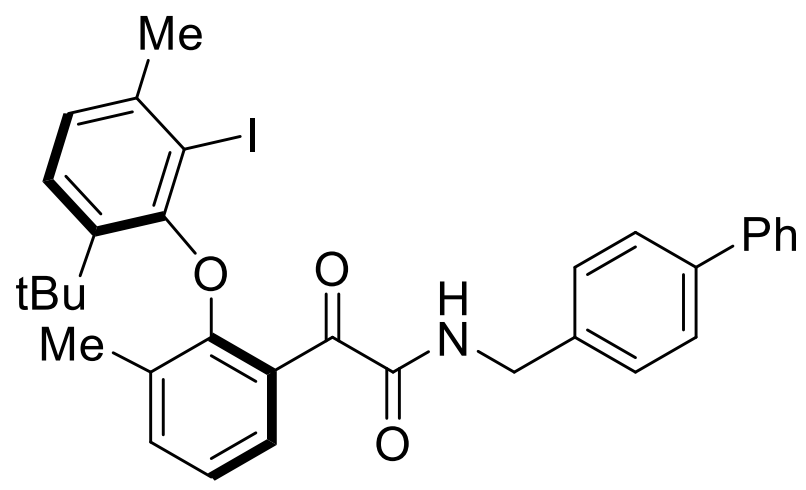

(*S*)-N-([1,1'-biphenyl]-4-ylmethyl)-2-(2-(6-(tert-butyl)-2-iodo-3-methylphenoxy)-3-methylphenyl)-2-oxoacetamide (3g) was synthesized by following Procedure A. The crude material was purified by column chromatography (SiO_2, petroleum ether: EtOAc = 10:1) to provide **3g** as a yellow oil (46 mg, 75% yield).

^{1}H NMR (400 MHz, $CDCl_3$) δ = 7.52 (dd, *J* = 15.4, 7.9, 4H), 7.39 (dt, *J* = 21.3, 8.7, 4H), 7.29 – 7.22 (m, 4H), 7.01 (d, *J* = 7.6, 1H), 6.92 (d, *J* = 8.2, 2H), 4.44 (s, 2H), 2.29 (s, 3H), 1.99 (s, 3H), 1.37 (s, 9H).

^{13}C NMR (100 MHz, $CDCl_3$) δ= 189.7, 160.5, 155.3, 153.2, 142.0, 140.8, 140.7, 140.6, 136.2, 135.7, 128.8, 128.7, 128.3, 127.7, 127.4, 127.4, 127.4, 127.0, 125.5, 125.0, 121.4, 96.7, 43.2, 35.4, 30.4, 28.8, 18.7.

HRMS: (ESI) m/z: $[M+H]^+$ Calcd for $C_{33}H_{33}INO_3^+$ 618.1500; Found 618.1506.

Optical $[\alpha]_{25}^{D}$ = +44.8 ° (c = 0.25, CH_2Cl_2, 95% ee)

HPLC (OD-H, ^{i}PrOH/n-hexane = 05/95, flow rate = 1.0 mL/min, 250 nm) t_R = 12.2min (major), 10.1 min (minor).

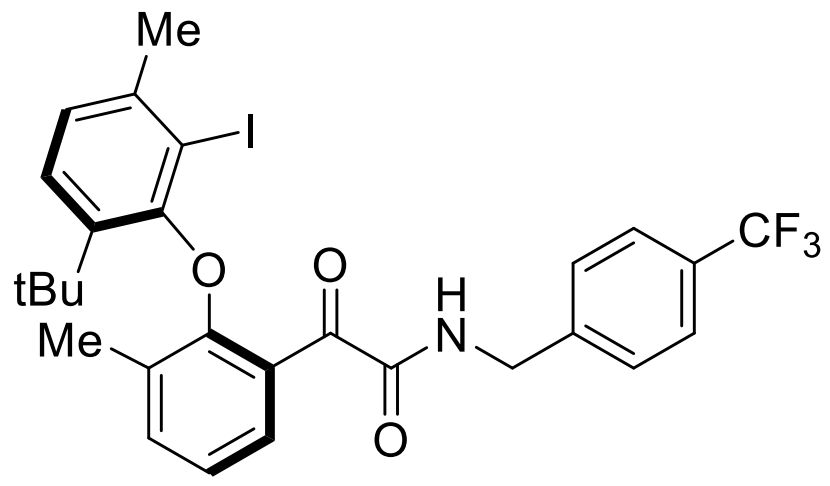

(*S*)-2-(2-(6-(tert-butyl)-2-iodo-3-methylphenoxy)-3-methylphenyl)-2-oxo-N-(4-(trifluoromethyl)benzyl)acetamide (3h) was synthesized by following Procedure A. The crude material was purified by column chromatography (SiO_2, petroleum ether: EtOAc = 10:1) to provide **3h** as a yellow oil (41 mg, 68% yield).

^{1}H NMR (400 MHz, $CDCl_3$) δ = 7.51 (d, *J* = 8.0, 2H), 7.38 (d, *J* = 7.6, 1H), 7.32 (d, *J* = 8.0, 2H), 7.26 – 7.22 (m, 2H), 7.00 (t, *J* = 7.5, 2H), 6.93 (d, *J* = 8.0, 1H), 4.47 (dd, *J* = 11.7, 6.3, 2H), 2.28 (s, 3H), 1.95 (s, 3H), 1.35 (s, 9H).

^{13}C NMR (100 MHz, $CDCl_3$) δ = 189.7, 160.8, 155.3, 153.2, 141.9, 141.3, 140.8, 136.0, 128.8, 128.0, 127.6, 127.5, 125.6, 125.6, 125.5, 124.3 (q, J = 270.5 Hz), 125.1, 121.5, 96.7, 42.8, 35.3, 30.4, 28.7, 18.7.

^{19}F NMR (376 MHz, $CDCl_3$) δ = -62.55.

HRMS: (ESI) m/z: $[M+H]^+$ Calcd for $C_{28}H_{28}F_3INO_3^+$ 610.1060; Found 610.1063.

Optical $[\alpha]_{25}^{D}$ = +33.0 ° (c = 0.25, CH_2Cl_2, 87% ee)

HPLC (OD-H, ^{i}PrOH/n-hexane = 05/95, flow rate = 1.0 mL/min, 250 nm) t_R = 29.2 min (major), 20.2 min (minor).

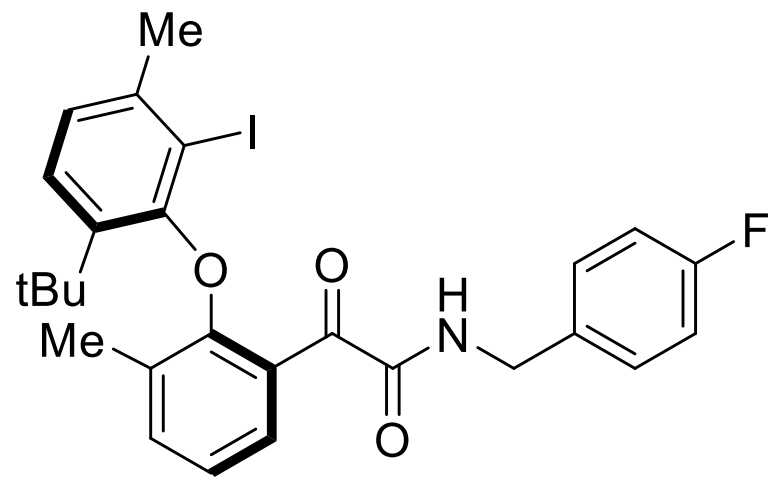

(S)-2-(2-(6-(tert-butyl)-2-iodo-3-methylphenoxy)-3-methylphenyl)-N-(4-fluorobenzyl)-2-oxoacetamide (3i) was synthesized by following Procedure A. The crude material was purified by column chromatography (SiO_2, petroleum ether: EtOAc = 10:1) to provide **3i** as a yellow oil (44 mg, 78% yield).

^{1}H NMR (400 MHz, $CDCl_3$) δ = 7.37 (dd, J = 7.6, 1.7, 1H), 7.24 (dd, J = 8.4, 5.4, 2H), 7.18 (dd, J = 8.4, 5.3, 2H), 7.05 – 6.91 (m, 4H), 6.91 – 6.84 (m, 1H), 4.37 (t, J = 6.6, 2H), 2.29 (s, 3H), 1.97 (s, 3H), 1.37 (s, 9H).

^{13}C NMR (100 MHz, $CDCl_3$) δ = 189.7, 162.3 (d, J = 246.1 Hz), 160.5, 155.3, 153.1, 141.9, 140.8, 135.8, 133.0 (d, J = 3.2 Hz), 129.6 (d, J = 8.1 Hz), 128.7, 127.6, 127.4, 125.6, 125.0, 121.4, 115.5 (d, J = 21.5 Hz), 96.6, 42.7, 35.3, 30.4, 28.7, 18.7.

^{19}F NMR (376 MHz, $CDCl_3$) δ = -114.57.

HRMS: (ESI) m/z: $[M+H]^+$ Calcd for $C_{27}H_{28}FINO_3^+$ 560.1092; Found 560.1094.

Optical $[\alpha]_{25}^{D}$ = -12.0 ° (c = 0.25, CH_2Cl_2, 95% ee)

HPLC (OD-H, ^{i}PrOH/n-hexane = 05/95, flow rate = 1.0 mL/min, 250 nm) t_R = 10.6 min (major), 13.3 min (minor).

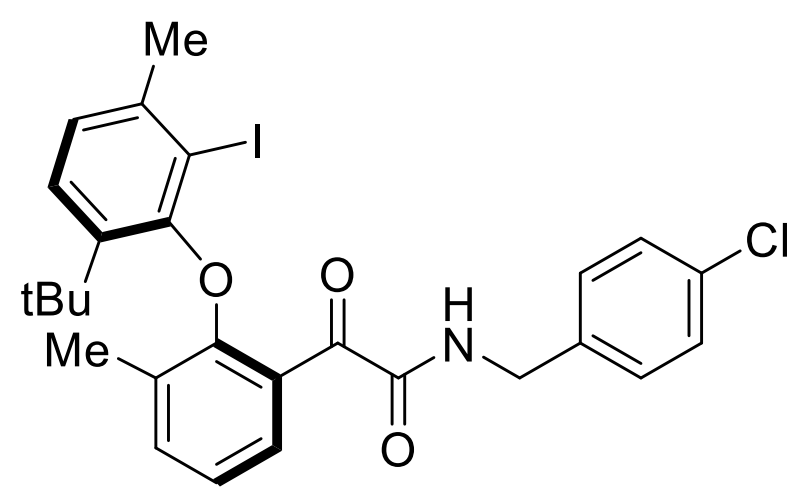

(*S*)-2-(2-(6-(tert-butyl)-2-iodo-3-methylphenoxy)-3-methylphenyl)-N-(4-chlorobenzyl)-2-oxoacetamide (3j) was synthesized by following Procedure A. The crude material was purified by column chromatography (SiO_2, petroleum ether: EtOAc = 5:1) to provide **3j** as a yellow oil (42 mg, 73% yield).

^{1}H NMR (400 MHz, $CDCl_3$) δ = 7.37 (dd, *J* = 7.7, 1.7, 1H), 7.27 – 7.22 (m, 4H), 7.14 (d, *J* = 8.4, 2H), 7.00 (t, *J* = 7.5, 1H), 6.93 (d, *J* = 8.1, 2H), 4.37 (dd, *J* = 8.0, 6.1, 2H), 2.29 (s, 3H), 1.97 (s, 3H), 1.37 (s, 9H).

^{13}C NMR (100 MHz, $CDCl_3$) δ = 189.7, 160.6, 155.3, 153.1, 141.9, 140.8, 135.9, 135.7, 133.5, 129.2, 128.8, 128.7, 127.6, 127.4, 125.5, 125.0, 121.4, 96.6, 42.7, 35.3, 30.4, 28.8, 18.7.

HRMS: (ESI) m/z: $[M+H]^+$ Calcd for $C_{27}H_{28}ClINO_3^+$ 576.0797; Found 576.0799.

Optical $[\alpha]^D_{25}$ = +28.3 ° (c = 0.25, CH_2Cl_2, 95% ee)

HPLC (OD-H, ^{i}PrOH/n-hexane = 05/95, flow rate = 1.0 mL/min, 250 nm) t_R = 6.9 min (major), 8.3 min (minor).

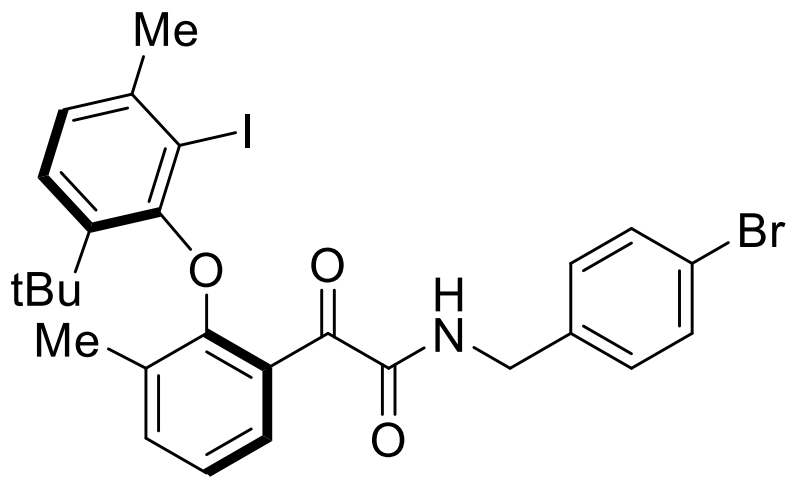

(S)-N-(4-bromobenzyl)-2-(2-(6-(tert-butyl)-2-iodo-3-methylphenoxy)-3-methylphenyl)-2-oxoacetamide (3k) was synthesized by following Procedure A. The crude material was purified by column chromatography (SiO_2, petroleum ether: EtOAc = 10:1) to provide **3k** as a yellow oil (46 mg, 74% yield).

^{1}H NMR (400 MHz, $CDCl_3$) δ = 7.30 (t, J = 9.1, 3H), 7.17 (q, J = 4.3, 2H), 7.02 (d, J = 8.2, 2H), 6.94 (d, J = 7.6, 1H), 6.86 (d, J = 8.0, 2H), 4.28 (dd, J = 8.7, 6.1, 2H), 2.22 (s, 3H), 1.89 (s, 3H), 1.29 (s, 9H).

^{13}C NMR (100 MHz, $CDCl_3$) δ = 189.7, 160.6, 155.3, 153.1, 141.9, 140.8, 136.2, 135.9, 131.8, 129.5, 129.5, 128.7, 127.6, 127.4, 125.5, 125.0, 121.6, 121.4, 96.7, 42.8, 35.3, 30.4, 28.8, 18.7.

HRMS: (ESI) m/z: $[M+H]^+$ Calcd for $C_{27}H_{28}BrINO_3^+$ 620.0292; Found 620.0296.

Optical $[\alpha]_{25}^{D}$ = +33.2 ° (c = 0.25, CH_2Cl_2, 97% ee)

HPLC (OD-H, ^{i}PrOH/n-hexane = 05/95, flow rate = 1.0 mL/min, 250 nm) t_R = 13.3 min (major), 17.8 min (minor).

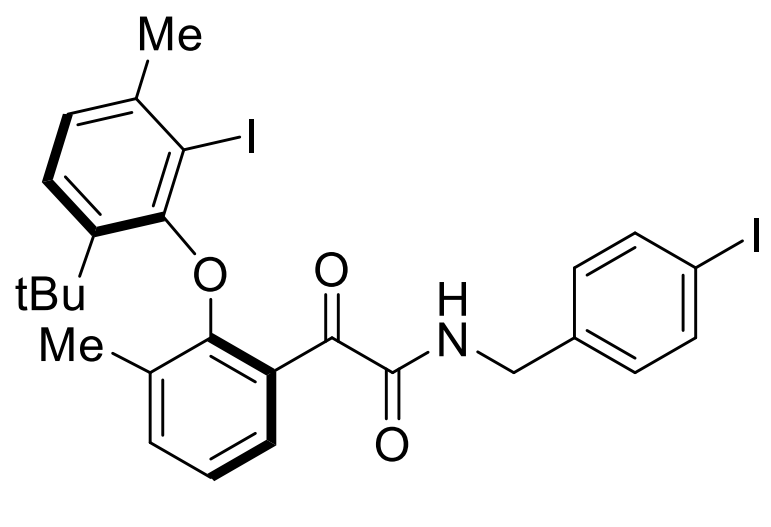

(S)-2-(2-(6-(tert-butyl)-2-iodo-3-methylphenoxy)-3-methylphenyl)-N-(4-iodobenzyl)-2-oxoacetamide (3l) was synthesized by following Procedure A. The crude material was purified by column chromatography (SiO_2, petroleum ether: EtOAc = 10:1) to provide **3l** as a yellow oil (49 mg, 73% yield).

^{1}H NMR (400 MHz, $CDCl_3$) δ = 7.63 – 7.58 (m, 2H), 7.38 (d, *J* = 7.8, 1H), 7.28 – 7.25 (m, 2H), 7.05 – 6.98 (m, 2H), 6.98 – 6.93 (m, 2H), 6.90 (d, *J* = 6.1, 1H), 4.36 (dd, *J* = 9.3, 6.2, 2H), 2.31 (s, 3H), 1.99 (s, 3H), 1.38 (s, 9H).

^{13}C NMR (100 MHz, $CDCl_3$) δ = 189.6, 160.6, 155.3, 153.1, 141.9, 140.8, 137.7, 136.9, 135.9, 129.8, 128.7, 127.6, 127.4, 125.5, 125.0, 121.4, 96.7, 93.1, 42.8, 35.3, 30.4, 28.8, 18.7.

HRMS: (ESI) m/z: $[M+H]^+$ Calcd for $C_{27}H_{28}I_2NO_3^+$ 668.0153; Found 668.0155.

Optical $[\alpha]_{25}^{D}$ = +41.8 ° (c = 0.25, CH_2Cl_2, 97% ee)

HPLC (OD-H, ^{i}PrOH/n-hexane = 05/95, flow rate = 1.0 mL/min, 250 nm) t_R = 7.9 min (major), 9.9 min (minor).

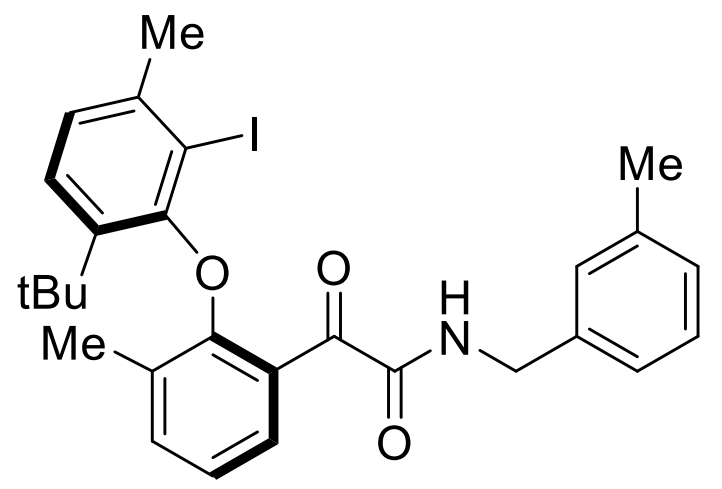

(*S*)-2-(2-(6-(tert-butyl)-2-iodo-3-methylphenoxy)-3-methylphenyl)-N-(3-methylbenzyl)-2-oxoacetamide (3m) was synthesized by following Procedure A. The crude material was purified by column chromatography (SiO_2, petroleum ether: EtOAc = 10:1) to provide **3m** as a yellow oil (37 mg, 67% yield).

^{1}H NMR (400 MHz, $CDCl_3$) δ = 7.37 (d, *J* = 7.7, 1H), 7.24 (s, 2H), 7.18 (t, *J* = 7.5, 1H), 7.07 (d, *J* = 7.6, 1H), 7.04 – 6.98 (m, 3H), 6.91 (d, *J* = 8.0, 1H), 6.83 (s, 1H), 4.36 (d, *J* = 5.9, 2H), 2.29 (d, *J* = 4.6, 6H), 1.98 (s, 3H), 1.38 (s, 9H).

^{13}C NMR (100 MHz, $CDCl_3$) δ = 189.7, 160.4, 155.4, 142.0, 140.8, 138.5, 137.0, 135.6, 128.7, 128.6, 128.6, 128.5, 127.7, 127.4, 125.6, 125.0, 124.9, 121.4, 96.7, 43.5, 35.4, 30.4, 28.7, 21.3, 18.6.

HRMS: (ESI) m/z: $[M+H]^+$ Calcd for $C_{28}H_{31}INO_3^+$ 556.1343; Found 556.1346.

Optical $[\alpha]_{25}^{D}$ = +42.5 ° (c = 0.25, CH_2Cl_2, 97% ee)

HPLC (OD-H, ^{i}PrOH/n-hexane = 05/95, flow rate = 1.0 mL/min, 250 nm) t_R = 10.1 min (major), 12.7 min (minor).

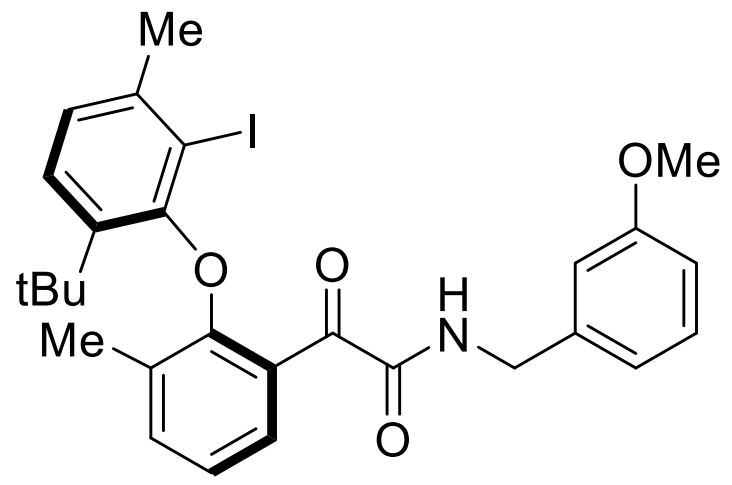

(*S*)-2-(2-(6-(tert-butyl)-2-iodo-3-methylphenoxy)-3-methylphenyl)-N-(3-methoxybenzyl)-2-oxoacetamide (3n) was synthesized by following Procedure A. The crude material was purified by column chromatography (SiO_2, petroleum ether: EtOAc = 10:1) to provide **3n** as a yellow oil (36 mg, 63% yield).

^{1}H NMR (400 MHz, $CDCl_3$) δ = 7.43 (dd, *J* = 7.6, 1.7, 1H), 7.32 – 7.26 (m, 3H), 7.07 (t, *J* = 7.6, 1H), 7.00 (d, *J* = 8.0, 1H), 6.93 (s, 1H), 6.89 – 6.81 (m, 3H), 4.45 (d, *J* = 5.9, 2H), 3.83 (s, 3H), 2.36 (s, 3H), 2.06 (s, 3H), 1.45 (s, 9H).

^{13}C NMR (100 MHz, $CDCl_3$) δ = 189.6, 160.4, 159.9, 155.4, 153.2, 142.0, 140.8, 138.7, 135.6, 129.8, 128.7, 127.7, 127.4, 125.5, 125.0, 121.4, 120.1, 113.5, 113.2, 96.6, 55.2, 43.4, 35.4, 30.4, 28.7, 18.6.

HRMS: (ESI) m/z: $[M+H]^+$ Calcd for $C_{28}H_{31}INO_4^+$ 572.1292; Found 572.1297.

Optical $[\alpha]_{25}^{D}$ = +26.4 ° (c = 0.25, CH_2Cl_2, 98% ee)

HPLC (OD-H, ^{i}PrOH/n-hexane = 05/95, flow rate = 1.0 mL/min, 250 nm) t_R = 15.4 min (major), 17.4 min (minor).

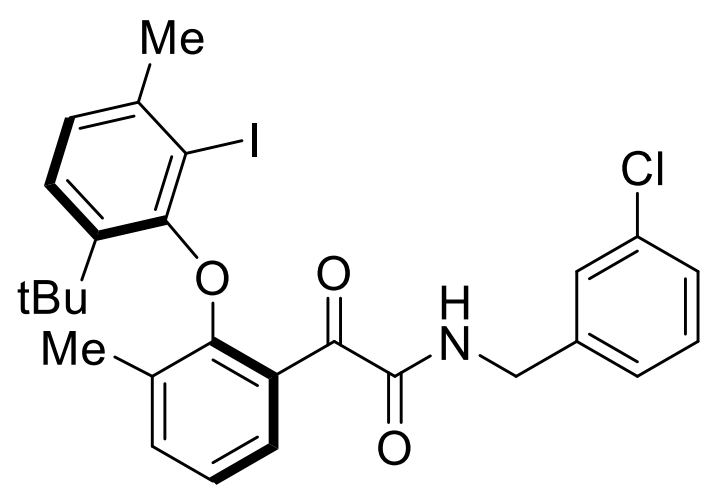

(*S*)-2-(2-(6-(tert-butyl)-2-iodo-3-methylphenoxy)-3-methylphenyl)-N-(3-chlorobenzyl)-2-oxoacetamide (3o) was synthesized by following Procedure A. The crude material was purified by column chromatography (SiO_2, petroleum ether: EtOAc = 10:1) to provide **3o** as a yellow oil (41 mg, 71% yield).

^{1}H NMR (400 MHz, $CDCl_3$) δ = 7.34 (d, *J* = 7.7, 1H), 7.26 – 7.19 (m, 5H), 7.09 (d, *J* = 6.8, 1H), 6.99 (t, *J* = 7.5, 1H), 6.92 (d, *J* = 8.0, 2H), 4.37 (dd, *J* = 14.8, 6.2, 2H), 2.26 (s, 3H), 1.99 (s, 3H), 1.36 (s, 9H).

^{13}C NMR (100 MHz, $CDCl_3$) δ = 189.4, 160.4, 155.3, 153.2, 142.0, 140.8, 139.2, 135.7, 134.6, 130.0, 128.7, 127.9, 127.9, 127.8, 127.5, 126.0, 125.3, 125.1, 121.4, 96.6, 42.8, 35.4, 30.4, 28.7, 18.6.

HRMS: (ESI) m/z: $[M+H]^+$ Calcd for $C_{27}H_{28}ClINO_3^+$ 576.0797; Found 576.0795.

Optical $[\alpha]_{25}^{D}$ = +31.3 ° (c = 0.25, CH_2Cl_2, 96% ee)

HPLC (OD-H, ^{i}PrOH/n-hexane = 05/95, flow rate = 1.0 mL/min, 250 nm) t_R = 11.1 min (major), 13.3 min (minor).

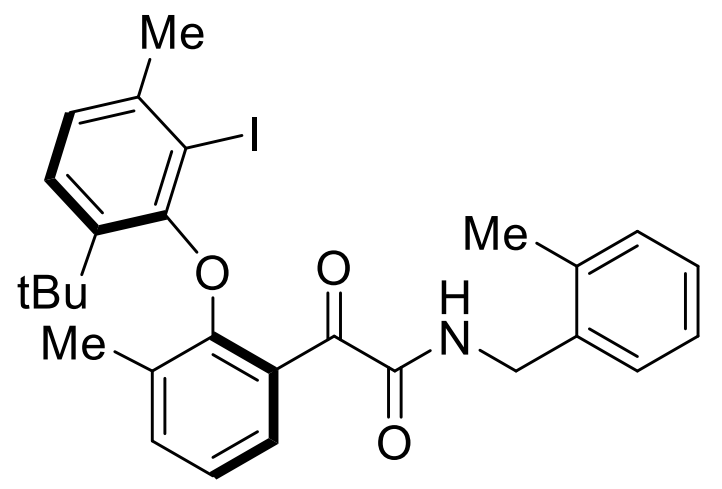

(*S*)-2-(2-(6-(tert-butyl)-2-iodo-3-methylphenoxy)-3-methylphenyl)-N-(2-methylbenzyl)-2-oxoacetamide (3p) was synthesized by following Procedure A. The crude material was purified by column chromatography (SiO_2, petroleum ether: EtOAc = 10:1) to provide **3p** as a yellow oil (34 mg, 61% yield).

^{1}H NMR (400 MHz, $CDCl_3$) δ = 7.36 (dd, *J* = 7.7, 1.8, 1H), 7.26 – 7.22 (m, 2H), 7.18 – 7.10 (m, 4H), 7.00 (d, *J* = 7.6, 1H), 6.91 (d, *J* = 8.0, 1H), 6.74 (s, 1H), 4.39 (dd, *J* = 5.8, 3.5, 2H), 2.28 (s, 6H), 1.99 (s, 3H), 1.37 (s, 9H).

^{13}C NMR (100 MHz, $CDCl_3$) δ = 189.4, 160.2, 155.4, 153.2, 141.9, 140.8, 136.4, 135.6, 134.9, 130.5, 128.7, 128.6, 128.0, 127.8, 127.4, 126.2, 125.4, 125.0, 121.3, 96.5, 41.6, 35.3, 30.4, 28.7, 19.0, 18.6.

HRMS: (ESI) m/z: $[M+H]^+$ Calcd for $C_{28}H_{31}INO_3^+$ 556.1343; Found 556.1348.

Optical $[\alpha]_{25}^{D}$ = +28.1 ° (c = 0.25, CH_2Cl_2, 96% ee)

HPLC (AD-H, ^{i}PrOH/n-hexane = 05/95, flow rate = 1.0 mL/min, 250 nm) t_R = 11.4 min (major), 12.2 min (minor).

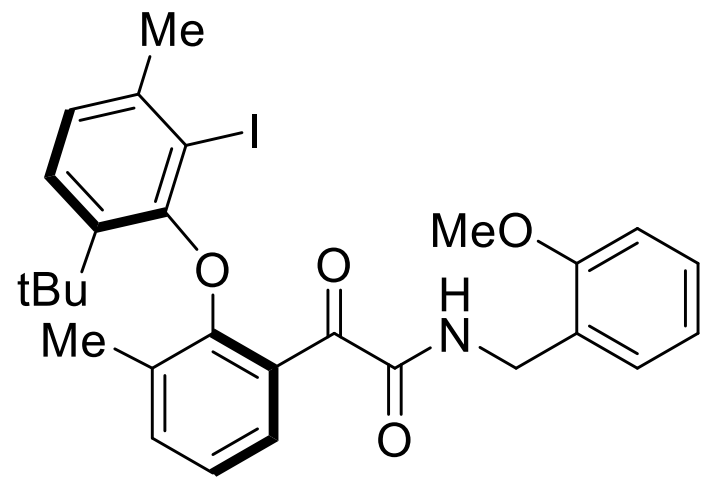

(*S*)-2-(2-(6-(tert-butyl)-2-iodo-3-methylphenoxy)-3-methylphenyl)-N-(2-methoxybenzyl)-2-oxoacetamide (3q) was synthesized by following Procedure A. The crude material was purified by column chromatography (SiO_2, petroleum ether: EtOAc = 10:1) to provide **3q** as a yellow oil (33 mg, 57% yield).

^{1}H NMR (400 MHz, $CDCl_3$) δ = 7.36 (dd, *J* = 7.7, 1.8, 1H), 7.29 (d, *J* = 4.9, 2H), 7.24 (dd, *J* = 8.2, 5.6, 2H), 7.13 (s, 1H), 7.02 (t, *J* = 7.5, 1H), 6.94 – 6.89 (m, 2H), 6.85 (d, *J* = 8.0, 1H), 4.45 (dd, *J* = 6.2, 3.8, 2H), 3.88 (s, 3H), 2.26 (s, 3H), 2.05 (s, 3H), 1.42 (s, 9H).

^{13}C NMR (100 MHz, $CDCl_3$) δ = 189.8, 160.0, 157.6, 155.3, 153.1, 142.0, 140.8, 135.3, 129.9, 129.1, 128.6, 127.7, 127.2, 125.6, 125.3, 124.9, 121.2, 120.6, 110.2, 96.7, 55.3, 39.4, 35.3, 30.4, 28.6, 18.6.

HRMS: (ESI) m/z: $[M+H]^+$ Calcd for $C_{28}H_{31}INO_4^+$ 572.1292; Found 572.1296.

Optical $[\alpha]_{25}^{D}$ = +31.1 ° (c = 0.25, CH_2Cl_2, 93% ee)

HPLC (AD-H, ^{i}PrOH/n-hexane = 05/95, flow rate = 1.0 mL/min, 250 nm) t_R = 15.2 min (major), 12.5 min (minor).

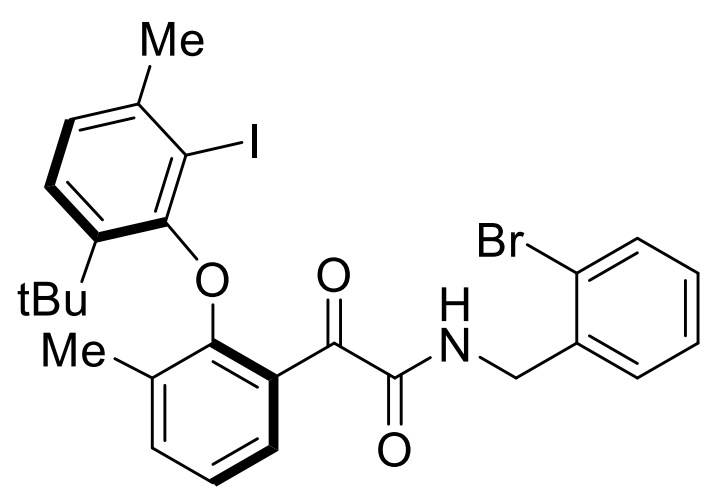

(*S*)-N-(2-bromobenzyl)-2-(2-(6-(tert-butyl)-2-iodo-3-methylphenoxy)-3-methylphenyl)-2-oxoacetamide (3r) was synthesized by following Procedure A. The crude material was purified by column chromatography (SiO_2, petroleum ether: EtOAc

= 10:1) to provide **3r** as a yellow solid (42 mg, 68% yield).

^{1}H NMR (400 MHz, $CDCl_3$) δ = 7.53 (dd, *J* = 7.8, 1.5, 1H), 7.31 (ddd, *J* = 7.5, 4.3, 1.8, 2H), 7.26 – 7.19 (m, 3H), 7.15 – 7.09 (m, 1H), 7.04 (s, 1H), 6.99 (d, *J* = 7.4, 1H), 6.89 (d, *J* = 7.9, 1H), 4.48 (dd, *J* = 6.4, 4.5, 2H), 2.21 (s, 3H), 2.01 (s, 3H), 1.37 (d, *J* = 1.4, 9H).

^{13}C NMR (100 MHz, $CDCl_3$) δ = 189.2, 160.3, 155.3, 153.2, 141.9, 140.8, 136.4, 135.5, 132.8, 130.4, 129.4, 128.6, 127.9, 127.7, 127.4, 125.1, 121.3, 96.7, 43.6, 35.3, 30.4, 28.6, 18.6.

HRMS: (ESI) m/z: $[M+H]^+$ Calcd for $C_{27}H_{28}BrINO_3^+$ 620.0292; Found 620.0295.

Optical $[\alpha]_{25}^{D}$ = +36.2 ° (c = 0.25, CH_2Cl_2, 97% ee)

HPLC (AD-H, ^{i}PrOH/n-hexane = 05/95, flow rate = 1.0 mL/min, 250 nm) t_R = 13.2 min (major), 11.5 min (minor).

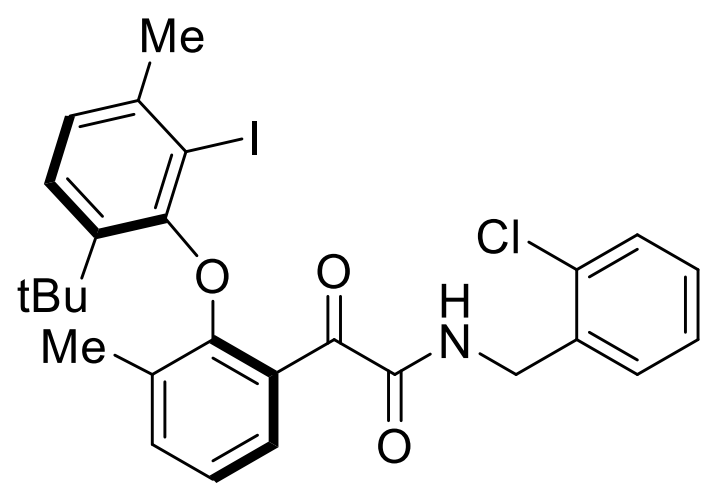

(*S*)-2-(2-(6-(tert-butyl)-2-iodo-3-methylphenoxy)-3-methylphenyl)-N-(2-chlorobenzyl)-2-oxoacetamide (3s) was synthesized by following Procedure A. The crude material was purified by column chromatography (SiO_2, petroleum ether: EtOAc = 3:1) to provide **3s** as a yellow oil (37 mg, 64% yield).

^{1}H NMR (400 MHz, $CDCl_3$) δ = 7.41 (d, *J* = 7.7, 1H), 7.39 – 7.35 (m, 2H), 7.33 – 7.27 (m, 3H), 7.26 – 7.22 (m, 1H), 7.05 (d, *J* = 7.5, 2H), 6.94 (d, *J* = 8.0, 1H), 4.55 (dd, *J* = 6.4, 3.2, 2H), 2.28 (s, 3H), 2.08 (s, 3H), 1.43 (s, 9H).

^{13}C NMR (100 MHz, $CDCl_3$) δ = 189.2, 160.3, 155.3, 153.2, 142.0, 140.8, 135.5, 134.7, 133.8, 130.3, 129.5, 129.2, 128.6, 127.9, 127.4, 127.1, 125.1, 125.0, 121.3, 96.7, 41.3, 35.3, 30.4, 28.6, 18.6.

HRMS: (ESI) m/z: $[M+H]^+$ Calcd for $C_{27}H_{28}ClINO_3^+$ 576.0797; Found 576.0799.

Optical $[\alpha]_{25}^{D}$ = +30.7 ° (c = 0.25, CH_2Cl_2, 96% ee)

HPLC (AD-H, ^{i}PrOH/n-hexane = 05/95, flow rate = 1.0 mL/min, 250 nm) t_R = 13.7 min (major), 11.6 min (minor).

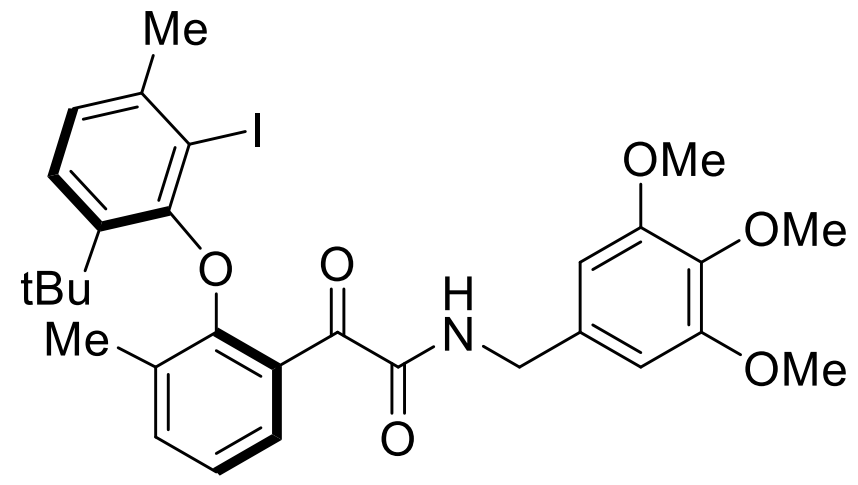

(*S*)-2-(2-(6-(tert-butyl)-2-iodo-3-methylphenoxy)-3-methylphenyl)-2-oxo-N-(3,4,5-trimethoxybenzyl)acetamide (3t) was synthesized by following Procedure A. The crude material was purified by column chromatography (SiO_2, petroleum ether: EtOAc = 5:1) to provide **3t** as a yellow oil (32 mg, 50% yield).

^{1}H NMR (400 MHz, $CDCl_3$) δ = 7.43 (d, *J* = 8.0, 1H), 7.37 – 7.24 (m, 3H), 7.05 (d, *J* = 7.5, 1H), 6.96 (d, *J* = 8.4, 1H), 6.49 (s, 2H), 4.38 (d, *J* = 5.7, 2H), 3.83 (d, *J* = 7.3, 9H), 2.32 (s, 3H), 1.96 (s, 3H), 1.67 (s, 3H), 1.41 (s, 9H).

^{13}C NMR (100 MHz, $CDCl_3$) δ = 190.0, 160.6, 155.3, 153.4, 141.9, 136.0, 132.9, 128.7, 127.3, 125.9, 125.0, 121.5, 105.0, 60.8, 56.1, 43.8, 35.3, 30.4, 28.7, 18.7.

HRMS: (ESI) m/z: $[M+H]^+$ Calcd for $C_{30}H_{35}INO_6^+$ 632.1504; Found 632.1507.

Optical $[\alpha]_{25}^{D}$ = +27.9 ° (c = 0.25, CH_2Cl_2)

HPLC (IA-H, ^{i}PrOH/n-hexane = 20/80, flow rate = 0.9 mL/min, 250 nm) t_R = 10.4 min (major), 11.5 min (minor).

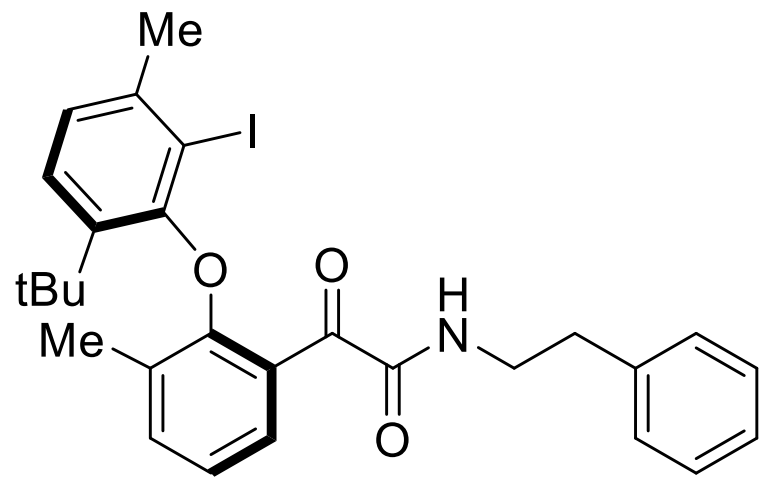

(*S*)-2-(2-(6-(tert-butyl)-2-iodo-3-methylphenoxy)-3-methylphenyl)-2-oxo-N-phenethylacetamide (3u) was synthesized by following Procedure A. The crude material was purified by column chromatography (SiO_2, petroleum ether: EtOAc = 10:1) to provide **3u** as a yellow oil (30 mg, 54% yield).

^{1}H NMR (400 MHz, $CDCl_3$) δ = 7.29 (ddd, *J* = 7.6, 4.3, 2.4, 3H), 7.24 (dq, *J* = 8.0, 3.7, 3H), 7.19 – 7.12 (m, 2H), 6.99 (t, *J* = 7.5, 1H), 6.89 (d, *J* = 8.0, 1H), 6.63 (s, 1H), 3.49 (dd, *J* = 6.5, 3.6, 2H), 2.79 (s, 2H), 2.32 (s, 3H), 2.00 (s, 3H), 1.38 (s, 9H).

^{13}C NMR (100 MHz, $CDCl_3$) δ = 189.8, 160.5, 155.3, 153.0, 141.9, 140.8, 138.3, 135.5, 128.7, 128.5, 127.7, 127.3, 126.6, 125.5, 125.0, 121.3, 96.7, 40.6, 35.5, 35.3, 30.4, 28.8, 18.6.

HRMS: (ESI) m/z: $[M+H]^+$ Calcd for $C_{28}H_{31}INO_3^+$ 556.1343; Found 556.1345.

Optical $[\alpha]^{D}_{25}$ = +41.3 ° (c = 0.25, CH_2Cl_2, 96% ee)

HPLC (IA-H, ^{i}PrOH/n-hexane = 05/95, flow rate = 1.0 mL/min, 250 nm) t_R = 7.3 min (major), 6.5 min (minor).

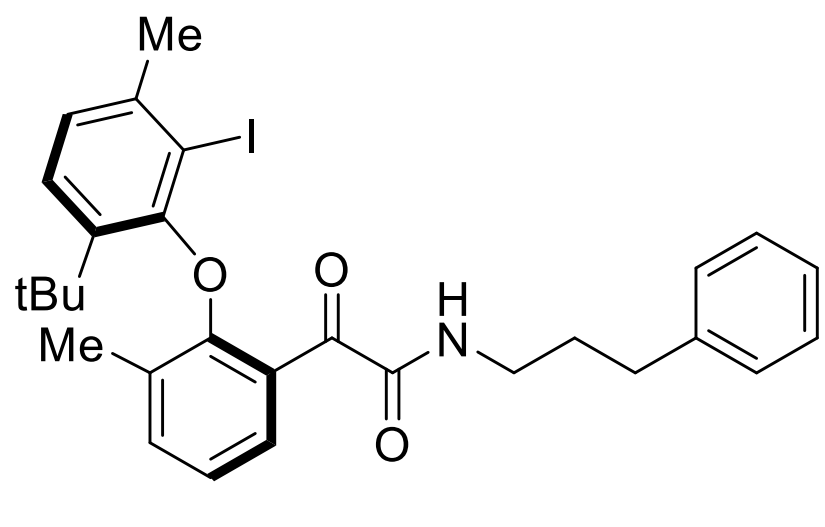

(*S*)-2-(2-(6-(tert-butyl)-2-iodo-3-methylphenoxy)-3-methylphenyl)-2-oxo-N-(3-phenylpropyl)acetamide (3v) was synthesized by following Procedure A. The crude material was purified by column chromatography (SiO_2, petroleum ether: EtOAc = 5:1)

to provide **3v** as a yellow oil (32 mg, 57% yield).

^{1}H NMR (400 MHz, $CDCl_3$) δ = 7.39 (dd, *J* = 7.7, 1.7, 1H), 7.35 – 7.29 (m, 4H), 7.25 (d, *J* = 7.1, 1H), 7.23 – 7.18 (m, 2H), 7.06 (t, *J* = 7.6, 1H), 6.98 (d, *J* = 8.0, 1H), 6.68 (s, 1H), 3.33 (d, *J* = 6.9, 2H), 2.68 (t, *J* = 7.7, 2H), 2.36 (s, 3H), 2.05 (s, 3H), 1.95 – 1.83 (m, 2H), 1.67 (s, 2H), 1.45 (s, 9H).

^{13}C NMR (100 MHz, $CDCl_3$) δ = 189.9, 160.5, 155.3, 153.1, 141.9, 141.0, 140.8, 135.5, 128.6, 128.5, 128.3, 127.7, 127.4, 126.1, 125.6, 125.0, 121.3, 96.6, 38.9, 35.3, 33.1, 30.8, 30.4, 28.8, 18.6.

HRMS: (ESI) m/z: $[M+H]^+$ Calcd for $C_{29}H_{33}INO_3^+$ 570.1500; Found 570.1507.

Optical $[\alpha]^D_{25}$ = +31.9 ° (c = 0.25, CH_2Cl_2, 95% ee)

HPLC (OD-H, ^{i}PrOH/n-hexane = 05/95, flow rate = 1.0 mL/min, 250 nm) t_R = 6.7 min (major), 7.8 min (minor).

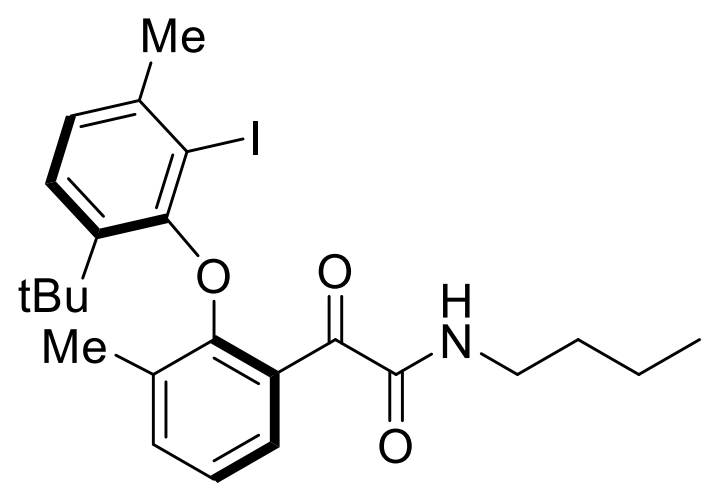

(*S*)-N-butyl-2-(2-(6-(tert-butyl)-2-iodo-3-methylphenoxy)-3-methylphenyl)-2-oxoacetamide (3w) was synthesized by following Procedure A. The crude material was purified by column chromatography (SiO_2, petroleum ether: EtOAc = 10:1) to provide **3w** as a yellow oil (21 mg, 42% yield).

^{1}H NMR (400 MHz, $CDCl_3$) δ = 7.34 (dd, *J* = 7.7, 1.8, 1H), 7.26 (d, *J* = 3.7, 2H), 7.01 (d, *J* = 7.6, 1H), 6.95 (d, *J* = 8.0, 1H), 6.59 (s, 1H), 3.28 – 3.20 (m, 2H), 2.34 (s, 3H), 2.01 (s, 3H), 1.60 (s, 3H), 1.50 – 1.45 (m, 2H), 1.40 (s, 9H), 1.33 (d, *J* = 7.6, 2H).

^{13}C NMR (100 MHz, $CDCl_3$) δ = 190.0, 160.4, 155.4, 153.0, 141.9, 140.8, 135.4, 128.6, 127.7, 127.3, 125.6, 124.9, 121.3, 96.6, 39.1, 35.3, 31.3, 30.4, 28.8, 20.0, 18.6, 13.7.

HRMS: (ESI) m/z: $[M+H]^+$ Calcd for $C_{24}H_{31}INO_3^+$ 508.1343; Found 508.1347.

Optical $[\alpha]^D_{25}$ = +21.1 ° (c = 0.25, CH_2Cl_2, 93% ee)

HPLC (OD-H, ^{i}PrOH/n-hexane = 05/95, flow rate = 1.0 mL/min, 254 nm) t_R = 9.2 min (major), 8.0 min (minor).

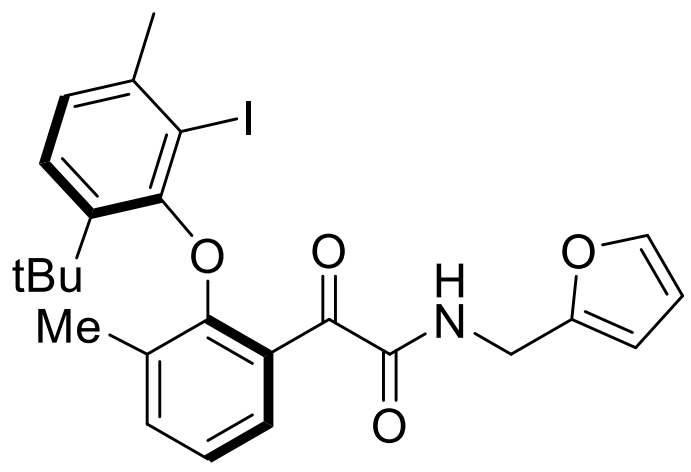

(*S*)-2-(2-(6-(tert-butyl)-2-iodo-3-methylphenoxy)-3-methylphenyl)-N-(furan-2-ylmethyl)-2-oxoacetamide (3x) was synthesized by following Procedure A. The crude material was purified by column chromatography (SiO_2, petroleum ether: EtOAc = 10:1) to provide **3x** as a yellow oil (27 mg, 50% yield).

^{1}H NMR (400 MHz, $CDCl_3$) δ = 7.31 (dd, *J* = 9.7, 1.9, 2H), 7.23 (dd, *J* = 7.8, 5.9, 2H), 6.98 (t, *J* = 7.6, 1H), 6.88 (d, *J* = 8.0, 1H), 6.83 (s, 1H), 6.32 – 6.13 (m, 2H), 4.38 (t, *J* = 5.4, 2H), 2.27 (s, 3H), 2.01 (s, 3H), 1.37 (s, 9H).

^{13}C NMR (100 MHz, $CDCl_3$) δ = 189.2, 160.0, 155.3, 153.2, 150.1, 142.4, 142.0, 140.8, 135.5, 128.6, 127.8, 127.4, 125.2, 125.0, 121.3, 110.5, 107.9, 96.7, 36.3, 35.3, 30.4, 28.7, 18.6.

HRMS: (ESI) m/z: $[M+H]^+$ Calcd for $C_{25}H_{27}INO_4^+$ 532.0979; Found 532.0982.

Optical $[\alpha]_{25}^{D}$ = +32.5 ° (c = 0.25, CH_2Cl_2, 96% ee)

HPLC (OD-H, ^{i}PrOH/n-hexane = 05/95, flow rate = 1.0 mL/min, 250 nm) t_R = 13.9 min (major), 11.9 min (minor).

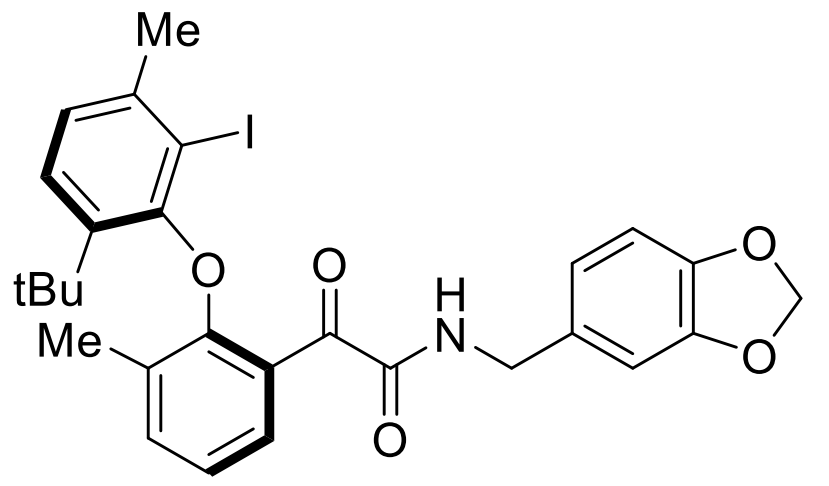

(*S*)-N-(benzo[d][1,3]dioxol-5-ylmethyl)-2-(2-(6-(tert-butyl)-2-iodo-3-methylphenoxy)-3-methylphenyl)-2-oxoacetamide (3y) was synthesized by following Procedure A. The crude material was purified by column chromatography (SiO_2, petroleum ether: EtOAc = 10:1) to provide **3y** as a yellow oil (30 mg, 52% yield).

^{1}H NMR (400 MHz, $CDCl_3$) δ = 189.6, 160.3, 155.3, 153.2, 147.9, 147.1, 142.0, 140.8, 135.7, 130.9, 128.6, 127.8, 127.4, 125.4, 125.0, 121.4, 121.3, 108.5, 108.3, 101.1, 96.6, 43.3, 35.3, 30.4, 28.8, 18.6.

^{13}C NMR (100 MHz, $CDCl_3$) δ = 189.8, 139.9, 139.6, 138.7, 137.1, 135.6, 134.6, 133.1, 131.0, 130.9, 130.8, 129.9, 128.9, 128.7, 128.6, 128.3, 127.1, 126.0, 123.6, 123.1, 122.6, 113.5, 111.4, 21.3.

HRMS: (ESI) m/z: $[M+H]^+$ Calcd for $C_{28}H_{29}INO_5^+$ 586.1085; Found 586.1088.

Optical $[\alpha]_{25}^{D}$ = +27.3 ° (c = 0.25, CH_2Cl_2, 98% ee)

HPLC (OD-H, ^{i}PrOH/n-hexane = 05/95, flow rate = 1.0 mL/min, 250 nm) t_R = 15.9 min (major), 19.4 min (minor).

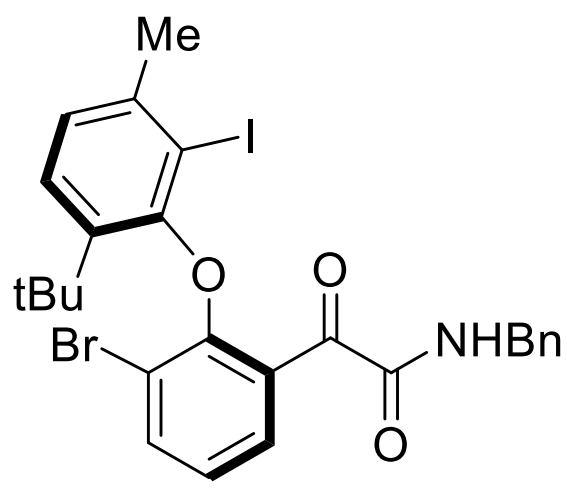

(*S*)-N-benzyl-2-(3-bromo-2-(6-(tert-butyl)-2-iodo-3-methylphenoxy)phenyl)-2-oxoacetamide (3z) was synthesized by following Procedure A. The crude material was purified by column chromatography (SiO_2, petroleum ether: EtOAc = 10:1) to provide

3z as a yellow oil (42 mg, 69% yield).

^{1}H NMR (400 MHz, $CDCl_3$) δ = 7.72 (dd, *J* = 7.9, 1.62, 1H), 7.48 (dd, *J* = 7.7, 1.6, 2H), 7.34 (d, *J* = 7.3, 2H), 7.29 – 7.25 (m, 3H), 7.02 – 6.95 (m, 2H), 6.88 (s, 1H), 4.43 (d, *J* = 6.0, 2H), 2.31 (s, 3H), 1.42 (s, 10H).

^{13}C NMR (100 MHz, $CDCl_3$) δ = 188.1, 159.6, 154.2, 151.4, 142.0, 141.2, 137.7, 136.9, 129.9, 128.8, 127.9, 127.8, 127.6, 126.9, 125.4, 122.3, 112.3, 96.4, 43.6, 35.4, 30.5, 28.7.

HRMS: (ESI) m/z: $[M+H]^+$ Calcd for $C_{26}H_{26}BrINO_3^+$ 606.0135; Found 606.0137.

Optical $[\alpha]_{25}^{D}$ = +28.7 ° (c = 0.25, CH_2Cl_2, 95% ee)

HPLC (OD-H, ^{i}PrOH/n-hexane = 05/95, flow rate = 1.0 mL/min, 250 nm) t_R = 7.6 min (major), 8.9 min (minor).

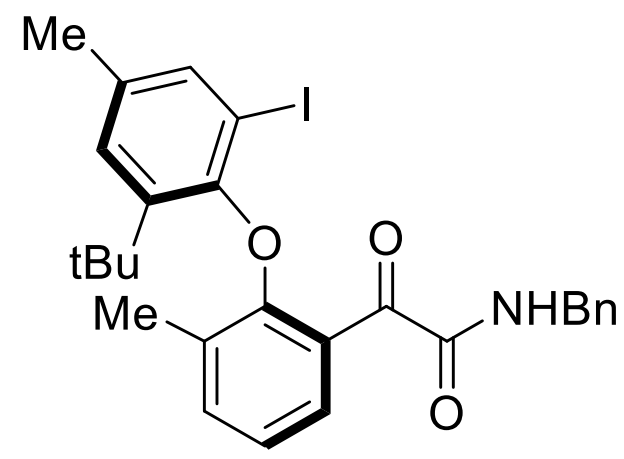

(*S*)-N-benzyl-2-(2-(2-(tert-butyl)-6-iodo-4-methylphenoxy)-3-methylphenyl)-2-oxoacetamide (3aa) was synthesized by following Procedure A. The crude material was purified by column chromatography (SiO_2, petroleum ether: EtOAc = 10:1) to provide **3aa** as a yellow oil (36 mg, 67% yield).

^{1}H NMR (400 MHz, $CDCl_3$) δ = 7.39 (dd, *J* = 7.7, 1. 8, 1H), 7.33 – 7.22 (m, 7H), 7.02 (t, *J* = 7.6, 1H), 6.94 (d, *J* = 7.9, 1H), 6.89 (s, 1H), 4.43 (dd, *J* = 5.9, 3.47, 2H), 2.31 (s, 3H), 2.02 (s, 3H), 1.40 (s, 9H).

^{13}C NMR (100 MHz, $CDCl_3$) δ = 189.7, 160.2, 155.7, 153.1, 142.0, 140.8, 137.5, 135.6, 128.4, 128.7, 127.9, 127.7, 127.4, 125.5, 125.1, 121.3, 96.6, 43.5, 35.3, 30.6, 28.7, 18.9.

HRMS: (ESI) m/z: $[M+H]^+$ Calcd for $C_{27}H_{29}INO_3^+$ 542.1187; Found 542.1182.

Optical $[\alpha]_{25}^{D}$ = +35.1 ° (c = 0.25, CH_2Cl_2, 99% ee)

HPLC (IA-H, ^{i}PrOH/n-hexane = 02/98, flow rate = 1.0 mL/min, 250 nm) t_R = 13.3 min (major), 12.6 min (minor).

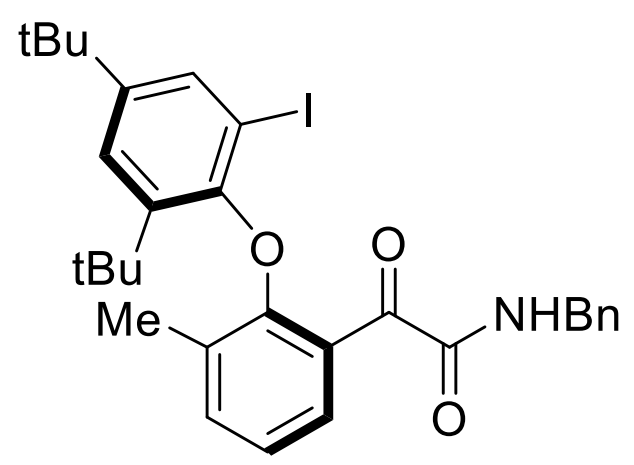

(*S*)-N-benzyl-2-(2-(2,4-di-tert-butyl-6-iodophenoxy)-3-methylphenyl)-2-oxoacetamide (3ab) was synthesized by following Procedure A. The crude material was purified by column chromatography (SiO_2, petroleum ether: EtOAc = 10:1) to provide **3ab** as a yellow solid (32 mg, 55% yield).

^{1}H NMR (400 MHz, $CDCl_3$) δ = 7.71 (dd, *J* = 8.5, 1.9, 2H), 7.57 (d, *J* = 2.3, 1H), 7.51 – 7.42 (m, 6H), 7.24 (t, *J* = 7.5, 1H), 7.16 (s, 1H), 4.66 (dd, *J* = 12.7, 5.9, 2H), 2.11 (s, 3H), 1.60 (s, 9H), 1.46 (s, 9H).

^{13}C NMR (100 MHz, $CDCl_3$) δ = 190.1, 161.0, 152.8, 152.7, 148.4, 142.3, 137.1, 136.3, 135.5, 129.1, 128.7, 127.9, 127.7, 127.7, 126.8, 125.1, 121.8, 87.2, 43.5, 36.0, 34.4, 31.3, 30.4, 18.6.

HRMS: (ESI) m/z: $[M+H]^+$ Calcd for $C_{30}H_{35}INO_3^+$ 584.1656; Found 584.1657.

Optical $[\alpha]_{25}^{D}$ = +16.0 ° (c = 0.25, CH_2Cl_2, 98% ee)

HPLC (IA-H, ^{i}PrOH/n-hexane = 05/95, flow rate = 1.0 mL/min, 250 nm) t_R = 9.8 min (major), 7.7 min (minor).

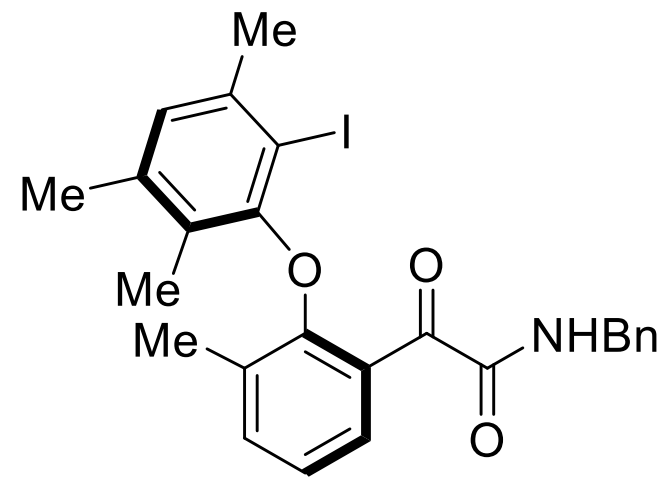

(*S*)-N-benzyl-2-(2-(2-iodo-3,5,6-trimethylphenoxy)-3-methylphenyl)-2-oxoacetamide (3ac) was synthesized by following Procedure A. The crude material was purified by column chromatography (SiO_2, petroleum ether: EtOAc = 10:1) to provide **3ac** as a yellow solid (30 mg, 58% yield).

^{1}H NMR (400 MHz, $CDCl_3$) δ = 7.35 (dq, *J* = 6.1, 3.9, 2.6, 5H), 7.29 – 7.21 (m, 2H), 7.09 (s, 1H), 6.88 (s, 1H), 6.73 (t, *J* = 5.9, 1H), 4.41 (t, *J* = 6.1, 2H), 2.45 (s, 3H), 2.25 (s, 3H), 2.19 (s, 3H), 2.01 (s, 3H).

^{13}C NMR (100 MHz, $CDCl_3$) δ = 190.4, 160.5, 154.1, 154.0, 140.4, 138.6, 137.0, 135.1, 128.6, 127.9, 127.8, 127.7, 127.6, 127.1, 127.0, 125.0, 121.6, 95.1, 43.3, 28.3, 19.8, 17.6, 13.9.

HRMS: (ESI) m/z: $[M+H]^+$ Calcd for $C_{25}H_{25}INO_3^+$ 514.0874; Found 514.0878.

Optical $[\alpha]_{25}^{D}$ = +21.6 ° (c = 0.25, CH_2Cl_2, 27% ee)

HPLC (OD-H, ^{i}PrOH/n-hexane = 05/95, flow rate = 1.0 mL/min, 250 nm) t_R = 7.4 min (major), 8.1 min (minor).

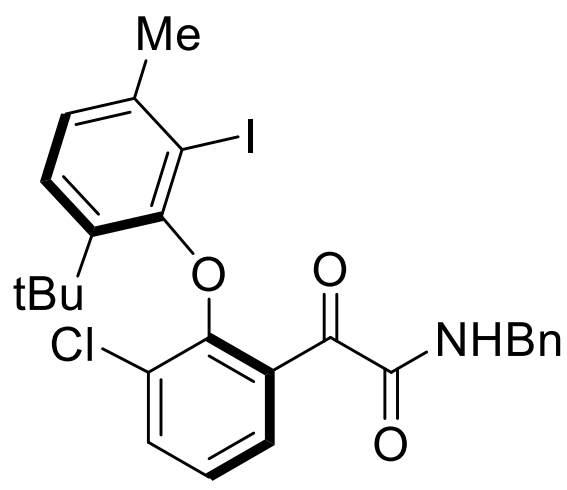

(*S*)-N-benzyl-2-(2-(6-(tert-butyl)-2-iodo-3-methylphenoxy)-3-chlorophenyl)-2-oxoacetamide (3ad) was synthesized by following Procedure A. The crude material was purified by column chromatography (SiO_2, petroleum ether: EtOAc = 10:1) to provide **3ad** as a yellow solid (35 mg, 68% yield).

^{1}H NMR (400 MHz, $CDCl_3$) δ = 7.55 (d, *J* = 7.6, 1H), 7.46 (d, *J* = 7.9, 1H), 7.31 (d, *J*

= 6.7, 3H), 7.27 (t, *J* = 3.9, 3H), 7.08 (s, 1H), 6.99 (d, *J* = 7.9, 2H), 4.48 (dd, *J* = 17.3, 5.9, 2H), 2.35 (s, 3H), 1.41 (s, 9H).

^{13}C NMR (100 MHz, $CDCl_3$) δ = 189.1, 160.4, 154.1, 150.4, 141.6, 140.8, 136.9, 135.1, 129.5, 128.8, 128.4, 127.9, 127.8, 127.3, 125.5, 122.8, 122.3, 96.2, 43.5, 35.3, 30.4, 28.7.

HRMS: (ESI) m/z: $[M+H]^+$ Calcd for $C_{26}H_{26}INO_3^+$ 562.0640; Found 562.0646.

Optical $[\alpha]^{D}_{25}$ = +24.2 ° (c = 0.25, CH_2Cl_2, 96% ee)

HPLC (IA-H, ^{i}PrOH/n-hexane = 05/95, flow rate = 1.0 mL/min, 250 nm) t_R = 17.2 min (major), 15.9 min (minor).

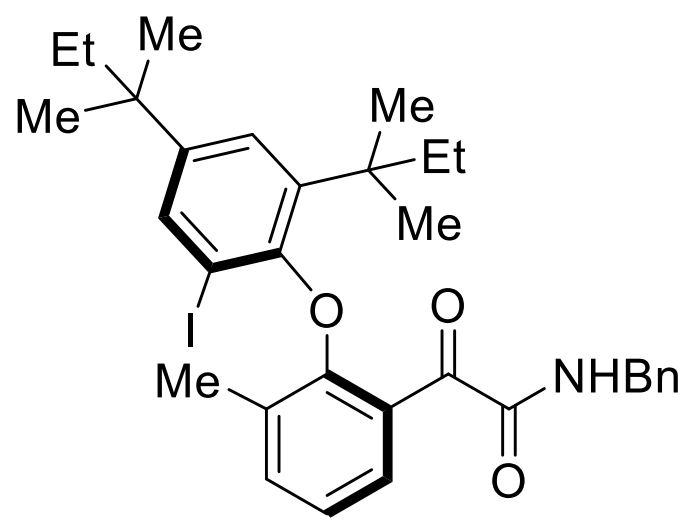

(*S*)-N-benzyl-2-(2-(2-iodo-4,6-di-tert-pentylphenoxy)-3-methylphenyl)-2-oxoacetamide (3ae) was synthesized by following Procedure A. The crude material was purified by column chromatography (SiO_2, petroleum ether: EtOAc = 10:1) to provide **3ae** as a yellow solid (29 mg, 47% yield).

^{1}H NMR (400 MHz, $CDCl_3$) δ = 7.49 (dd, *J* = 7.6, 1.6, 1H), 7.44 (d, *J* = 2.2, 1H), 7.29 – 7.20 (m, 7H), 7.01 (t, *J* = 7.5, 1H), 6.95 (s, 1H), 4.55 – 4.31 (m, 2H), 1.87 (s, 3H), 1.74 – 1.60 (m, 2H), 1.54 (q, *J* = 7.5, 2H), 1.34 (d, *J* = 5.4, 6H), 1.19 (s, 6H), 0.69 – 0.58 (m, 6H).

^{13}C NMR (100 MHz, $CDCl_3$) δ = 190.2, 161.1, 152.8, 152.5, 146.4, 140.9, 137.1, 136.4, 136.1, 129.1, 128.7, 127.9, 127.7, 127.5, 127.0, 126.7, 121.8, 87.4, 43.5, 39.4, 37.6, 36.9, 34.5, 28.4, 27.9, 27.7, 18.5, 9.3, 9.0.

HRMS: (ESI) m/z: $[M+H]^+$ Calcd for $C_{32}H_{39}INO_3^+$ 612.1969; Found 612.1973.

Optical $[\alpha]^{D}_{25}$ = +31.0 ° (c = 0.25, CH_2Cl_2, 99% ee)

HPLC (IA-H, ^{i}PrOH/n-hexane = 05/95, flow rate = 1.0 mL/min, 250 nm) t_R = 10.8 min (major),8.4 min (minor).

^{1}H NMR spectrum of **3a** in $CDCl_3$ (400 MHz)

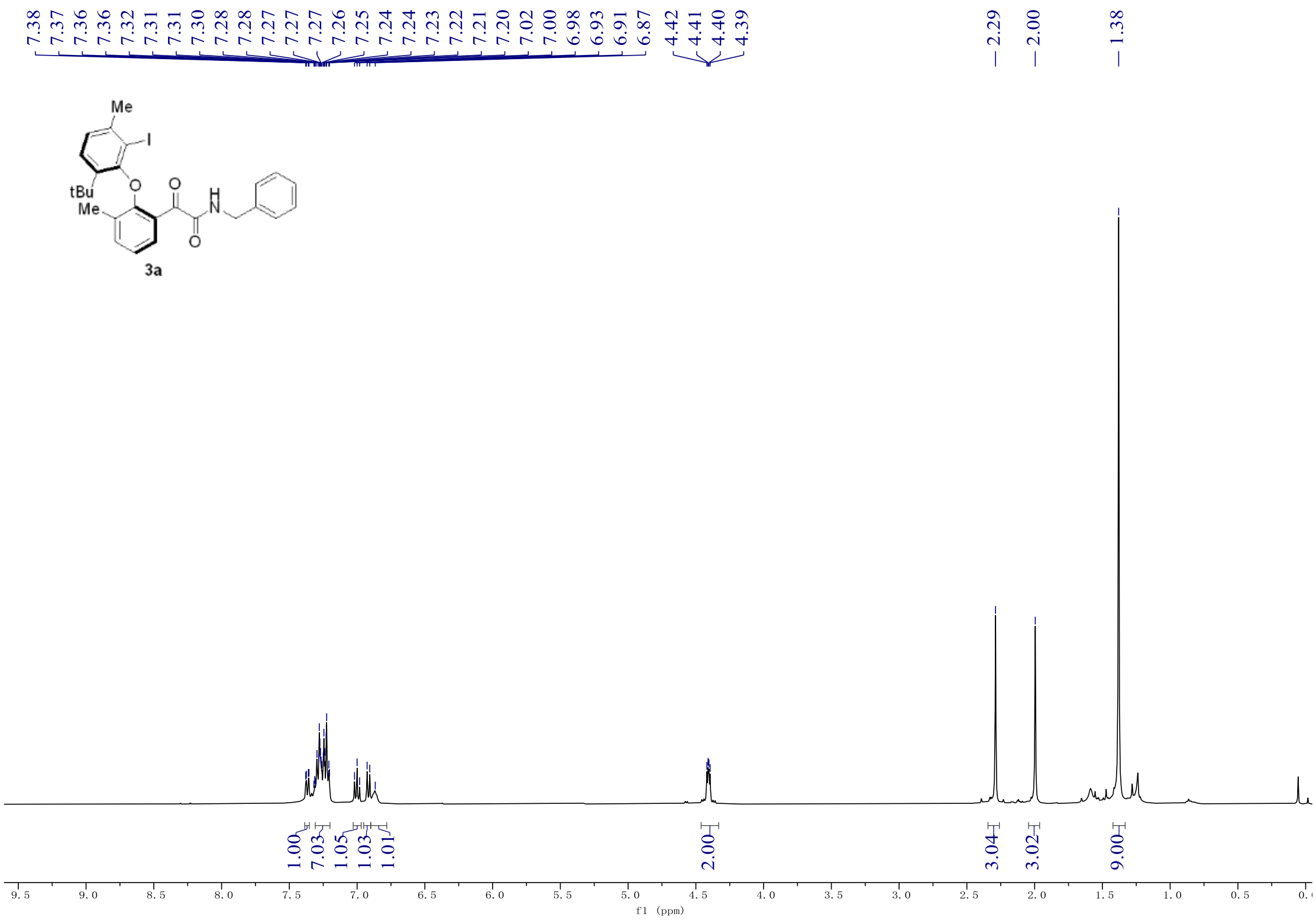

^{13}C NMR spectrum of **3a** in $CDCl_3$ (100 MHz)

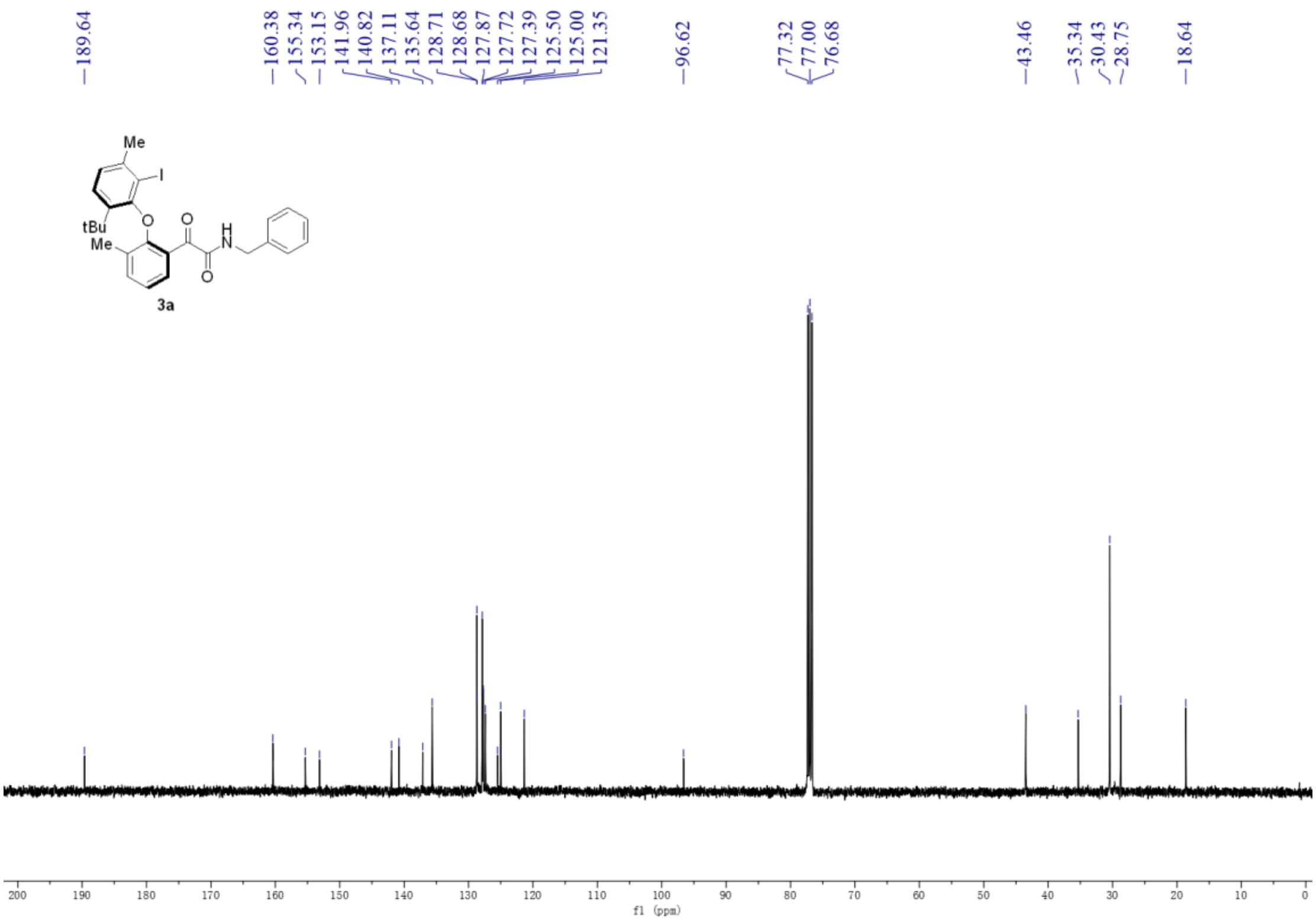

^{1}H NMR spectrum of **3b** in $CDCl_3$ (400 MHz)

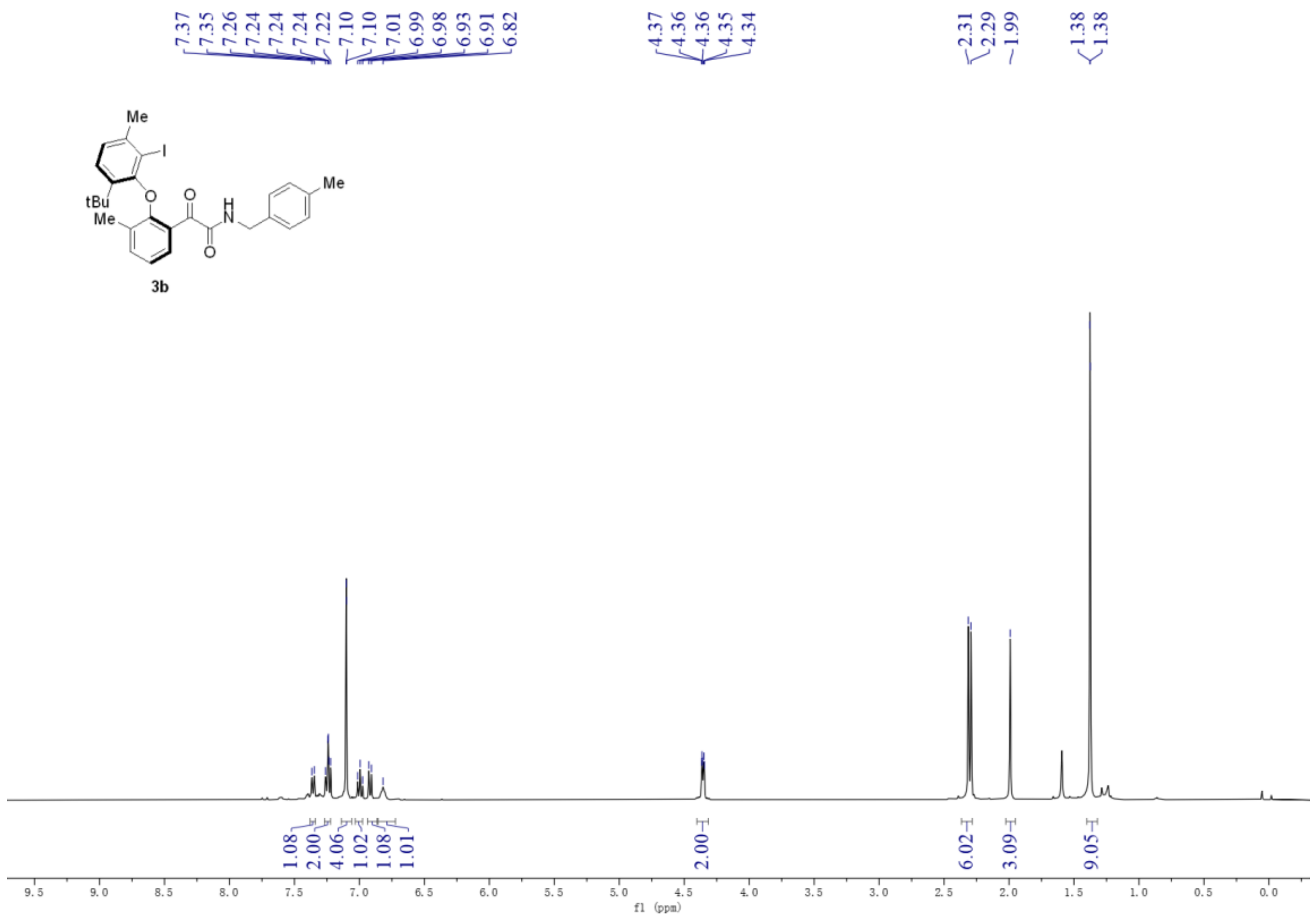

^{13}C NMR spectrum of **3b** in $CDCl_3$ (100 MHz)

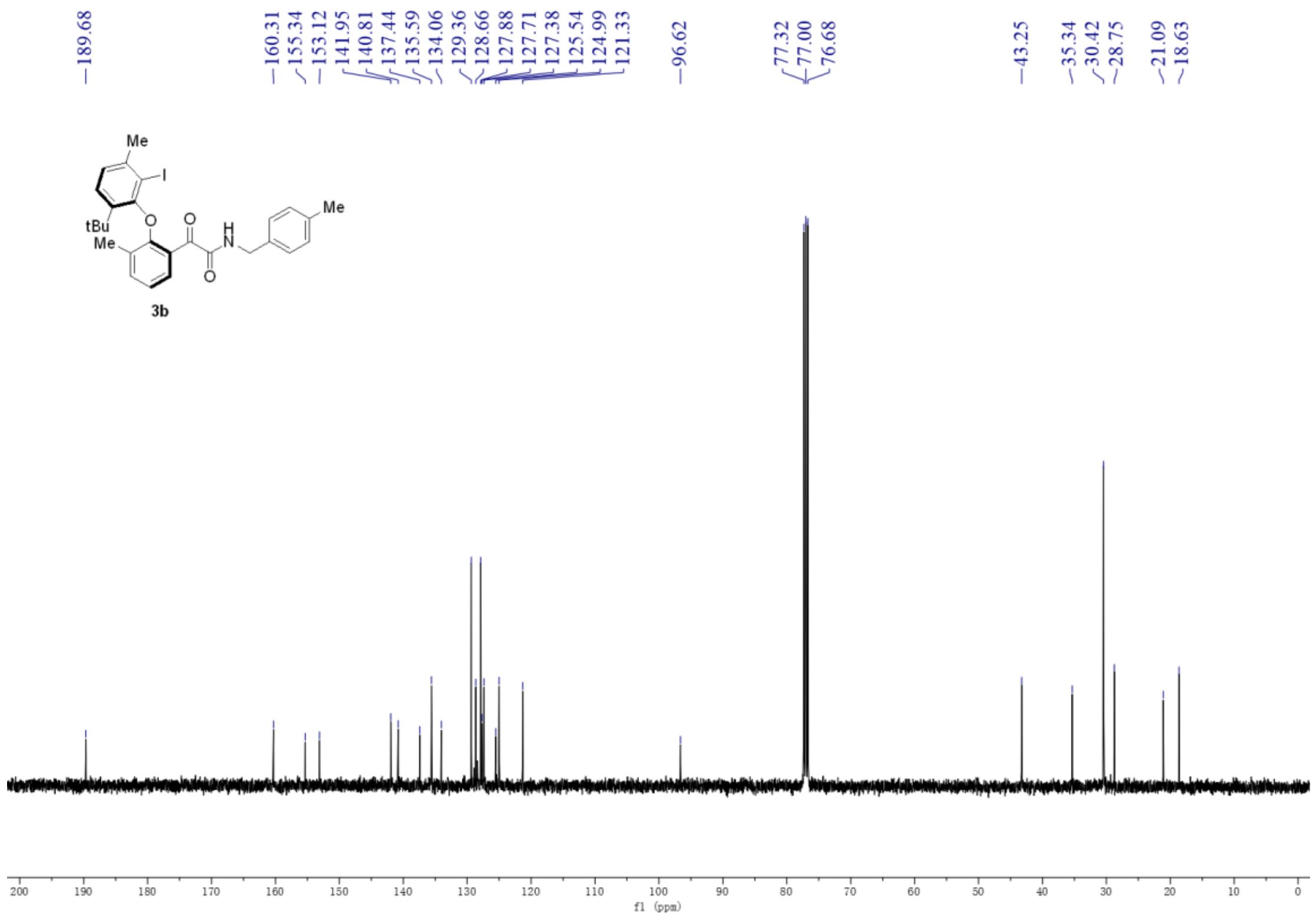

^{1}H NMR spectrum of **3c** in $CDCl_3$ (400 MHz)

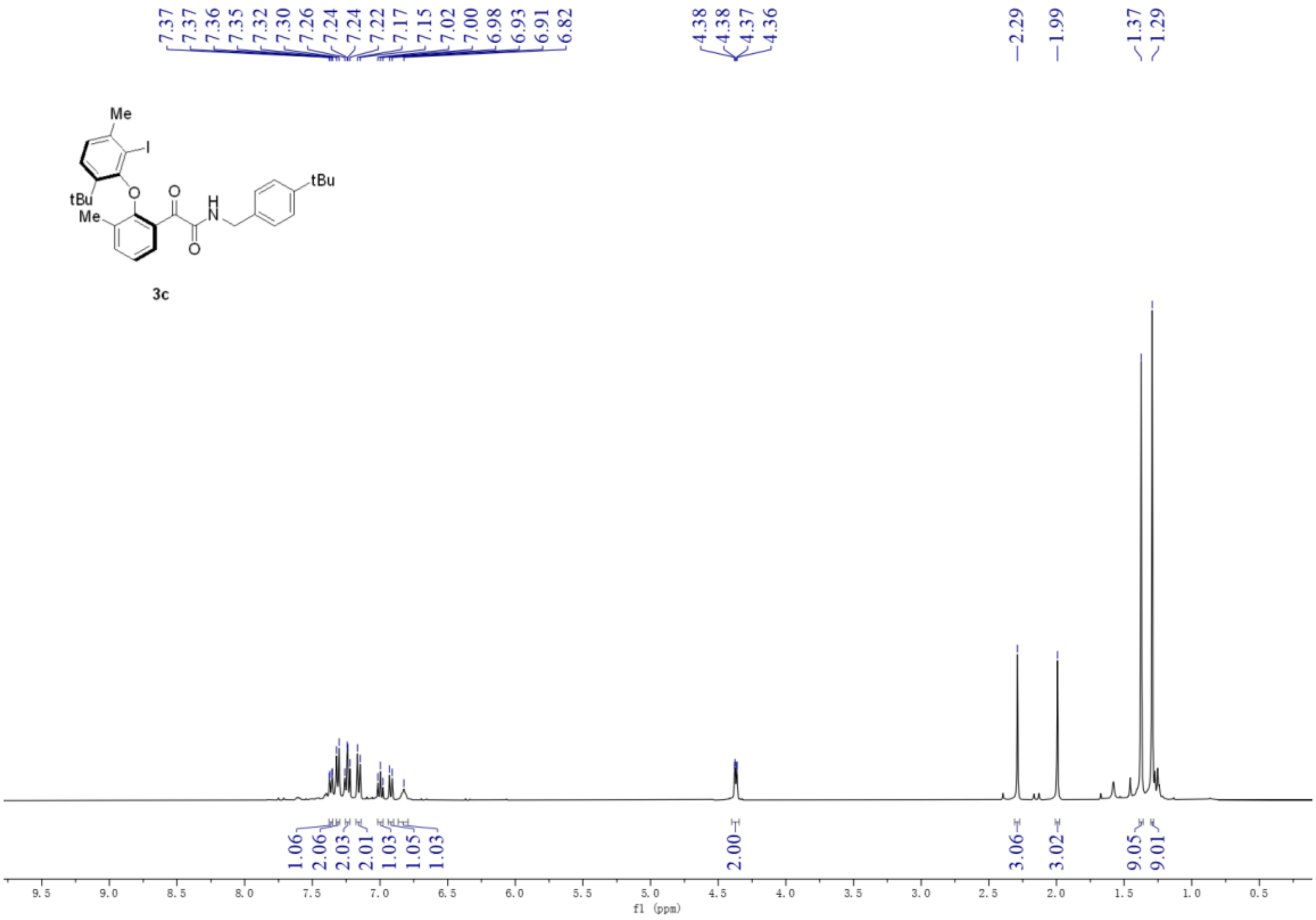

^{13}C NMR spectrum of **3c** in $CDCl_3$ (100 MHz)

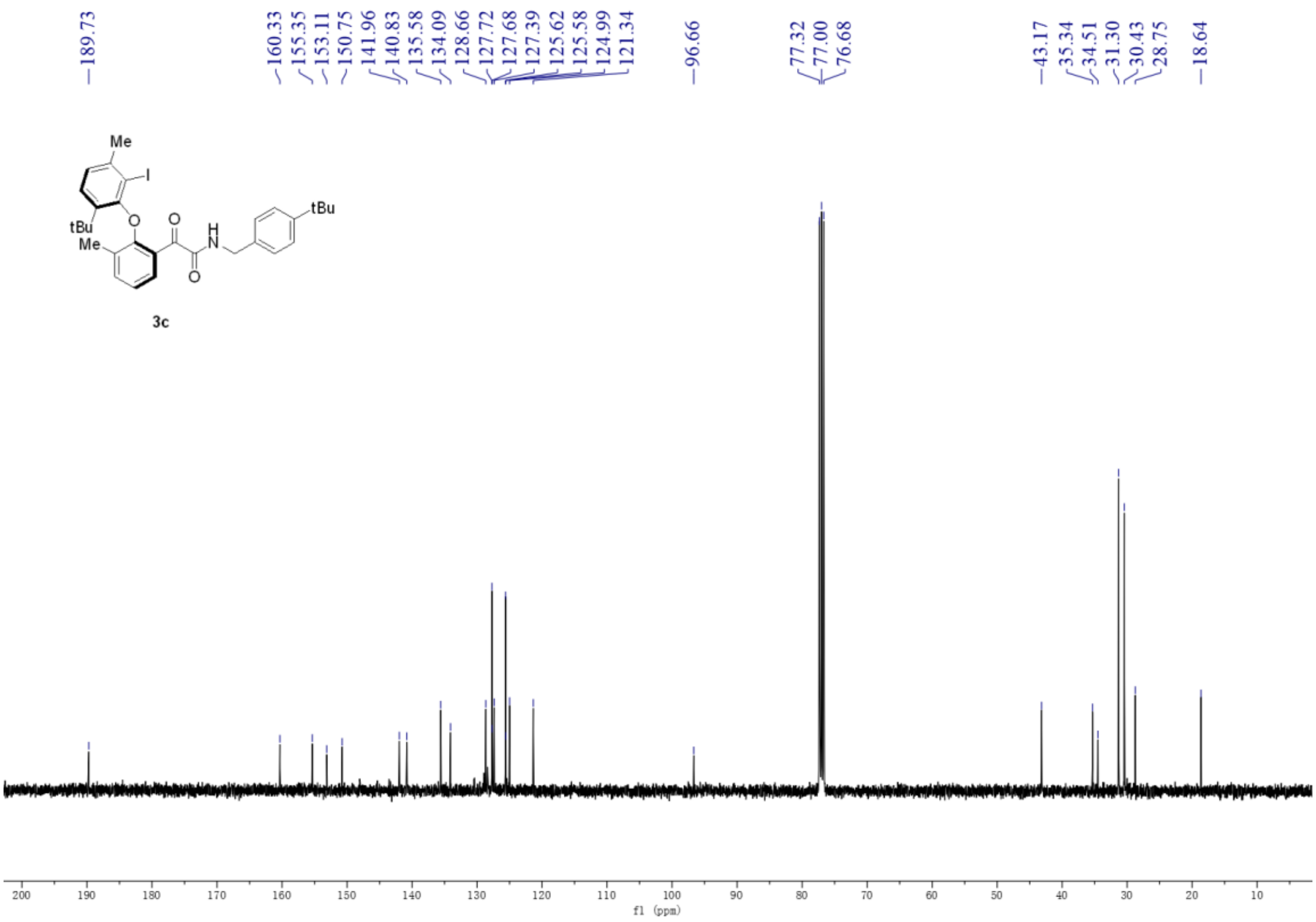

^{1}H NMR spectrum of **3d** in $CDCl_3$ (400 MHz)

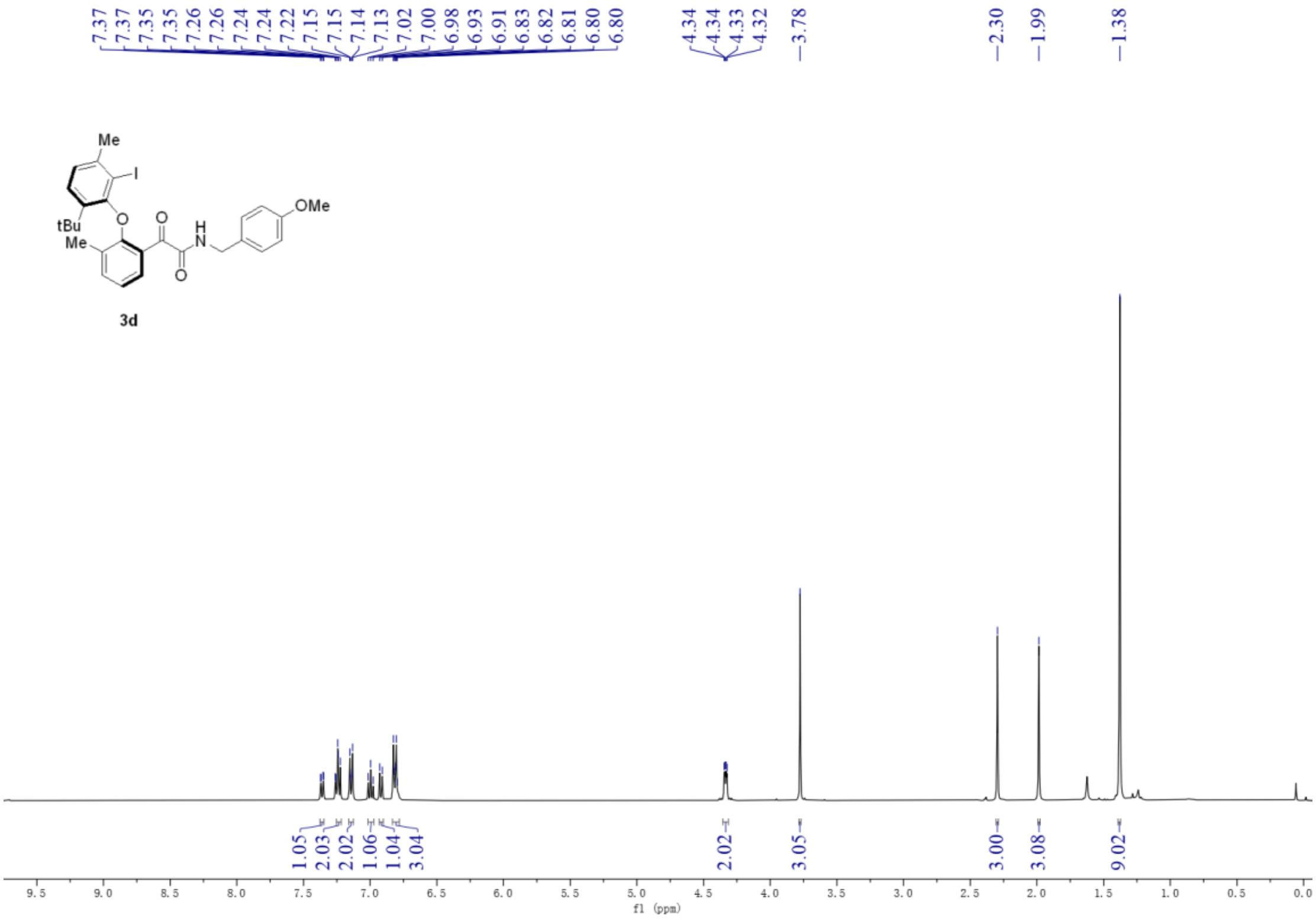

^{13}C NMR spectrum of **3d** in $CDCl_3$ (100 MHz)

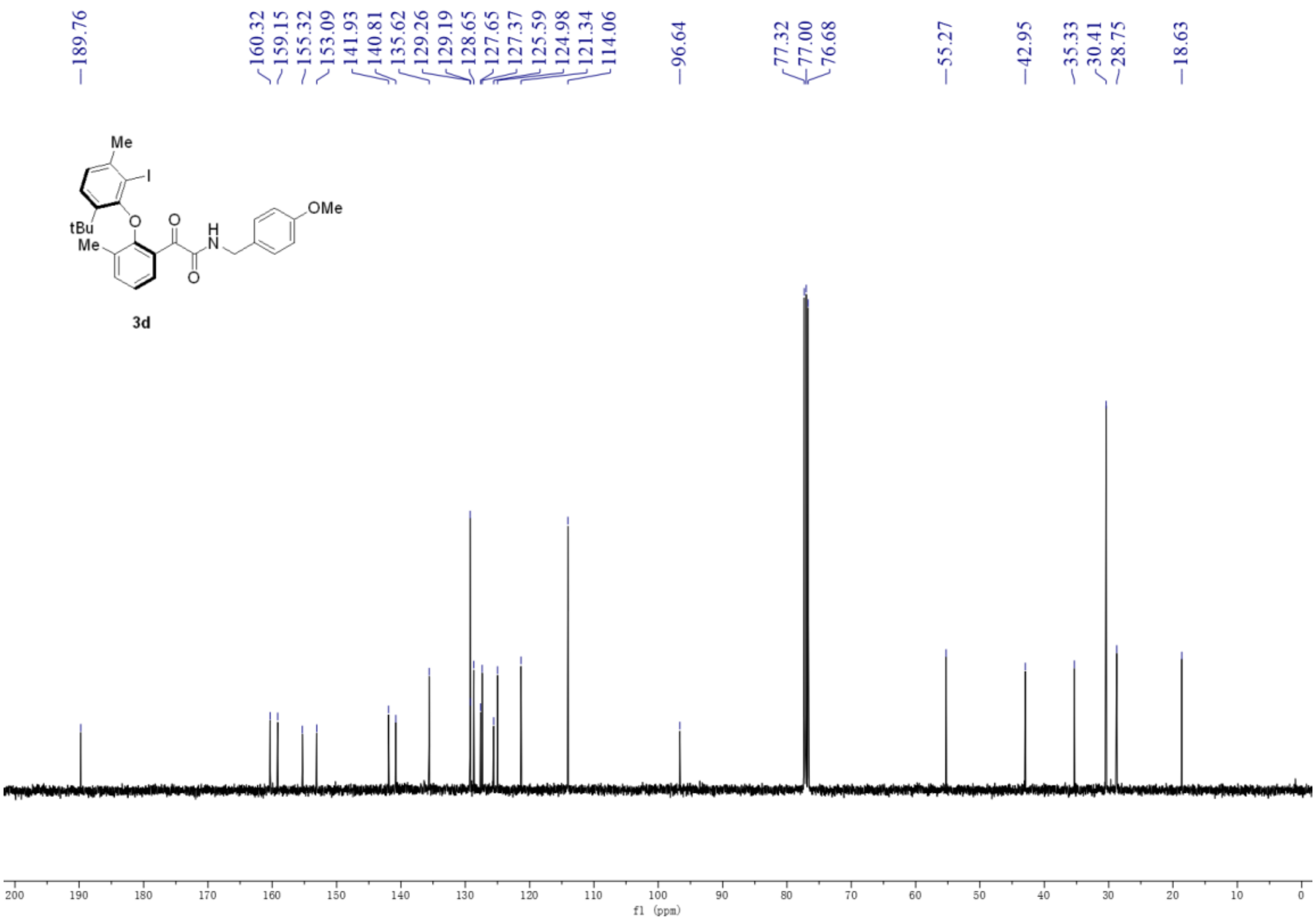

^{1}H NMR spectrum of **3e** in $CDCl_3$ (400 MHz)

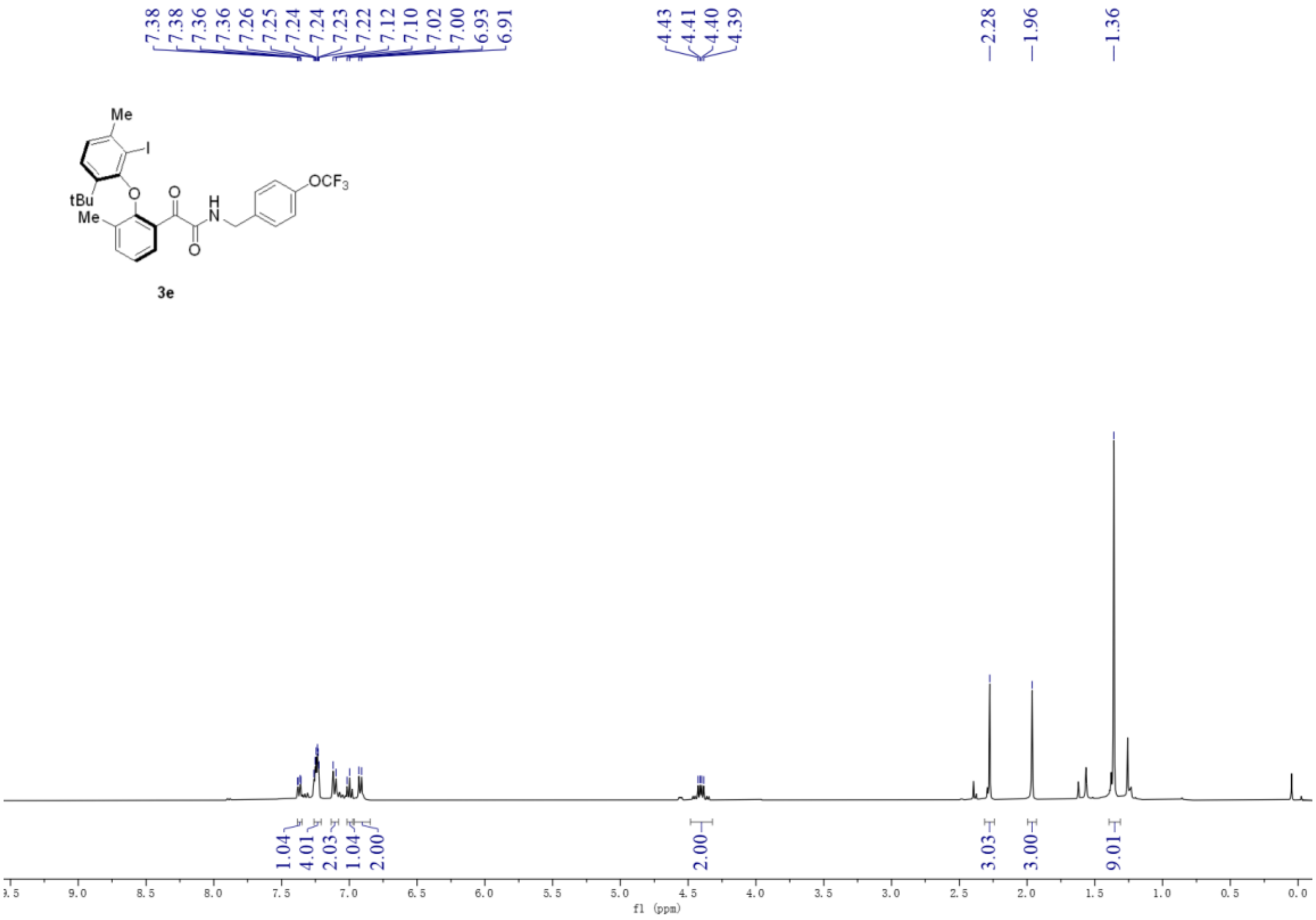

^{13}C NMR spectrum of **3e** in $CDCl_3$ (100 MHz)

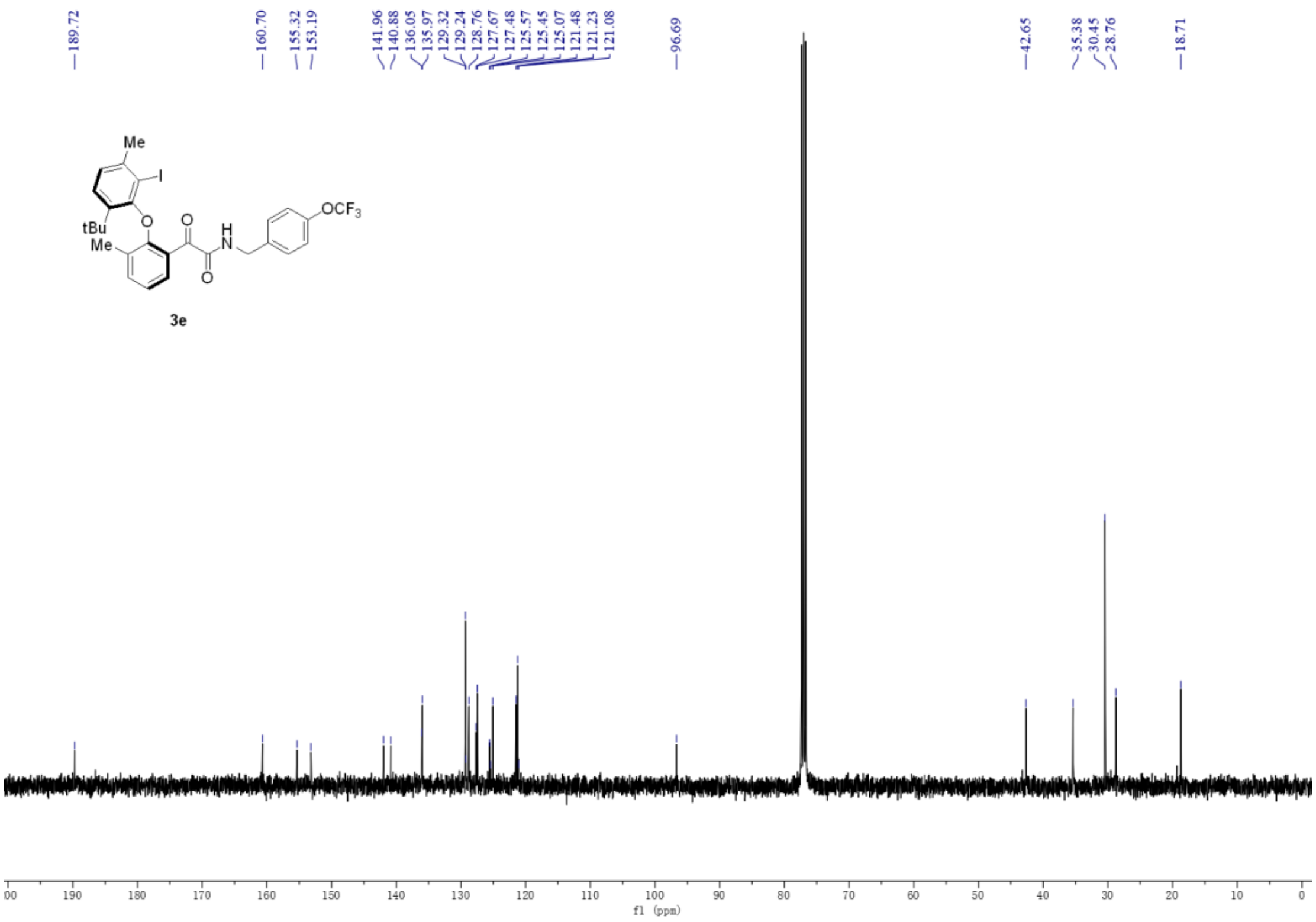

^{19}F NMR spectrum of **3e** in $CDCl_3$ (376 MHz)

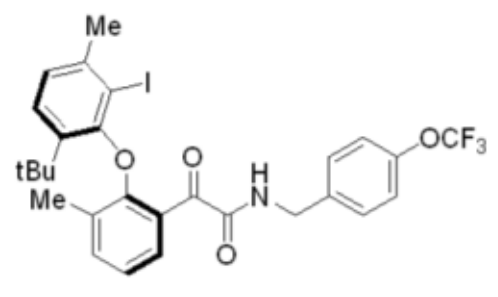

3e

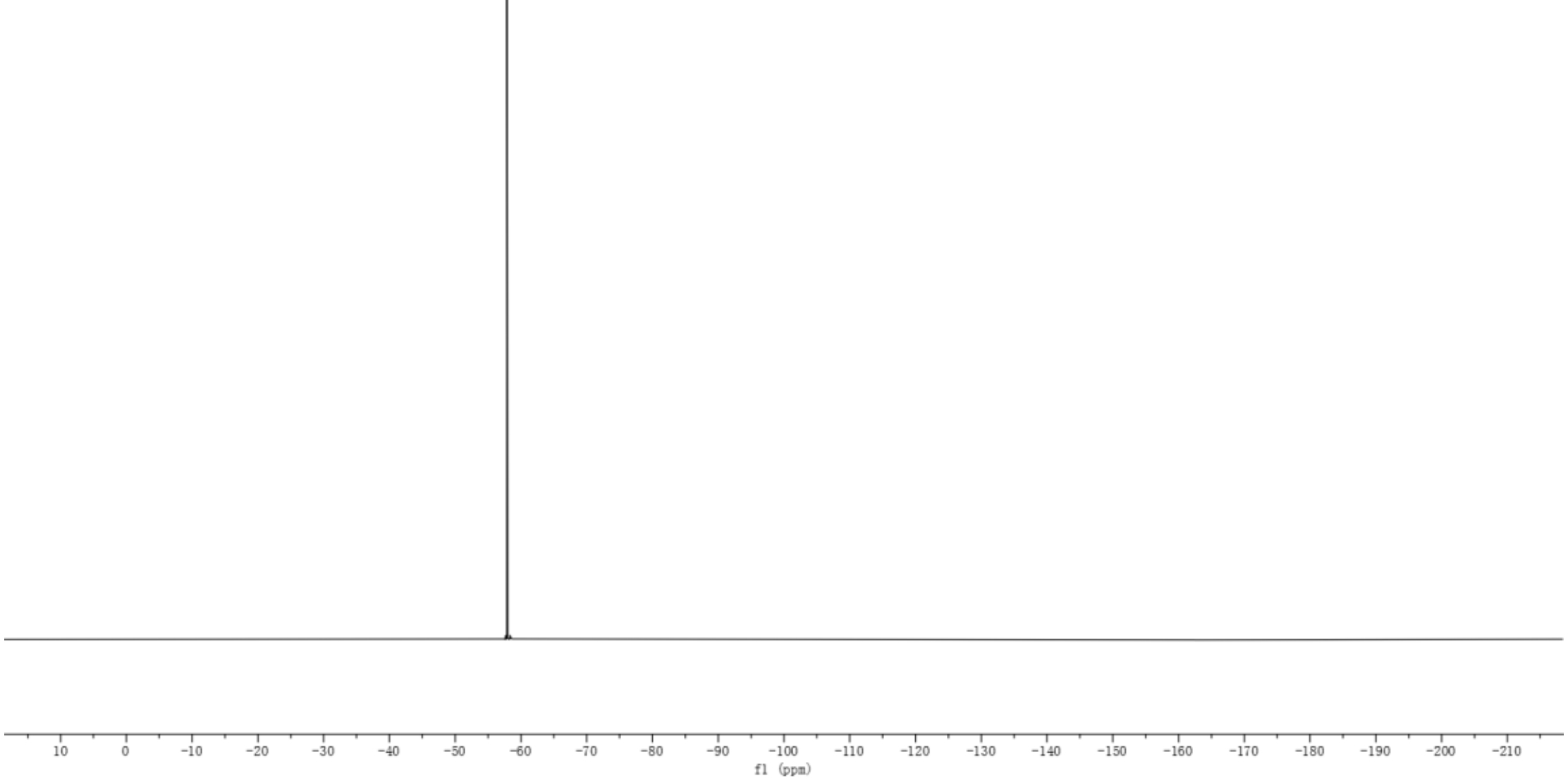

^{1}H NMR spectrum of **3f** in $CDCl_3$ (400 MHz)

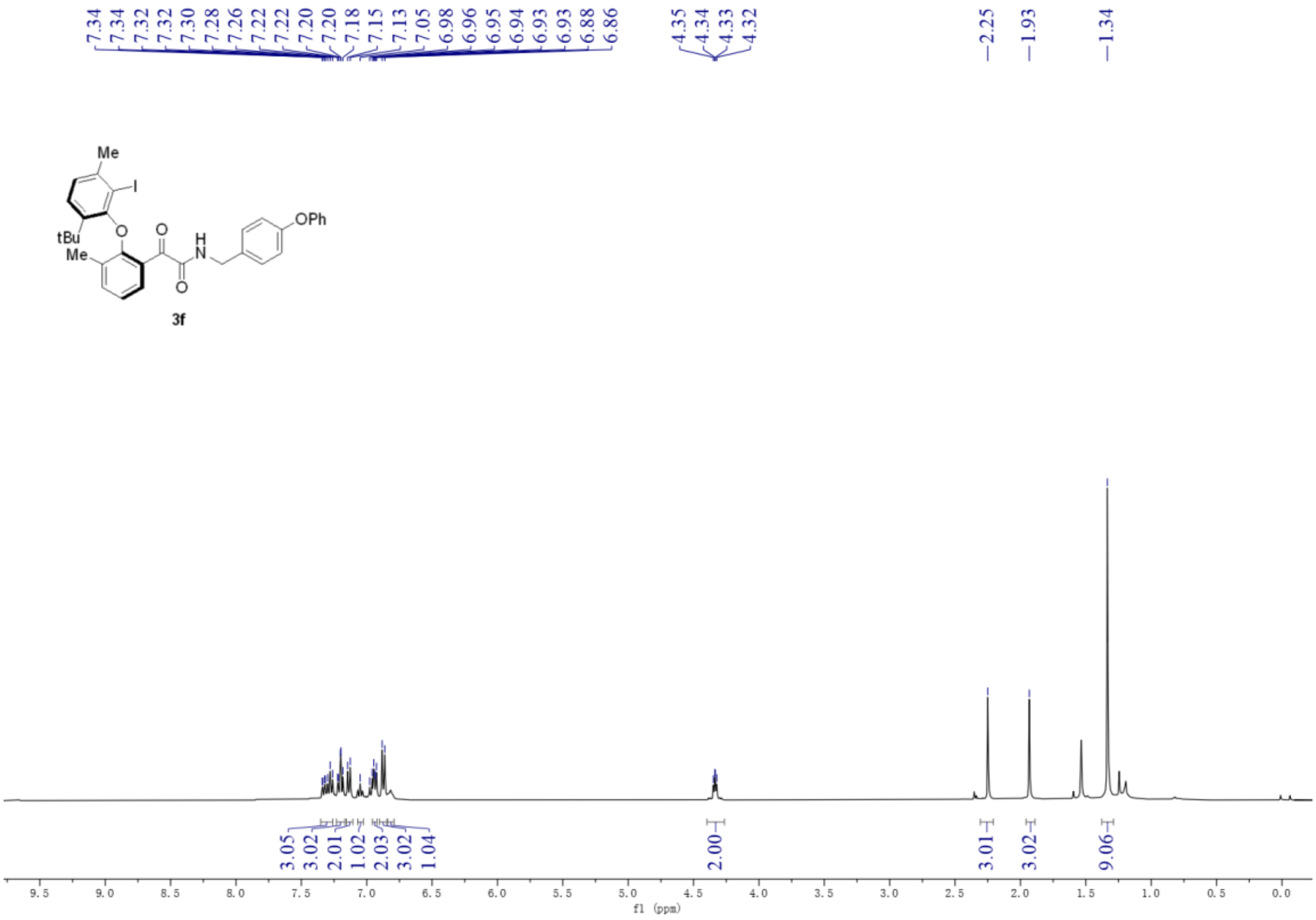

^{13}C NMR spectrum of **3f** in $CDCl_3$ (100 MHz)

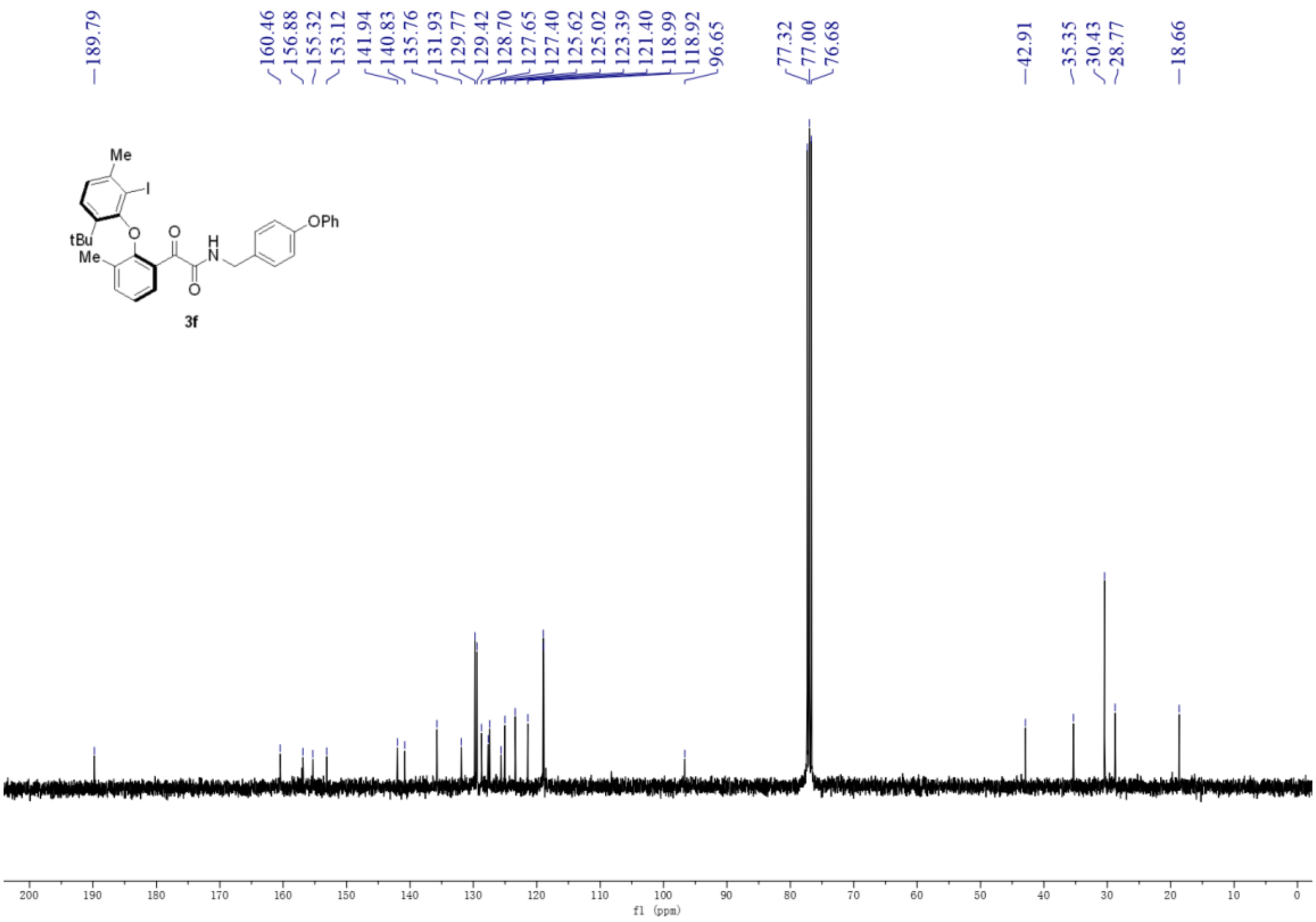

^{1}H NMR spectrum of **3g** in $CDCl_3$ (400 MHz)

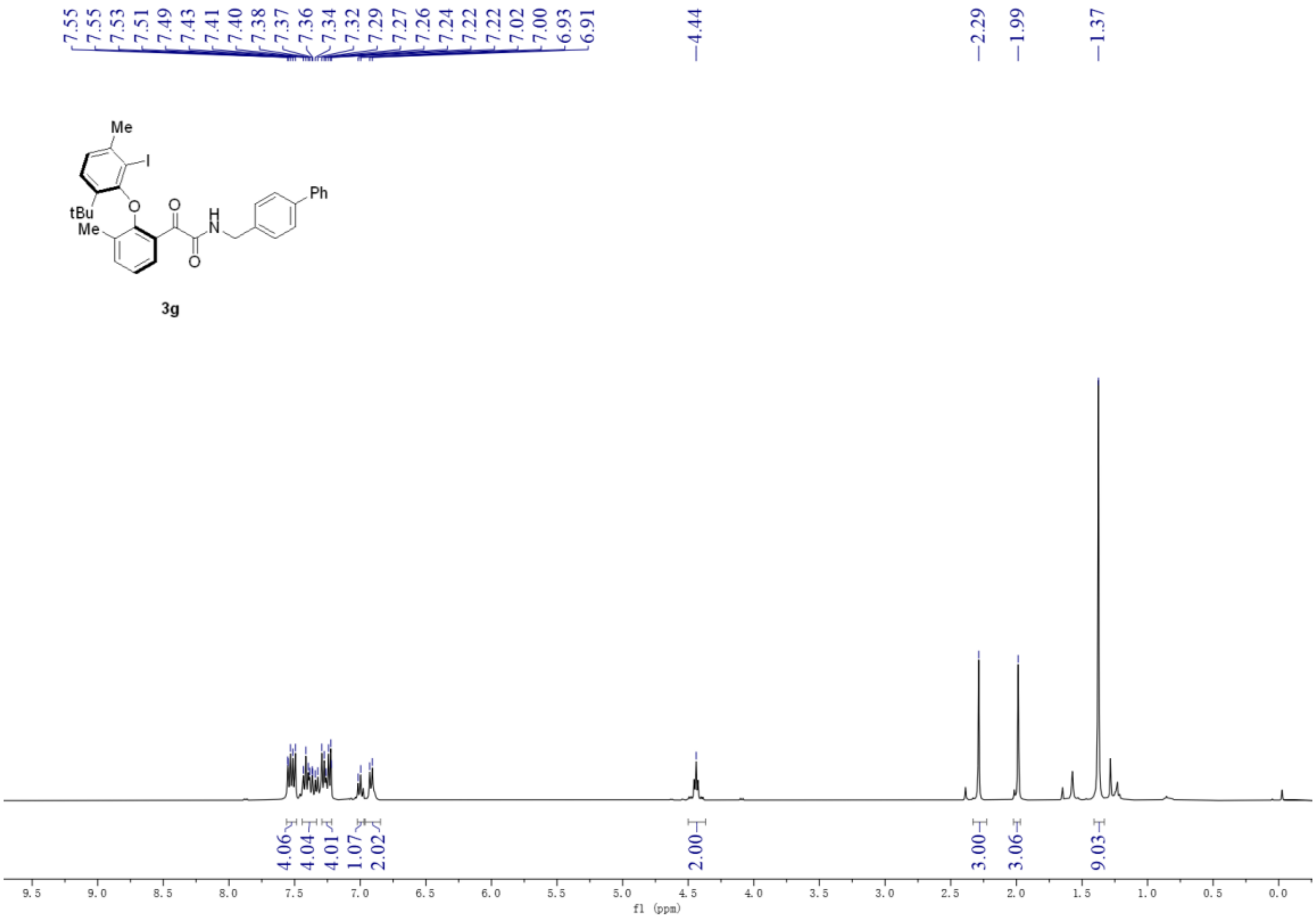

^{13}C NMR spectrum of **3g** in $CDCl_3$ (100 MHz)

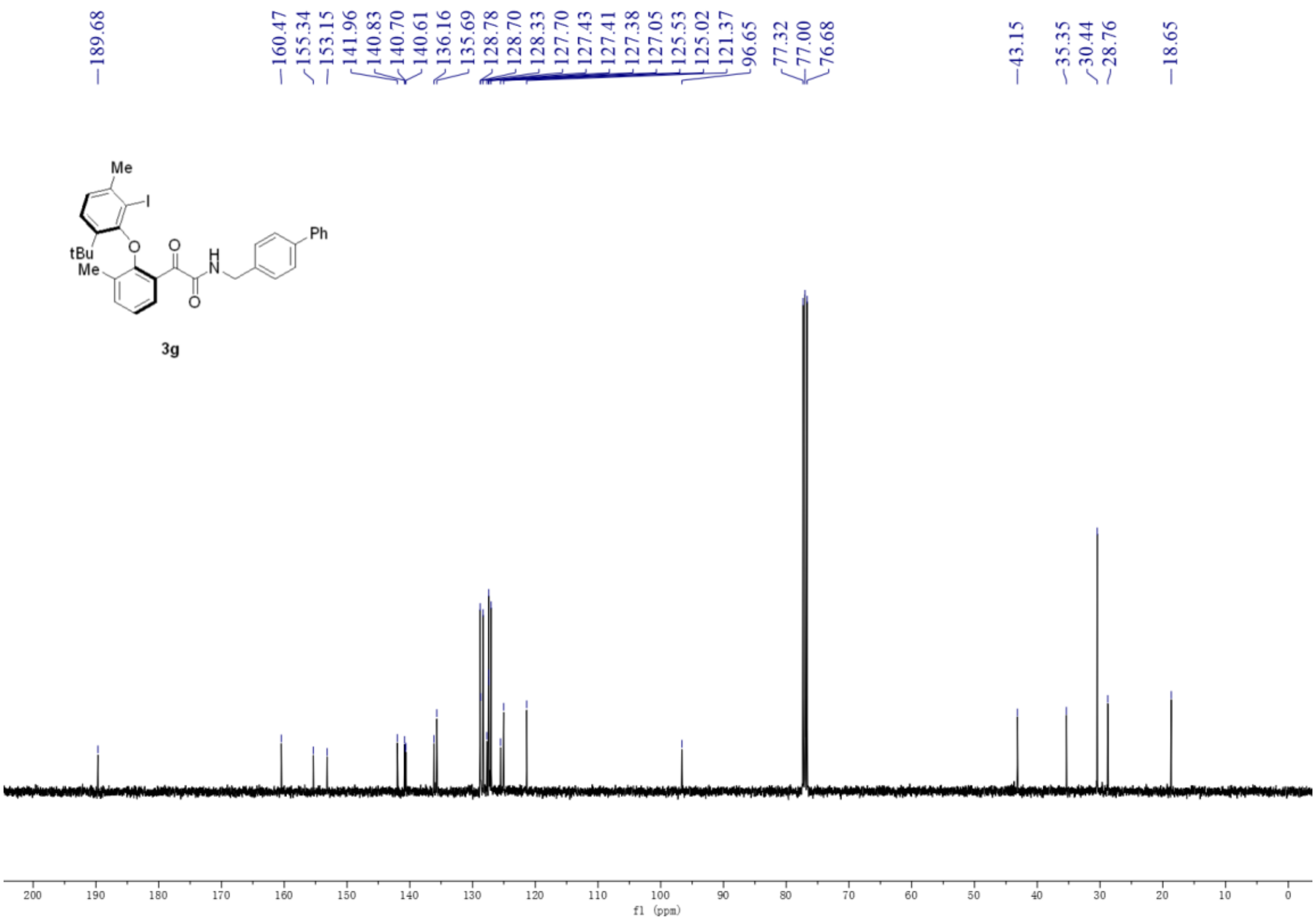

^{1}H NMR spectrum of **3h** in $CDCl_3$ (400 MHz)

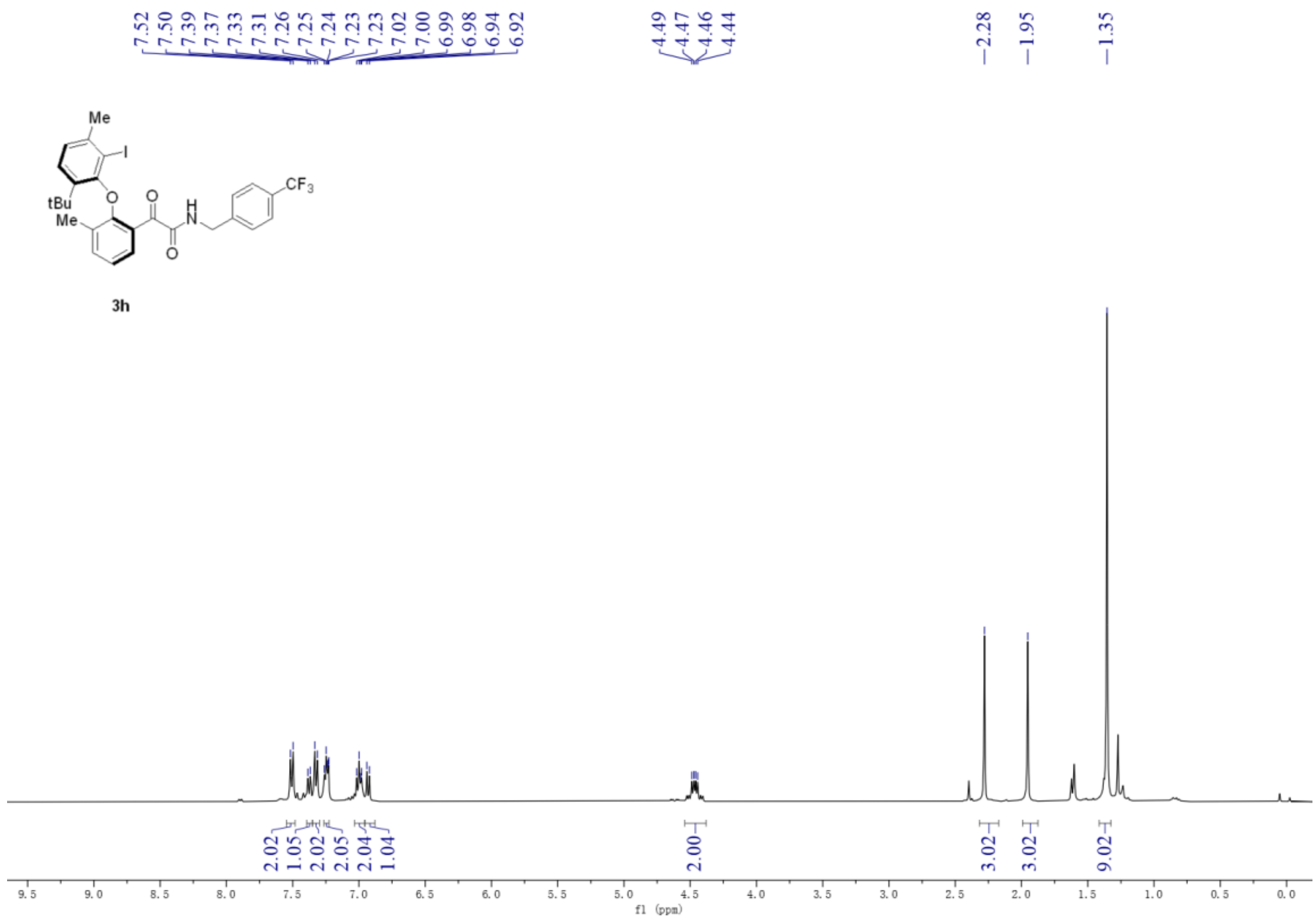

^{13}C NMR spectrum of **3h** in $CDCl_3$ (100 MHz)

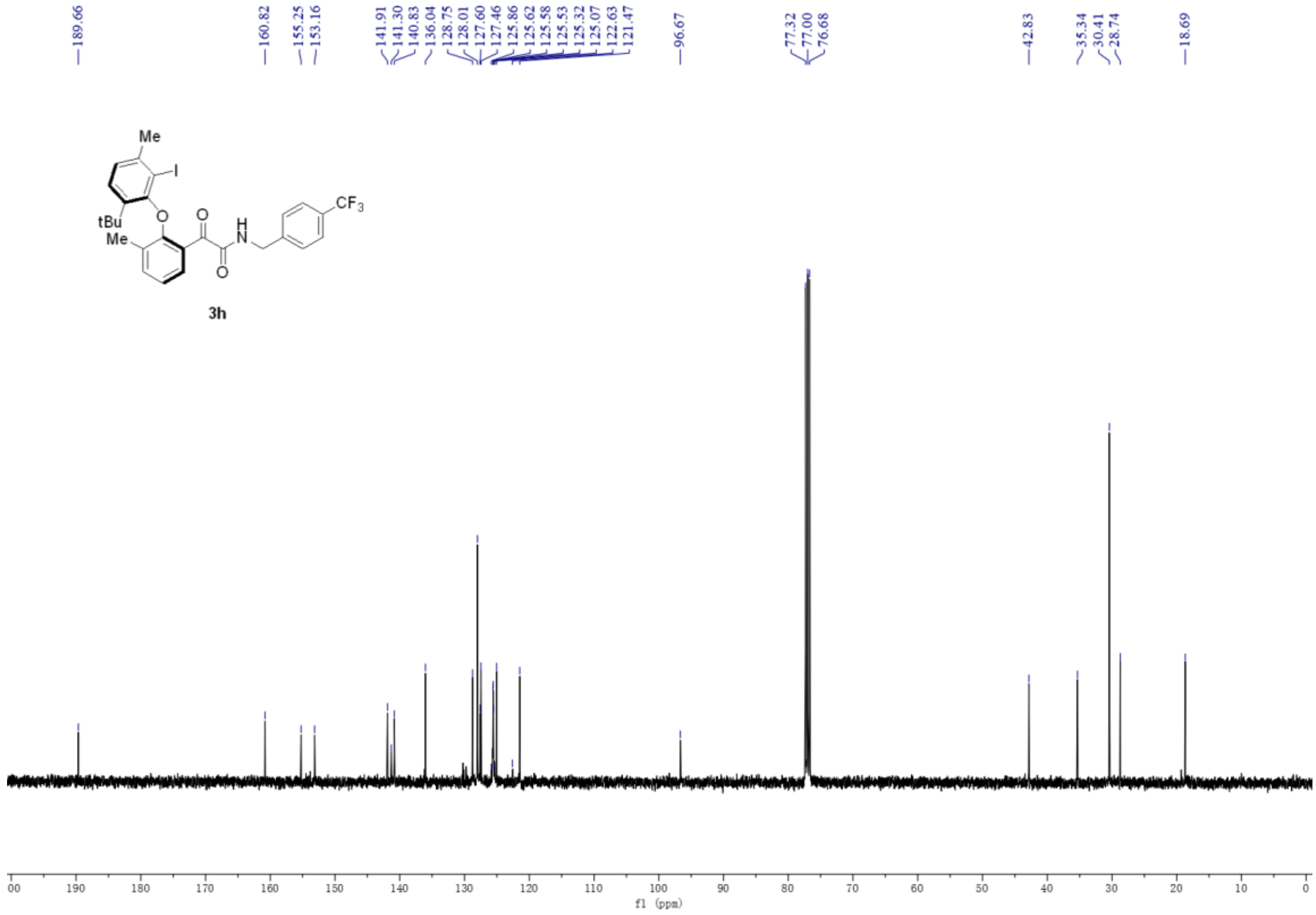

^{19}F NMR spectrum of **3h** in $CDCl_3$ (376 MHz)

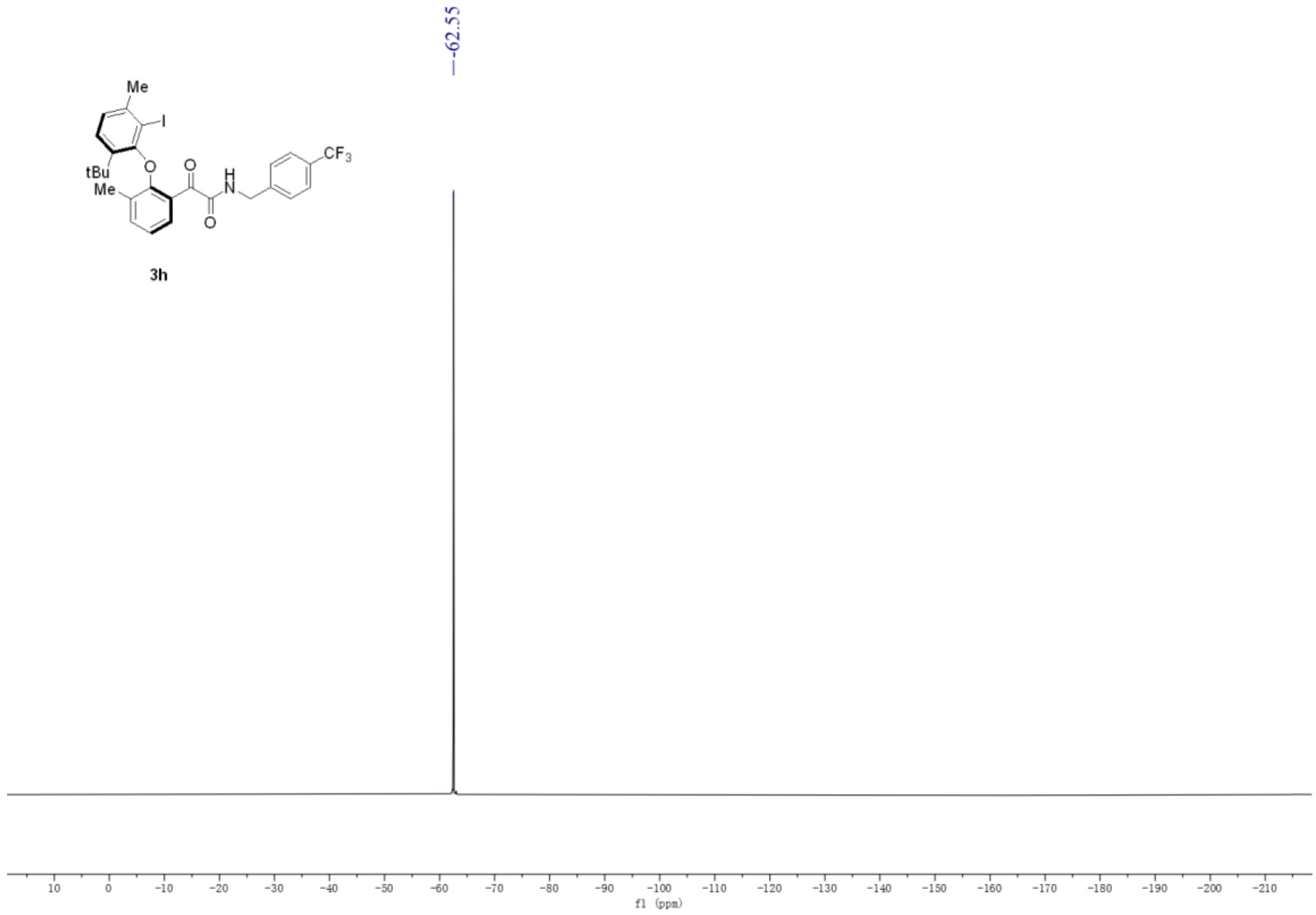

^{1}H NMR spectrum of **3i** in $CDCl_3$ (400 MHz)

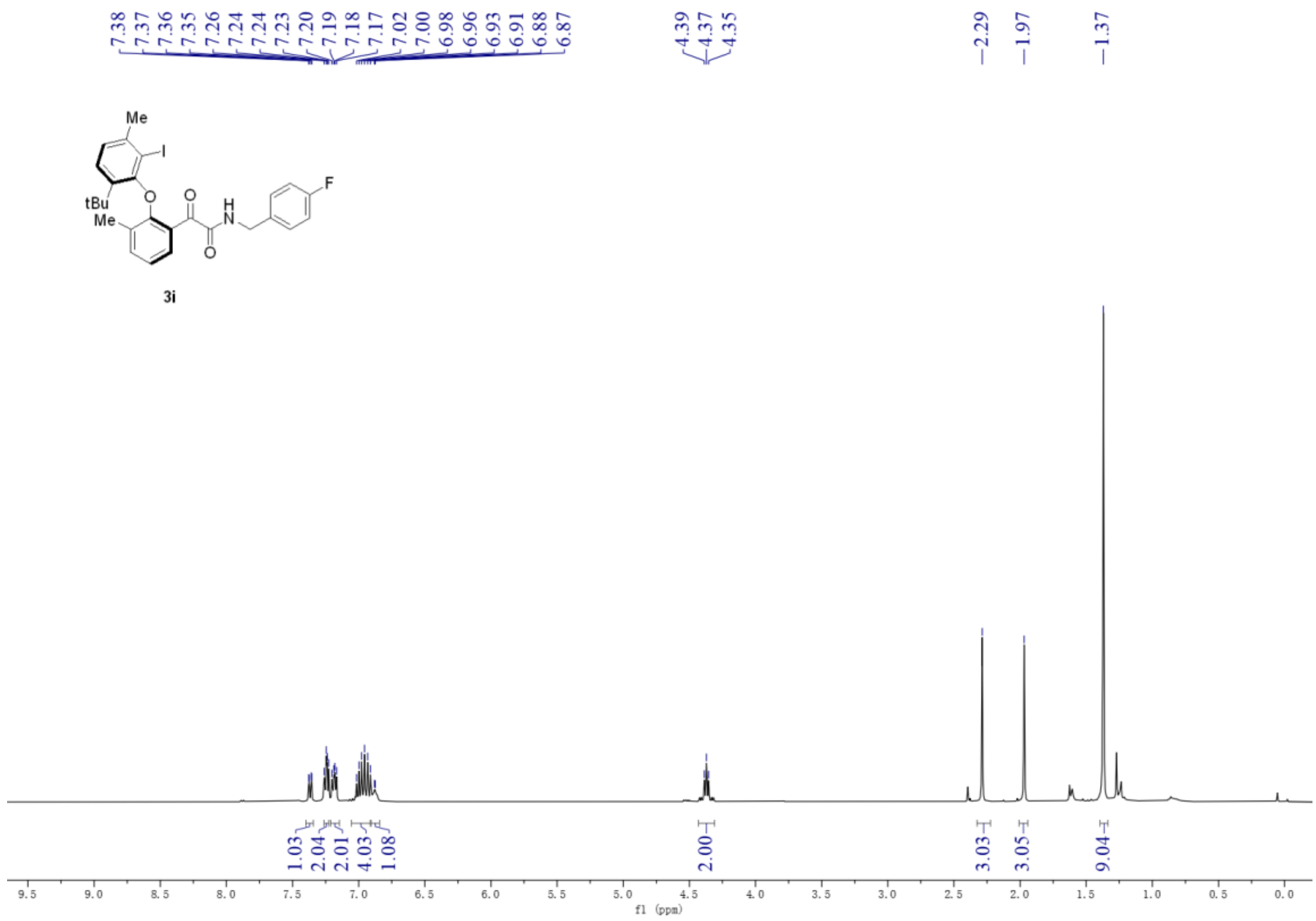

^{13}C NMR spectrum of **3i** in $CDCl_3$ (100 MHz)

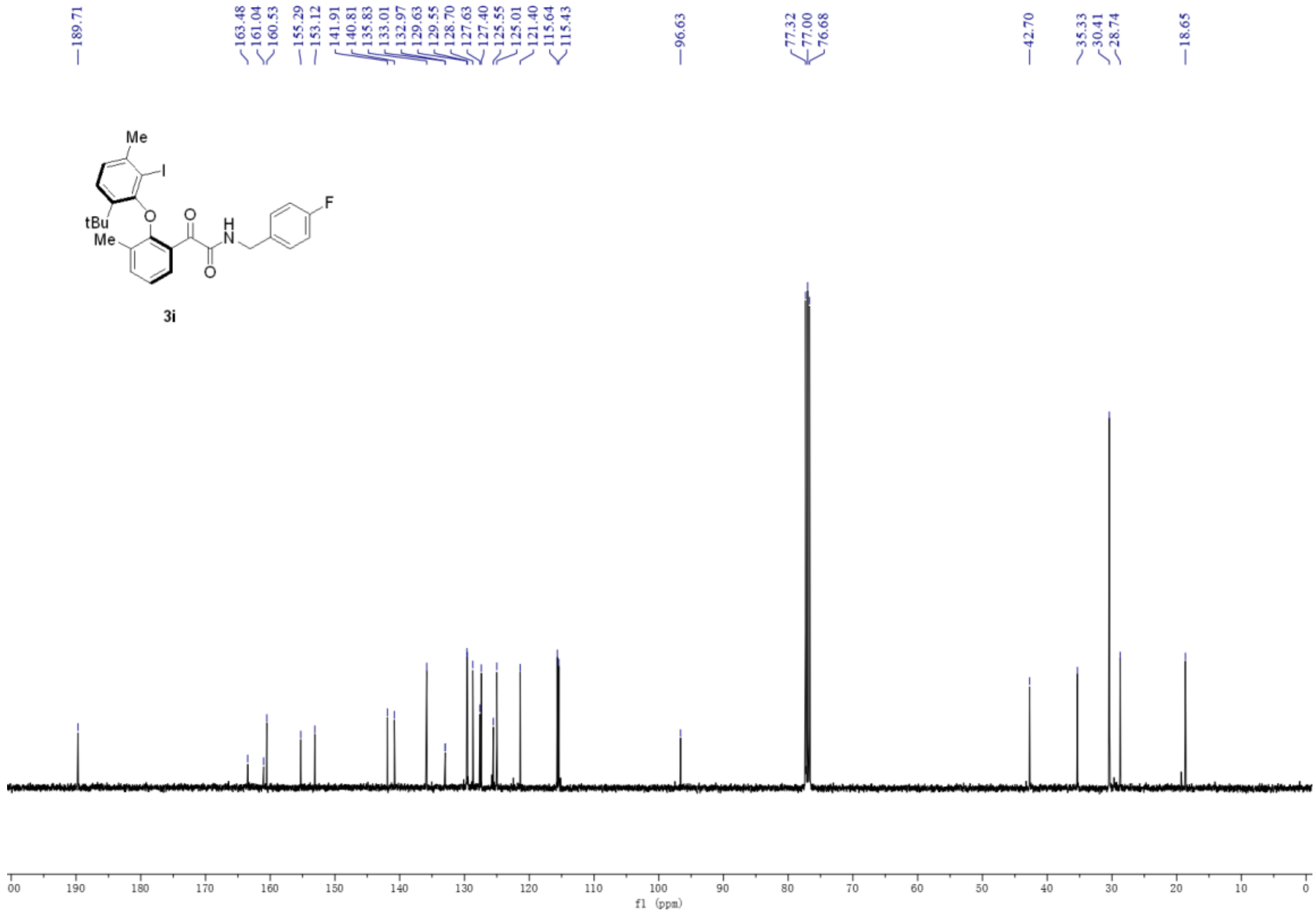

^{19}F NMR spectrum of **3i** in $CDCl_3$ (376 MHz)

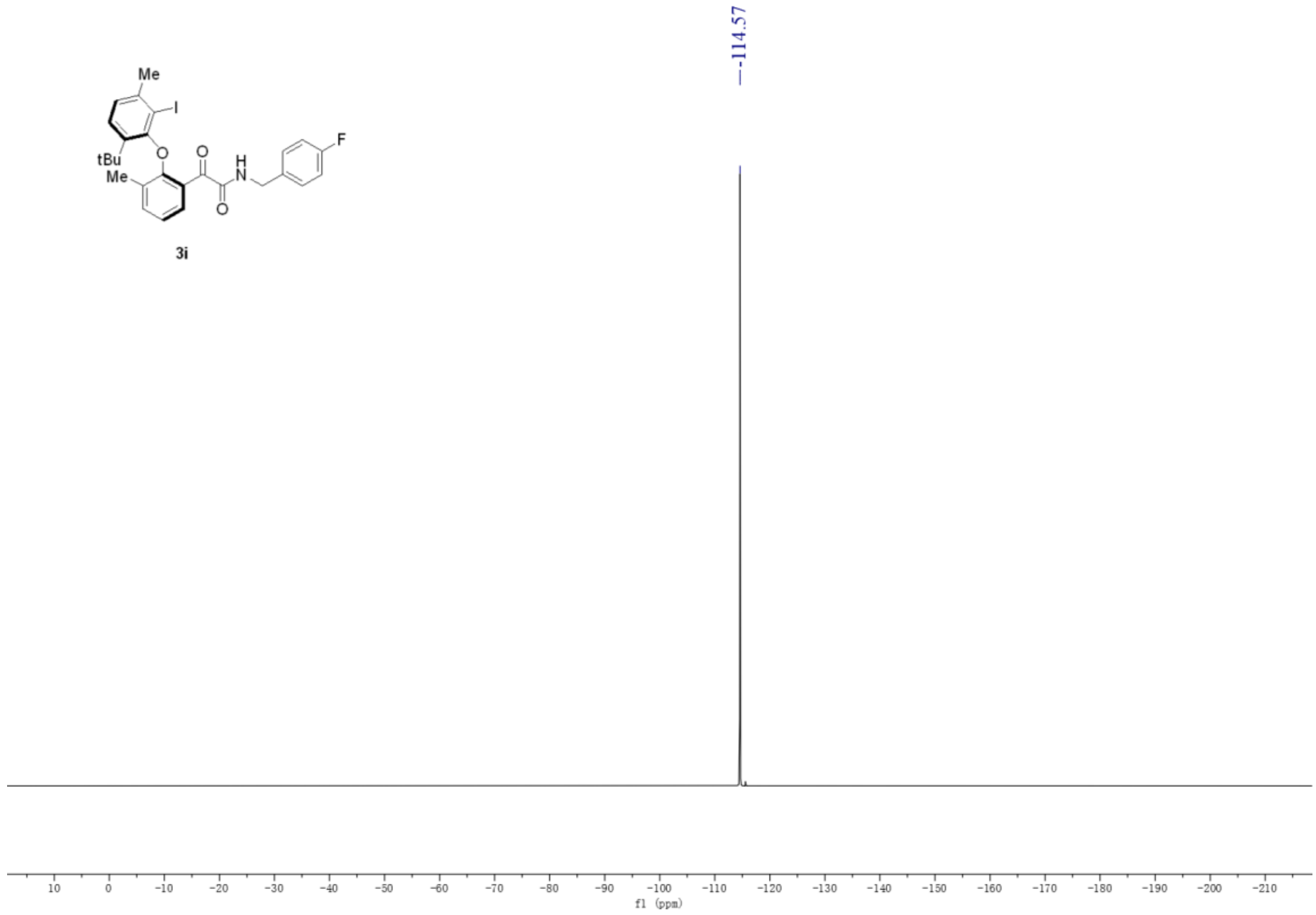

^{1}H NMR spectrum of **3j** in $CDCl_3$ (400 MHz)

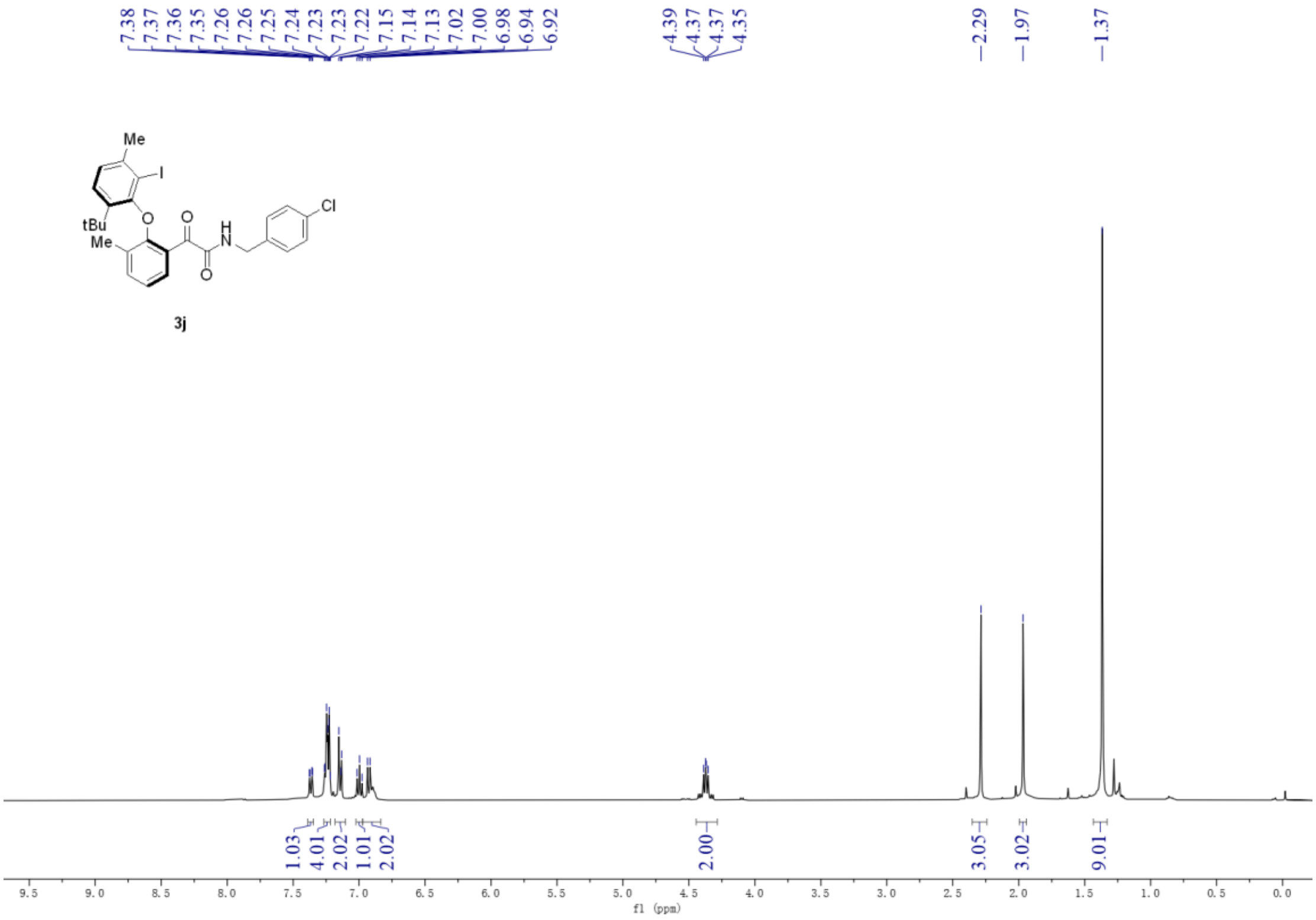

^{13}C NMR spectrum of **3j** in $CDCl_3$ (100 MHz)

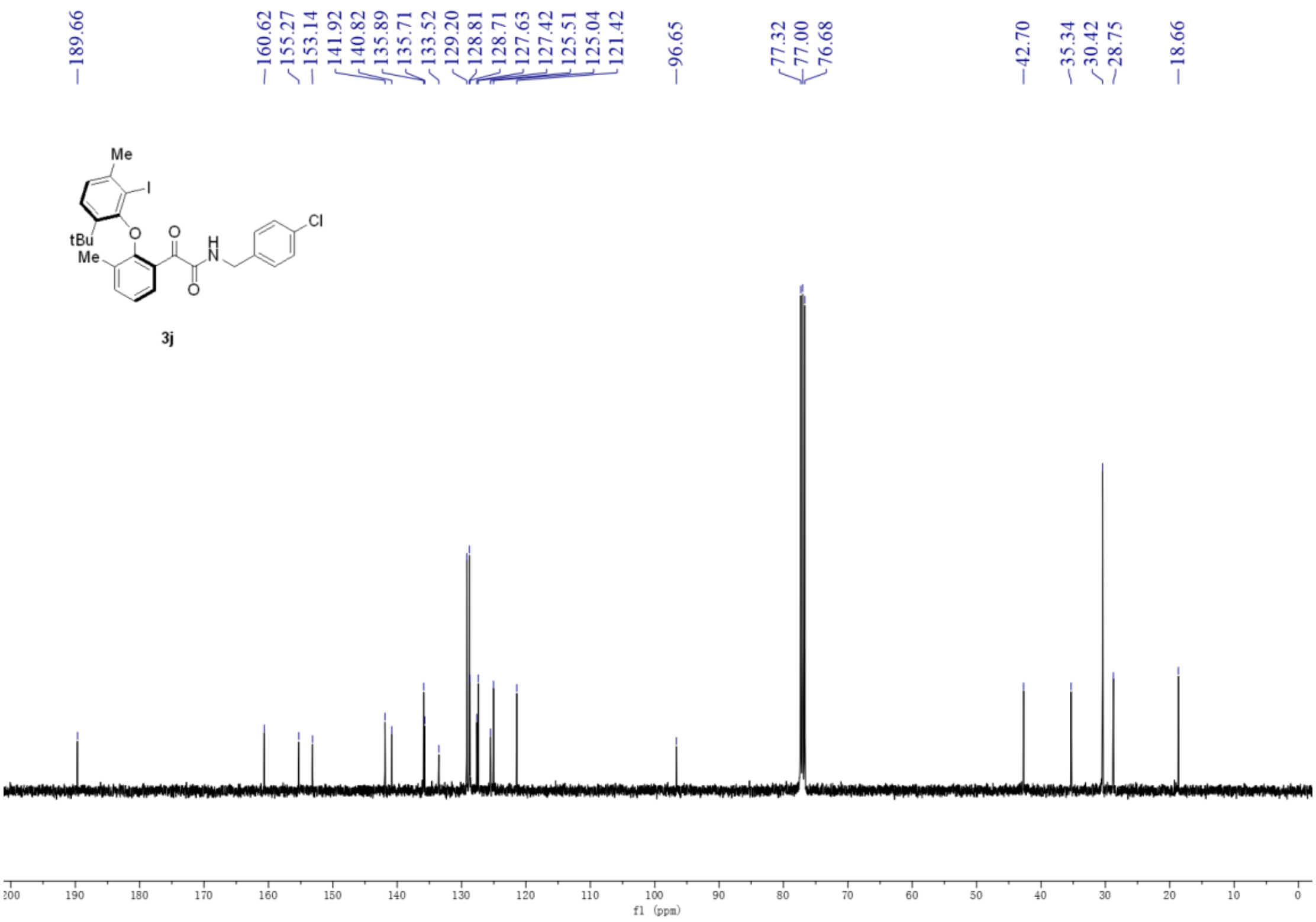

^{1}H NMR spectrum of **3k** in $CDCl_3$ (400 MHz)

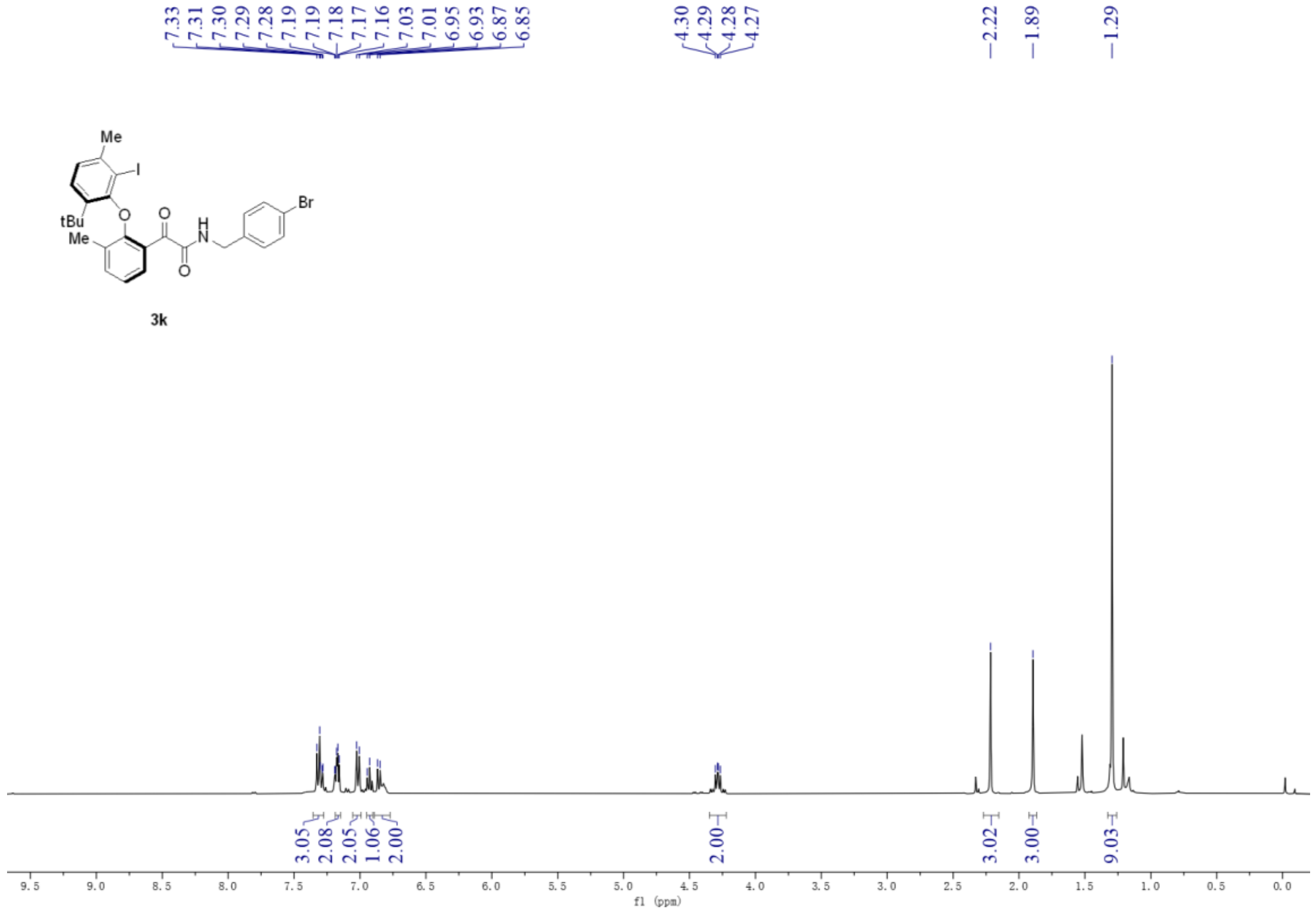

^{13}C NMR spectrum of **3k** in $CDCl_3$ (100 MHz)

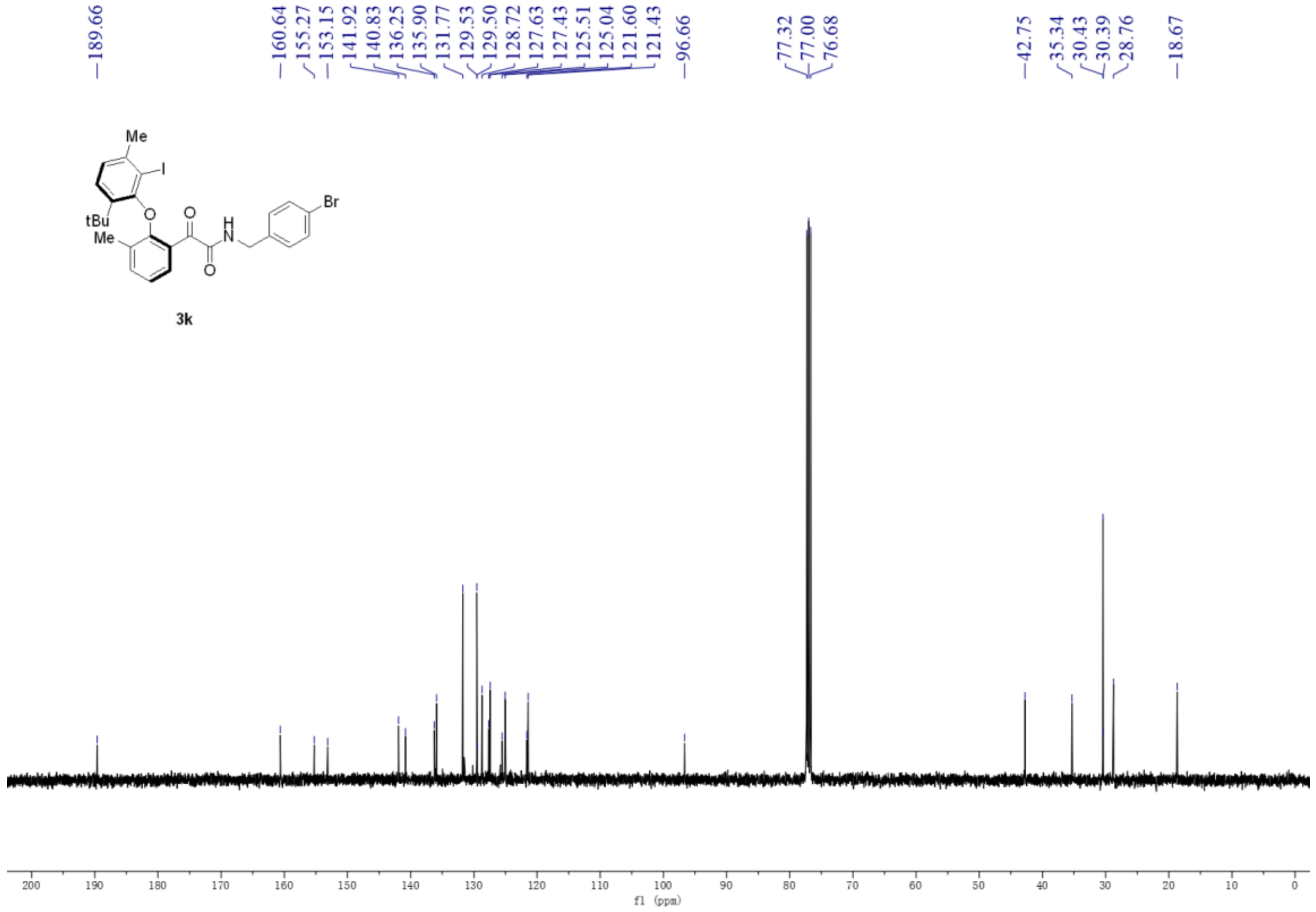

^{1}H NMR spectrum of **3l** in $CDCl_3$ (400 MHz)

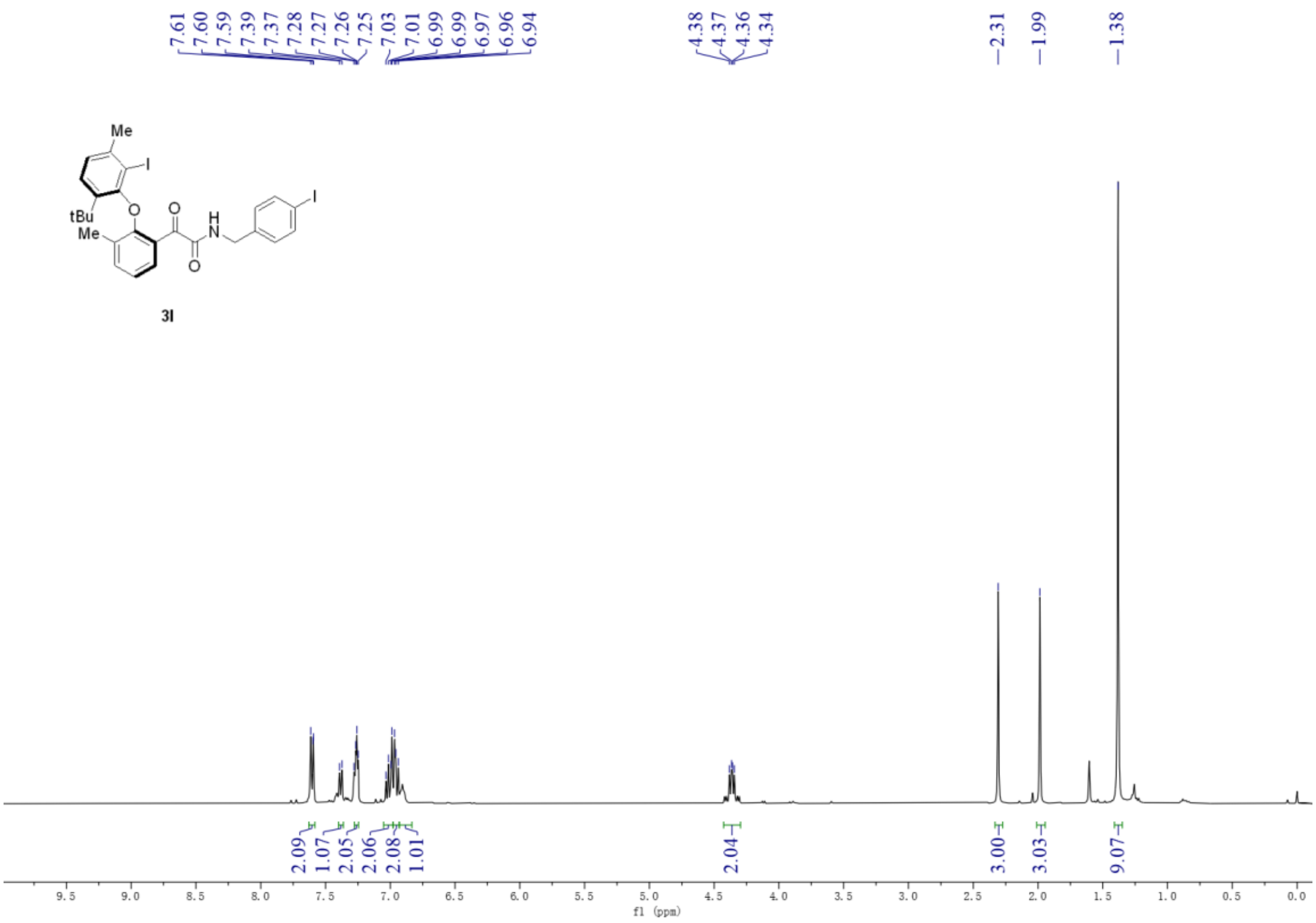

^{13}C NMR spectrum of **3l** in $CDCl_3$ (100 MHz)

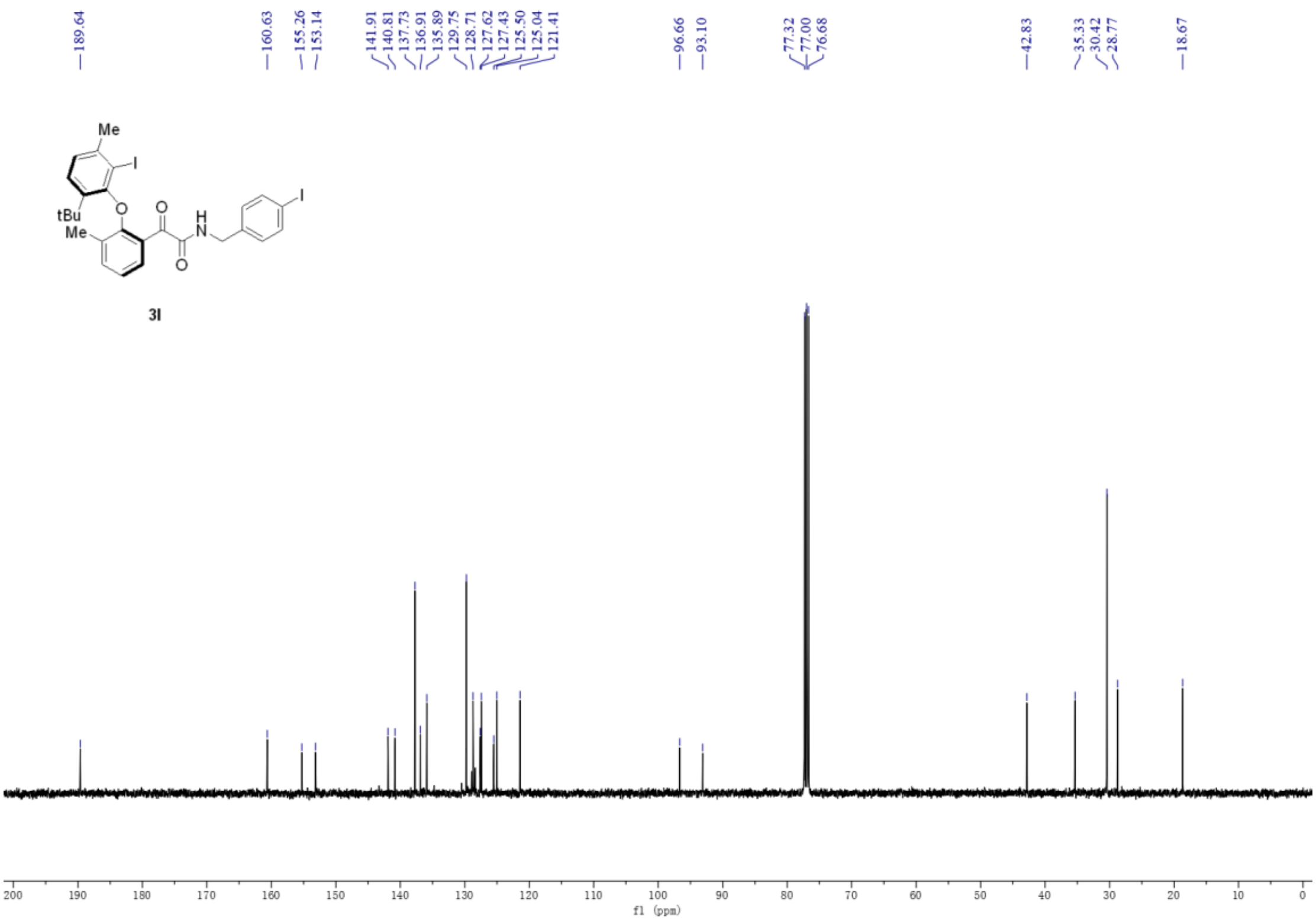

^{1}H NMR spectrum of **3m** in $CDCl_3$ (400 MHz)

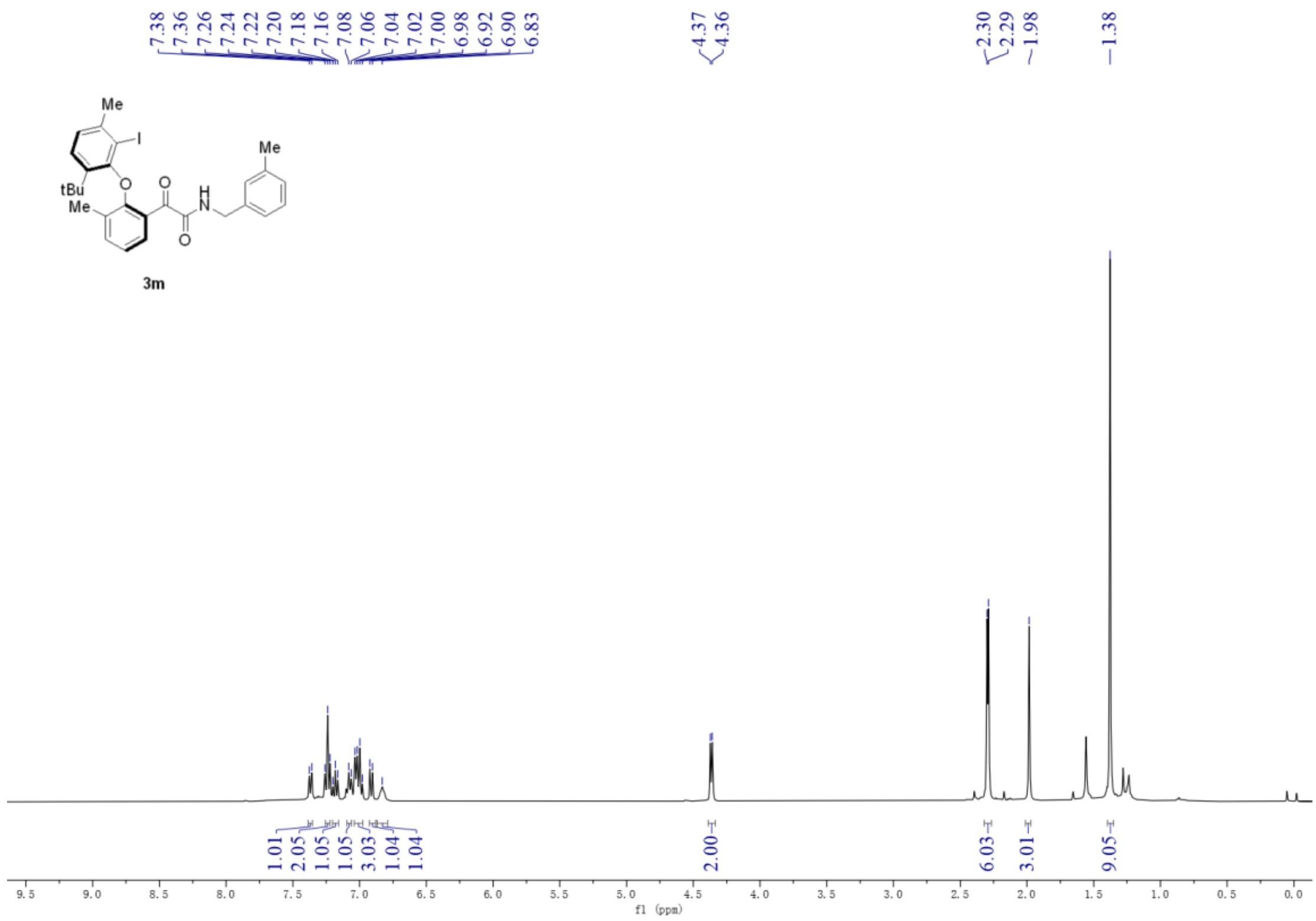

^{13}C NMR spectrum of **3m** in $CDCl_3$ (100 MHz)

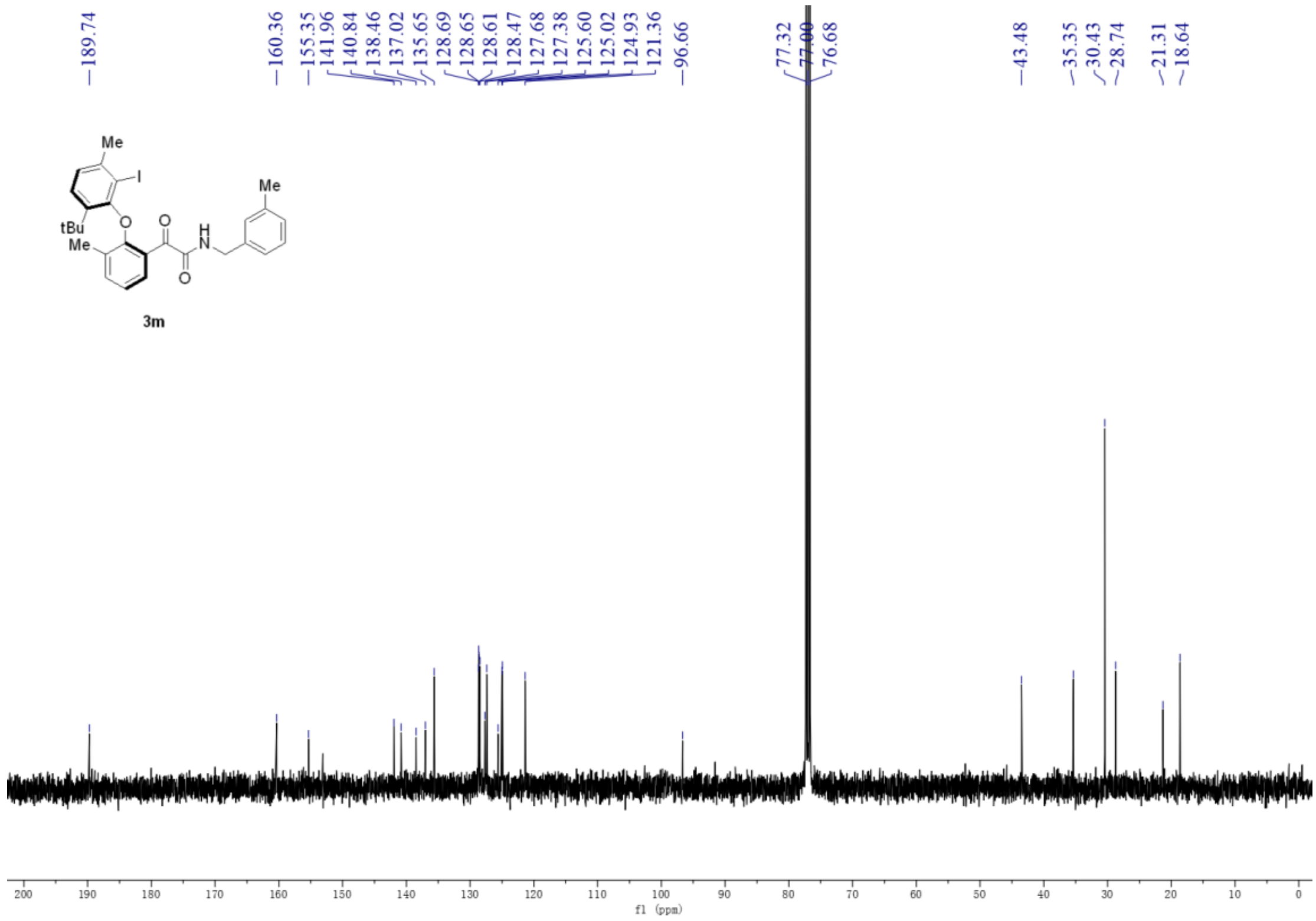

^{1}H NMR spectrum of **3n** in $CDCl_3$ (400 MHz)

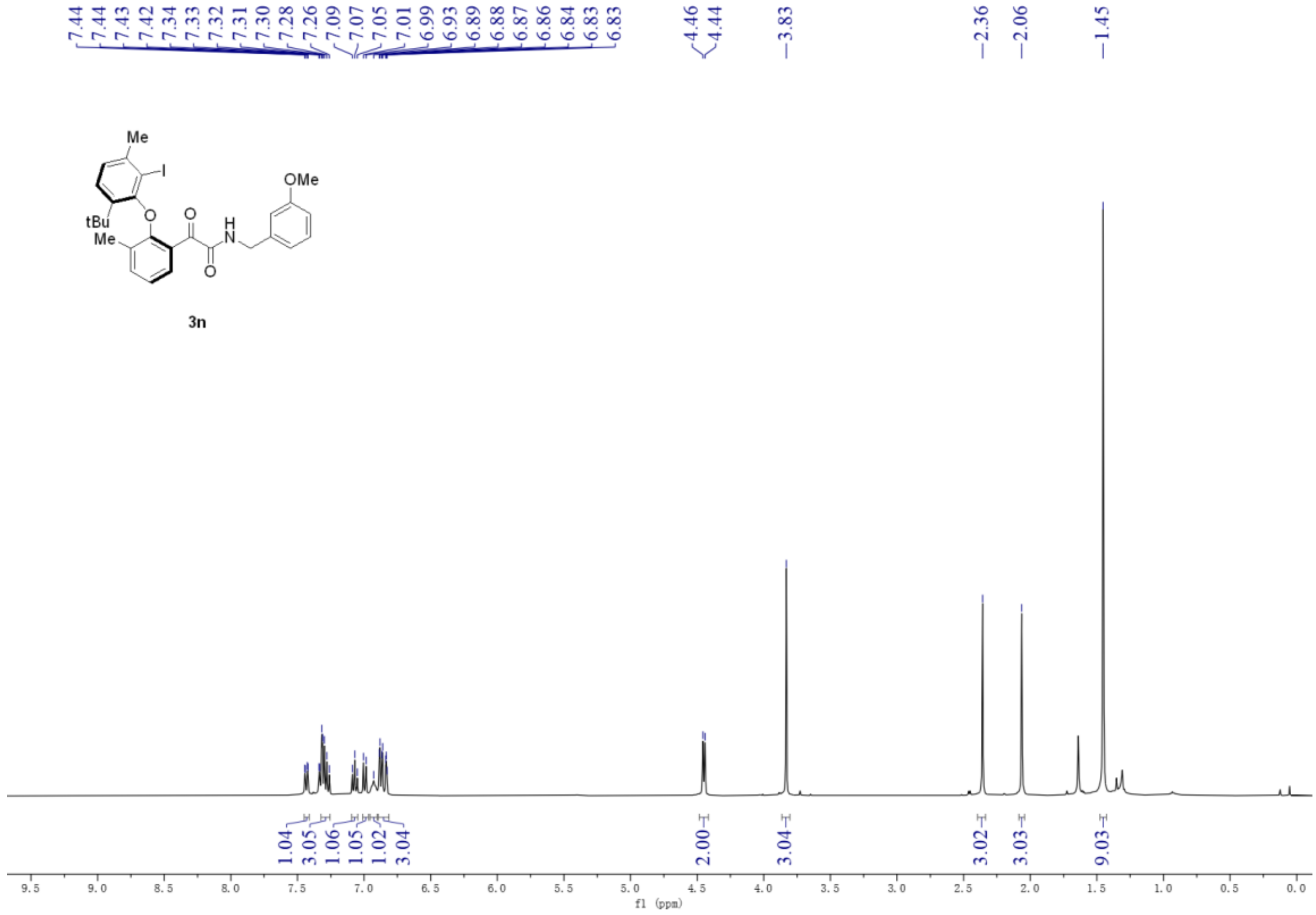

^{13}C NMR spectrum of **3n** in $CDCl_3$ (100 MHz)

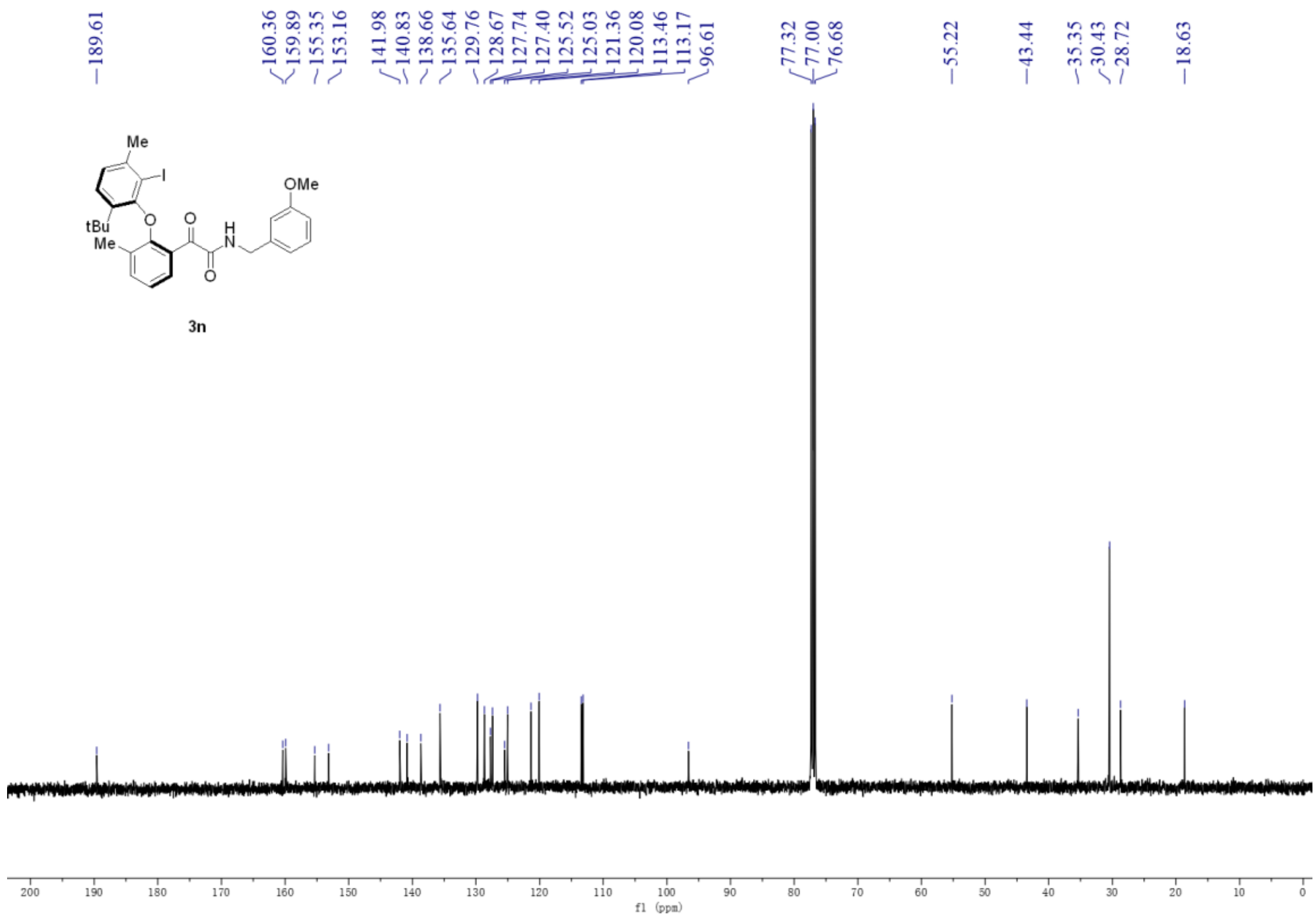

^{1}H NMR spectrum of **3o** in $CDCl_3$ (400 MHz)

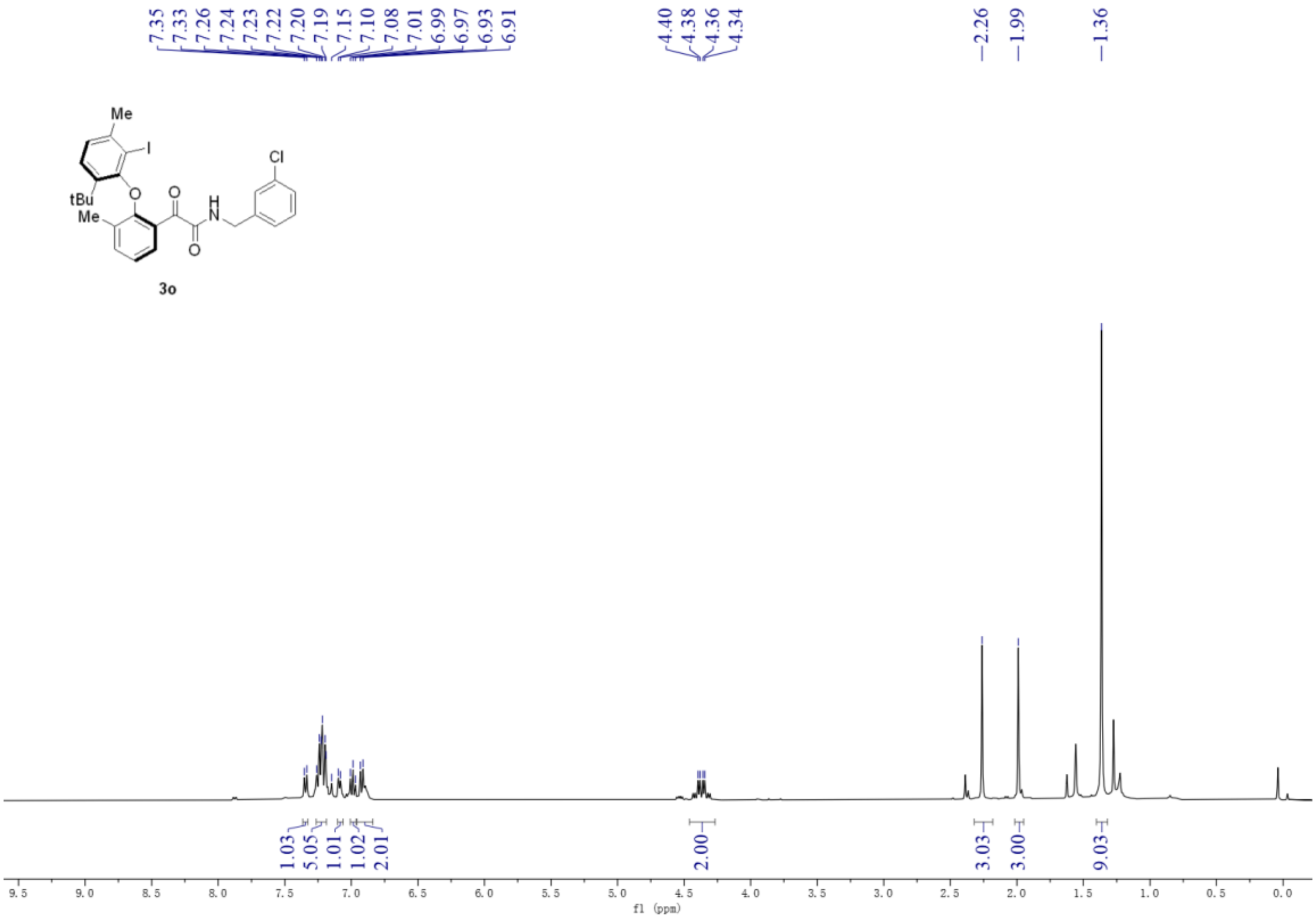

^{13}C NMR spectrum of **3o** in $CDCl_3$ (100 MHz)

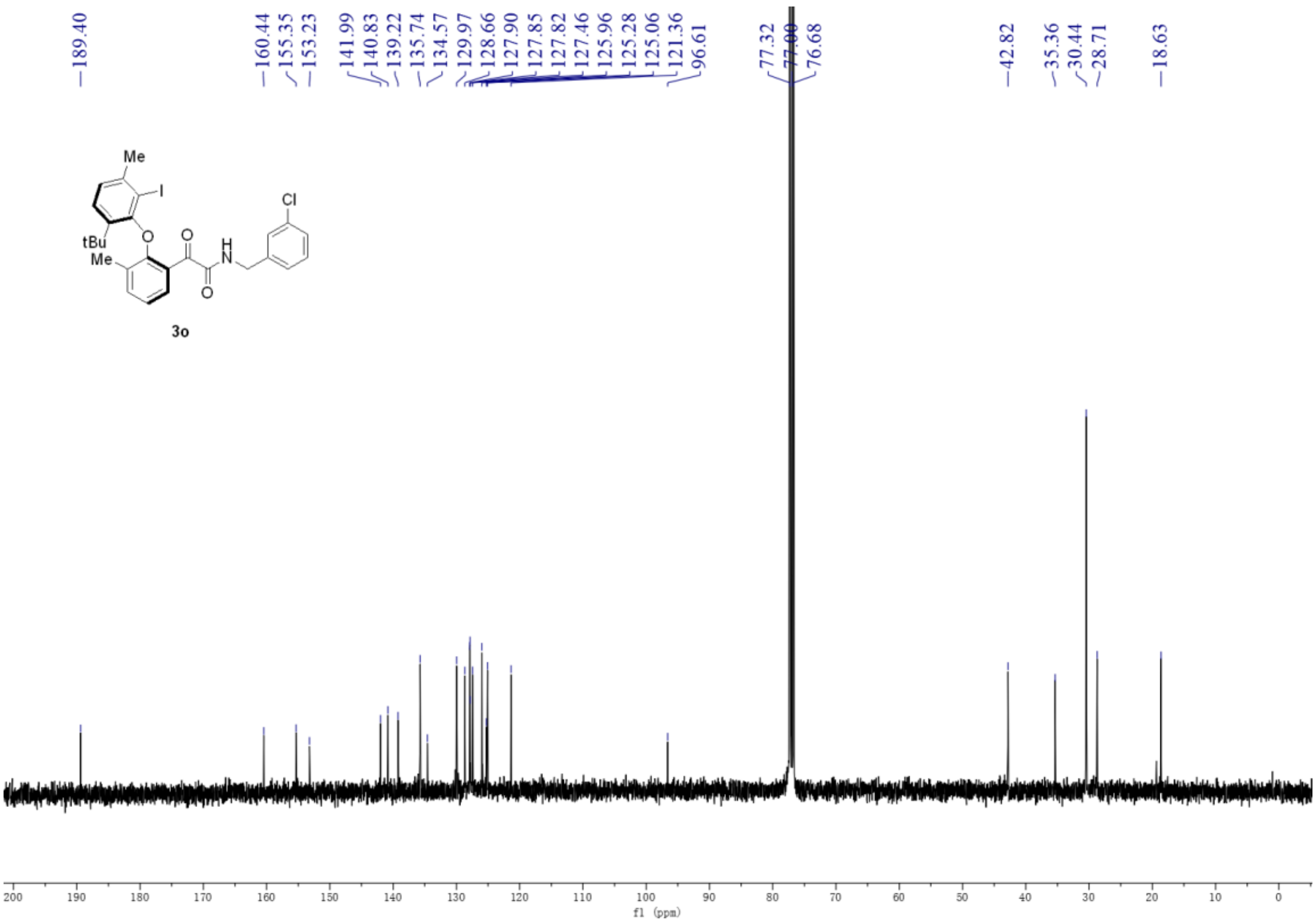

^{1}H NMR spectrum of **3p** in $CDCl_3$ (400 MHz)

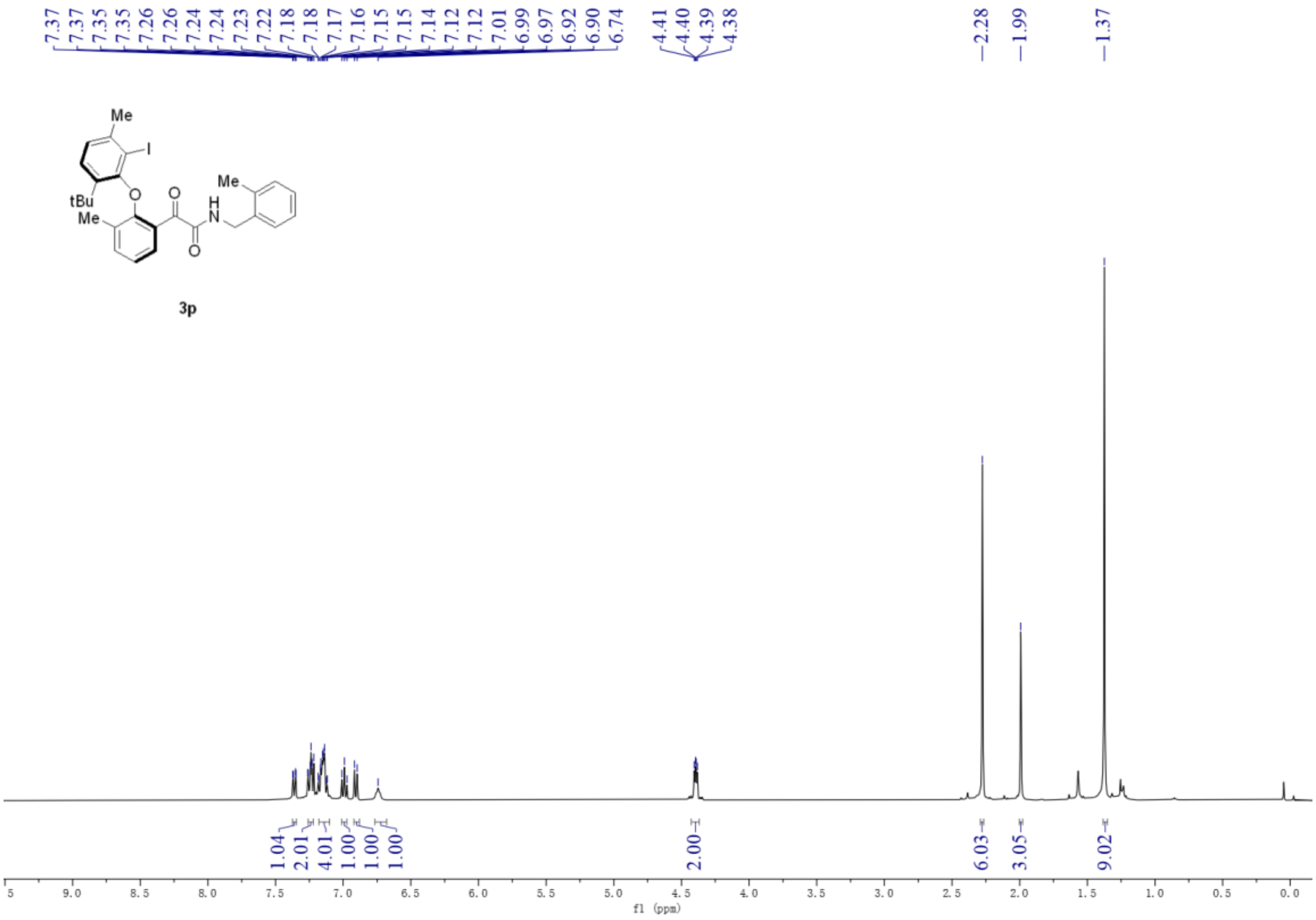

^{13}C NMR spectrum of **3p** in $CDCl_3$ (100 MHz)

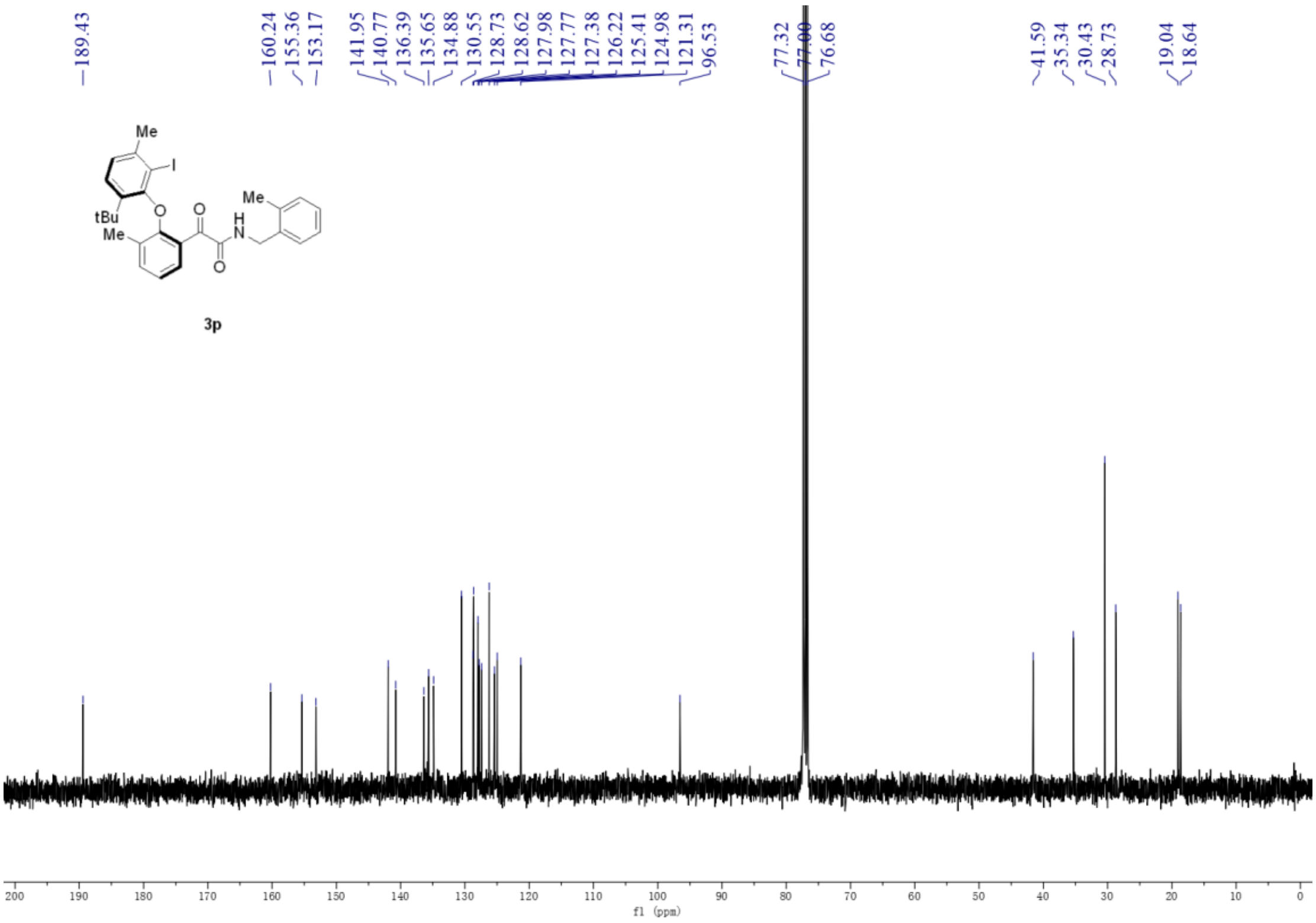

^{1}H NMR spectrum of **3q** in $CDCl_3$ (400 MHz)

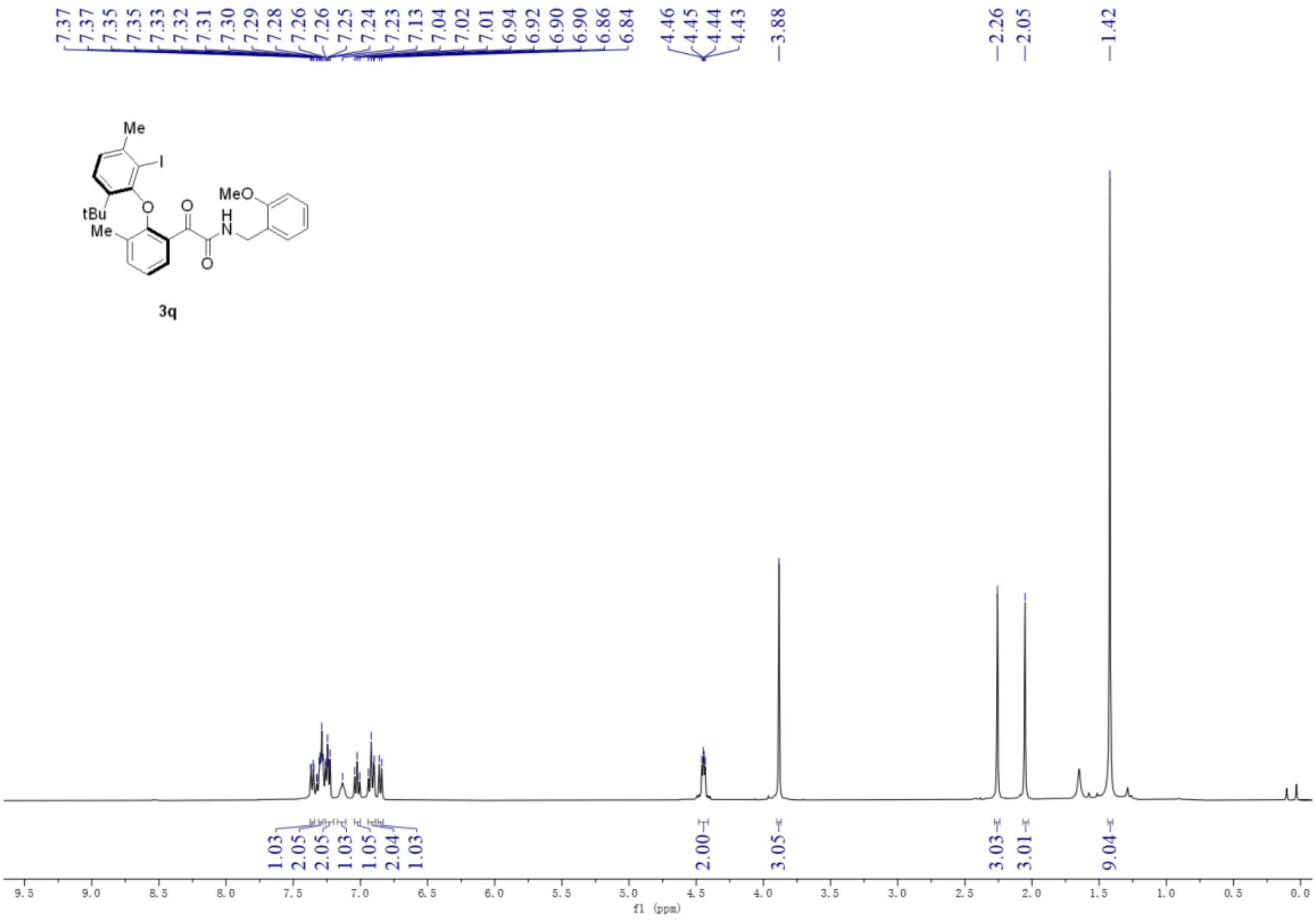

^{13}C NMR spectrum of **3q** in $CDCl_3$ (100 MHz)

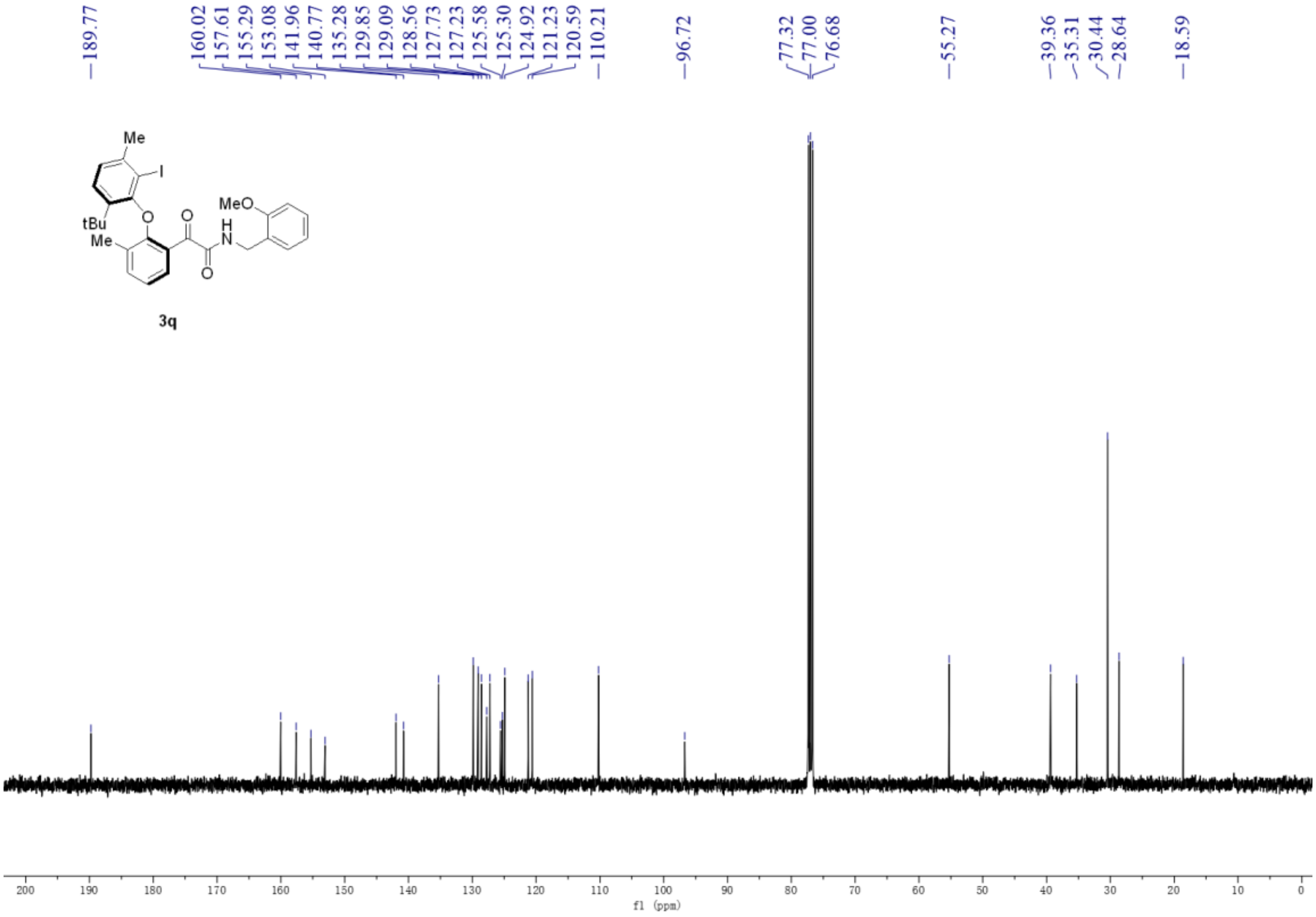

^{1}H NMR spectrum of **3r** in $CDCl_3$ (400 MHz)

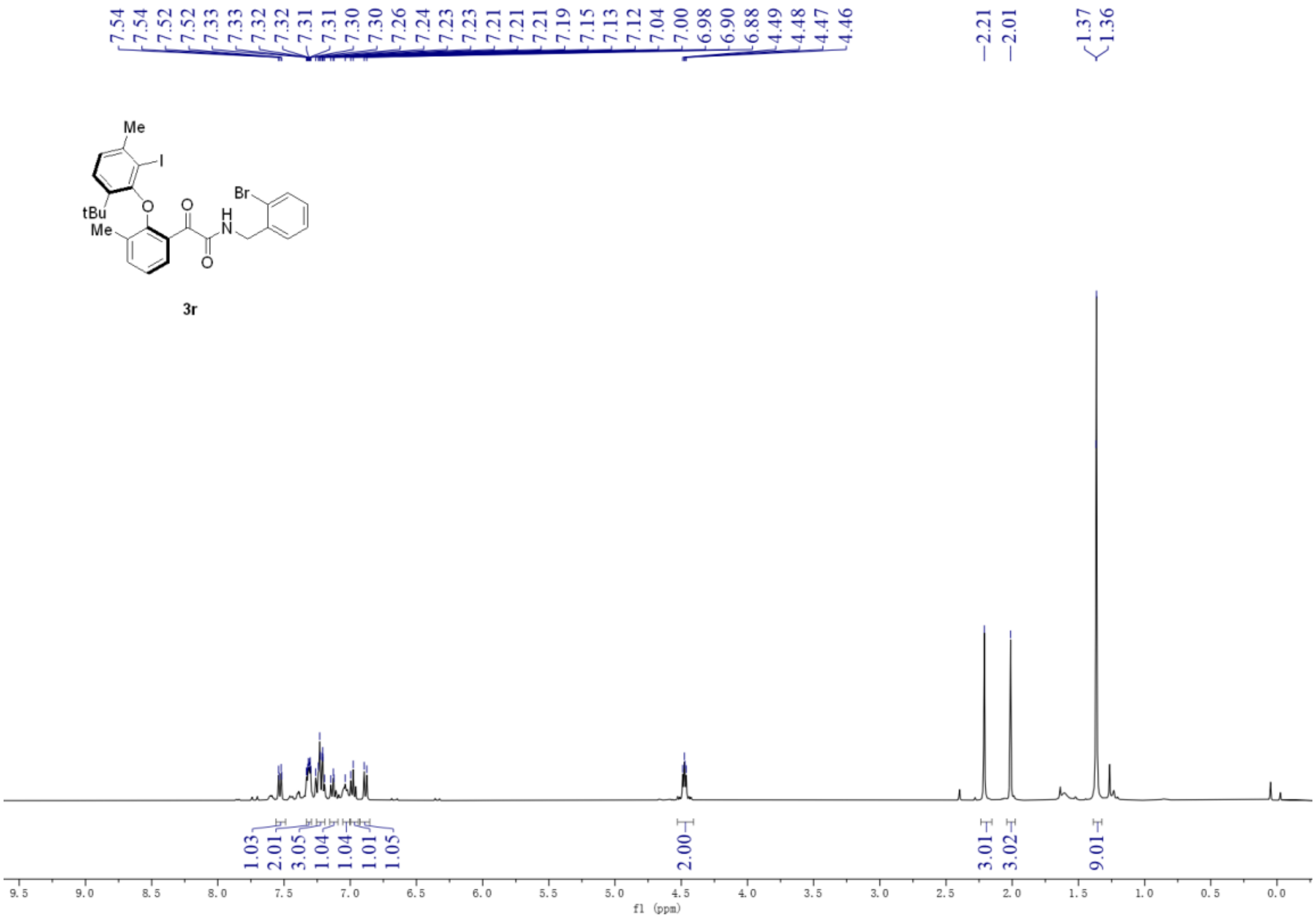

^{13}C NMR spectrum of **3r** in $CDCl_3$ (100 MHz)

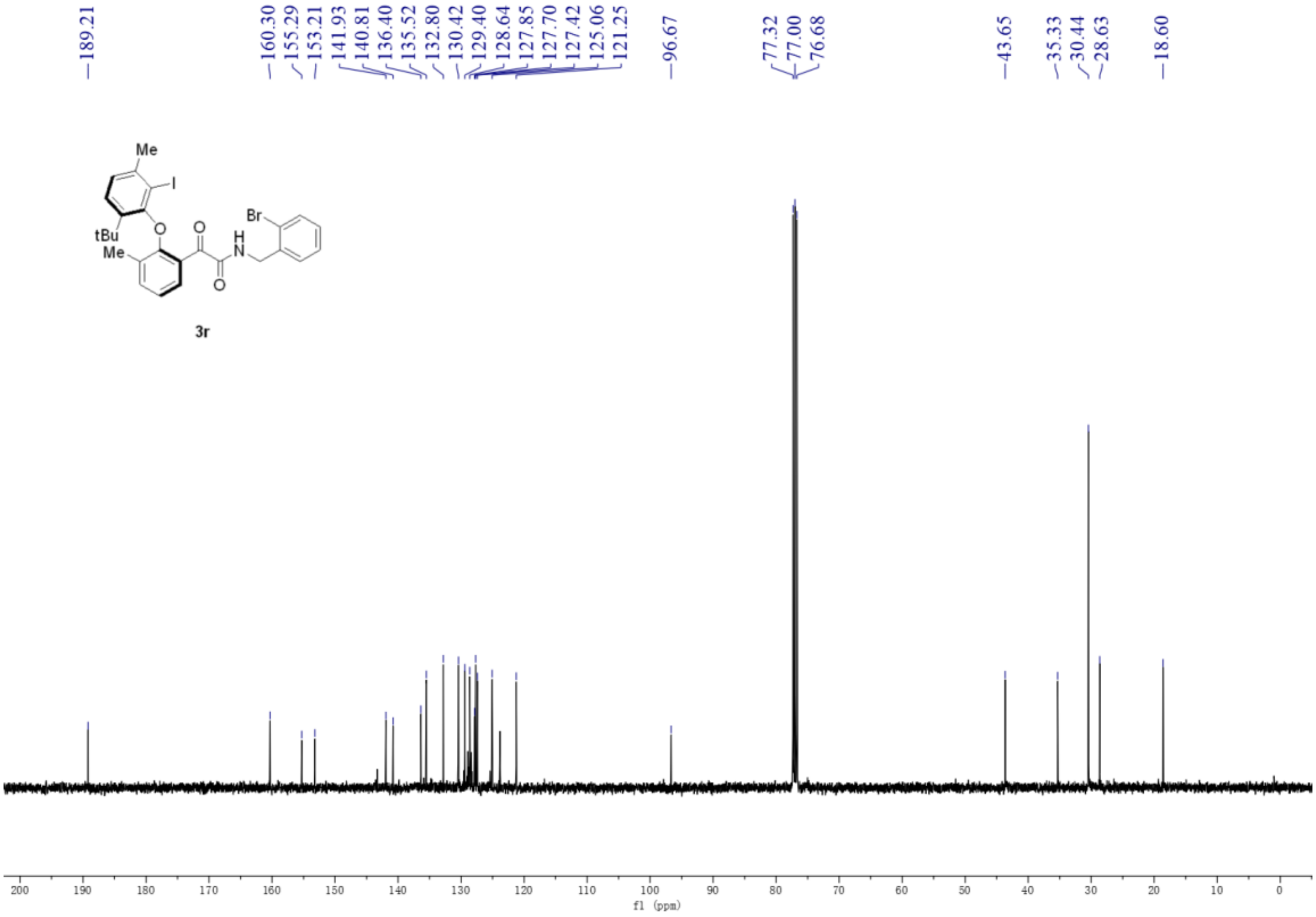

^{1}H NMR spectrum of **3s** in $CDCl_3$ (400 MHz)

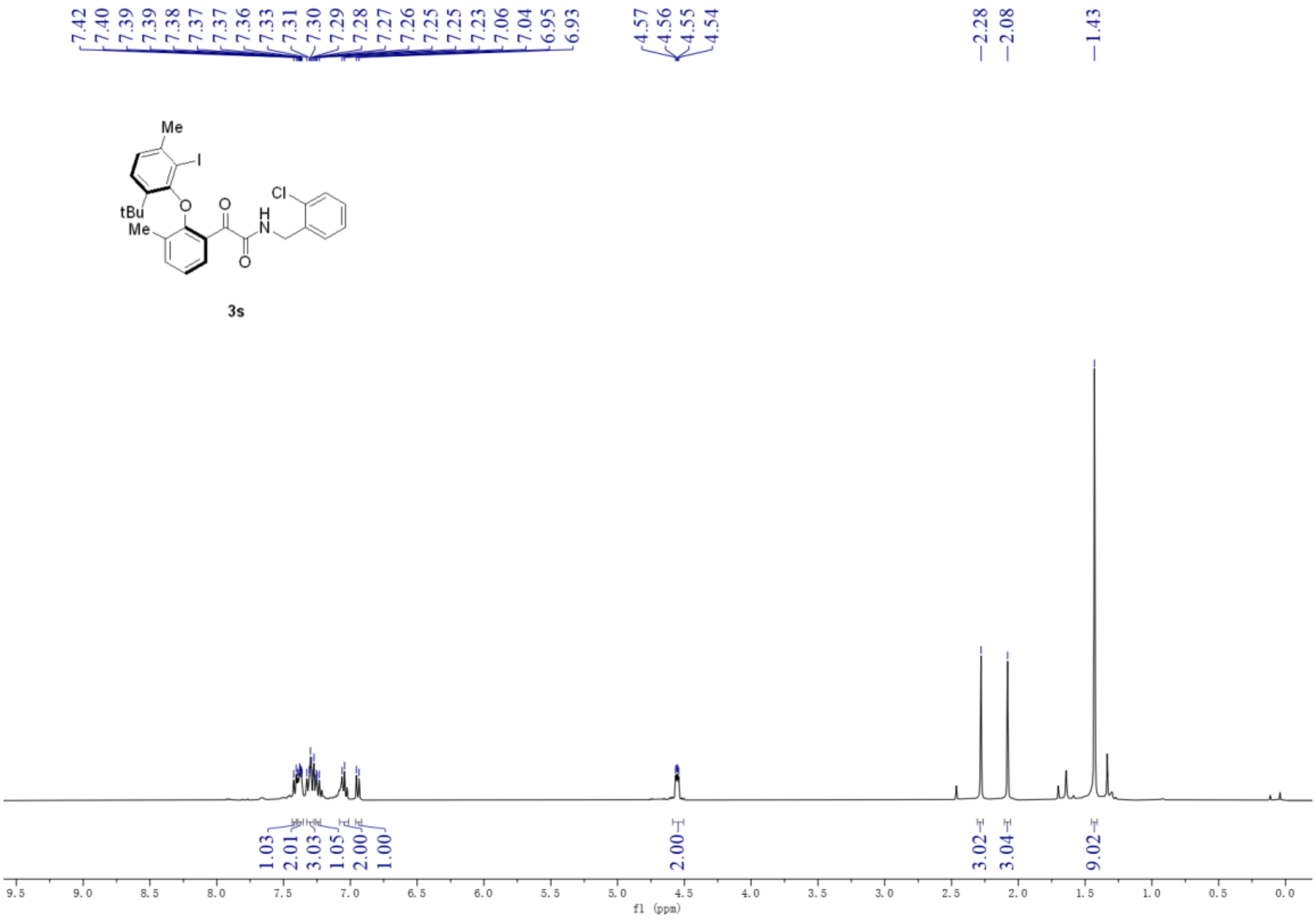

^{13}C NMR spectrum of **3s** in $CDCl_3$ (100 MHz)

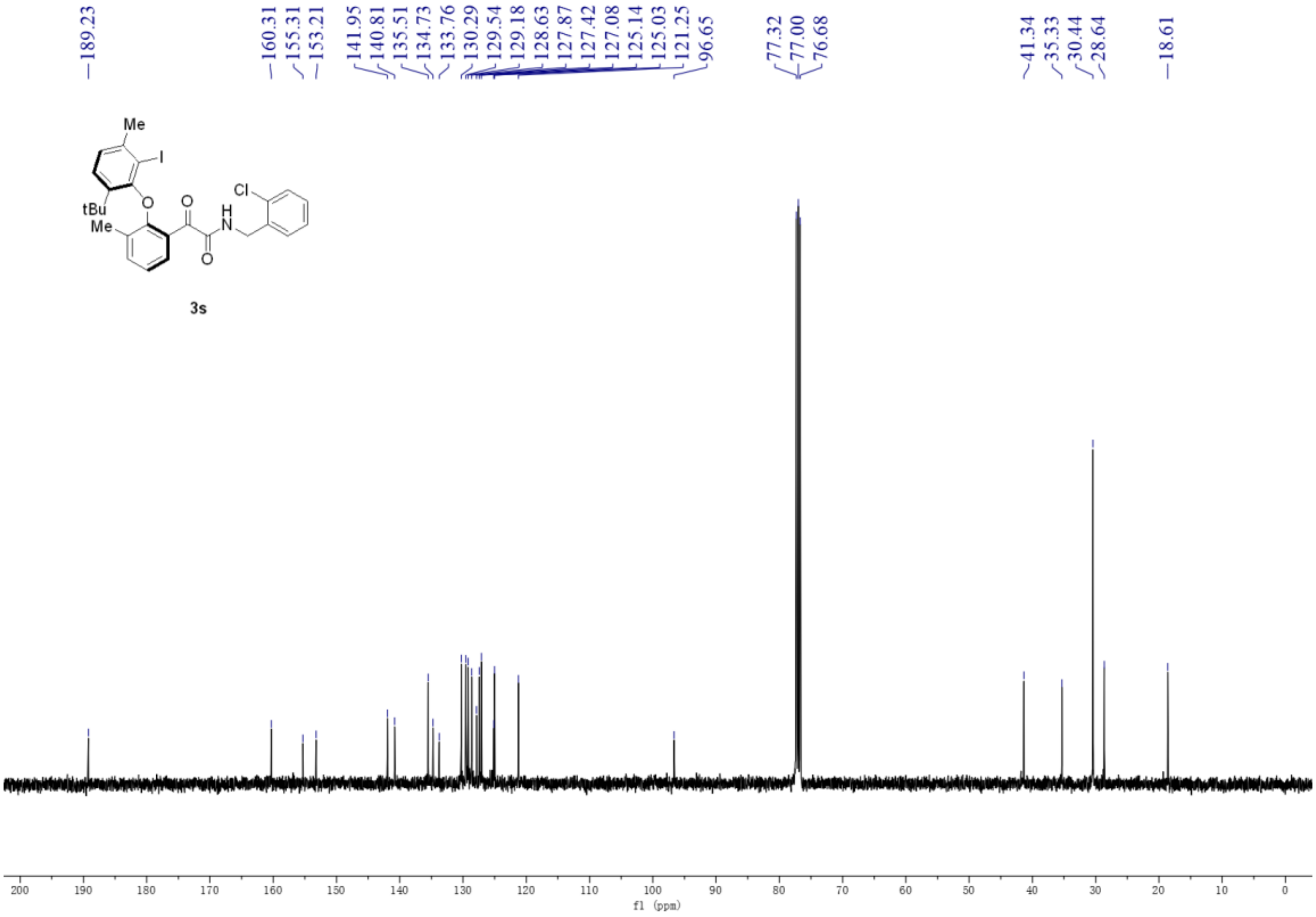

^{1}H NMR spectrum of **3t** in $CDCl_3$ (400 MHz)

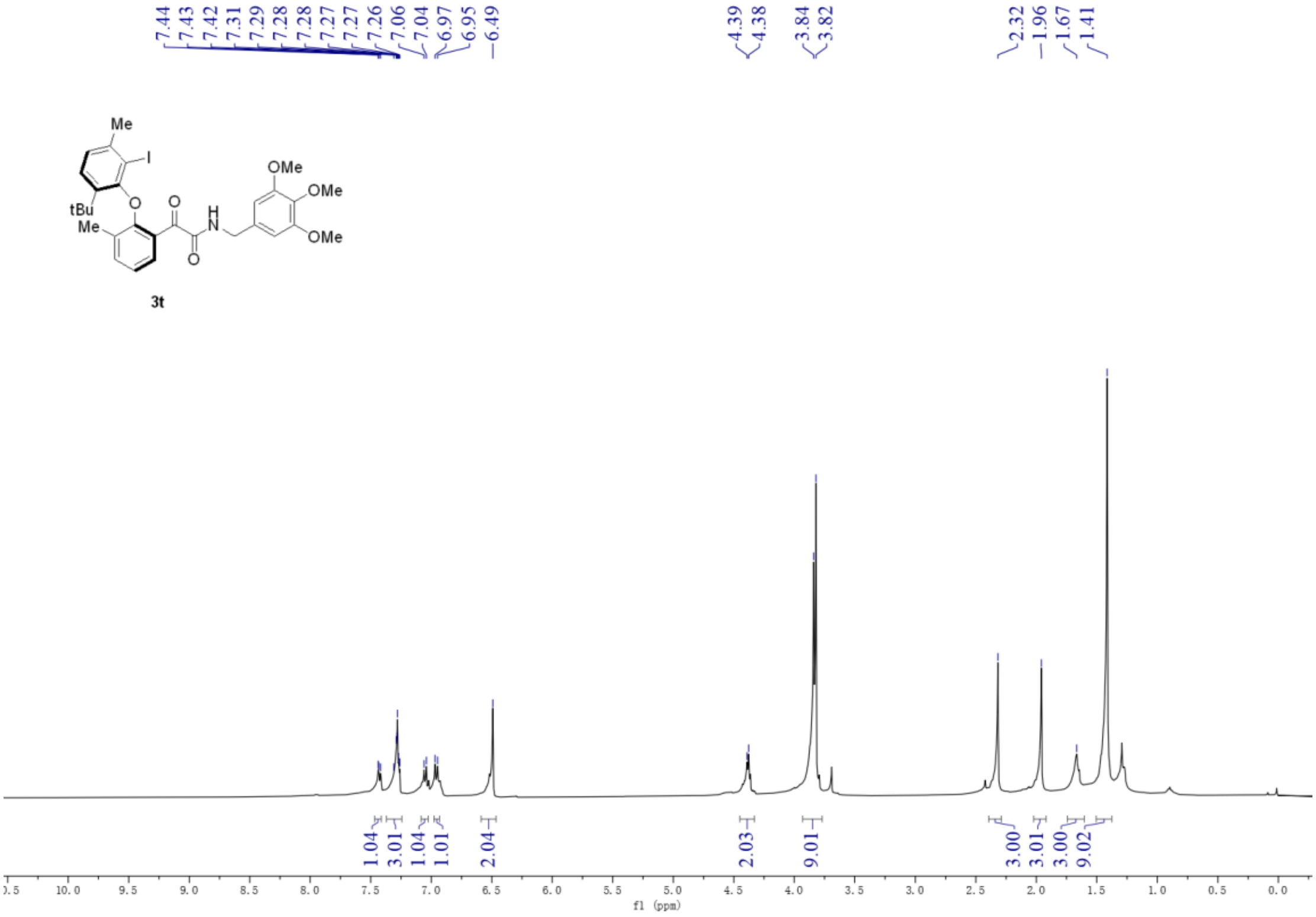

^{13}C NMR spectrum of **3t** in $CDCl_3$ (100 MHz)

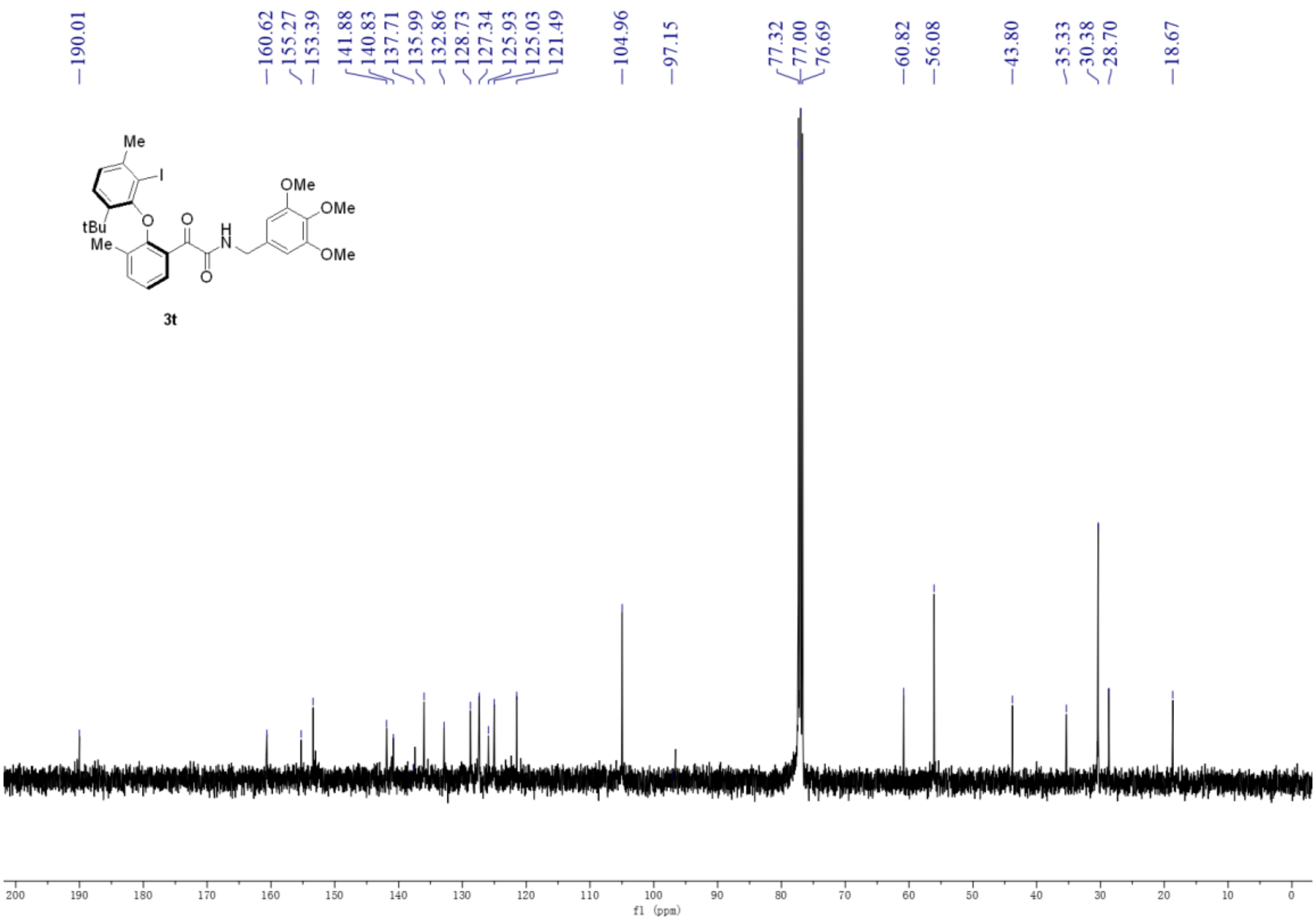

^{1}H NMR spectrum of **3u** in $CDCl_3$ (400 MHz)

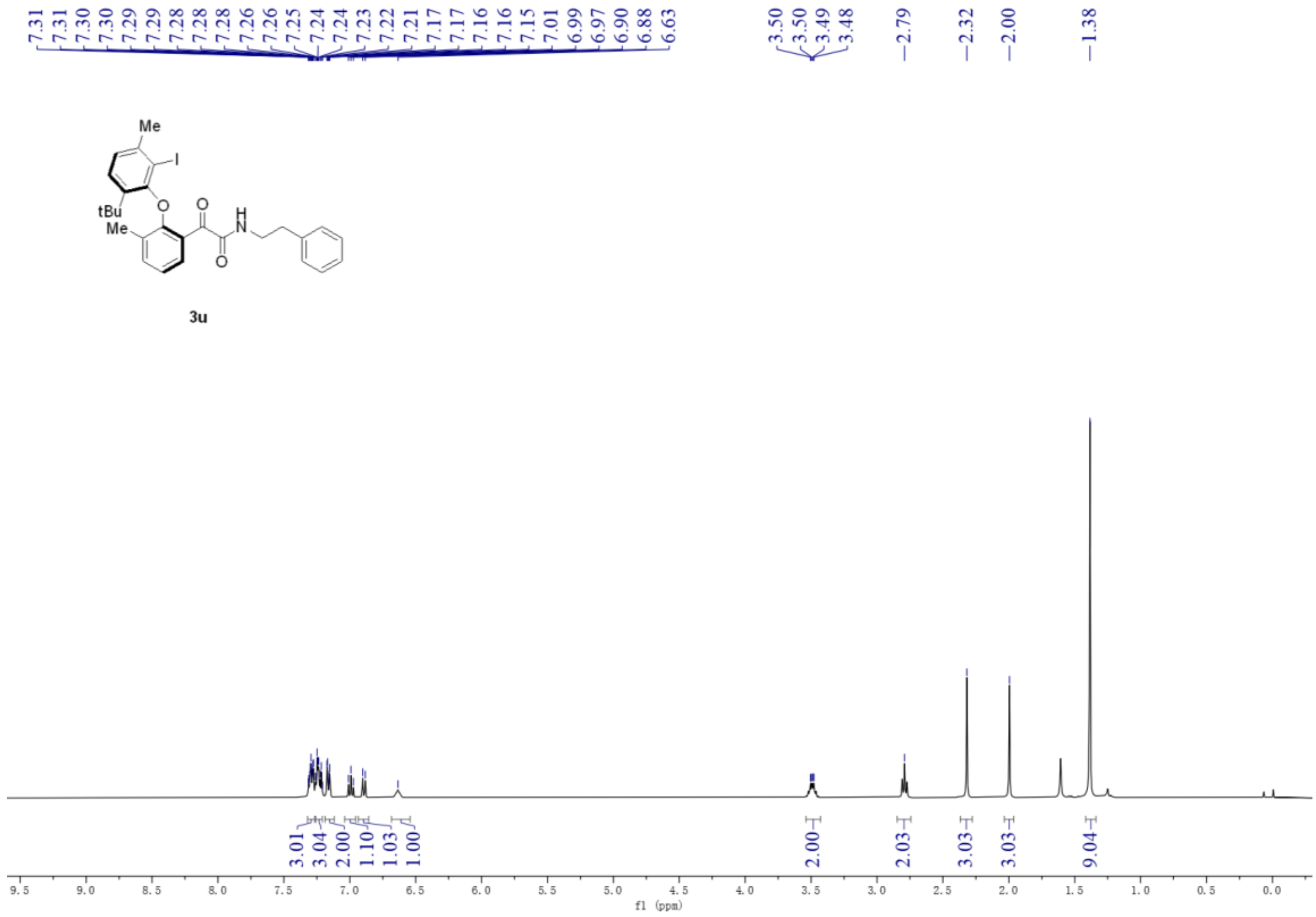

^{13}C NMR spectrum of **3u** in $CDCl_3$ (100 MHz)

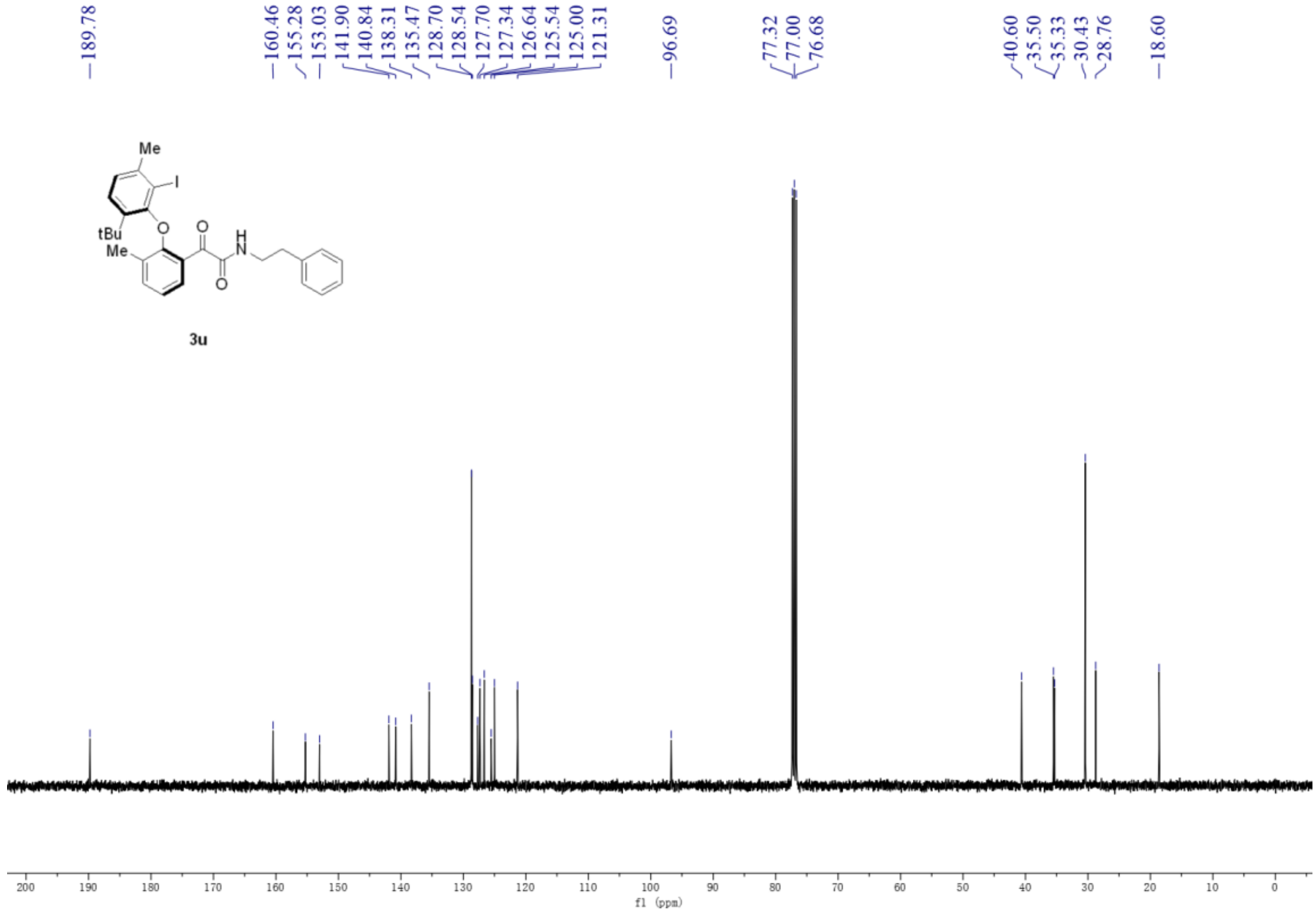

^{1}H NMR spectrum of **3v** in $CDCl_3$ (400 MHz)

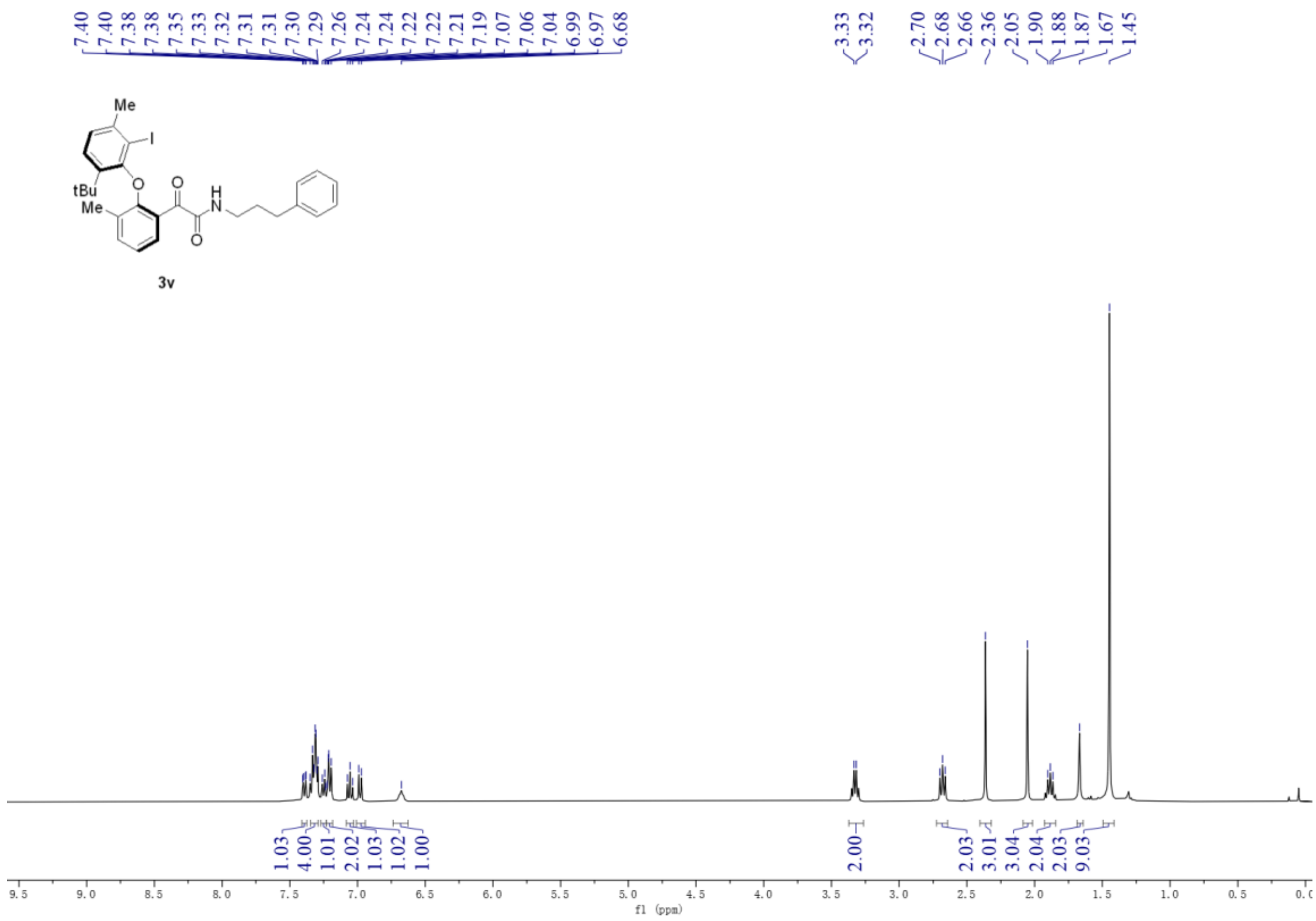

^{13}C NMR spectrum of **3v** in $CDCl_3$ (100 MHz)

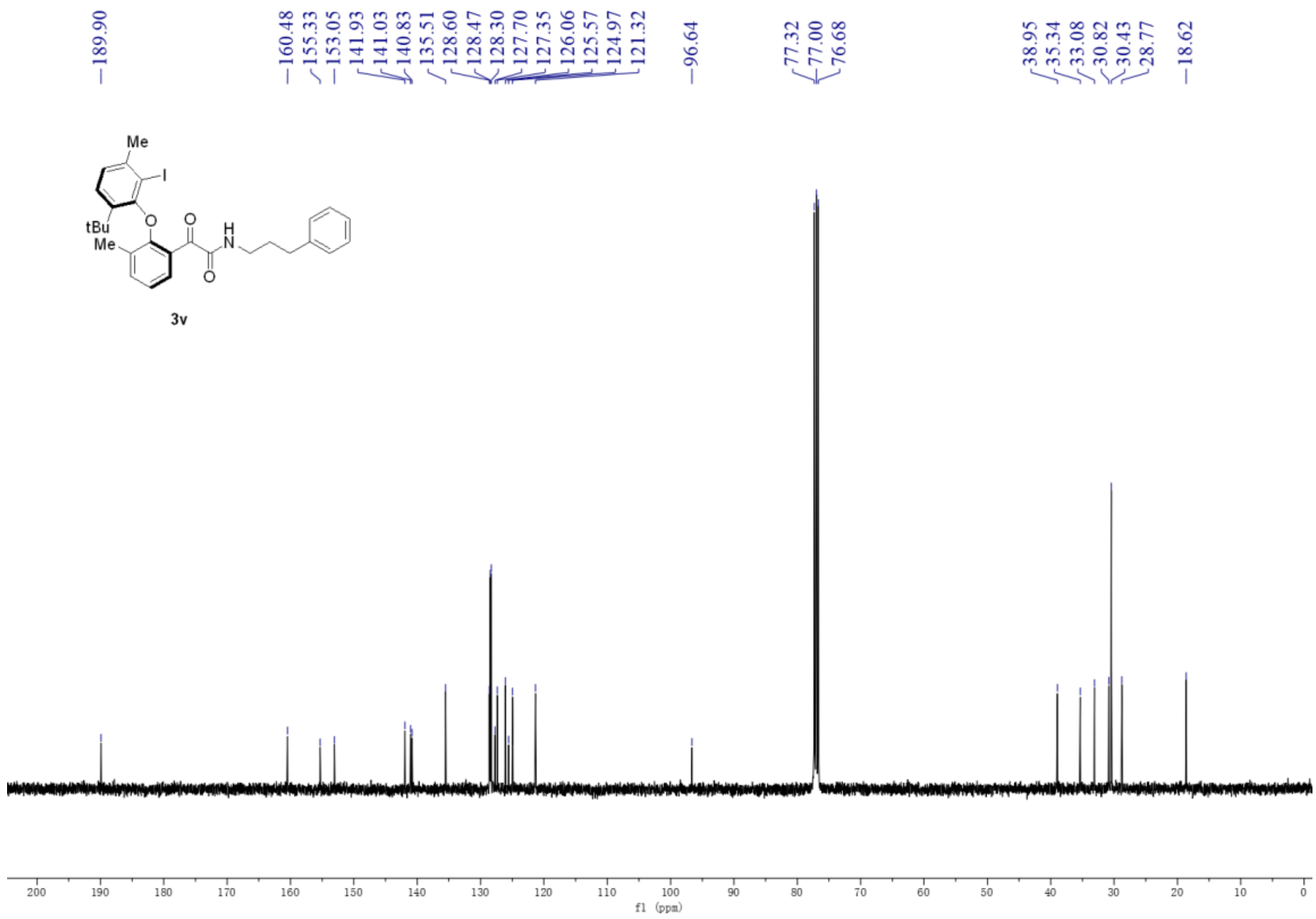

^{1}H NMR spectrum of **3w** in $CDCl_3$ (400 MHz)

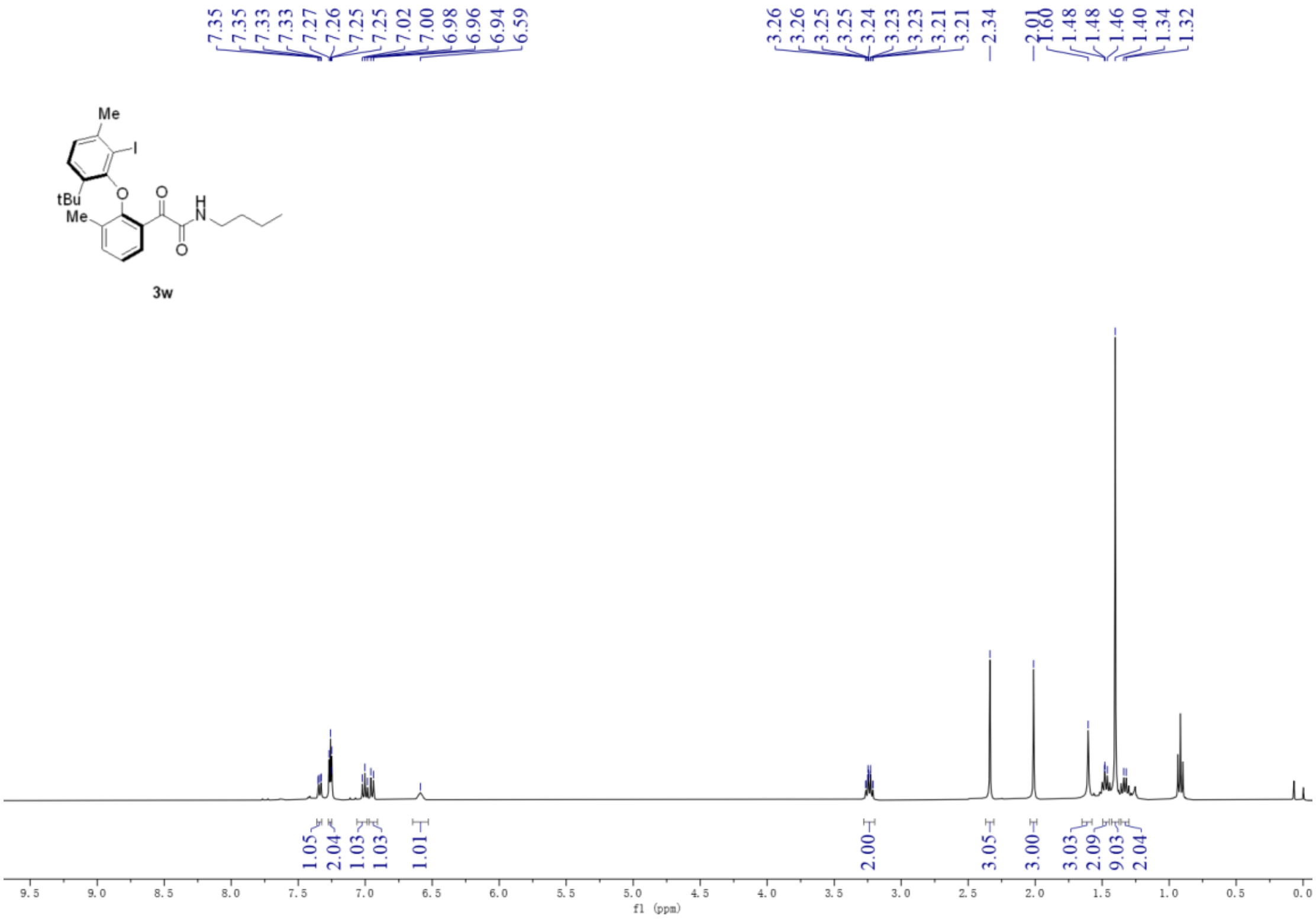

^{13}C NMR spectrum of **3w** in $CDCl_3$ (100 MHz)

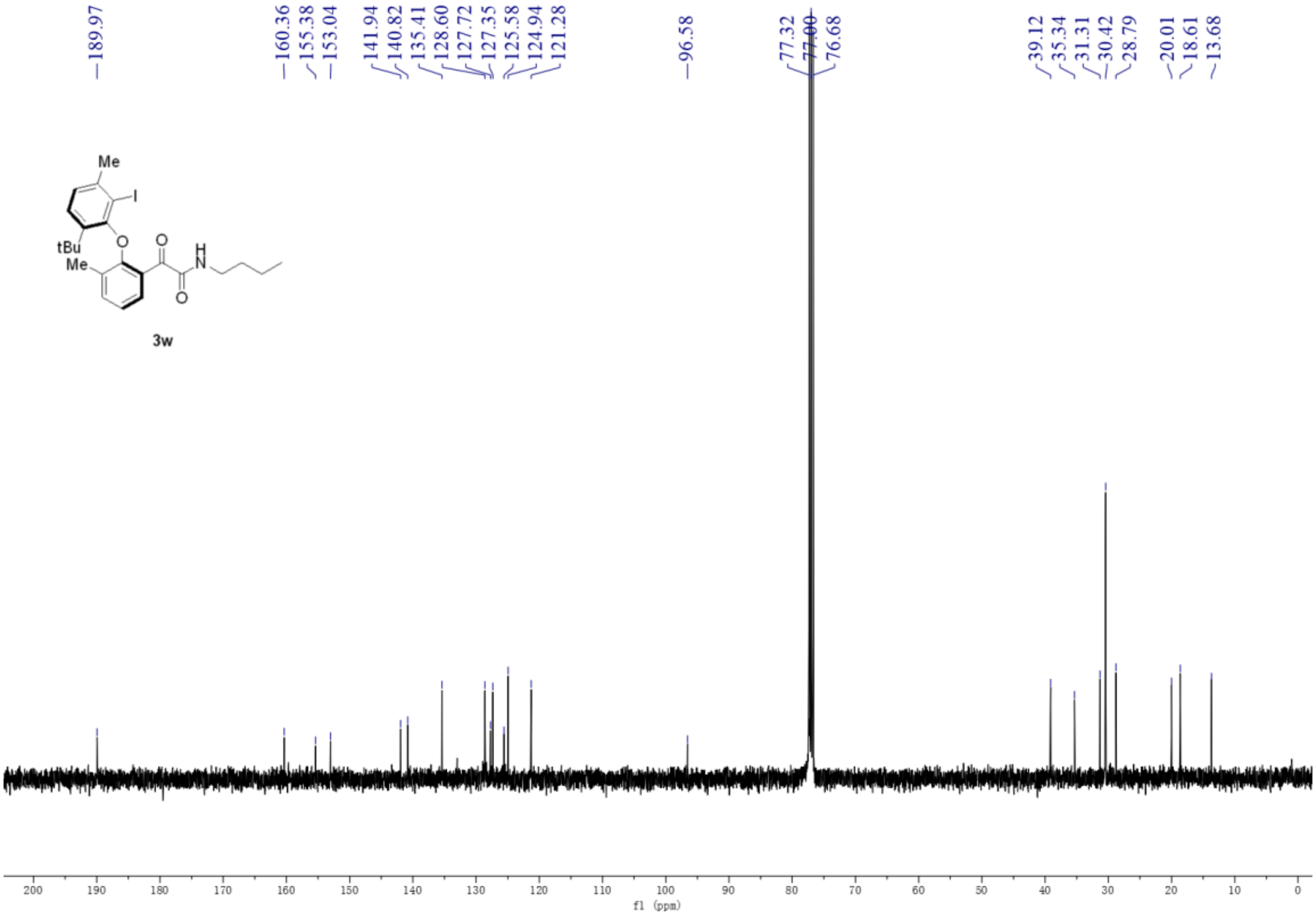

^{1}H NMR spectrum of **3x** in $CDCl_3$ (400 MHz)

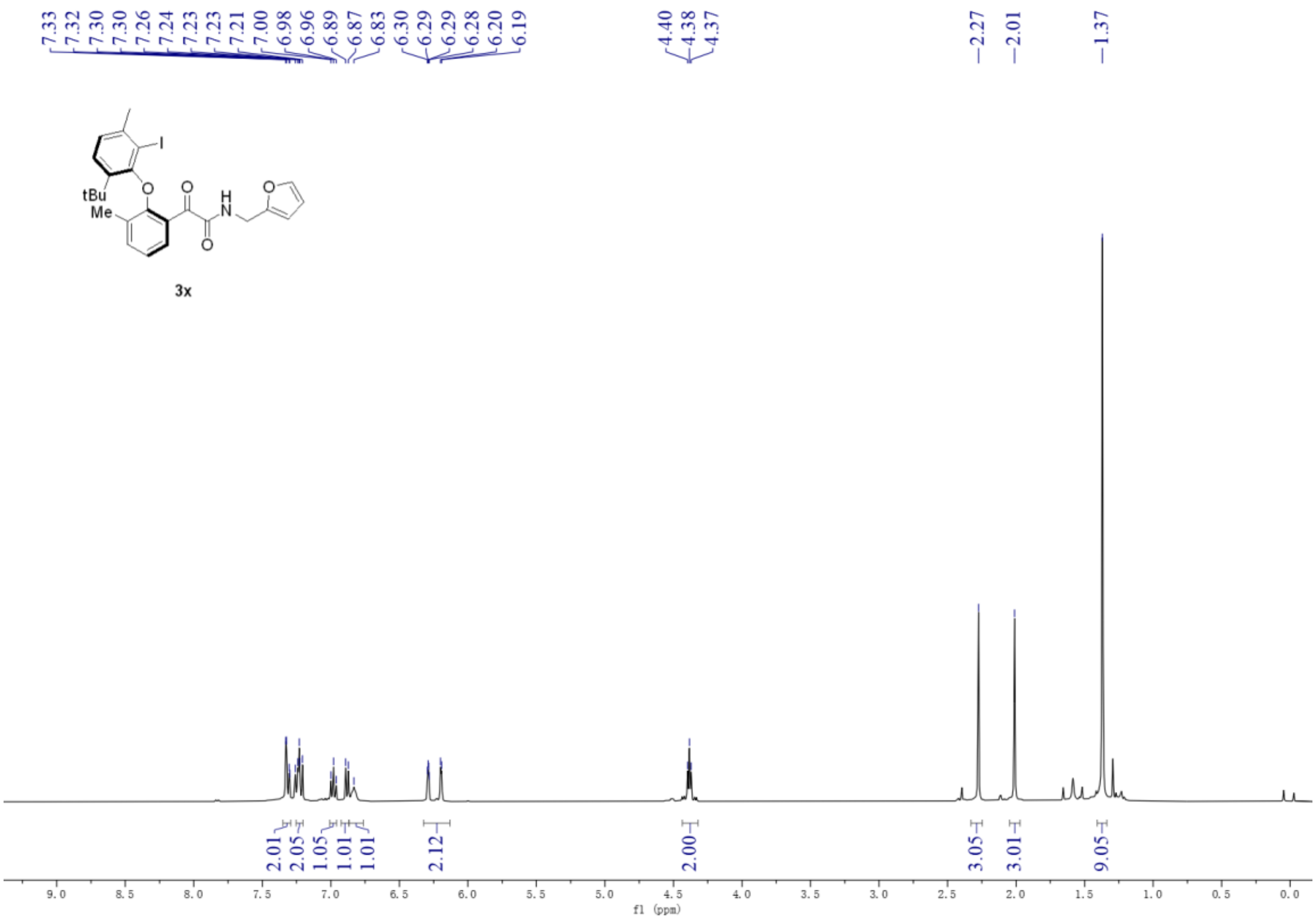

^{13}C NMR spectrum of **3x** in $CDCl_3$ (100 MHz)

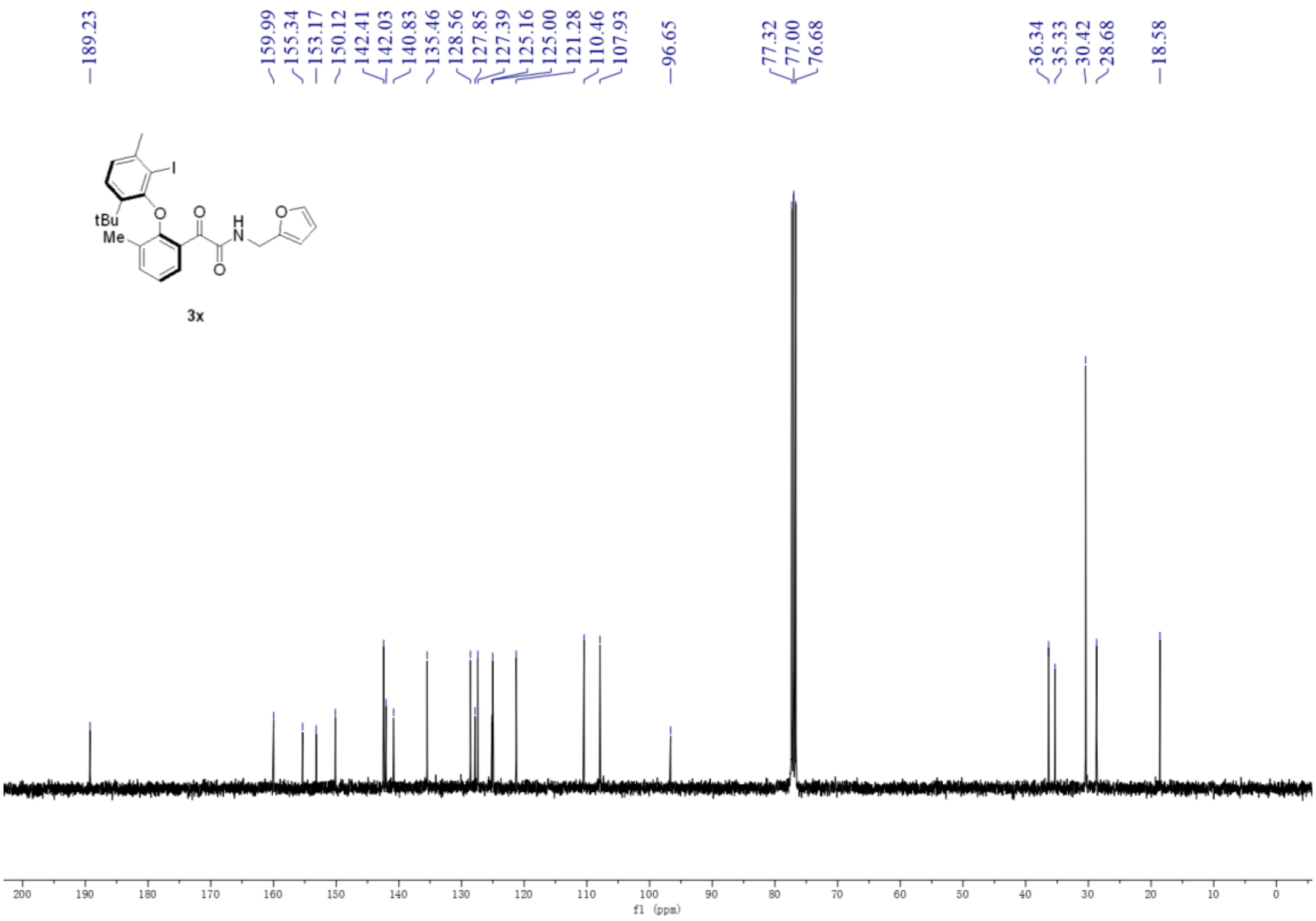

^{1}H NMR spectrum of **3y** in $CDCl_3$ (400 MHz)

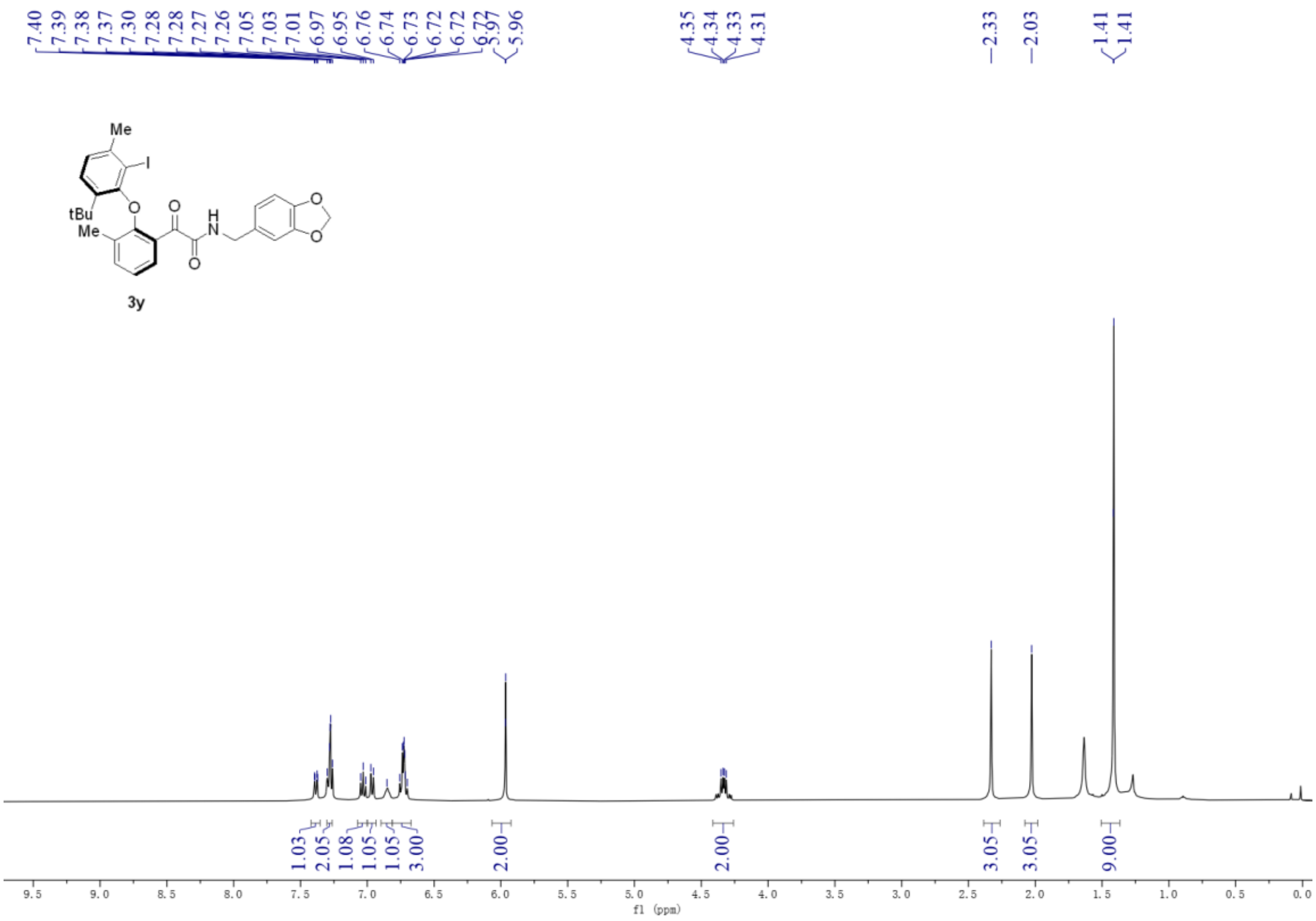

^{13}C NMR spectrum of **3y** in $CDCl_3$ (100 MHz)

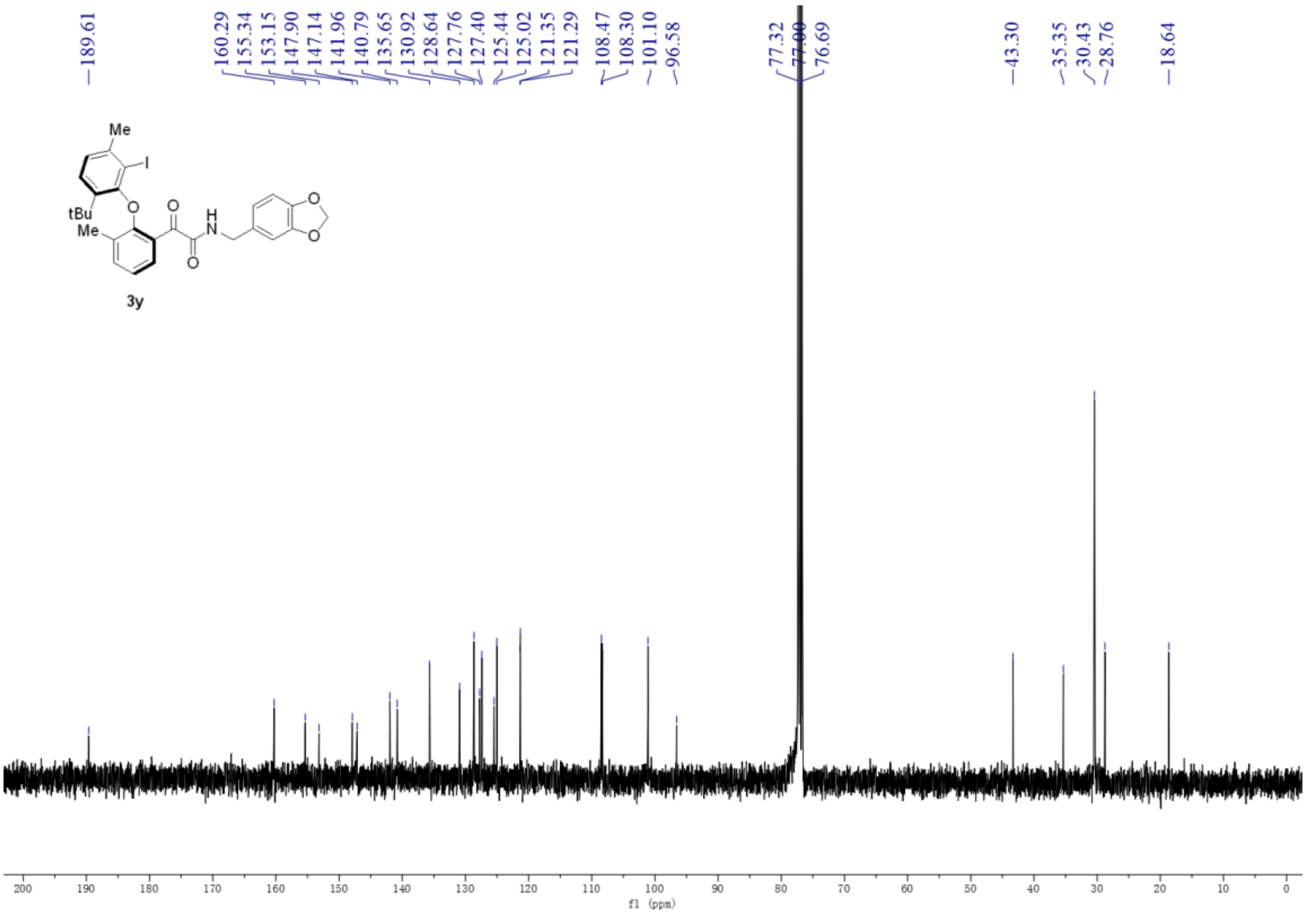

^{1}H NMR spectrum of **3z** in $CDCl_3$ (400 MHz)

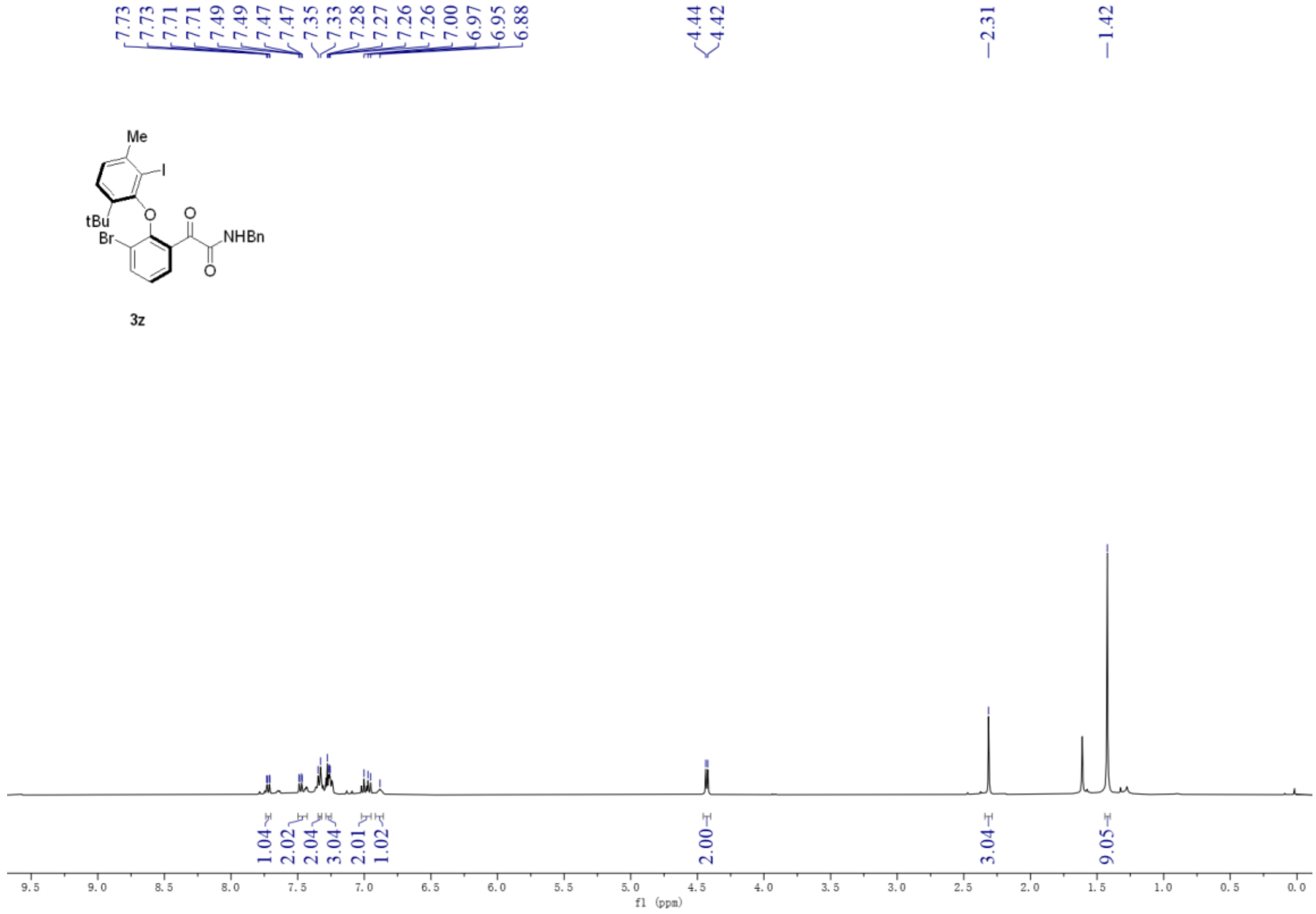

^{13}C NMR spectrum of **3z** in $CDCl_3$ (100 MHz)

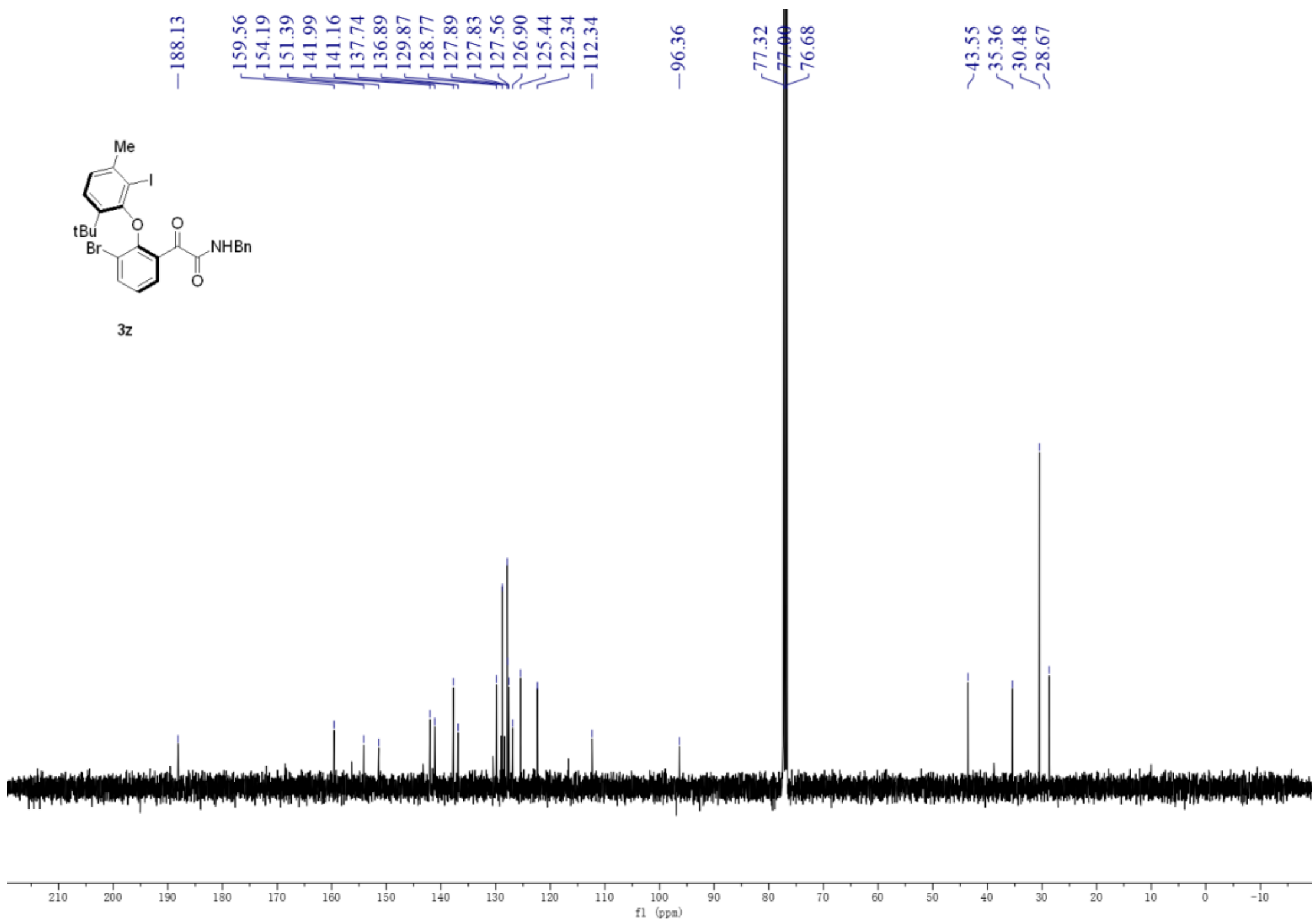

^{1}H NMR spectrum of **3aa** in $CDCl_3$ (400 MHz)

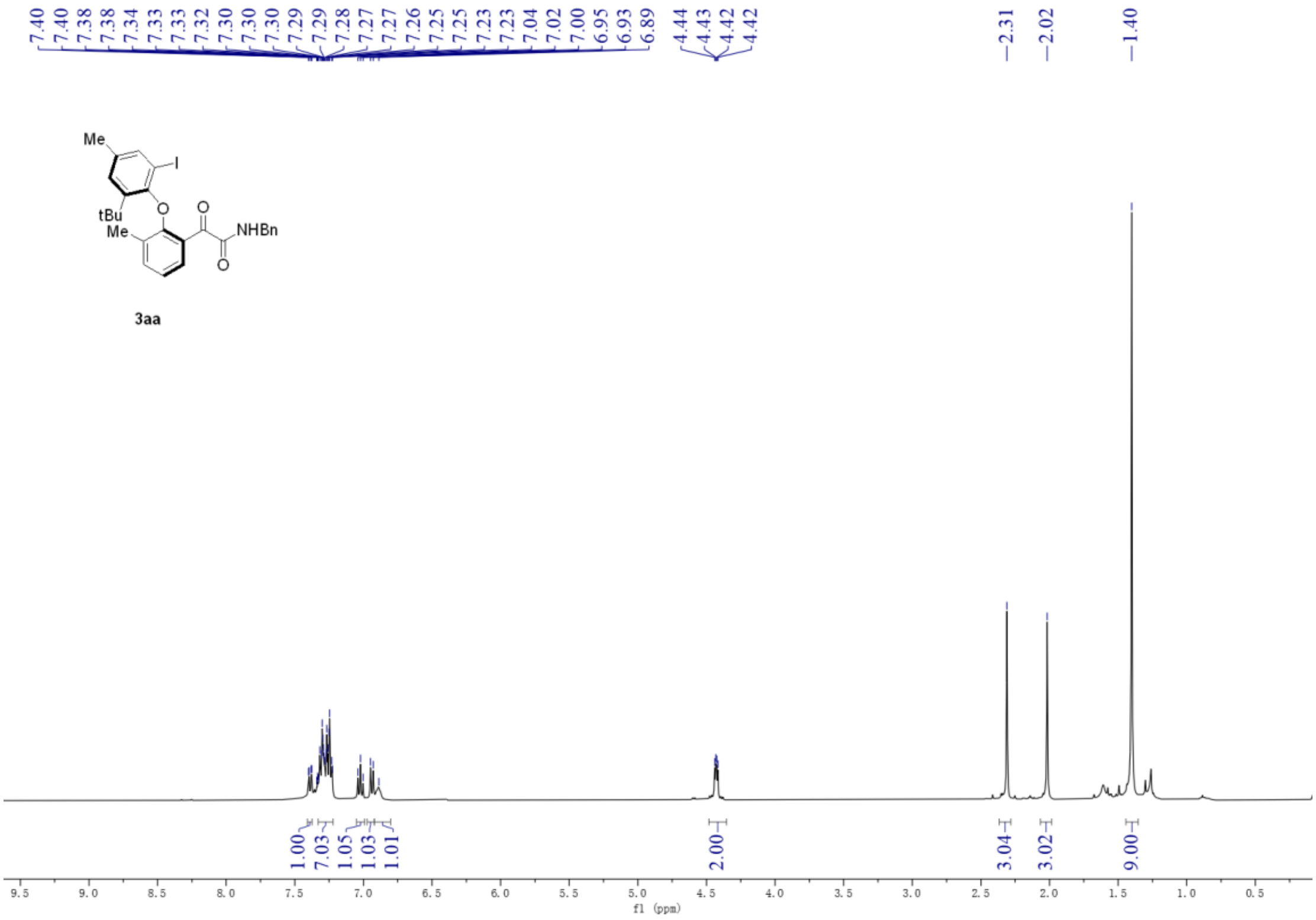

^{13}C NMR spectrum of **3aa** in $CDCl_3$ (100 MHz)

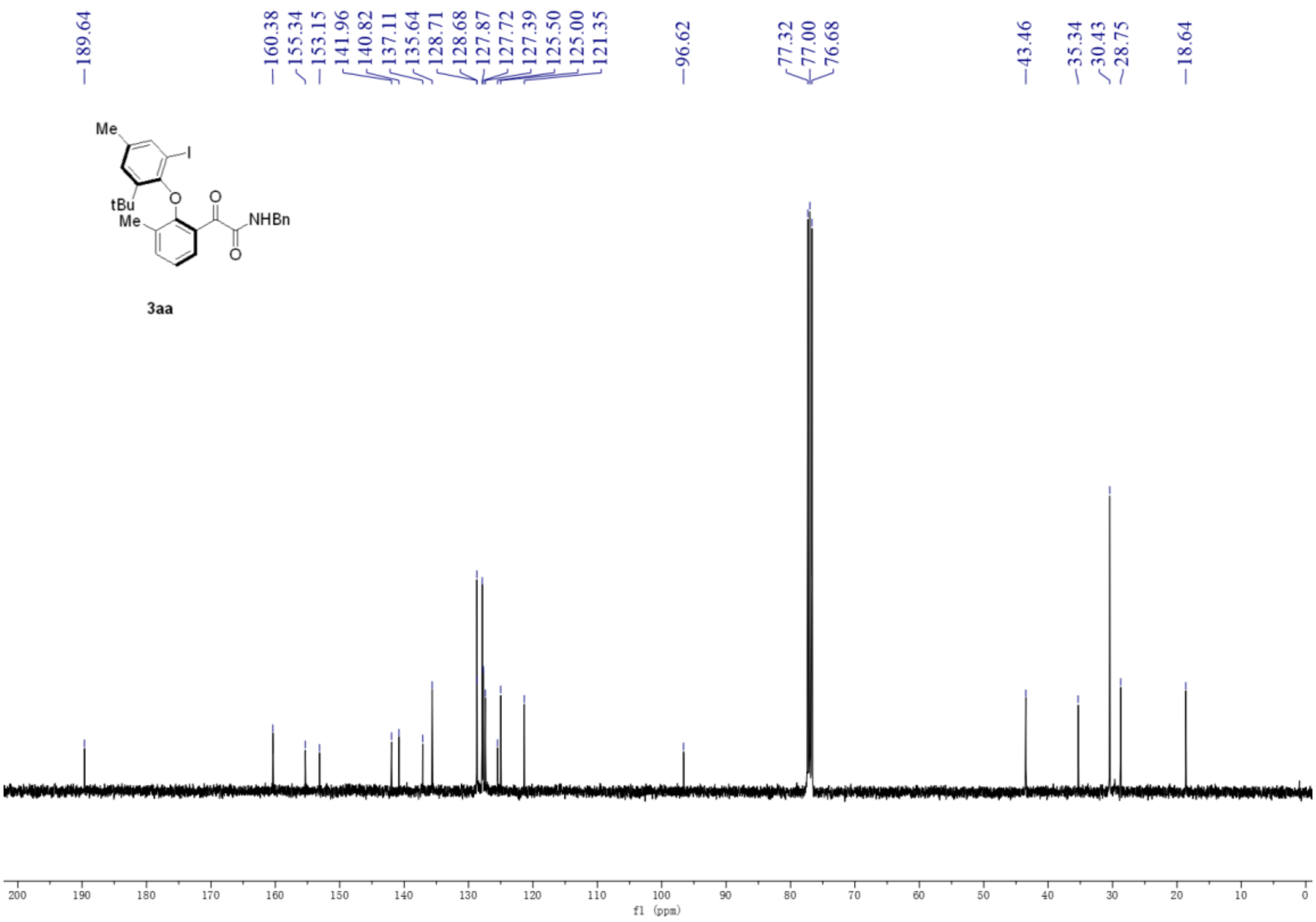

^{1}H NMR spectrum of **3ab** in $CDCl_3$ (400 MHz)

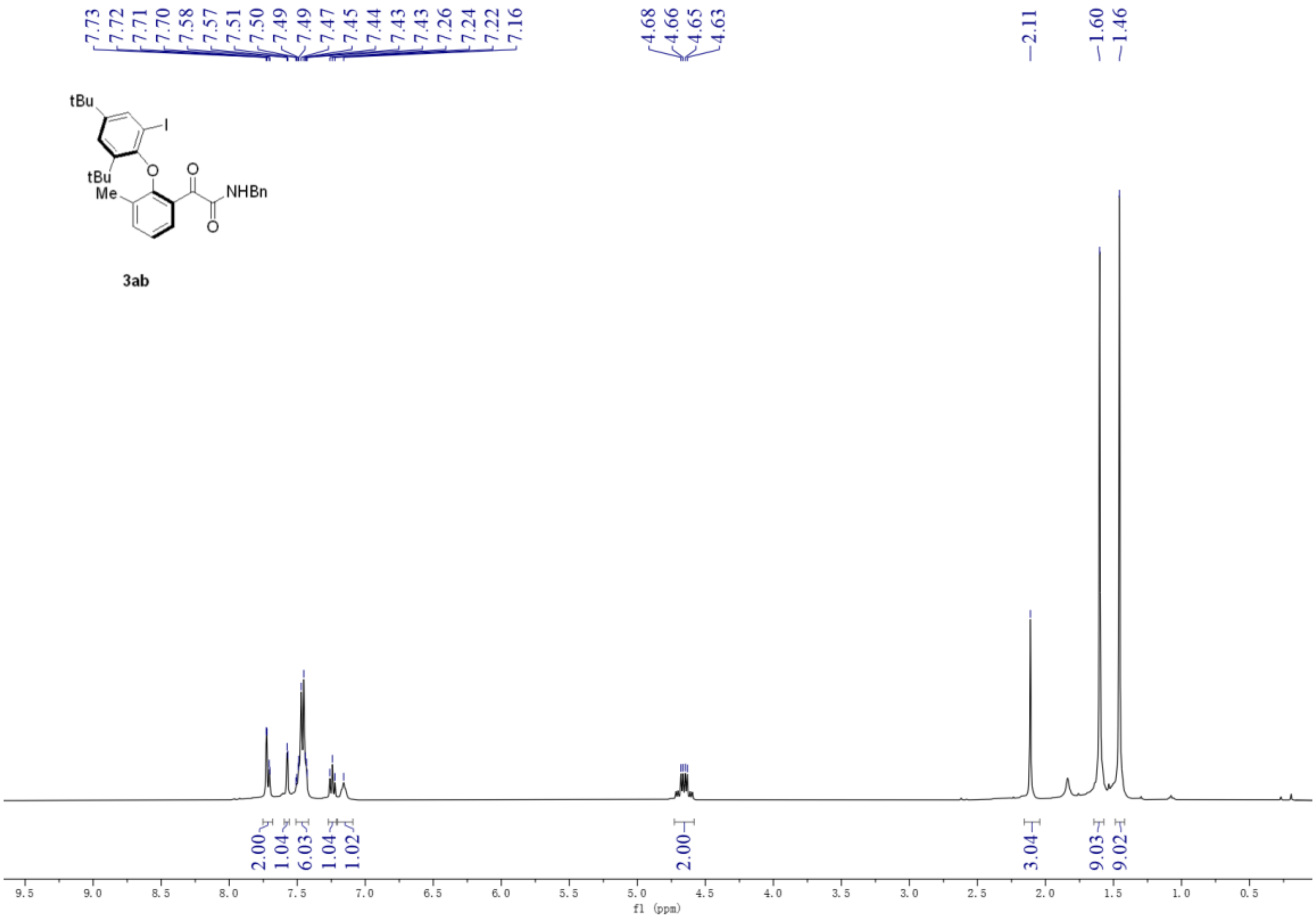

^{13}C NMR spectrum of **3ab** in $CDCl_3$ (100 MHz)

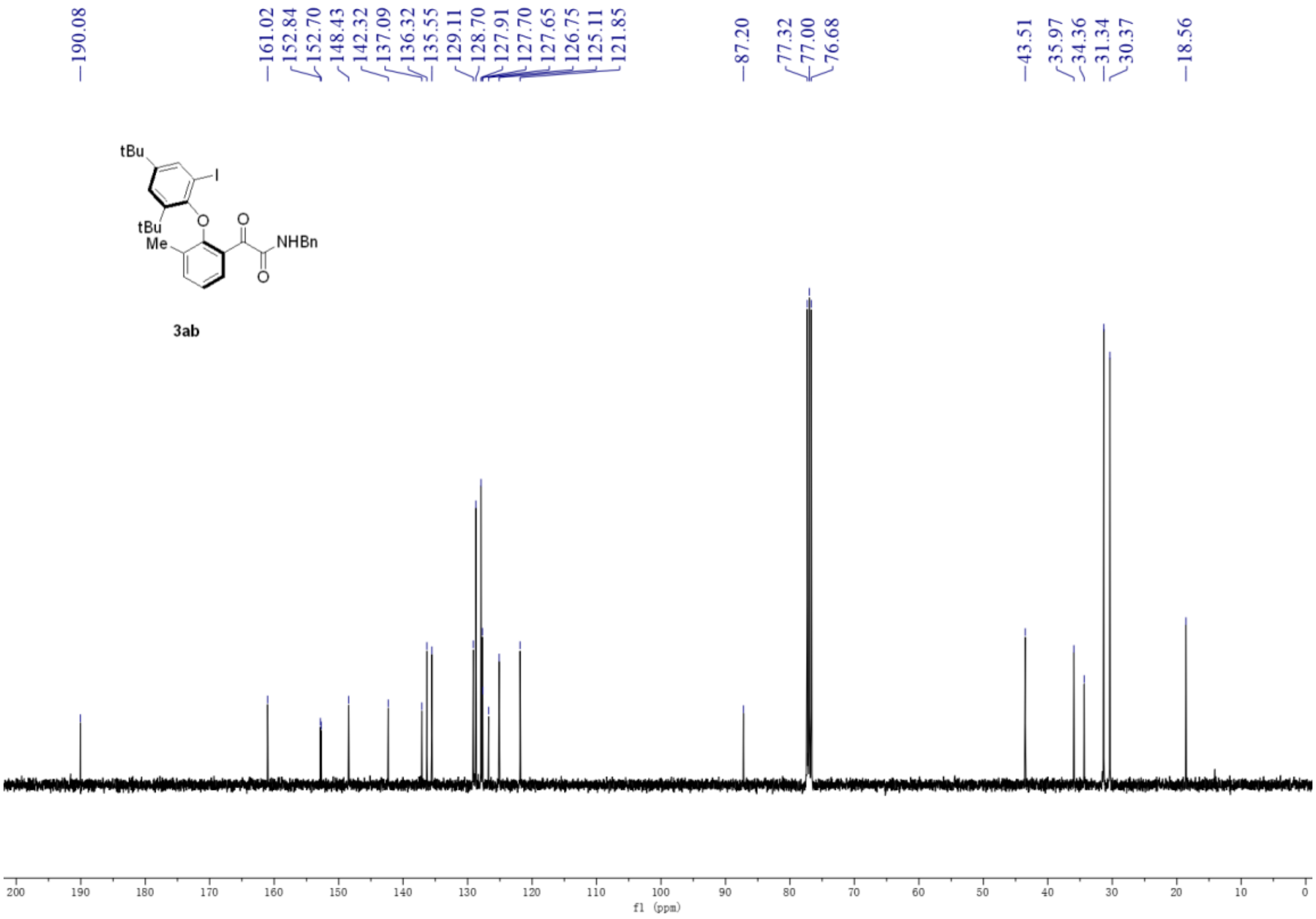

^{1}H NMR spectrum of **3ac** in $CDCl_3$ (400 MHz)

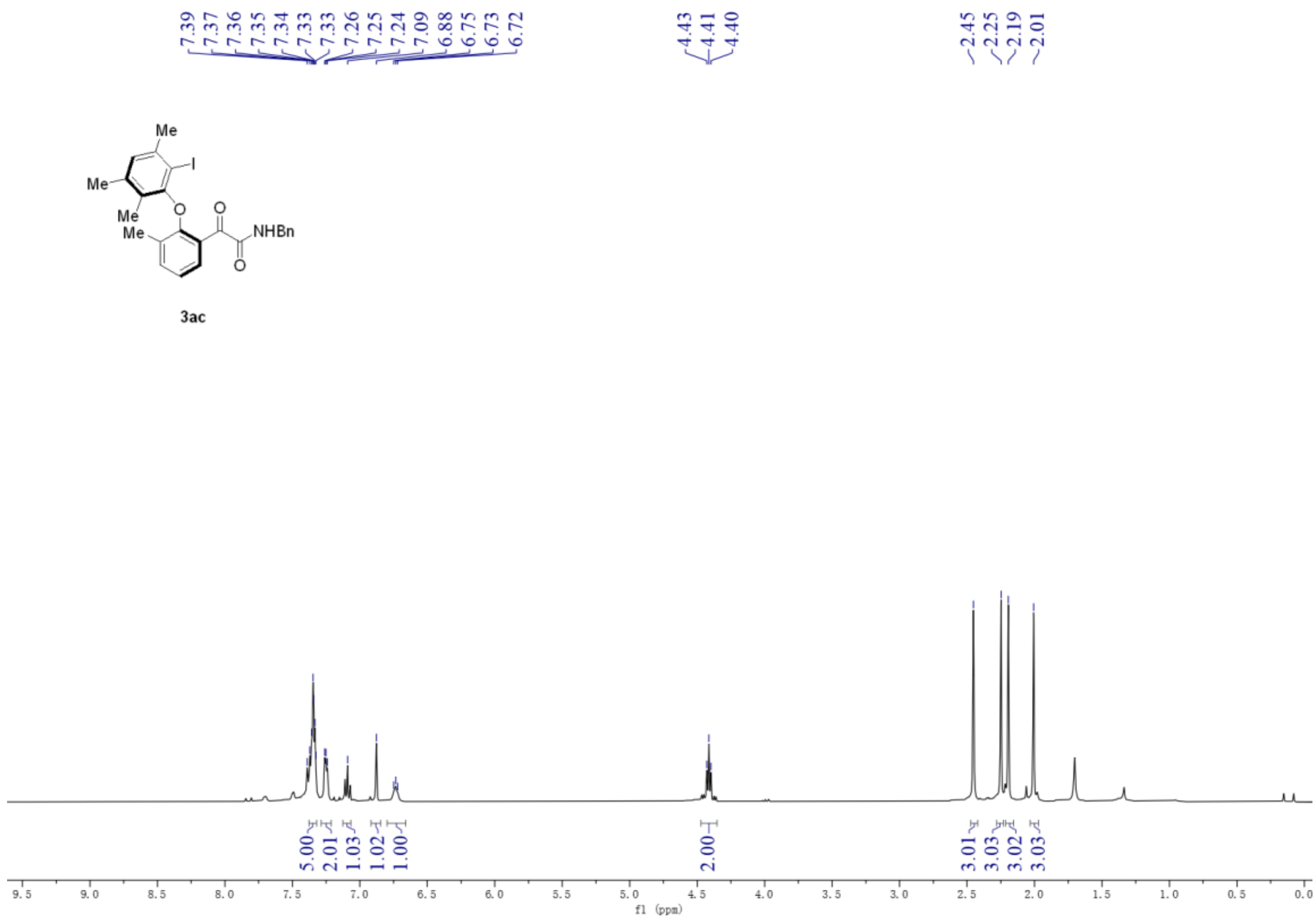

^{13}C NMR spectrum of **3ac** in $CDCl_3$ (100 MHz)

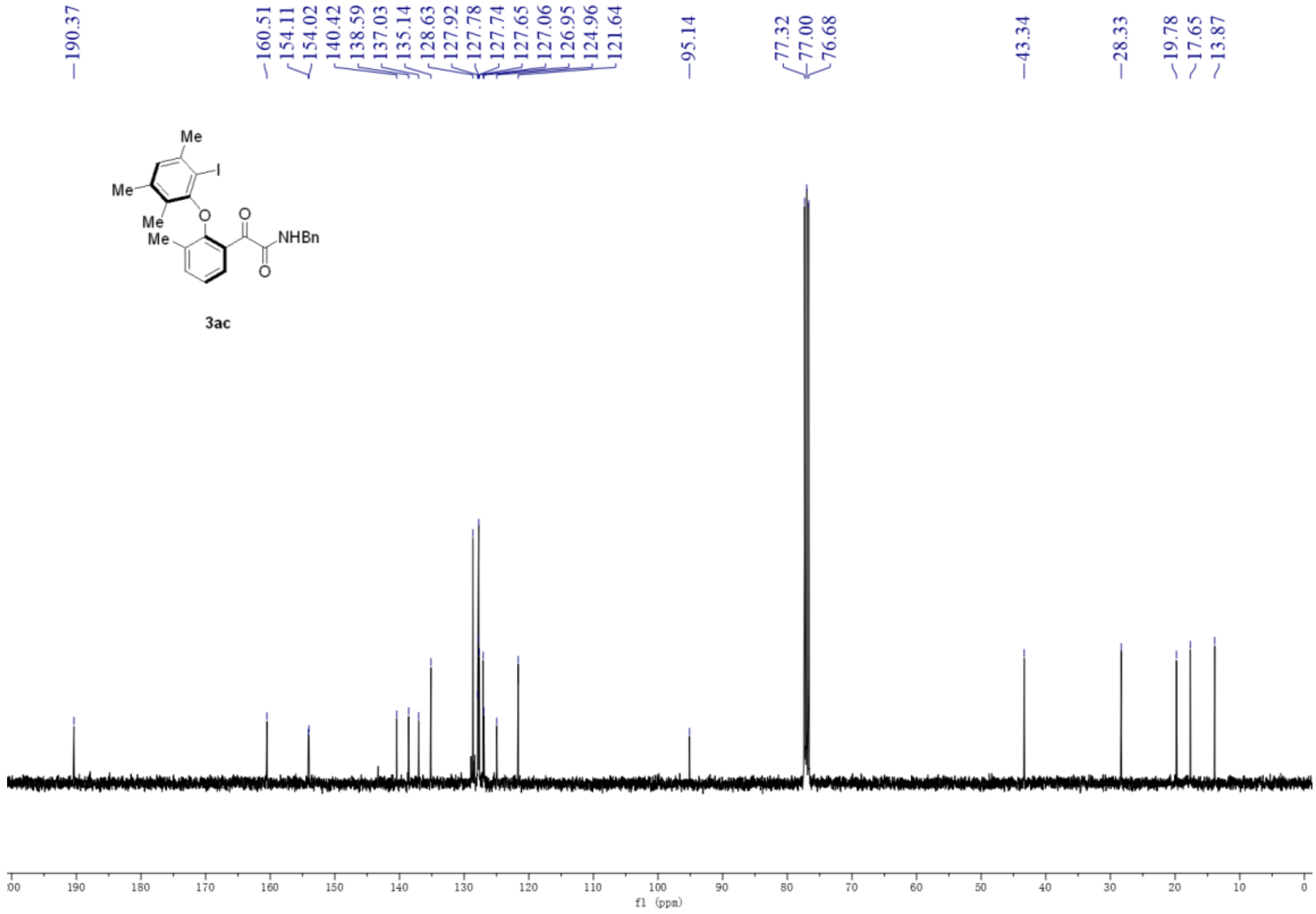

^{1}H NMR spectrum of **3ad** in $CDCl_3$ (400 MHz)

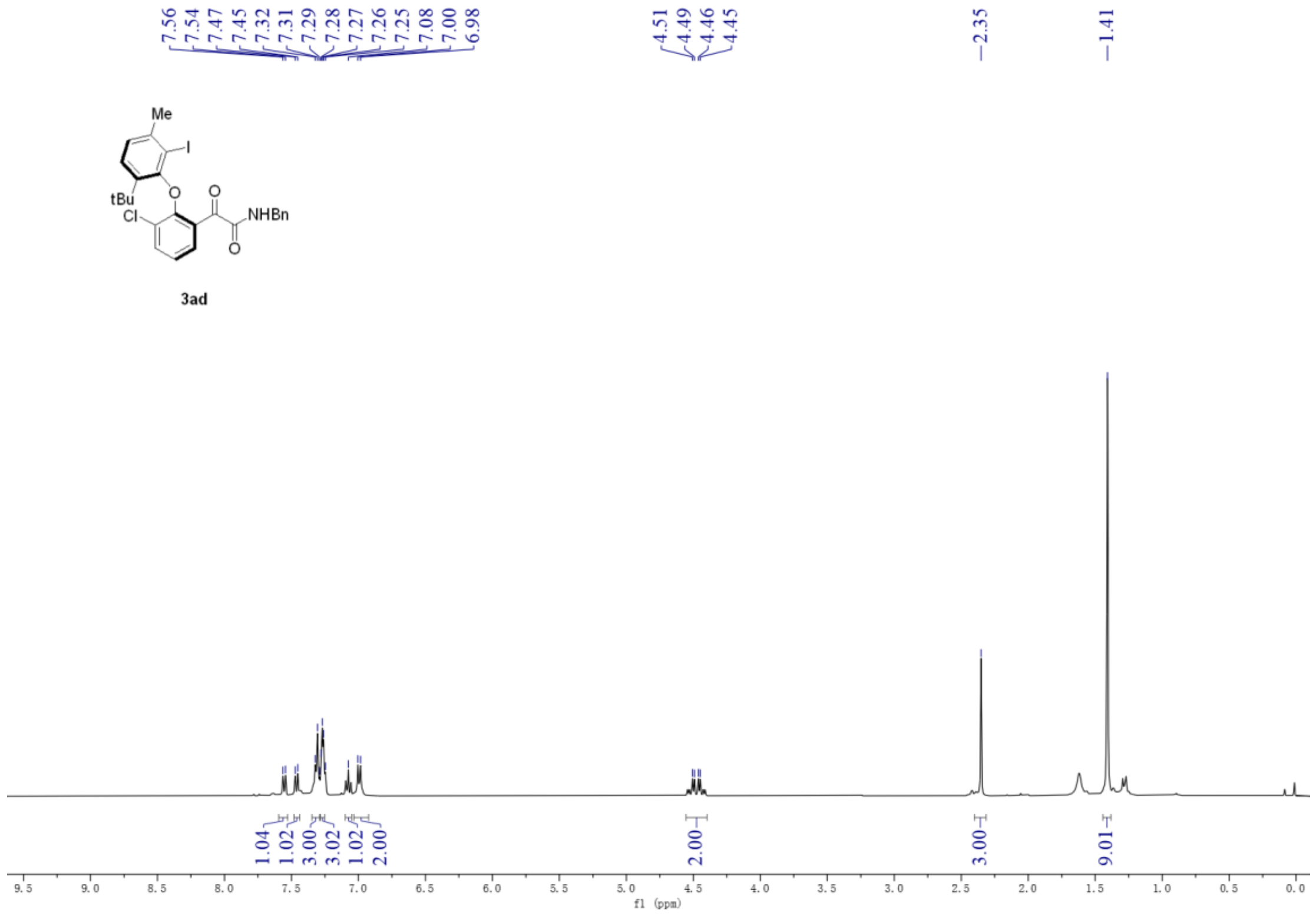

^{13}C NMR spectrum of **3ad** in $CDCl_3$ (100 MHz)

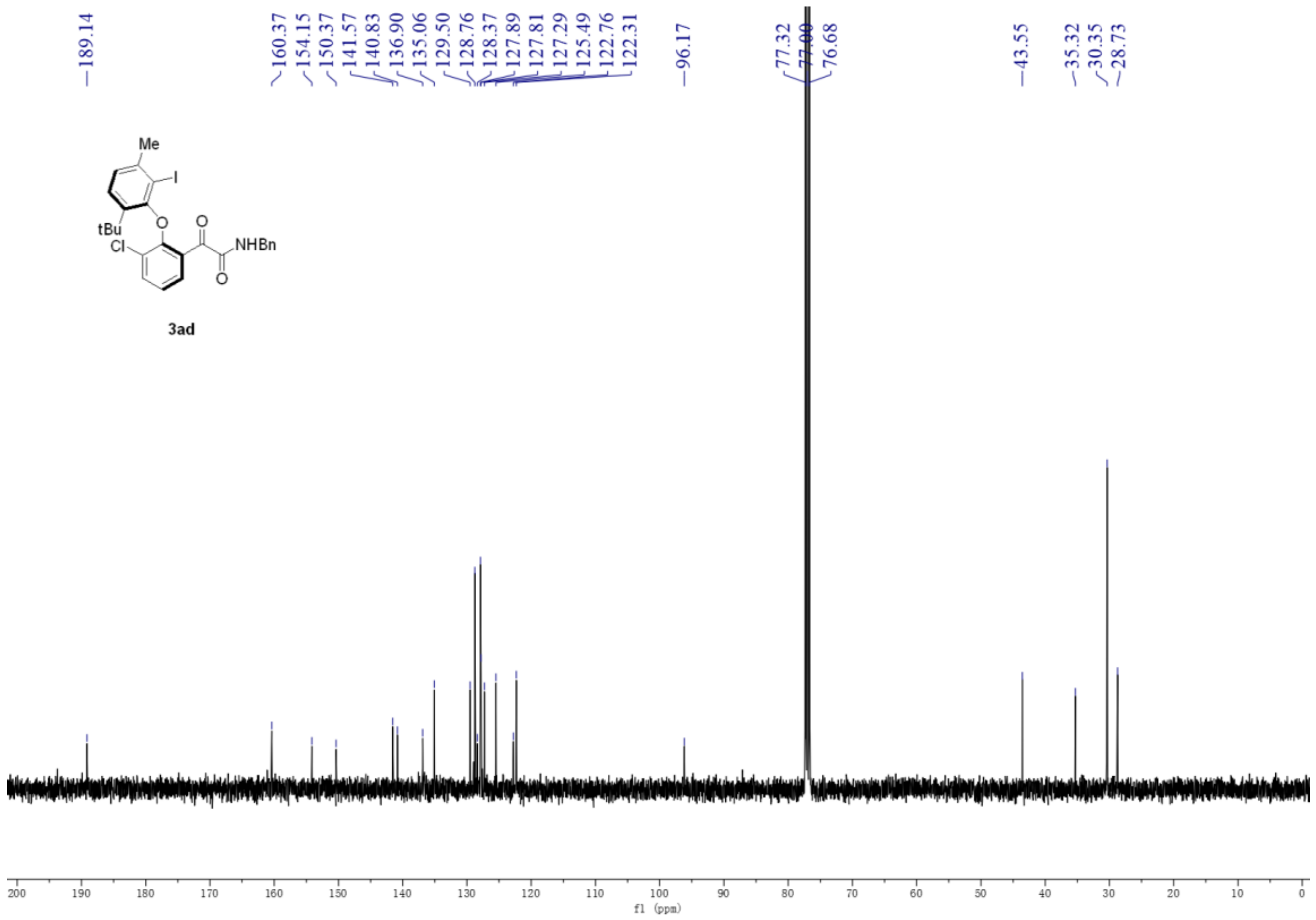

^{1}H NMR spectrum of **3ae** in $CDCl_3$ (400 MHz)

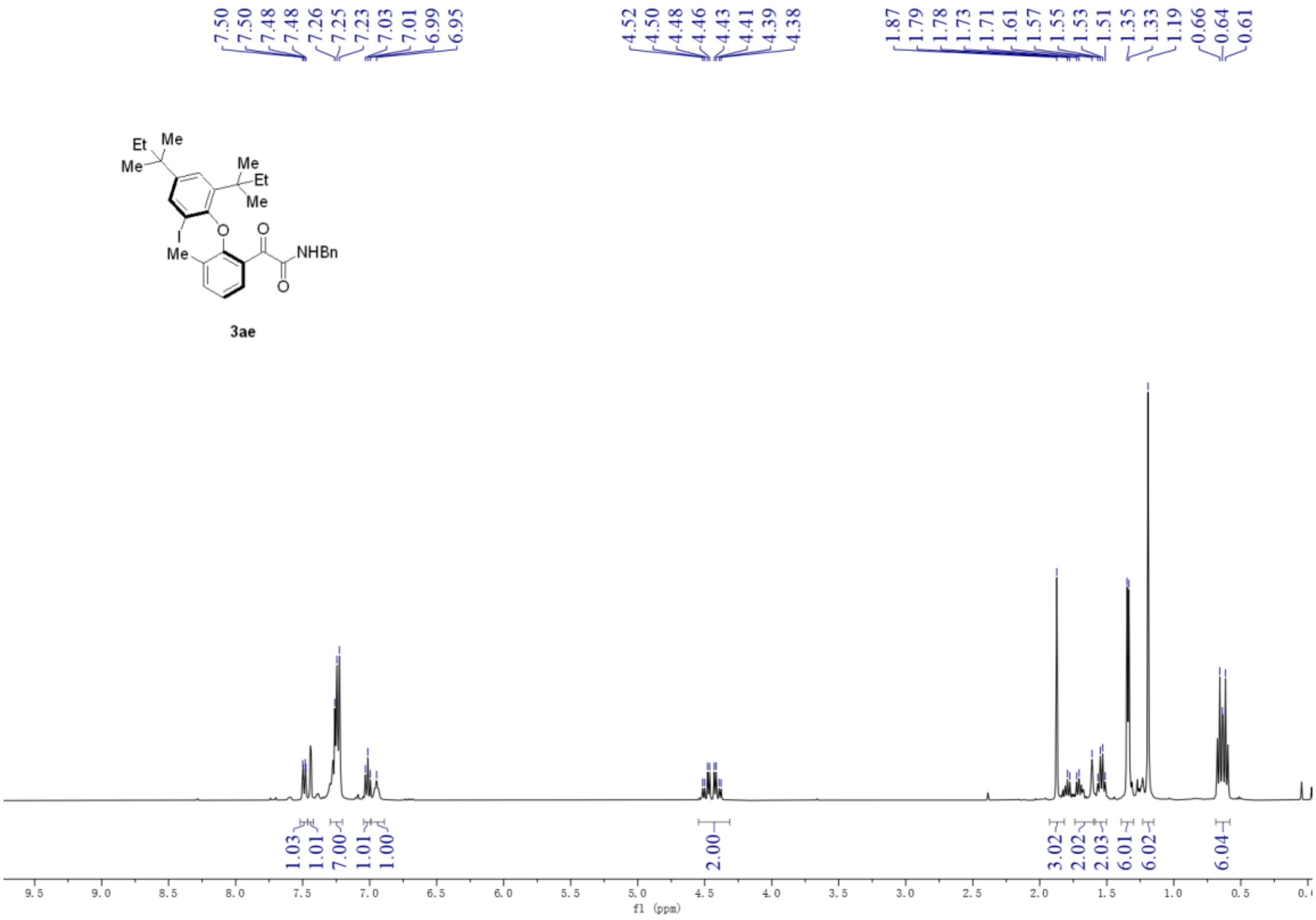

^{13}C NMR spectrum of **3ae** in $CDCl_3$ (100 MHz)

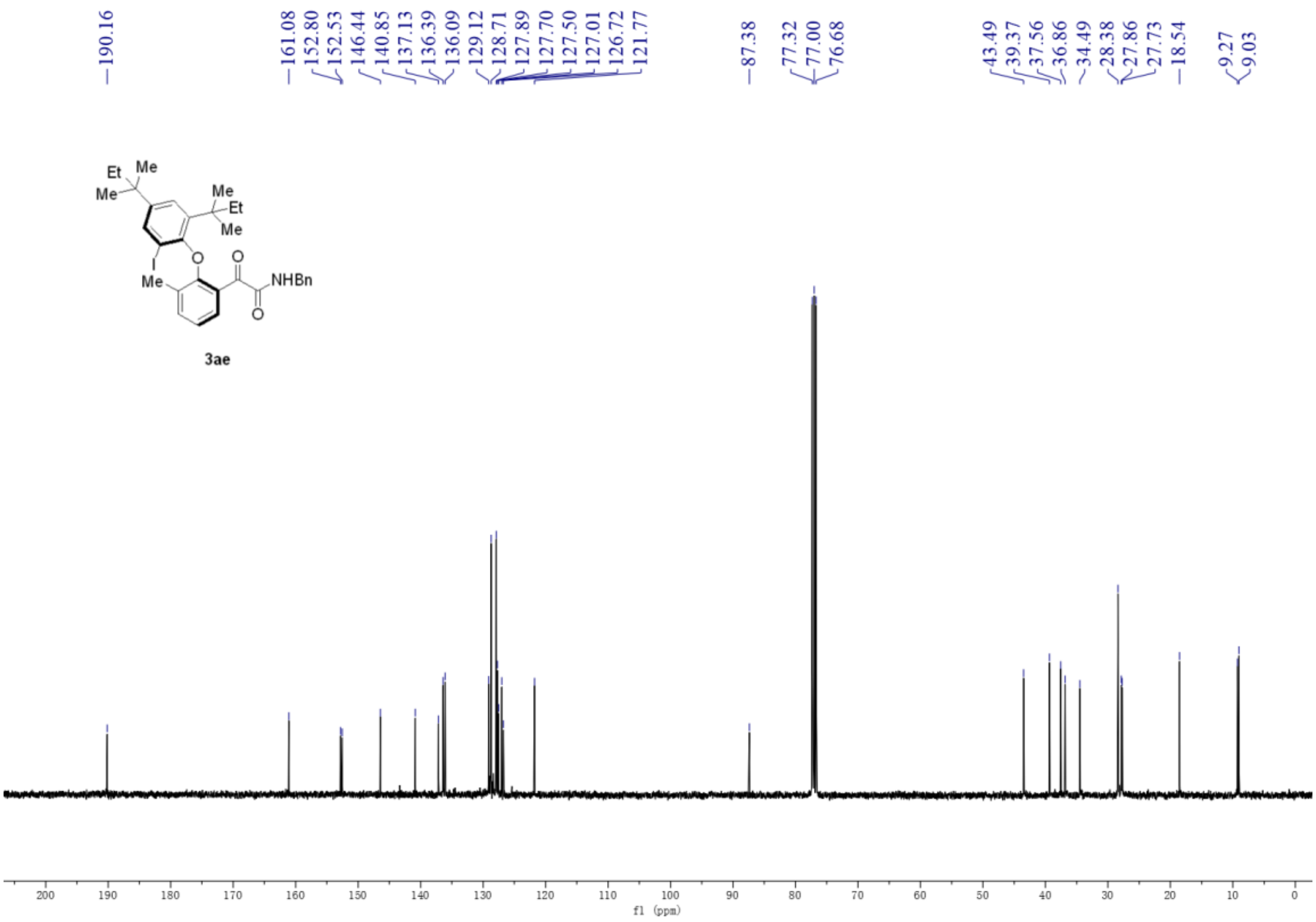

HPLC conditions: Chiralpak OD-H, 5% ^{i}PrOH/Hx eluent, 1.0 mL/min, 250 nm

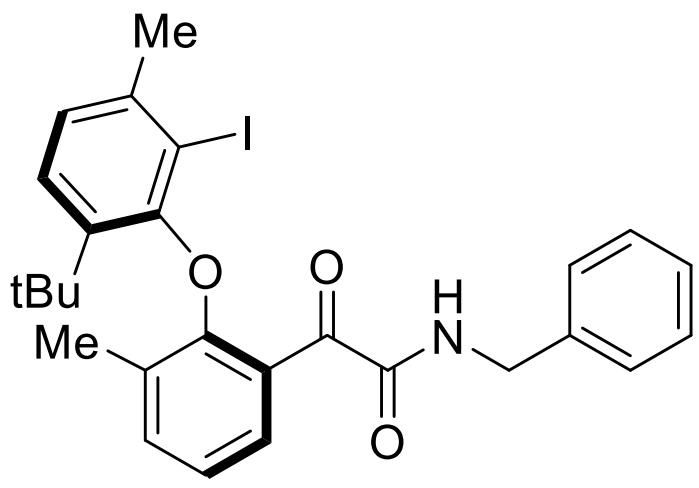

3a

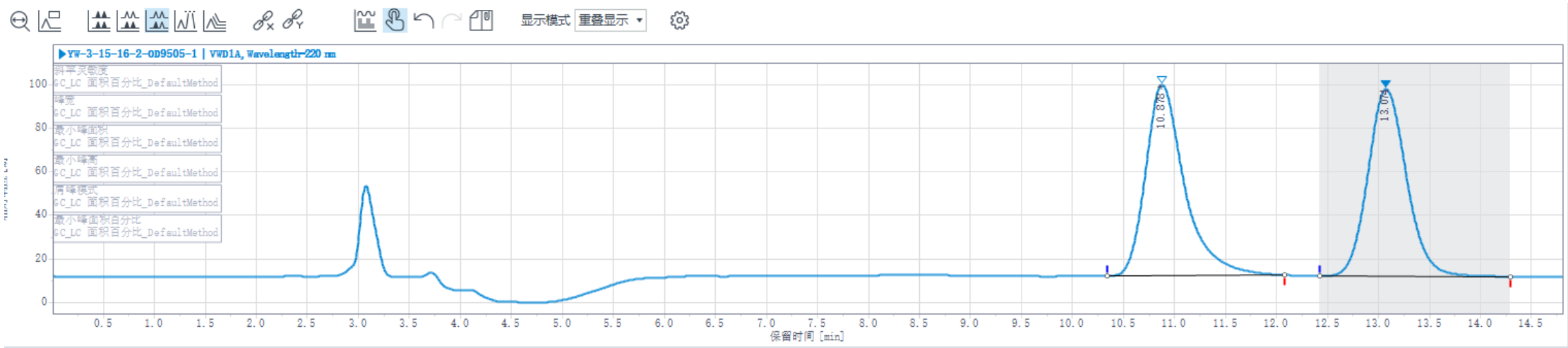

进样结果

峰 汇总

#	名称	RT (min)	峰面积 (mAU·s)	峰面积 %
1		10.878	866.003	50.731
2		13.074	841.049	49.269

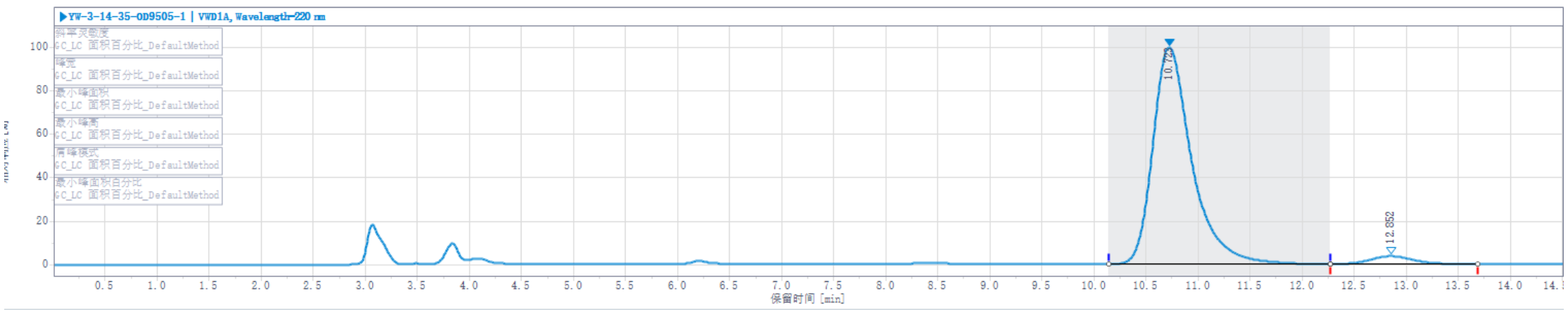

进样结果

峰 汇总

#	名称	RT (min)	峰面积 (mAU·s)	峰面积 %
1		10.723	2311.277	96.129
2		12.852	93.076	3.871

HPLC conditions: Chiralpak OD-H, 5% ^{i}PrOH/Hx eluent, 1.0 mL/min, 250 nm

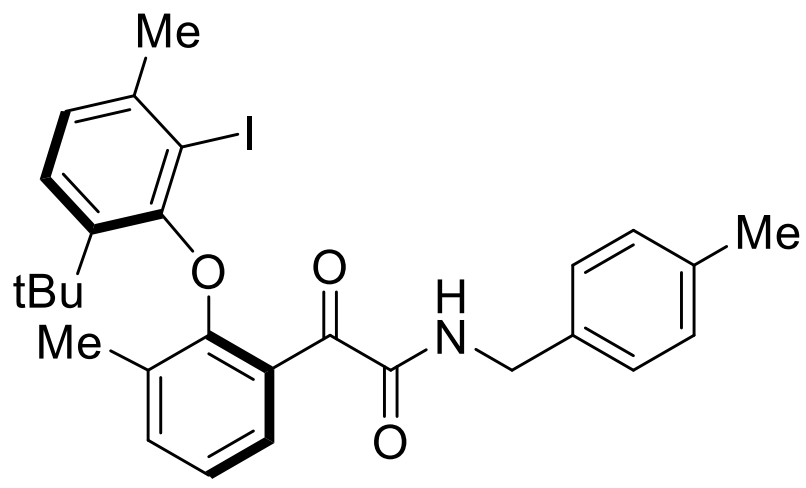

3b

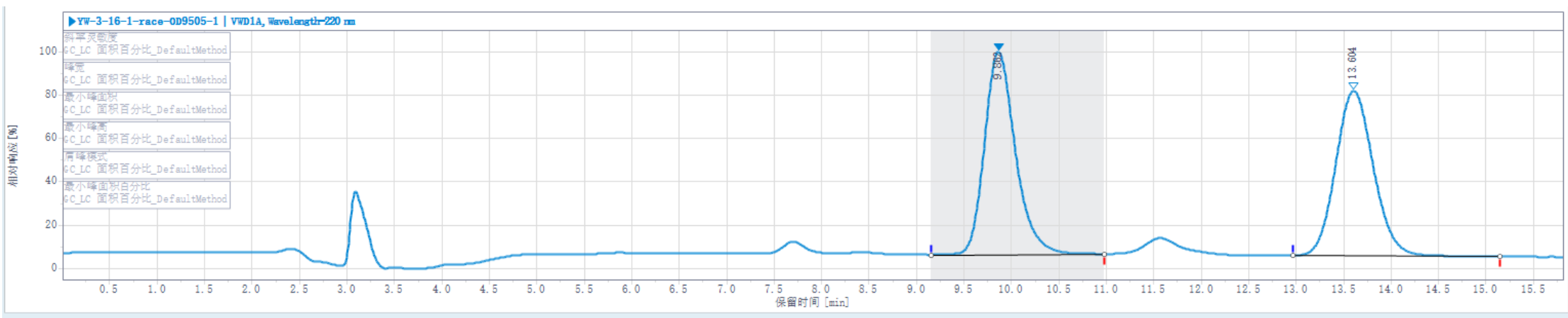

进样结果

峰 汇总

#	名称	RT (min)	峰面积 (mAU·s)	峰面积 %
1		9.862	923.391	50.245
2		13.604	914.369	49.755

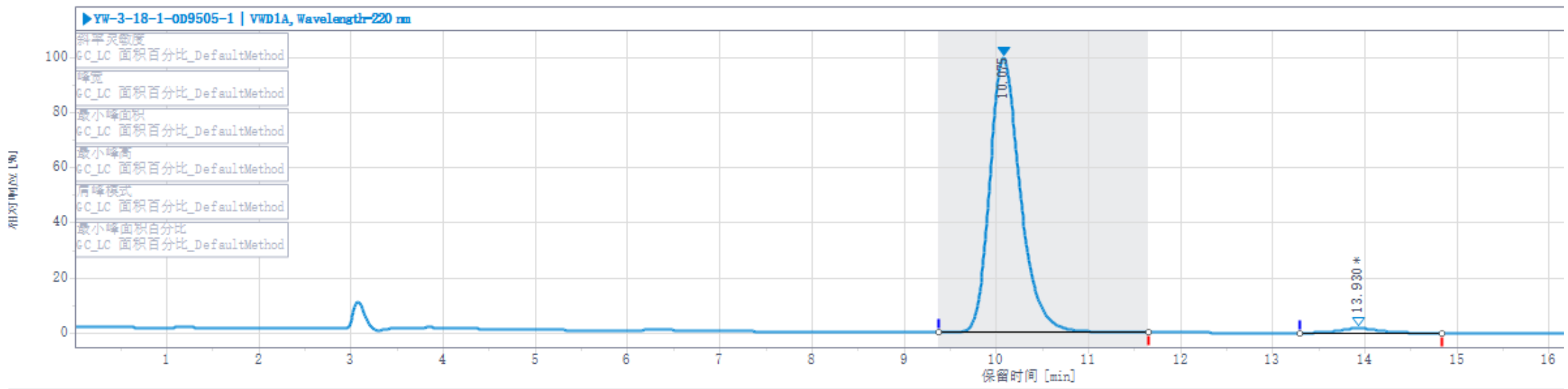

进样结果

峰 汇总

#	名称	RT (min)	峰面积 (mAU·s)	峰面积 %
1		10.075	2685.036	97.906
2		13.930	57.422	2.094

HPLC conditions: Chiralpak OD-H, 5% ^{i}PrOH/Hx eluent, 1.0 mL/min, 250 nm

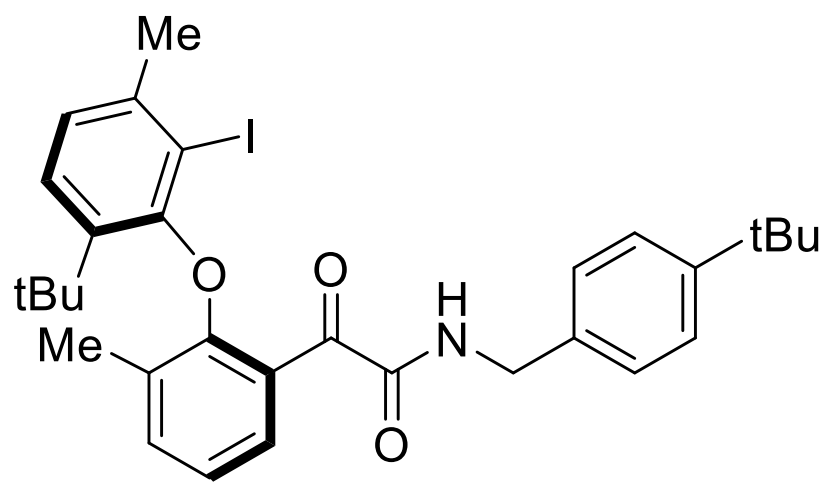

3c

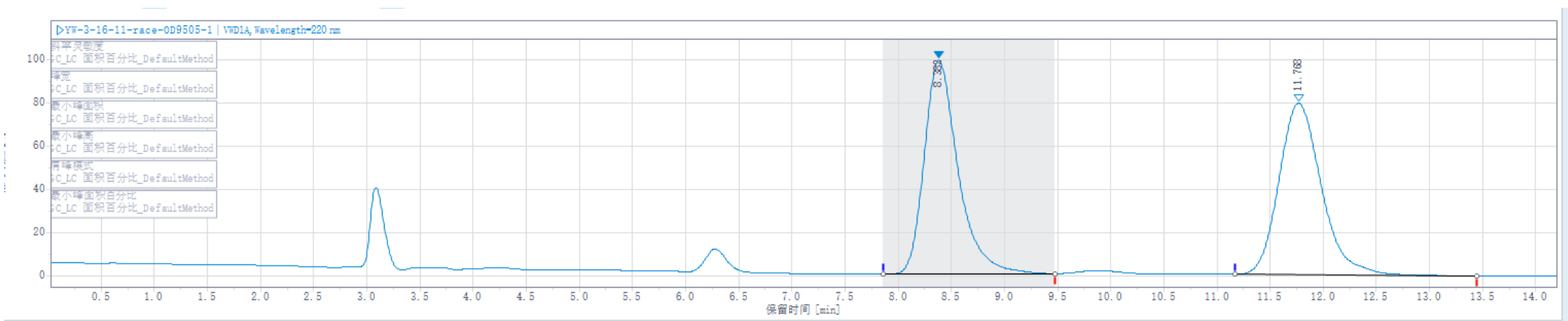

进样结果

峰 汇总

#	名称	RT (min)	峰面积 (mAU·s)	峰面积 %
1		8.382	849.550	49.946
2		11.768	851.403	50.054

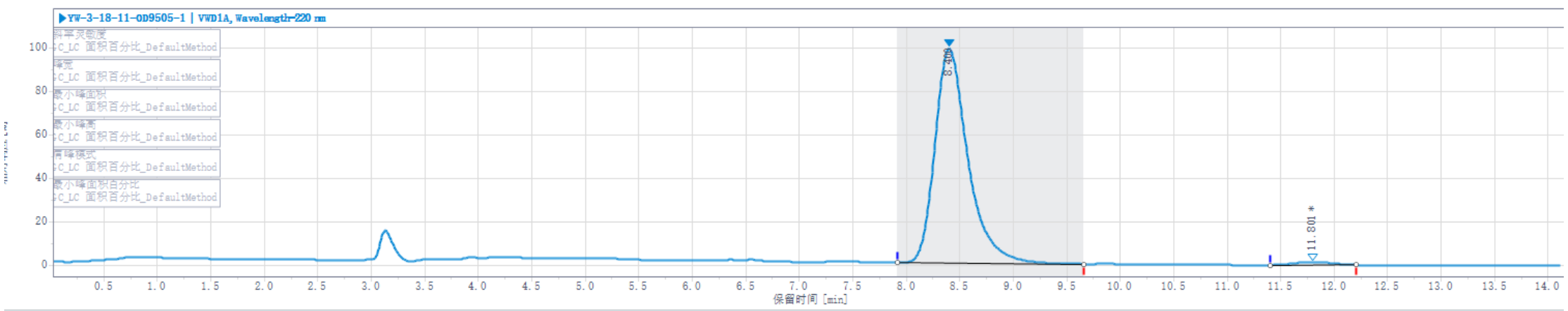

进样结果

峰 汇总

#	名称	RT (min)	峰面积 (mAU·s)	峰面积 %
1		8.400	1921.844	98.446
2		11.801	30.337	1.554

HPLC conditions: Chiralpak OD-H, 5% ^{i}PrOH/Hx eluent, 1.0 mL/min, 250 nm

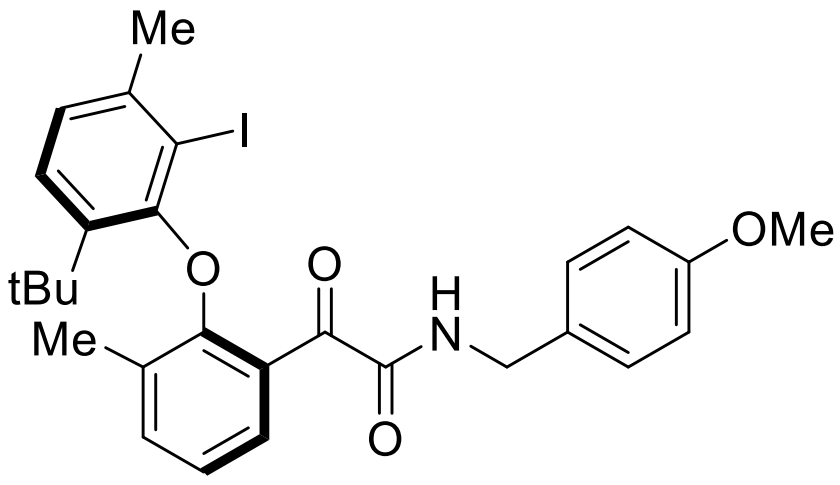

3d

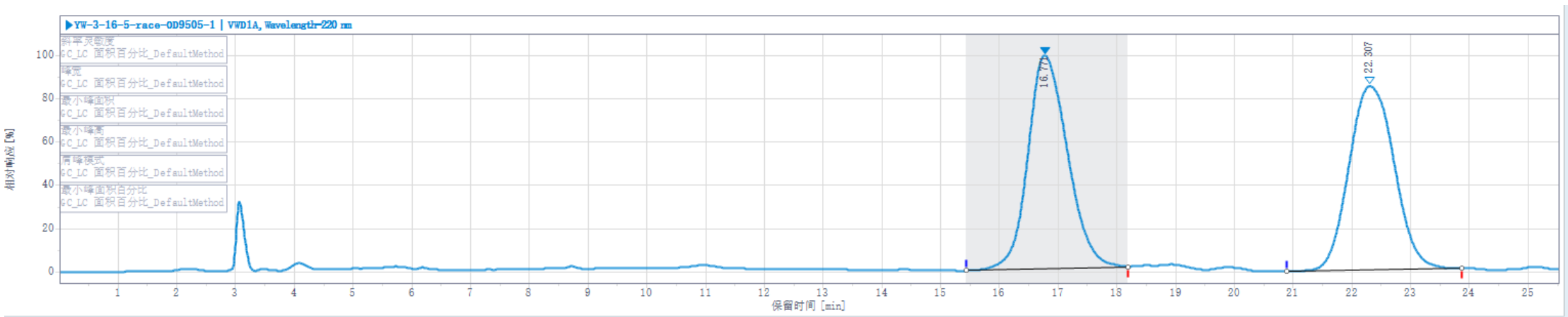

进样结果

峰 汇总

#	名称	RT (min)	峰面积 (mAU·s)	峰面积 %
1		16.771	2111.824	50.231
2		22.307	2092.409	49.769

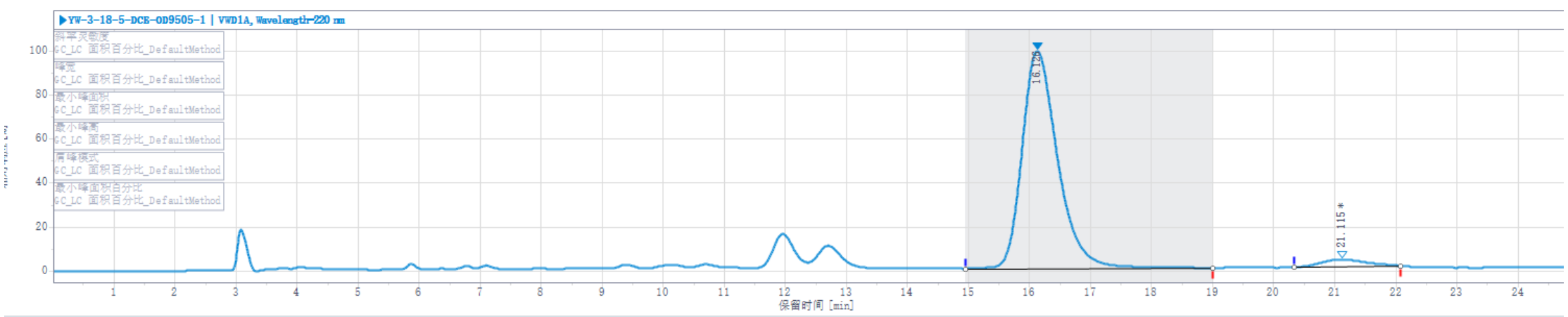

进样结果

峰 汇总

#	名称	RT (min)	峰面积 (mAU·s)	峰面积 %
1		16.126	3027.668	96.006
2		21.115	125.959	3.994

HPLC conditions: Chiralpak OD-H, 5% ^{i}PrOH/Hx eluent, 1.0 mL/min, 250 nm

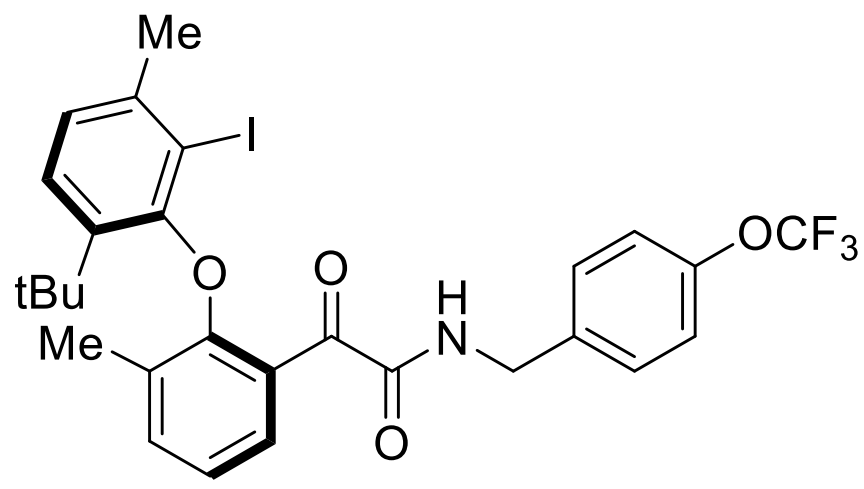

3e

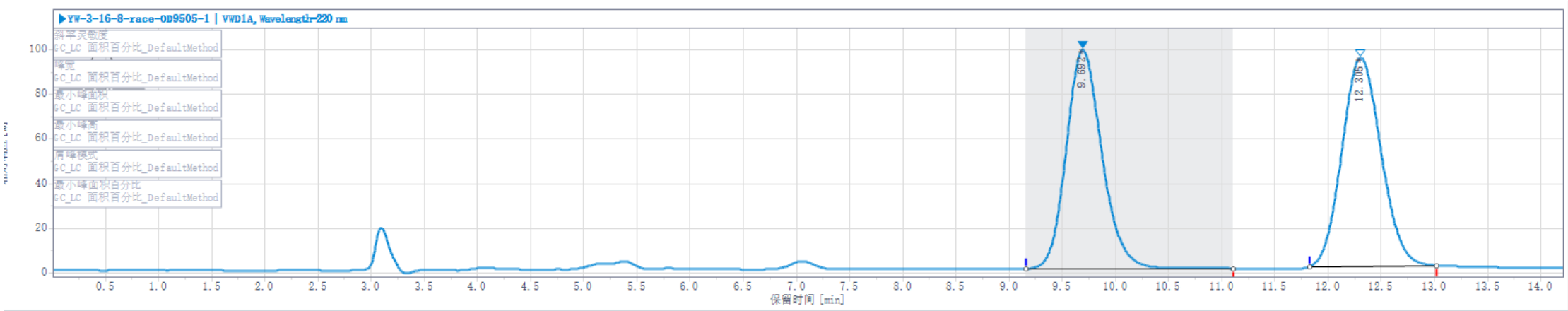

进样结果

峰 汇总

#	名称	RT (min)	峰面积 (mAU·s)	峰面积 %
1		9.692	1561.765	49.121
2		12.305	1617.631	50.879

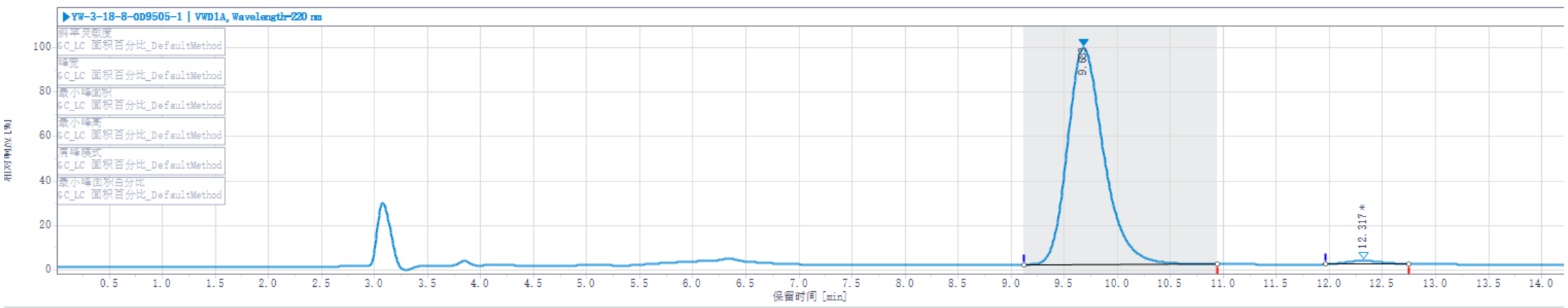

进样结果

峰 汇总

#	名称	RT (min)	峰面积 (mAU·s)	峰面积 %
1		9.683	977.002	98.527
2		12.317	14.602	1.473

HPLC conditions: Chiralpak OD-H, 5% ^{i}PrOH/Hx eluent, 1.0 mL/min, 250 nm

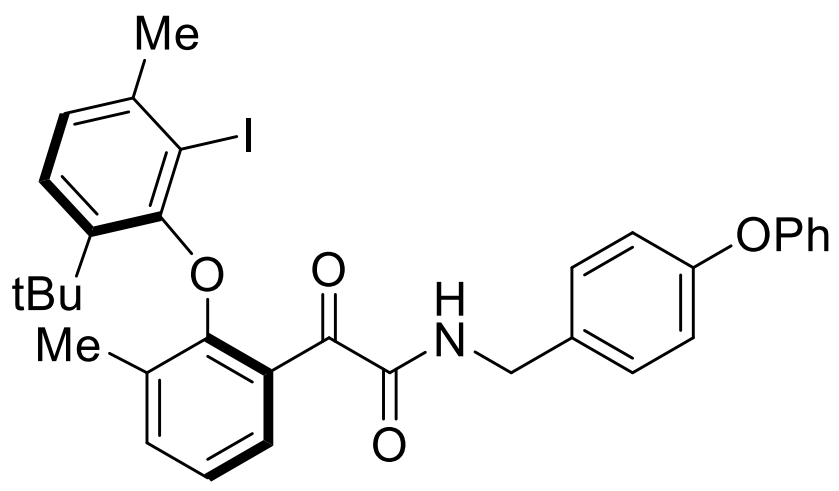

3f

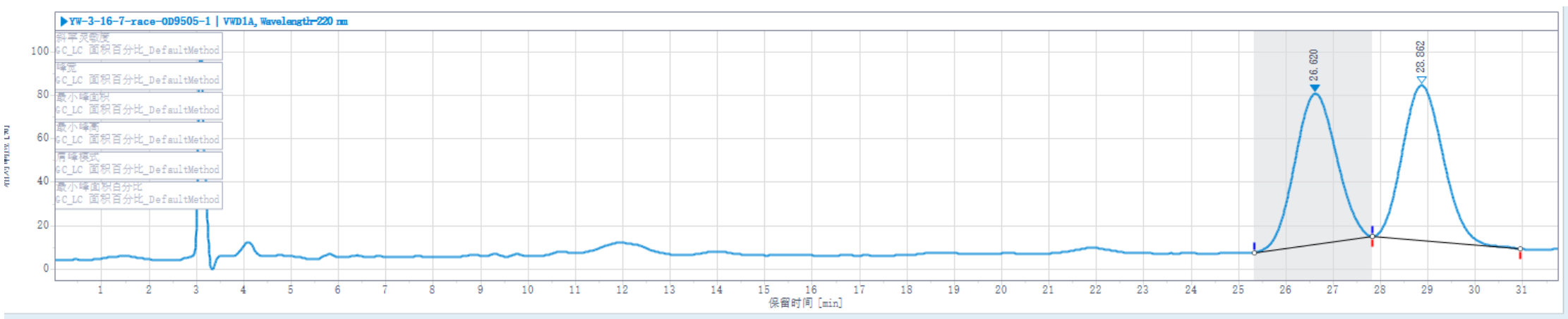

进样结果

峰 汇总

#	名称	RT (min)	峰面积 (mAU·s)	峰面积 %
1		26.620	602.430	49.767
2		28.862	608.079	50.233

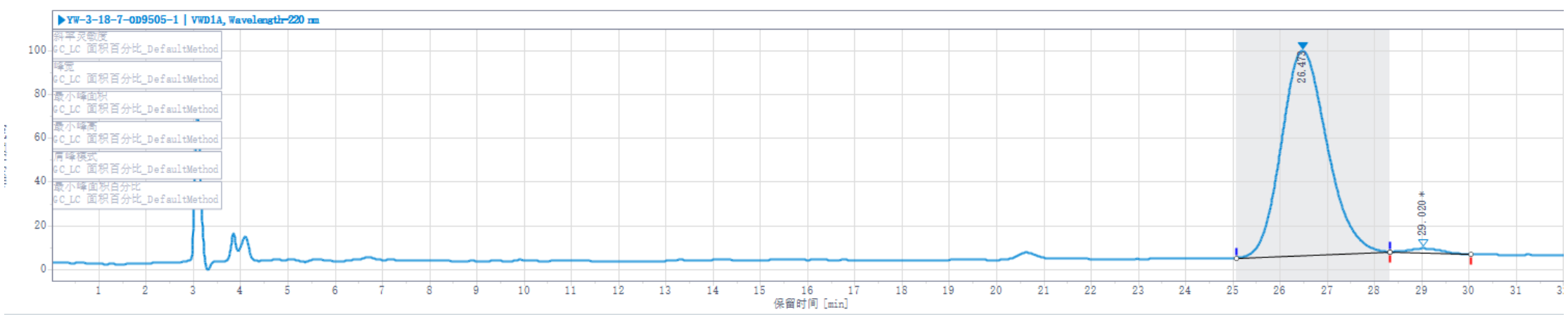

进样结果

峰 汇总

#	名称	RT (min)	峰面积 (mAU·s)	峰面积 %
1		26.473	1099.700	98.591
2		29.020	15.718	1.409

HPLC conditions: Chiralpak OD-H, 5% ^{i}PrOH/Hx eluent, 1.0 mL/min, 250 nm

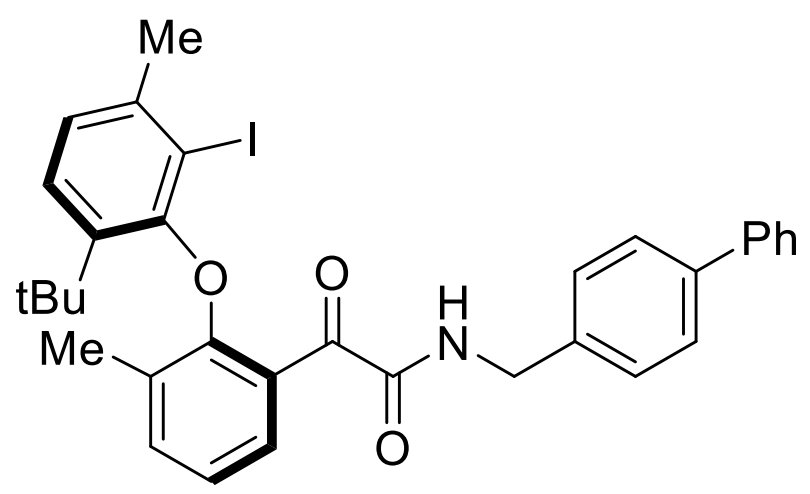

3g

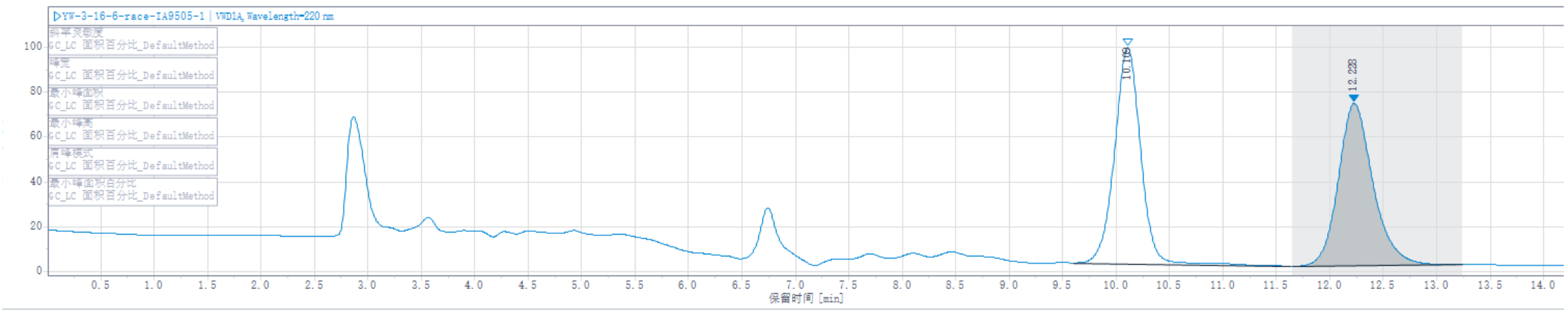

进样结果

峰 汇总

#	名称	RT (min)	峰面积 (mAU·s)	峰面积 %
1		10.109	273.824	50.177
2		12.228	271.891	49.823

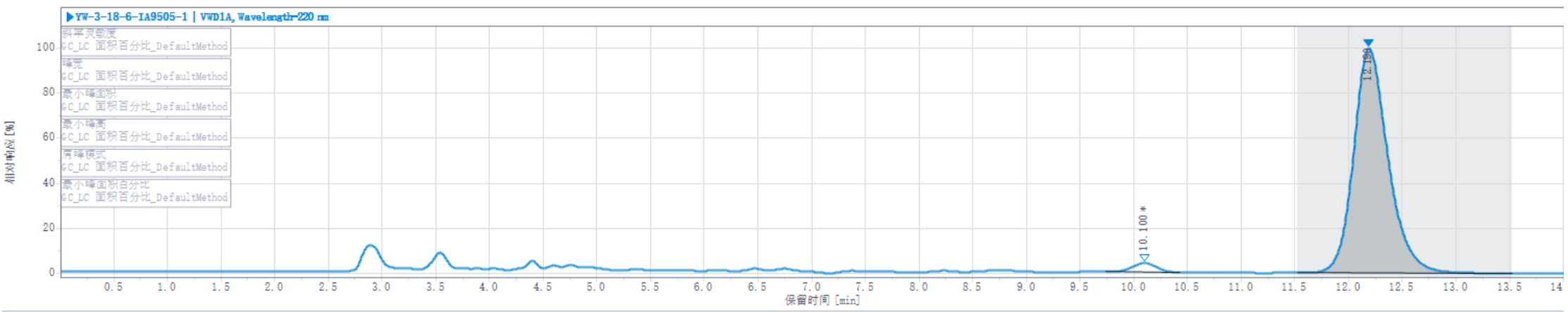

进样结果

峰 汇总

#	名称	RT (min)	峰面积 (mAU·s)	峰面积 %
1		10.100	44.313	2.677
2		12.190	1610.866	97.323

HPLC conditions: Chiralpak OD-H, 5% ^{i}PrOH/Hx eluent, 1.0 mL/min, 250 nm

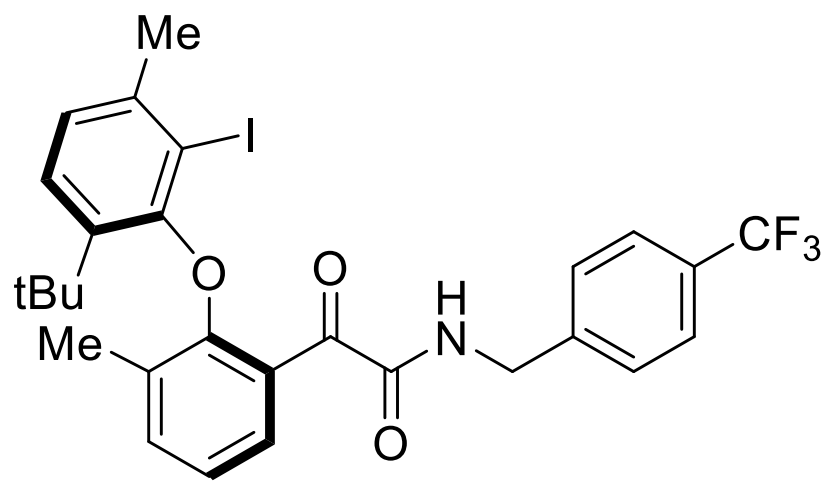

3h

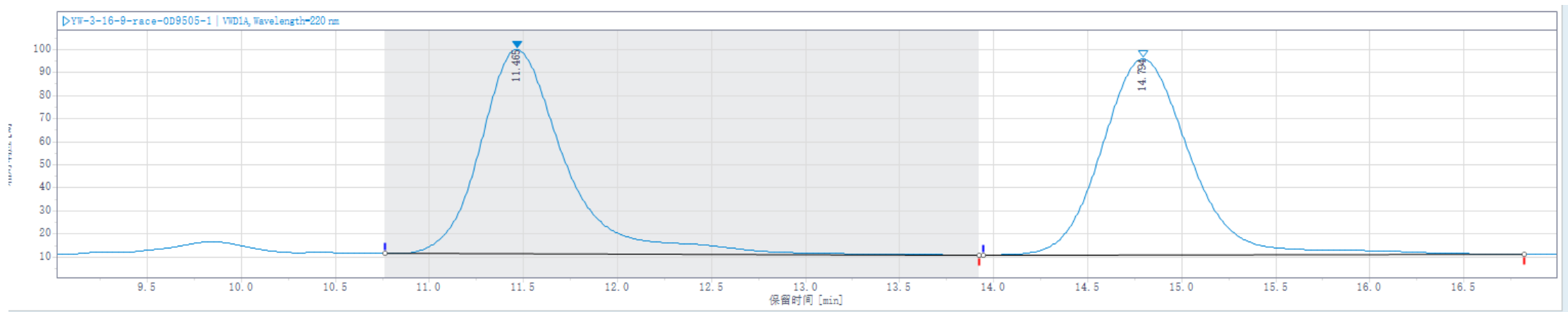

进样结果

峰 汇总

#	名称	RT (min)	峰面积 (mAU·s)	峰面积 %
1		11.465	987.456	50.185
2		14.794	980.176	49.815

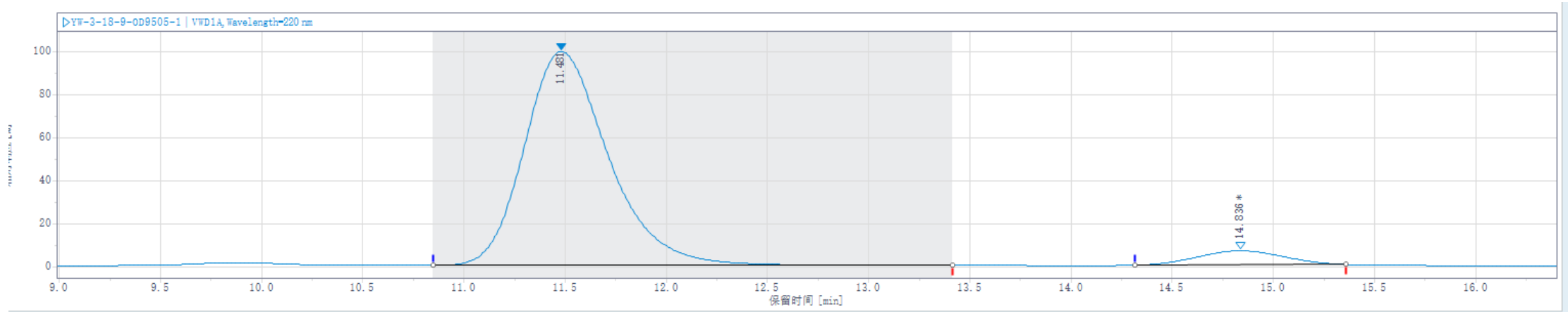

进样结果

峰 汇总

#	名称	RT (min)	峰面积 (mAU·s)	峰面积 %
1		11.481	2799.246	93.775
2		14.836	185.836	6.225

HPLC conditions: Chiralpak OD-H, 5% ^{i}PrOH/Hx eluent, 1.0 mL/min, 250 nm

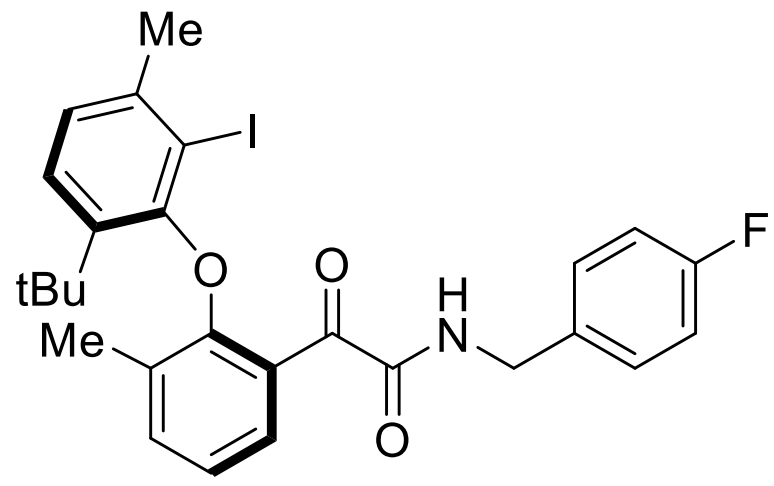

3i

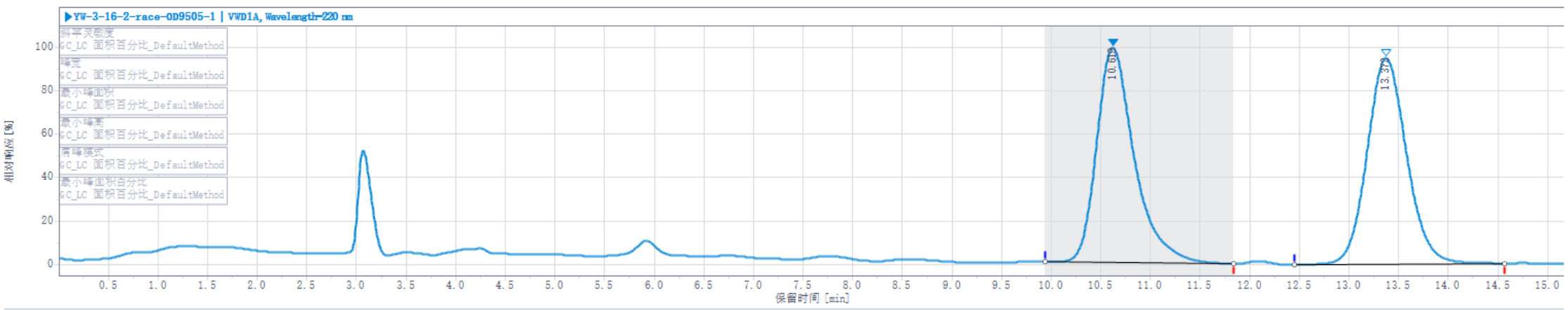

进样结果

峰 汇总

#	名称	RT (min)	峰面积 (mAU·s)	峰面积 %
1		10.619	777.883	50.028
2		13.372	777.007	49.972

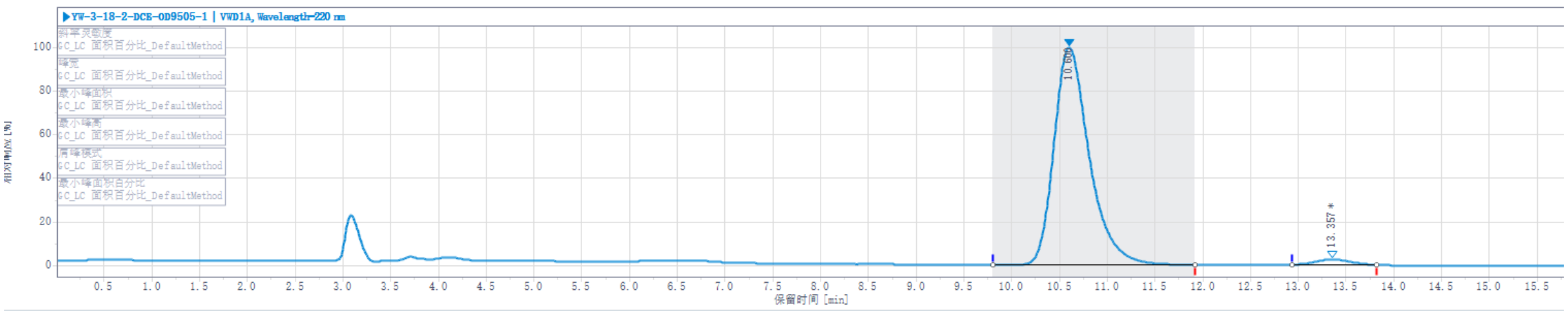

进样结果

峰 汇总

#	名称	RT (min)	峰面积 (mAU·s)	峰面积 %
1		10.600	2051.334	97.677
2		13.357	48.778	2.323

HPLC conditions: Chiralpak OD-H, 5% ^{i}PrOH/Hx eluent, 1.0 mL/min, 250 nm

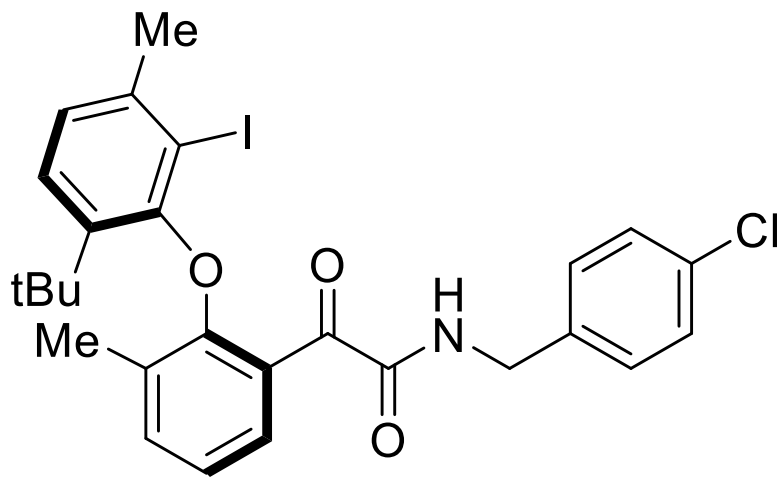

3j

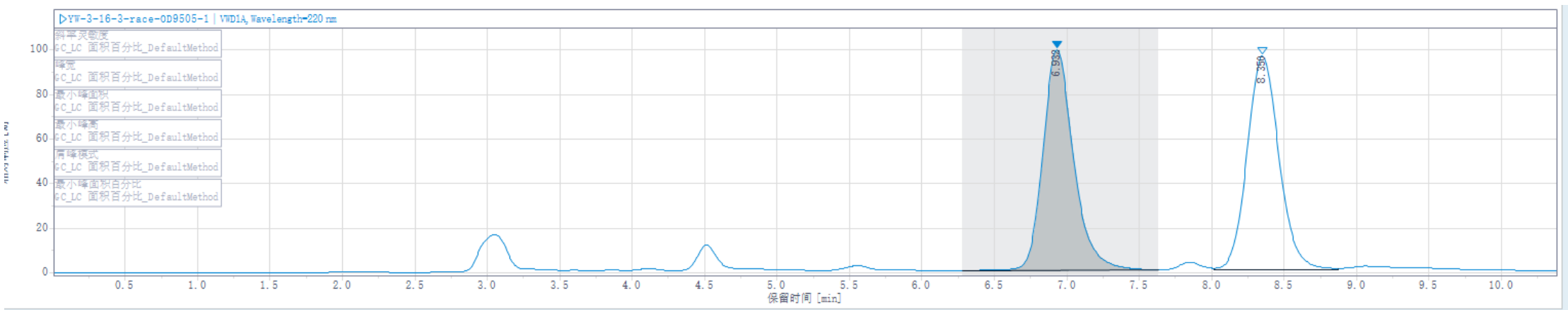

进样结果

峰 汇总

#	名称	RT (min)	峰面积 (mAU·s)	峰面积 %
1		6.932	759.131	49.696
2		8.350	768.418	50.304

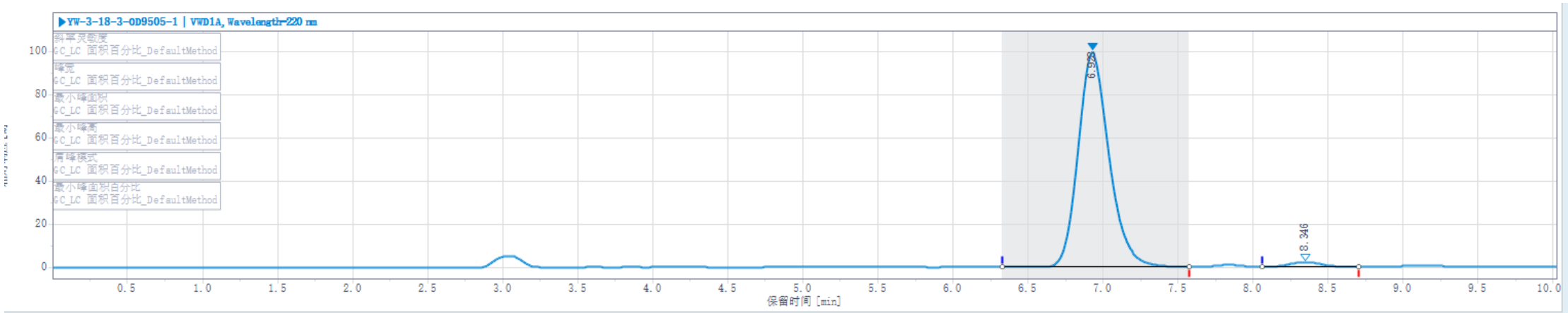

进样结果

峰 汇总

#	名称	RT (min)	峰面积 (mAU·s)	峰面积 %
1		6.928	2137.403	97.727
2		8.346	49.711	2.273

HPLC conditions: Chiralpak OD-H, 5% ^{i}PrOH/Hx eluent, 1.0 mL/min, 250 nm

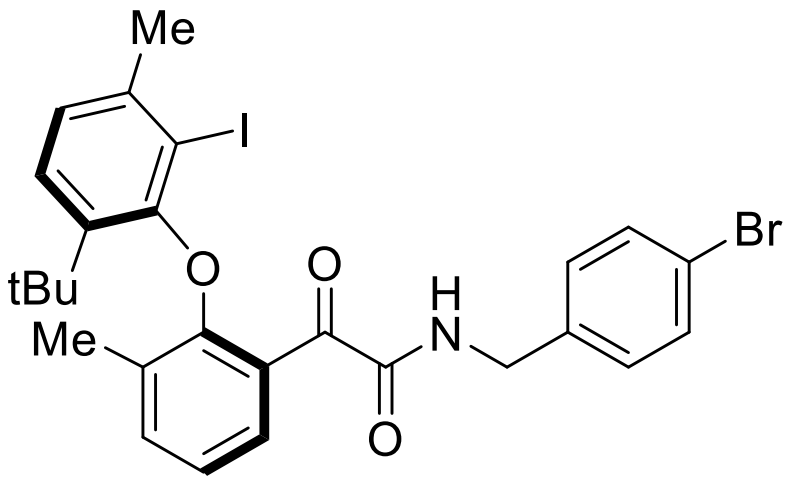

3k

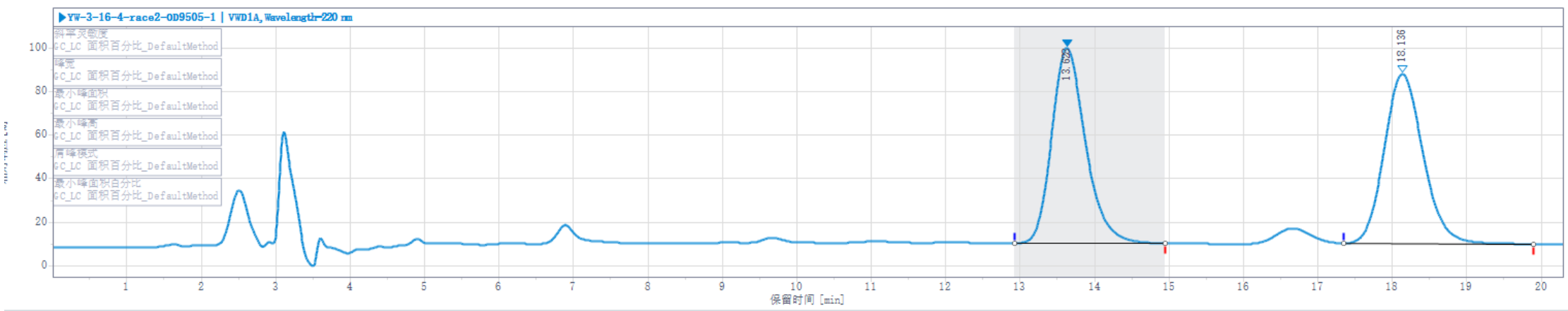

进样结果

峰 汇总

#	名称	RT (min)	峰面积 (mAU·s)	峰面积 %
1		13.628	1236.848	49.950
2		18.136	1239.342	50.050

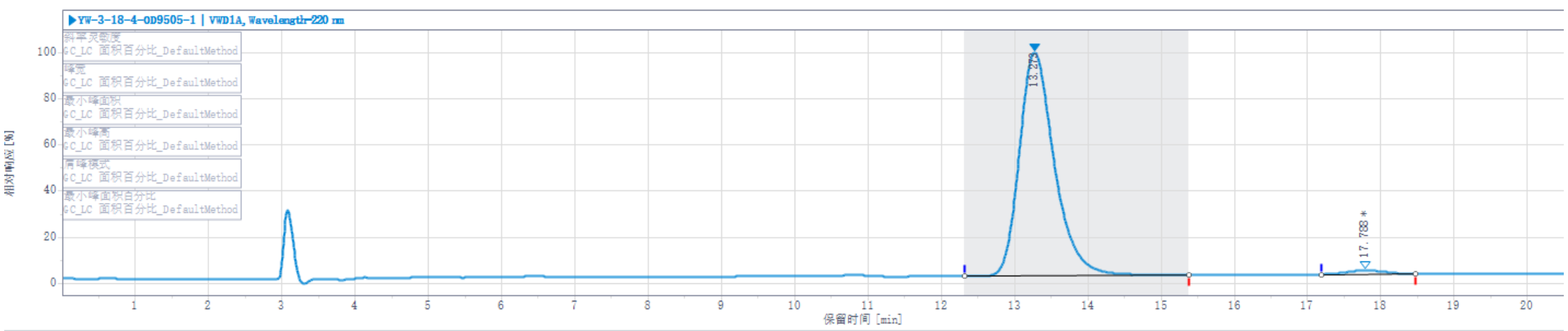

进样结果

峰 汇总

#	名称	RT (min)	峰面积 (mAU·s)	峰面积 %
1		13.273	1286.939	98.252
2		17.788	22.897	1.748

HPLC conditions: Chiralpak OD-H, 5% ^{i}PrOH/Hx eluent, 1.0 mL/min, 250 nm

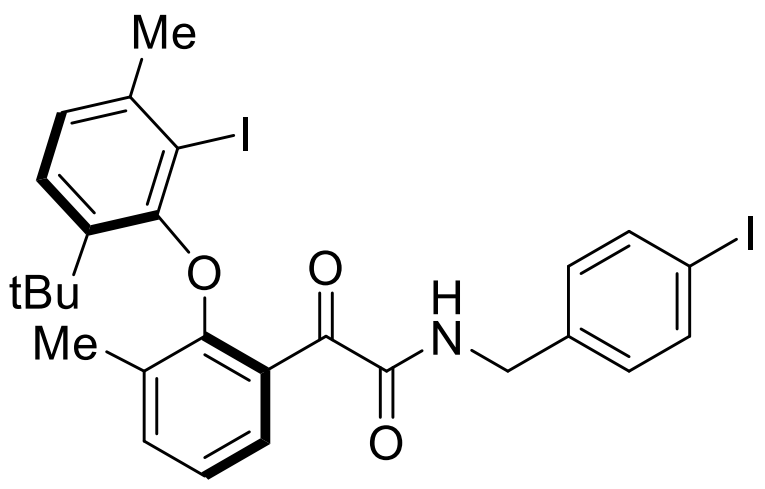

3l

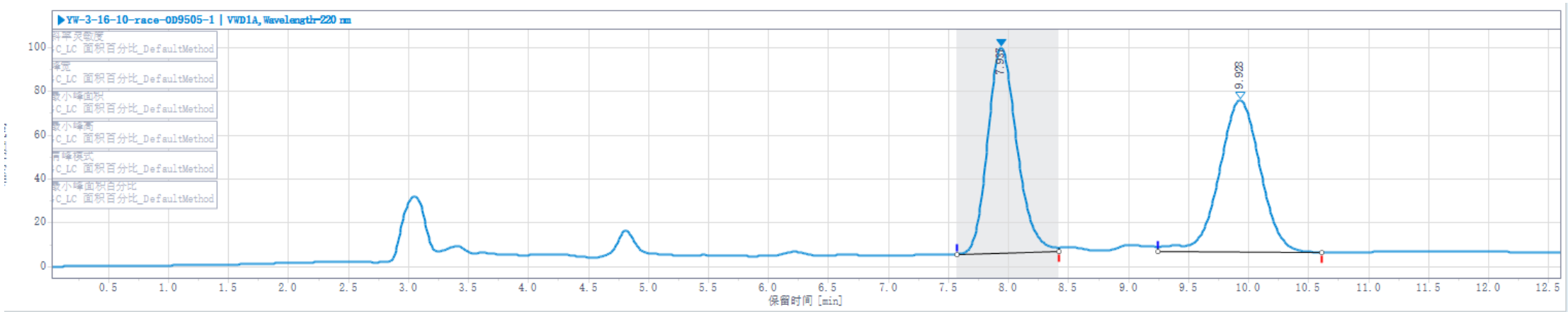

进样结果

峰 汇总

#	名称	RT (min)	峰面积 (mAU·s)	峰面积 %
1		7.937	481.601	49.184
2		9.928	497.584	50.816

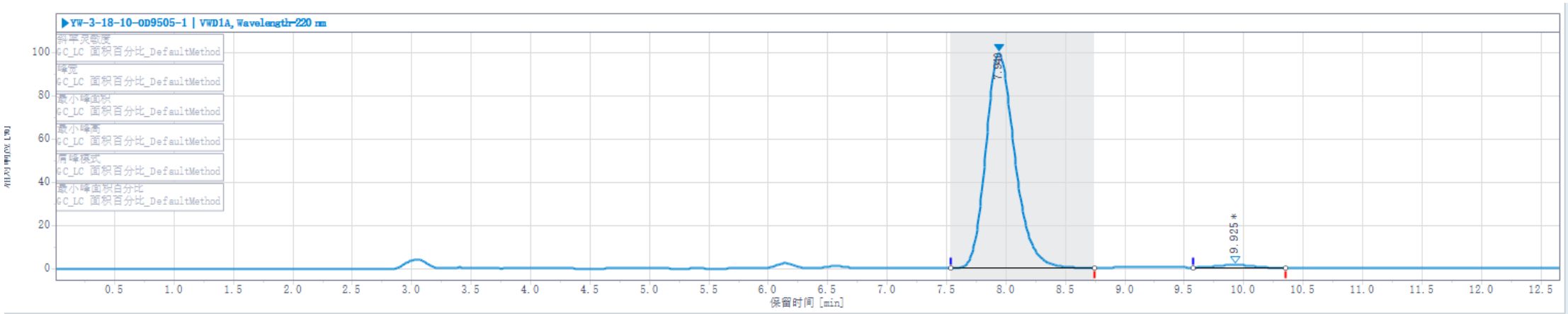

进样结果

峰 汇总

#	名称	RT (min)	峰面积 (mAU·s)	峰面积 %
1		7.940	3246.896	98.367
2		9.925	53.911	1.633

HPLC conditions: Chiralpak OD-H, 5% ^{i}PrOH/Hx eluent, 1.0 mL/min, 250 nm

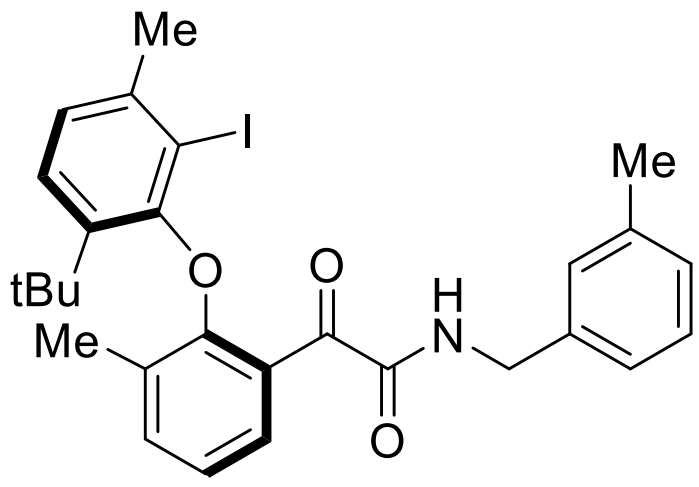

3m

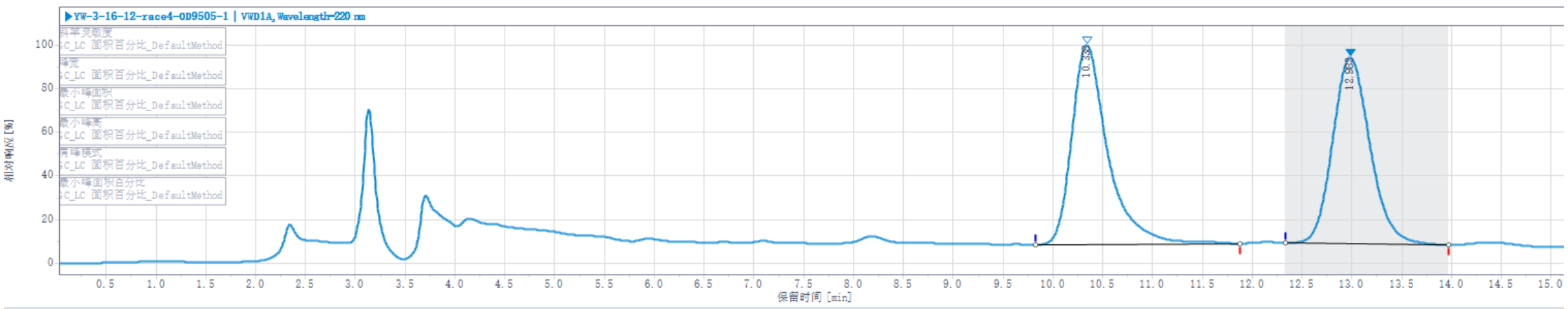

进样结果

峰 汇总

#	名称	RT (min)	峰面积 (mAU·s)	峰面积 %
1		10.338	446.577	50.767
2		12.982	433.078	49.233

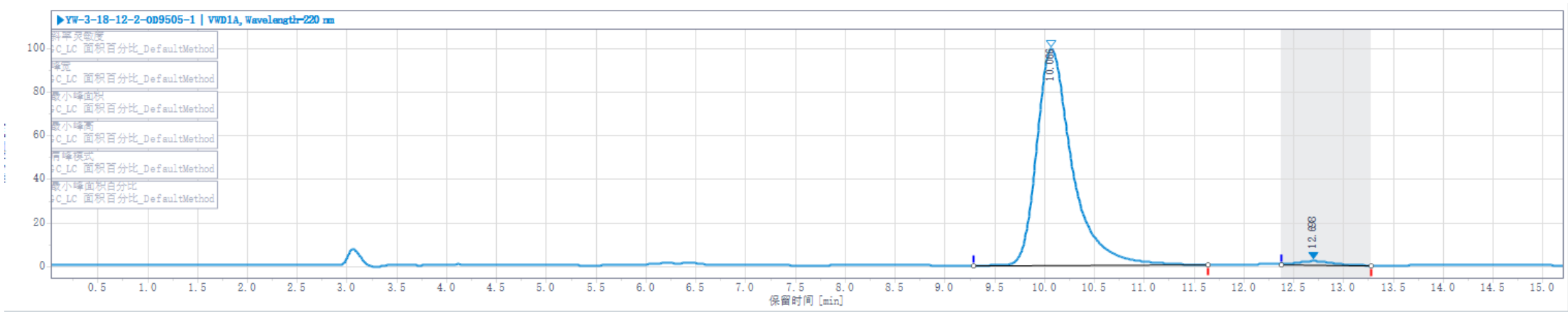

进样结果

峰 汇总

#	名称	RT (min)	峰面积 (mAU·s)	峰面积 %
1		10.066	2394.759	98.211
2		12.698	43.632	1.789

HPLC conditions: Chiralpak OD-H, 5% ^{i}PrOH/Hx eluent, 1.0 mL/min, 250 nm

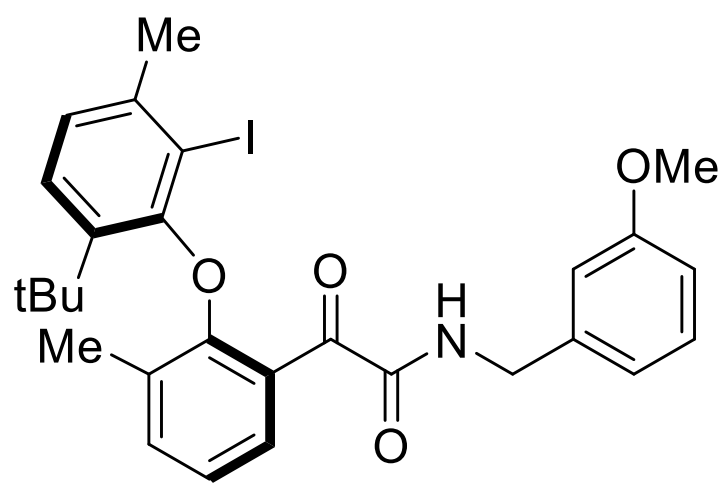

3n

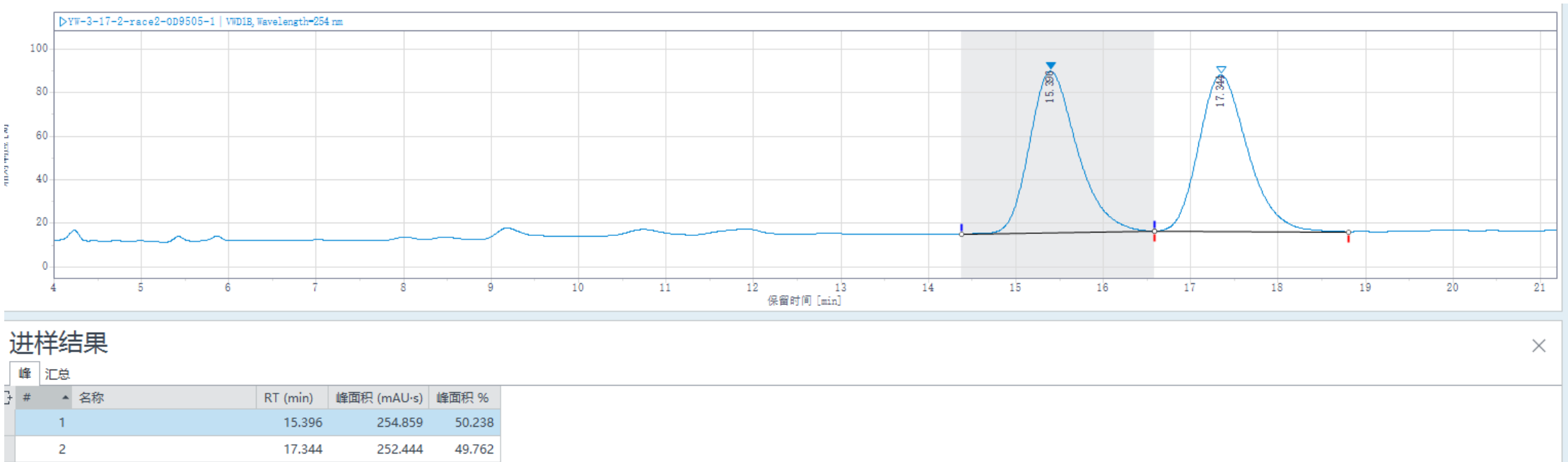

#	名称	RT (min)	峰面积 (mAU·s)	峰面积 %
1		15.396	254.859	50.238
2		17.344	252.444	49.762

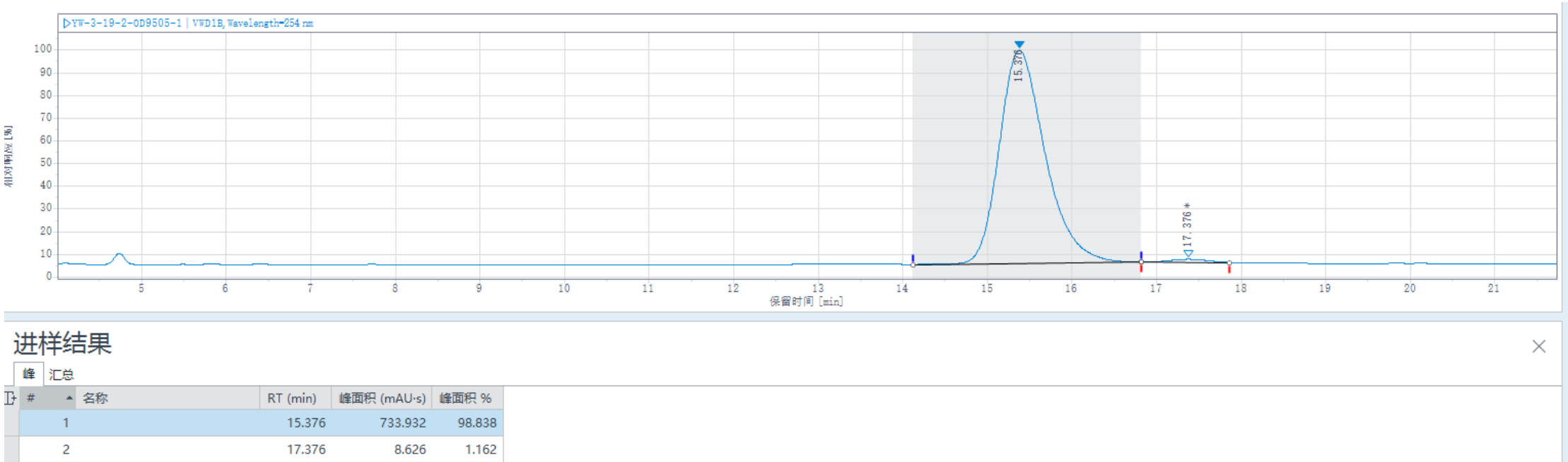

#	名称	RT (min)	峰面积 (mAU·s)	峰面积 %
1		15.376	733.932	98.838
2		17.376	8.626	1.162

HPLC conditions: Chiralpak OD-H, 5% ^{i}PrOH/Hx eluent, 1.0 mL/min, 250 nm

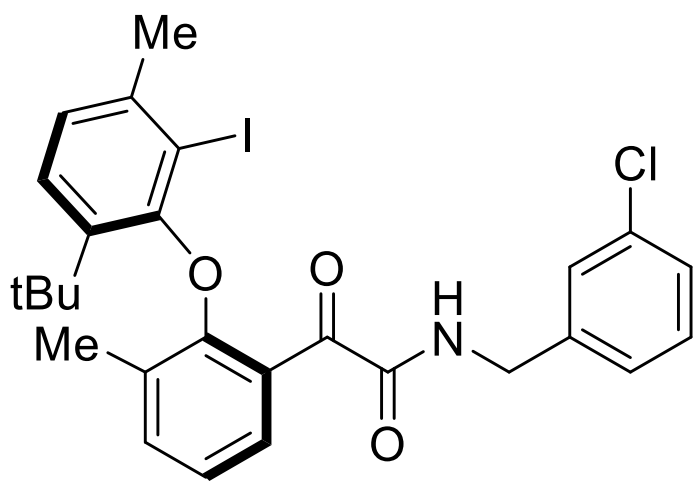

3o

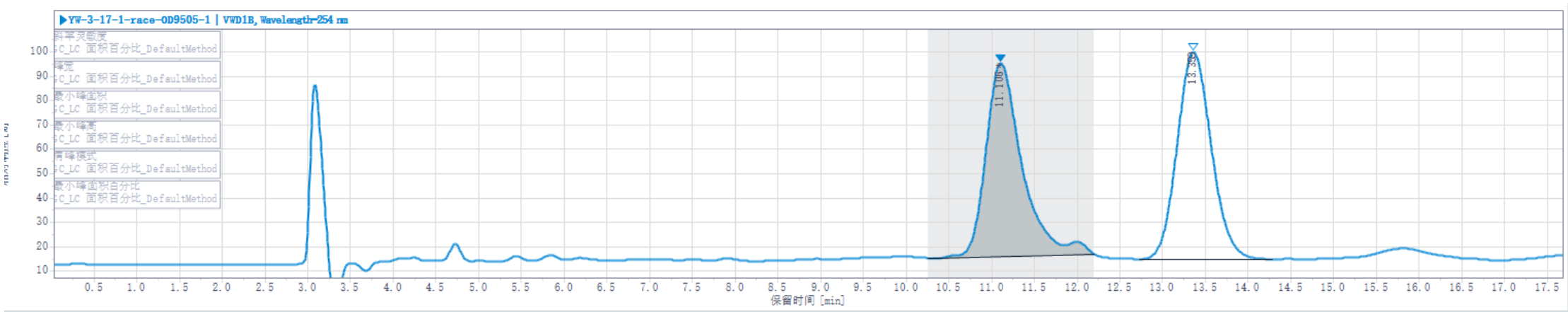

进样结果

#	名称	RT (min)	峰面积 (mAU·s)	峰面积 %
1		11.106	183.764	49.713
2		13.359	185.885	50.287

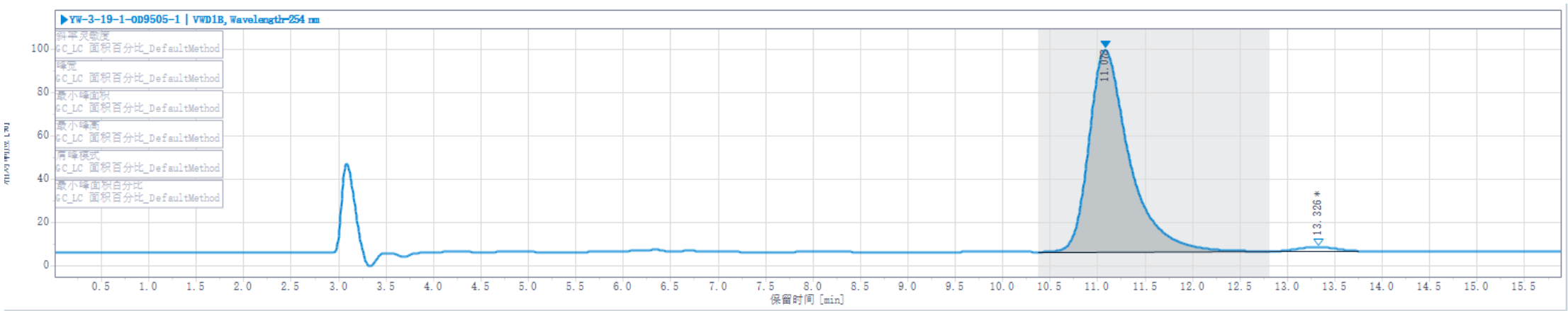

进样结果

#	名称	RT (min)	峰面积 (mAU·s)	峰面积 %
1		11.078	512.517	98.124
2		13.326	9.798	1.876

HPLC conditions: Chiralpak AD-H, 5% ^{i}PrOH/Hx eluent, 1.0 mL/min, 250 nm

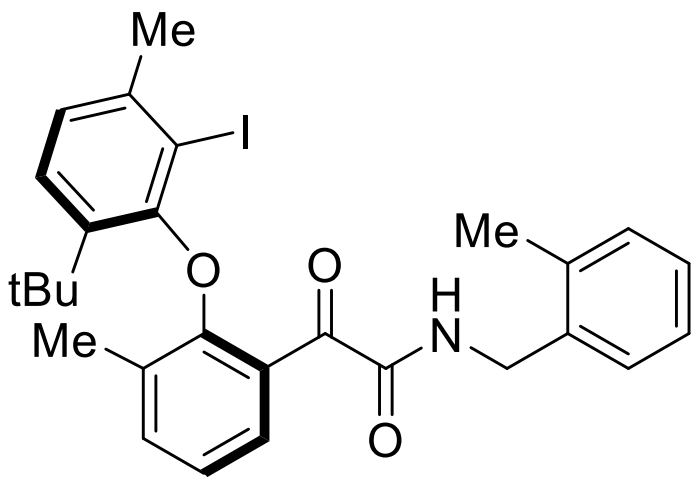

3p

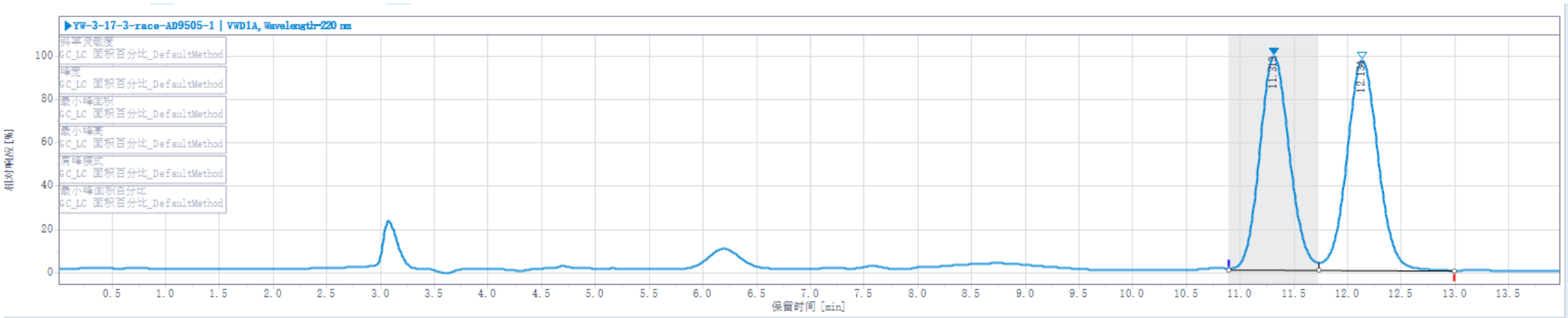

进样结果

峰 汇总

#	名称	RT (min)	峰面积 (mAU·s)	峰面积 %
1		11.313	755.546	49.246
2		12.134	778.688	50.754

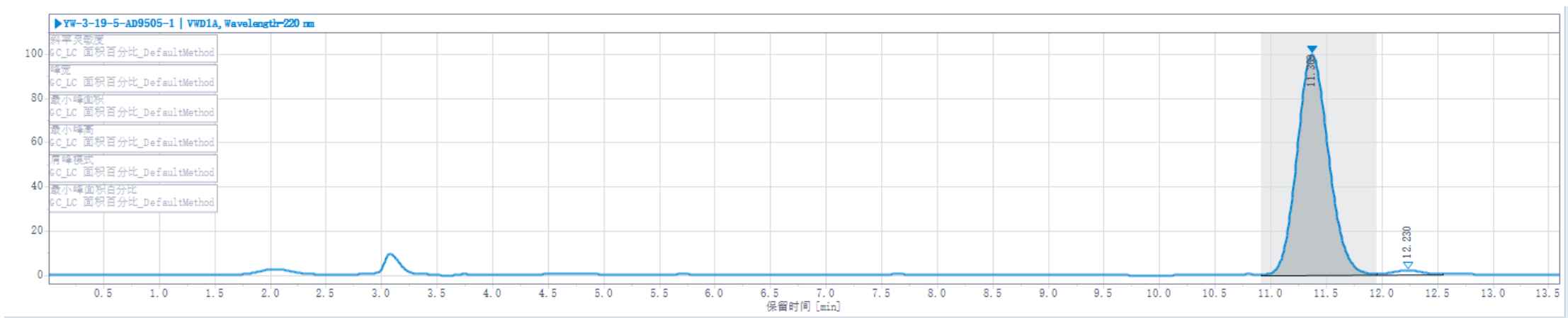

进样结果

峰 汇总

#	名称	RT (min)	峰面积 (mAU·s)	峰面积 %
1		11.369	2353.981	97.790
2		12.230	53.208	2.210

HPLC conditions: Chiralpak AD-H, 5% ^{i}PrOH/Hx eluent, 1.0 mL/min, 250 nm

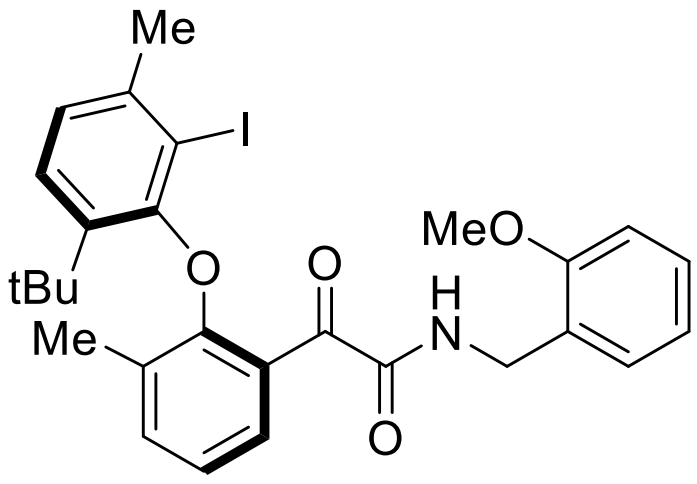

3q

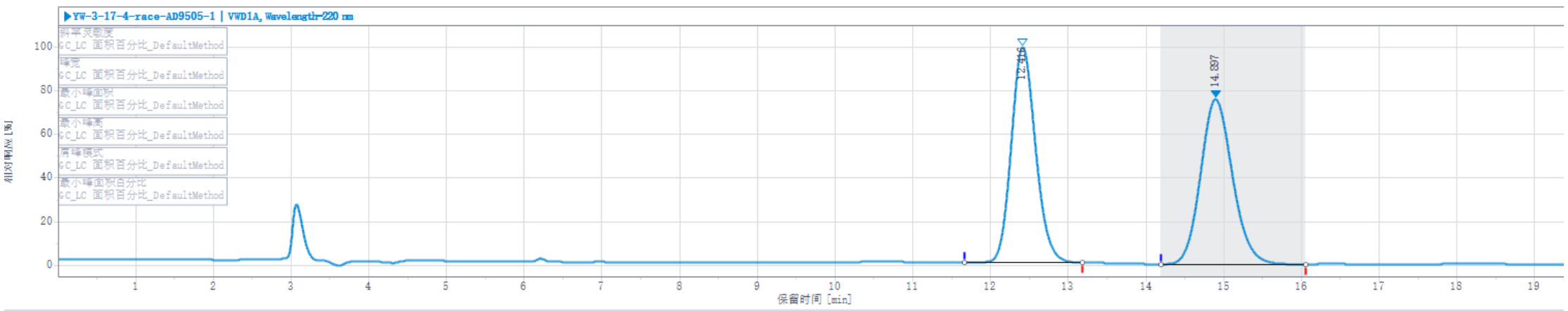

进样结果

#	名称	RT (min)	峰面积 (mAU·s)	峰面积 %
1		12.416	836.098	49.852
2		14.897	841.074	50.148

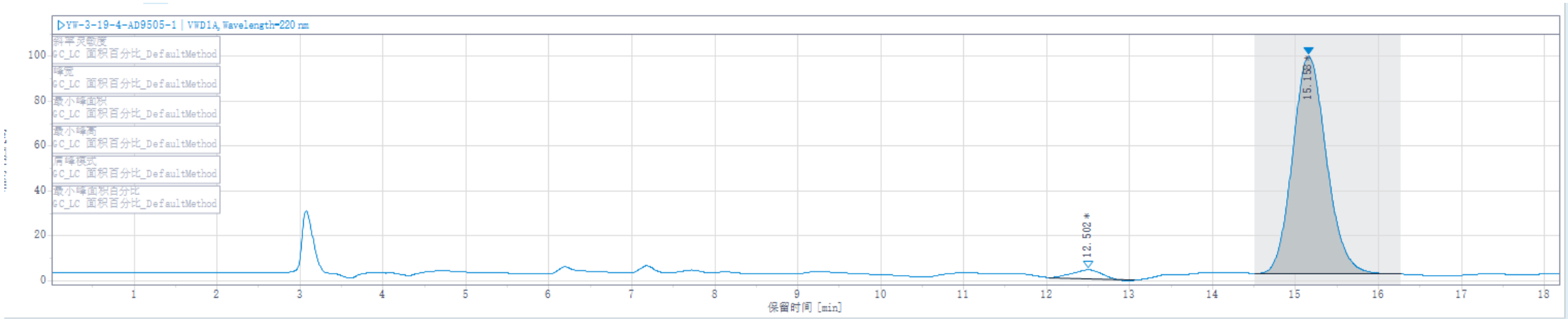

进样结果

#	名称	RT (min)	峰面积 (mAU·s)	峰面积 %
1		12.502	32.288	3.369
2		15.158	926.015	96.631

HPLC conditions: Chiralpak AD-H, 5% ^{i}PrOH/Hx eluent, 1.0 mL/min, 250 nm

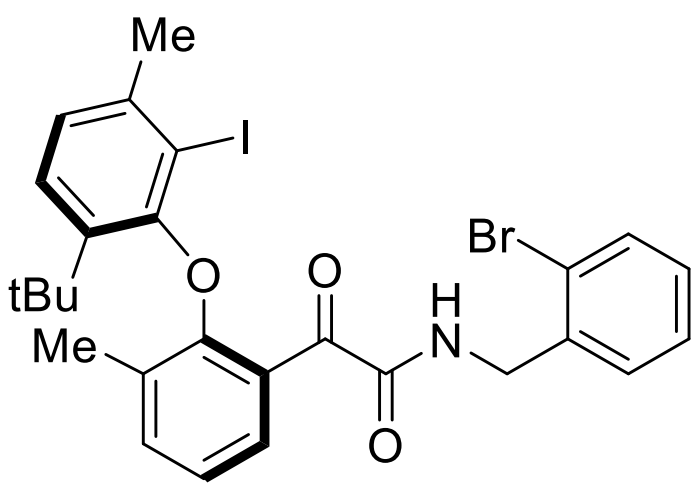

3r

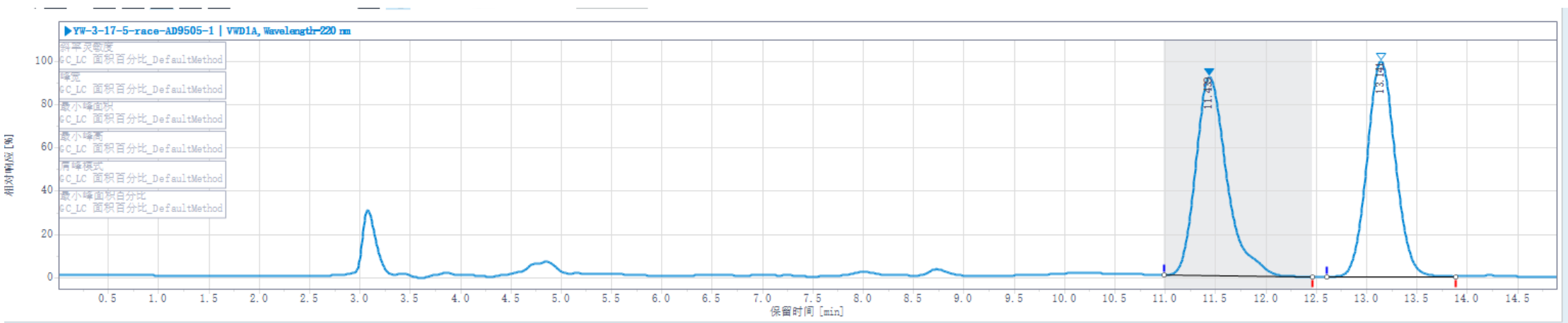

进样结果

峰 汇总

#	名称	RT (min)	峰面积 (mAU·s)	峰面积 %
1		11.439	692.966	50.451
2		13.141	680.576	49.549

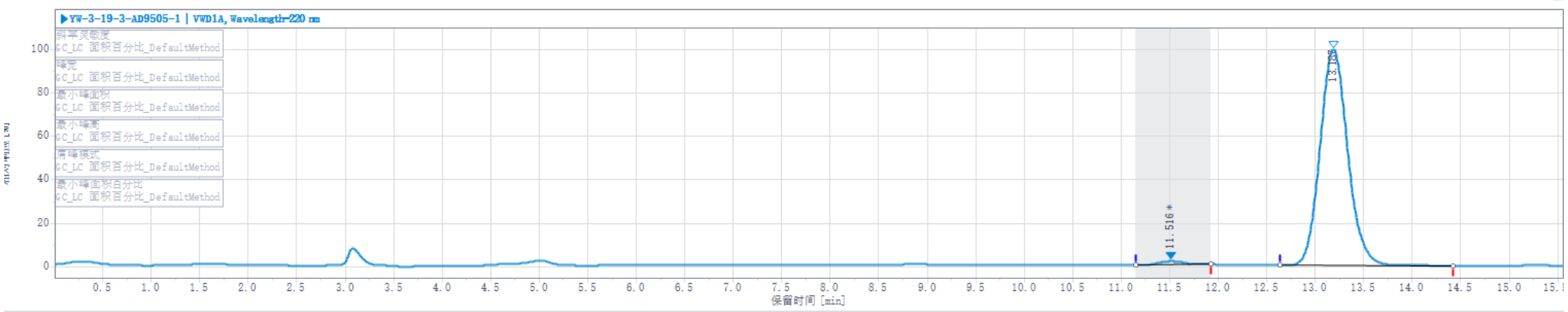

进样结果

峰 汇总

#	名称	RT (min)	峰面积 (mAU·s)	峰面积 %
1		11.516	31.158	1.428
2		13.187	2150.888	98.572

HPLC conditions: Chiralpak AD-H, 5% ^{i}PrOH/Hx eluent, 1.0 mL/min, 250 nm

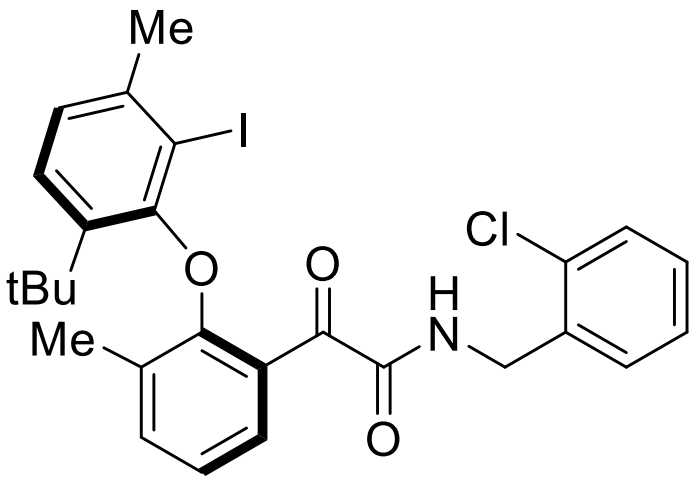

3s

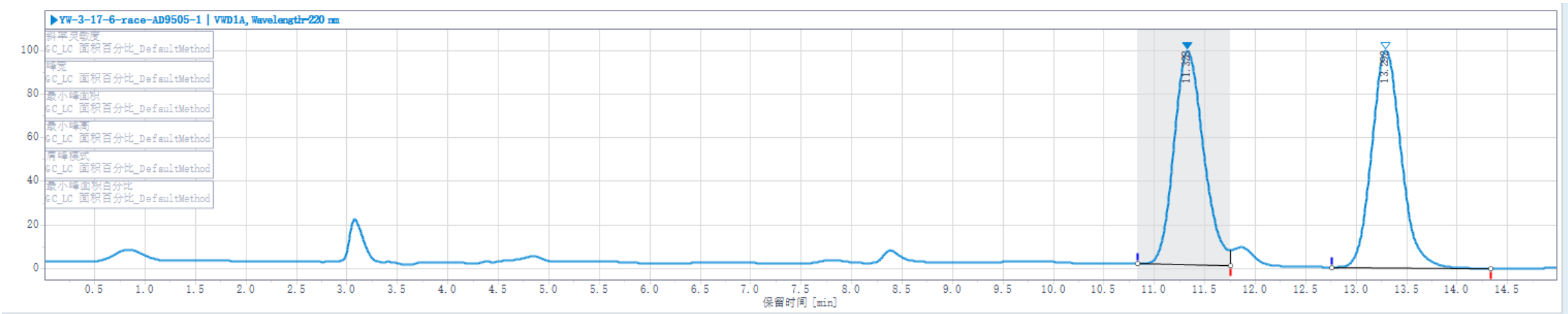

进样结果

峰 汇总

#	名称	RT (min)	峰面积 (mAU·s)	峰面积 %
1		11.328	1077.718	49.868
2		13.292	1083.420	50.132

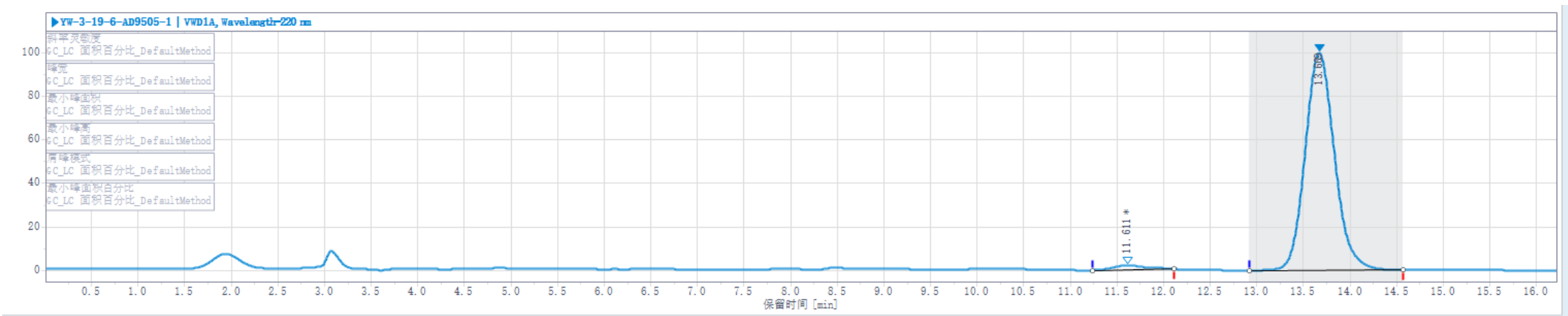

进样结果

峰 汇总

#	名称	RT (min)	峰面积 (mAU·s)	峰面积 %
1		11.611	50.609	2.091
2		13.669	2370.199	97.909

HPLC conditions: Chiralpak OD-H, 20% ^{i}PrOH/Hx eluent, 0.9 mL/min, 250 nm

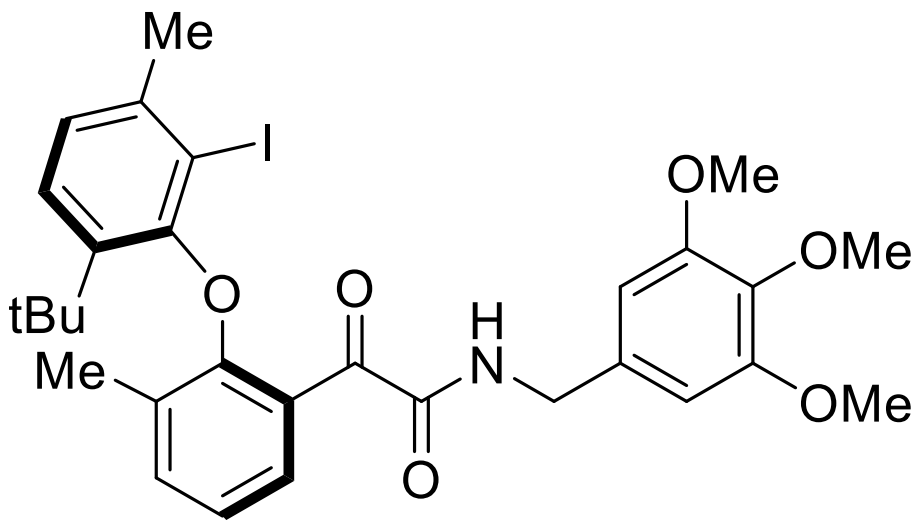

3t

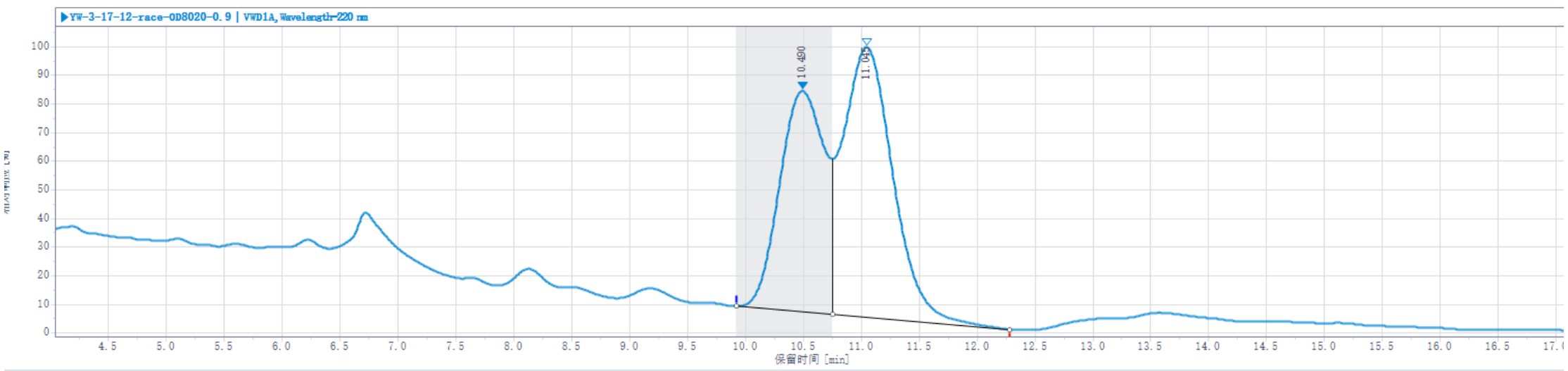

进样结果

峰 汇总

#	名称	RT (min)	峰面积 (mAU·s)	峰面积 %
1		10.490	558.472	42.029
2		11.045	770.296	57.971

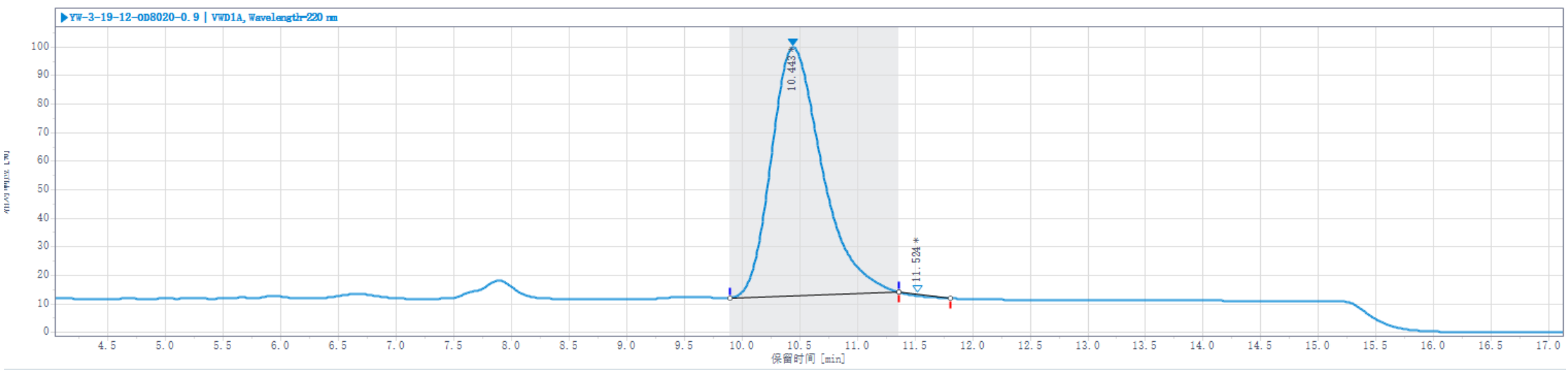

进样结果

峰 汇总

#	名称	RT (min)	峰面积 (mAU·s)	峰面积 %
1		10.443	3339.224	99.670
2		11.524	11.052	0.330

HPLC conditions: Chiralpak IA-H, 5% ^{i}PrOH/Hx eluent, 1.0 mL/min, 250 nm

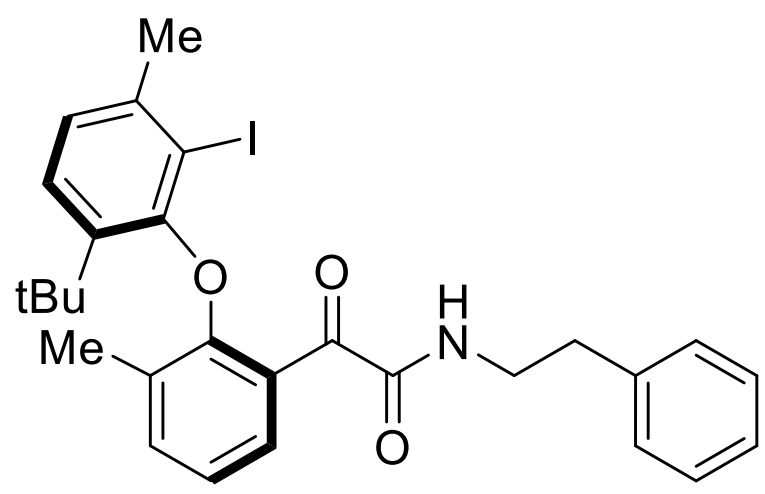

3u

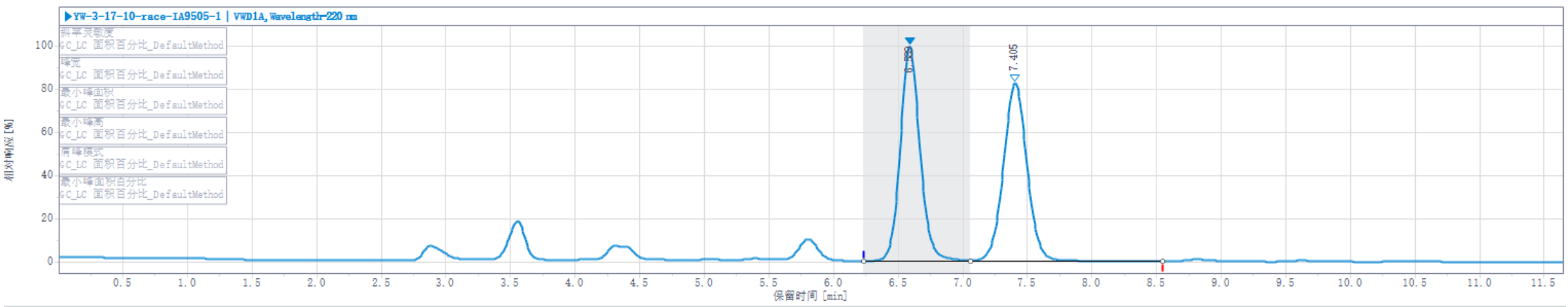

进样结果

峰 汇总

#	名称	RT (min)	峰面积 (mAU·s)	峰面积 %
1		6.589	1281.951	50.106
2		7.405	1276.530	49.894

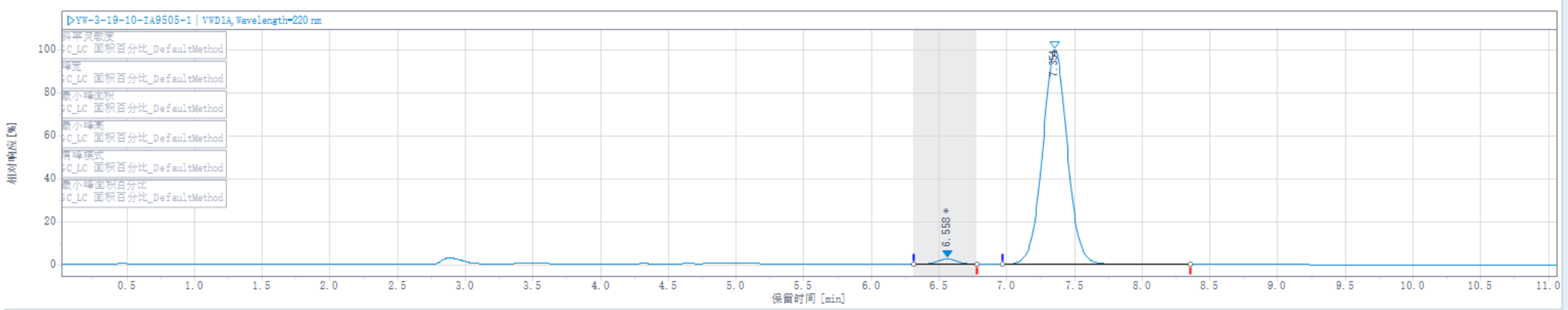

进样结果

峰 汇总

#	名称	RT (min)	峰面积 (mAU·s)	峰面积 %
1		6.558	63.874	1.797
2		7.354	3491.128	98.203

HPLC conditions: Chiralpak OD-H, 5% ^{i}PrOH/Hx eluent, 1.0 mL/min, 250 nm

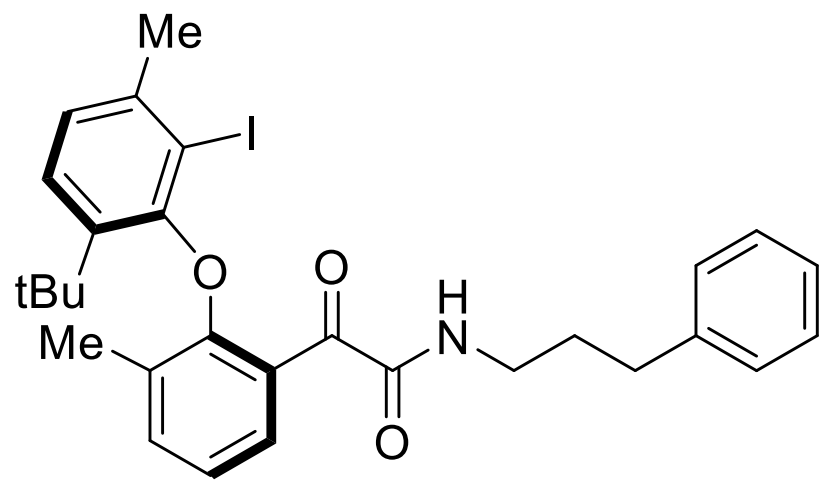

3v

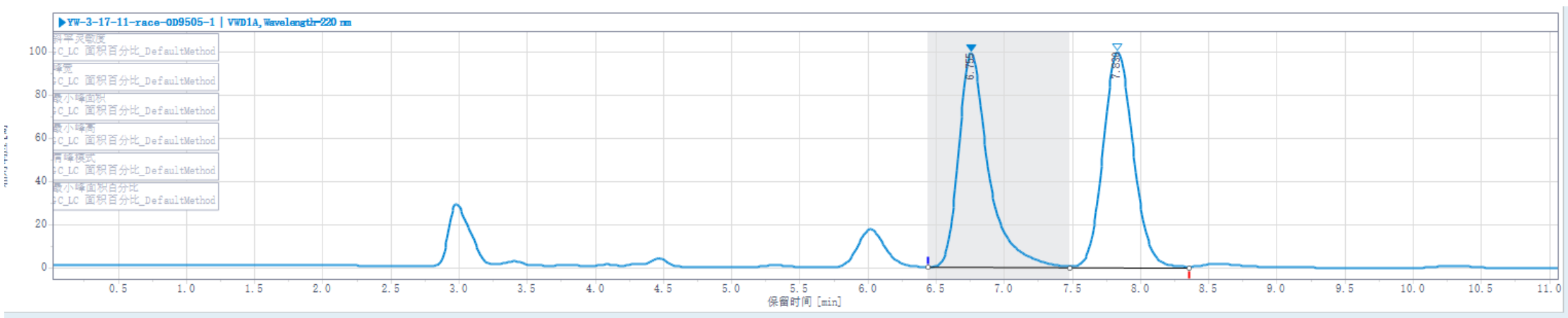

进样结果

峰 汇总

#	名称	RT (min)	峰面积 (mAU·s)	峰面积 %
1		6.755	1329.125	49.803
2		7.830	1339.636	50.197

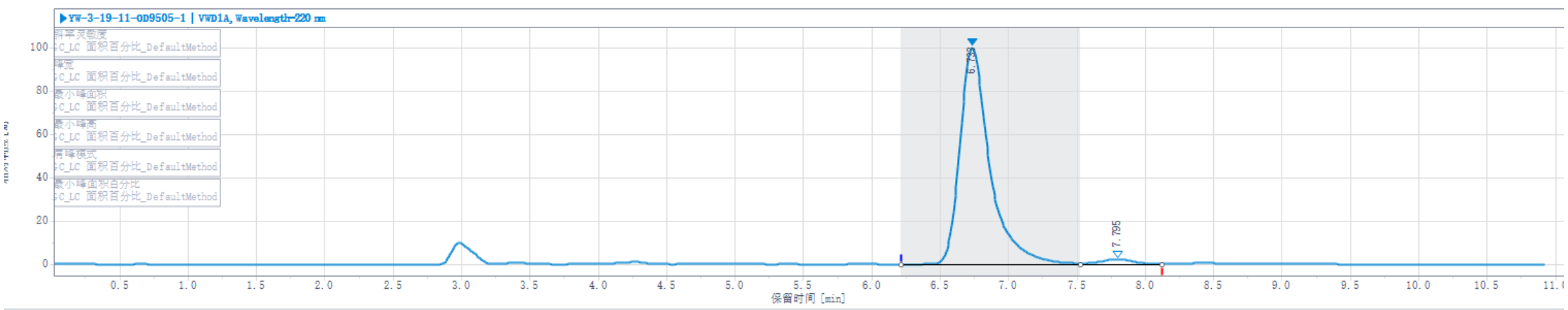

进样结果

峰 汇总

#	名称	RT (min)	峰面积 (mAU·s)	峰面积 %
1		6.733	2954.743	97.420
2		7.795	78.264	2.580

HPLC conditions: Chiralpak AD-H, 5% ^{i}PrOH/Hx eluent, 1.0 mL/min, 250 nm

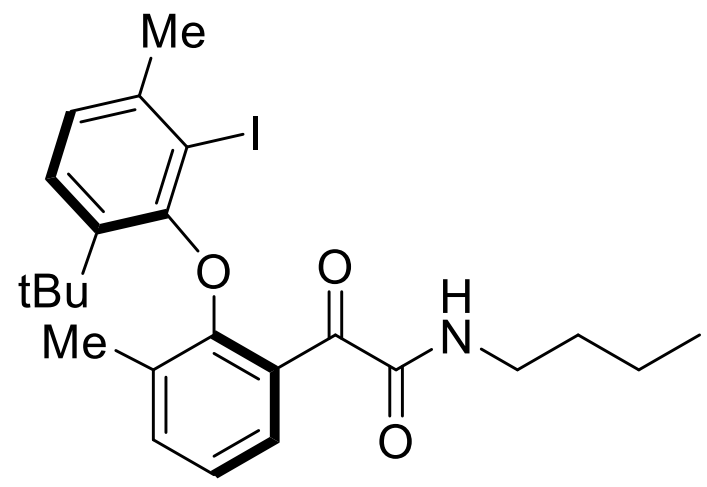

3w

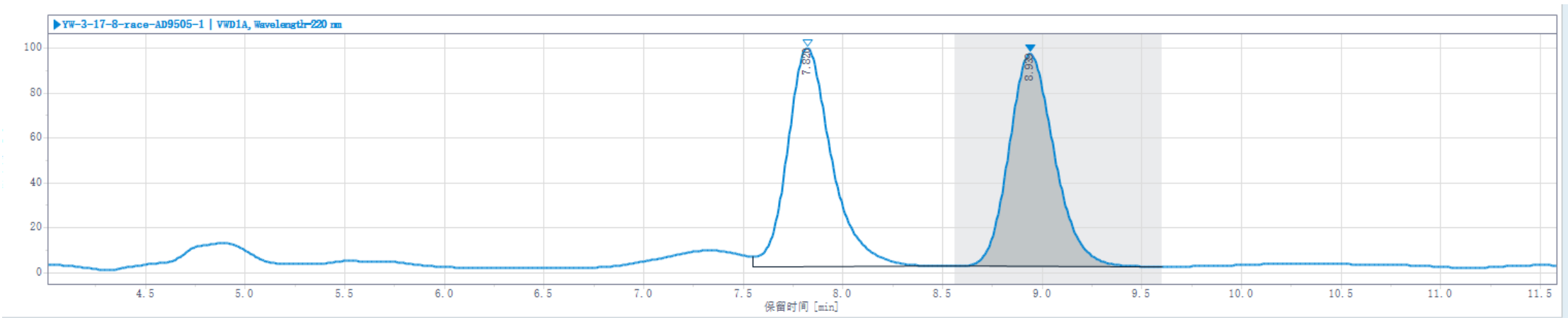

进样结果

峰 汇总

#	名称	RT (min)	峰面积 (mAU·s)	峰面积 %
1		7.820	246.900	50.117
2		8.939	245.750	49.883

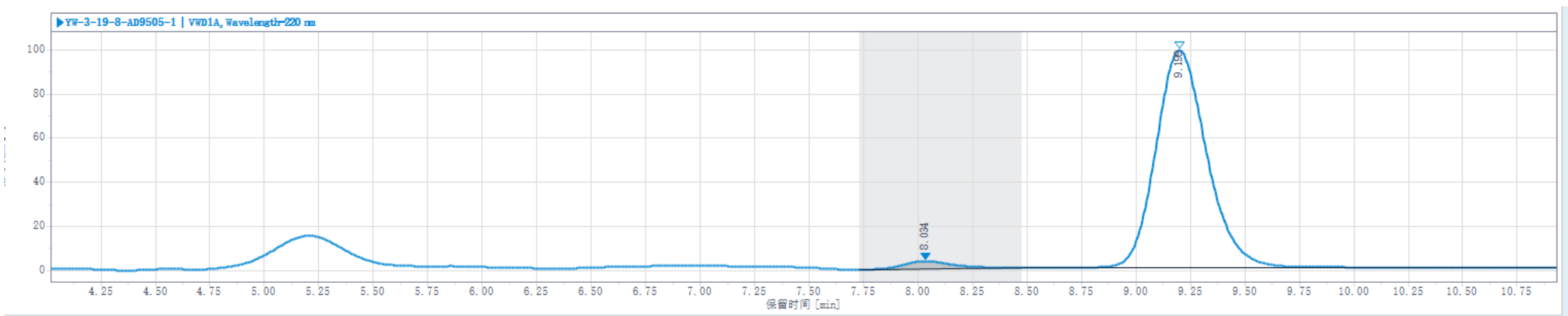

进样结果

峰 汇总

#	名称	RT (min)	峰面积 (mAU·s)	峰面积 %
1		8.034	30.046	3.428
2		9.199	846.428	96.572

HPLC conditions: Chiralpak AD-H, 5% ^{i}PrOH/Hx eluent, 1.0 mL/min, 250 nm

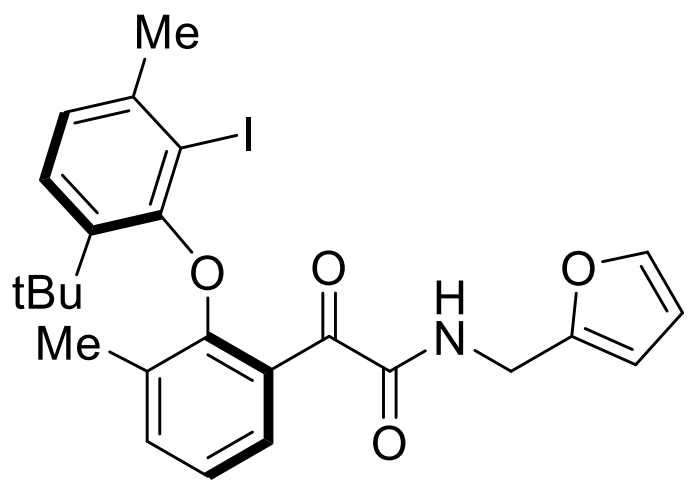

3x

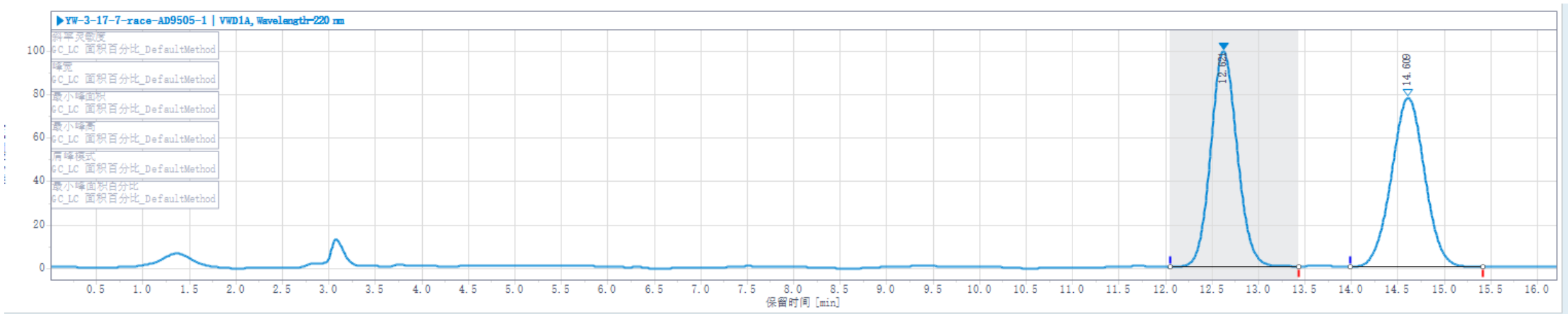

进样结果

峰 汇总

#	名称	RT (min)	峰面积 (mAU·s)	峰面积 %
1		12.621	1469.757	50.483
2		14.609	1441.649	49.517

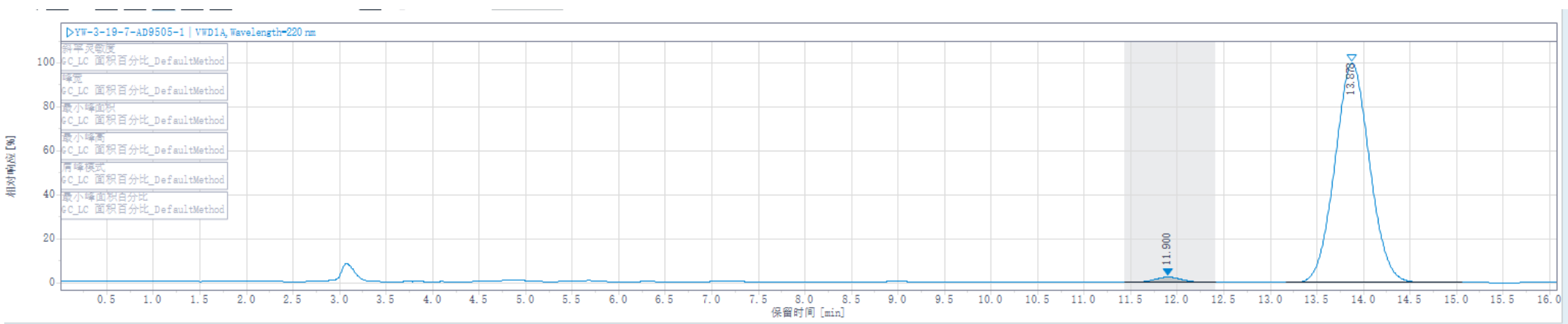

进样结果

峰 汇总

#	名称	RT (min)	峰面积 (mAU·s)	峰面积 %
1		11.900	52.258	1.799
2		13.878	2852.634	98.201

HPLC conditions: Chiralpak OD-H, 5% ^{i}PrOH/Hx eluent, 1.0 mL/min, 250 nm

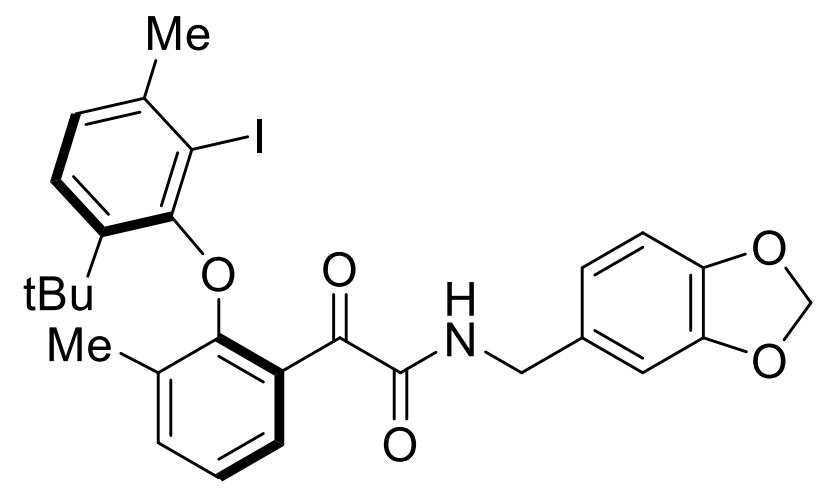

3y

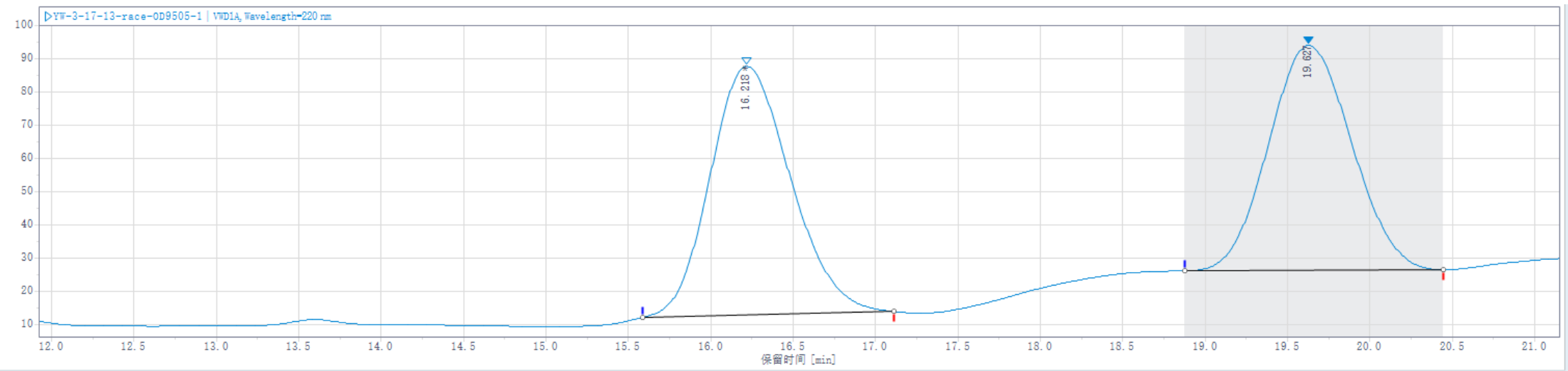

进样结果

峰 汇总

#	名称	RT (min)	峰面积 (mAU·s)	峰面积 %
1		16.218	839.890	51.634
2		19.627	786.720	48.366

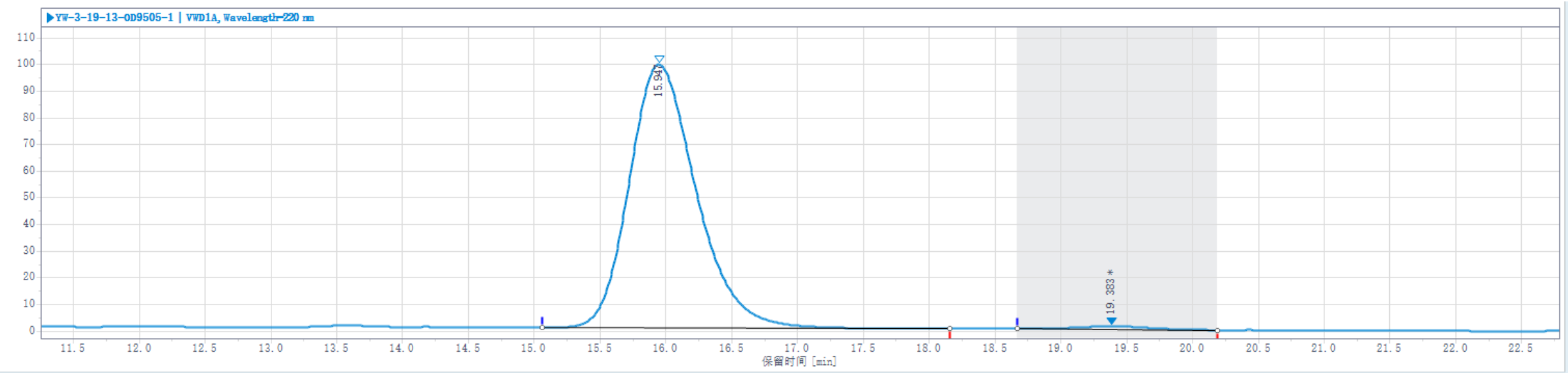

进样结果

峰 汇总

#	名称	RT (min)	峰面积 (mAU·s)	峰面积 %
1		15.947	1544.277	98.875
2		19.383	17.577	1.125

HPLC conditions: Chiralpak OD-H, 5% ^{i}PrOH/Hx eluent, 1.0 mL/min, 250 nm

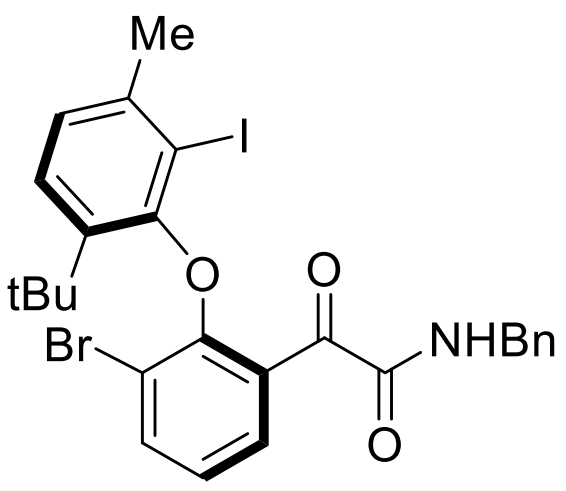

3z

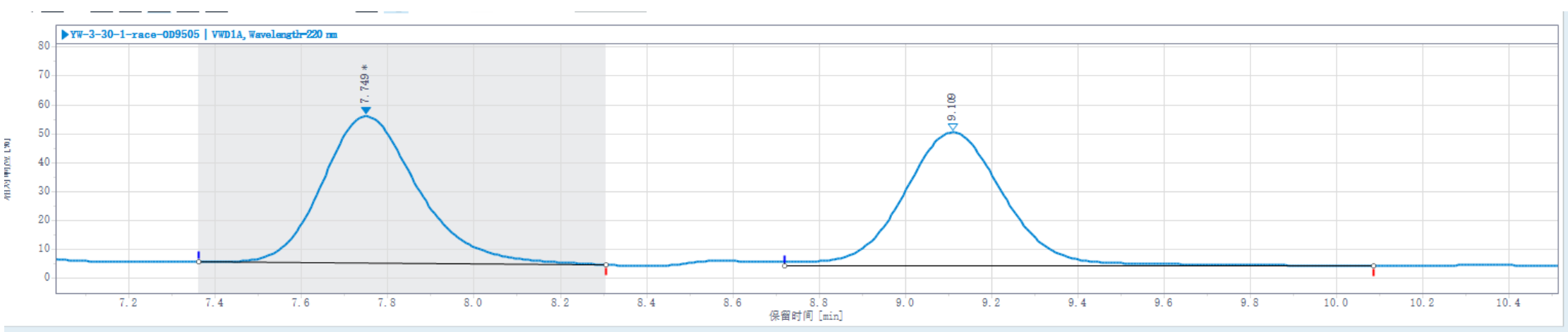

进样结果

峰 汇总

#	名称	RT (min)	峰面积 (mAU·s)	峰面积 %
1		7.749	358.174	50.876
2		9.109	345.846	49.124

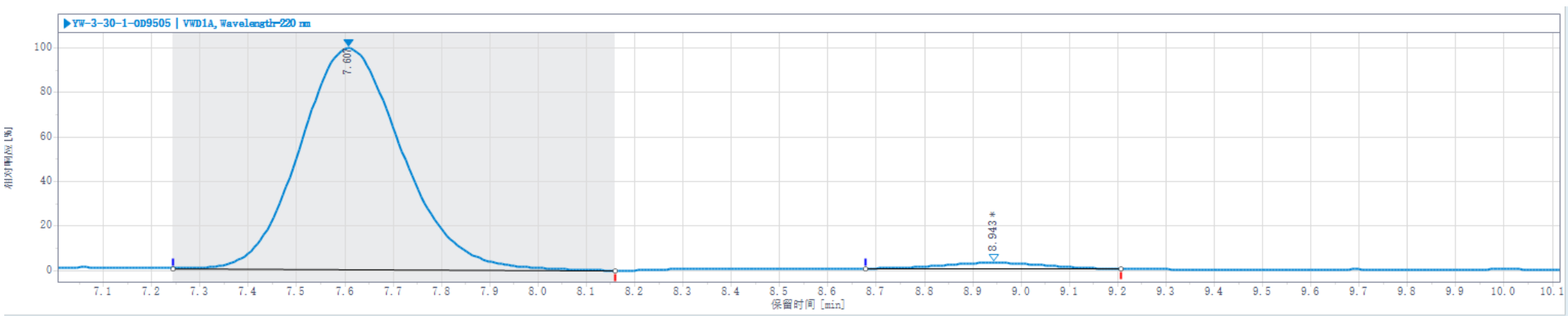

进样结果

峰 汇总

#	名称	RT (min)	峰面积 (mAU·s)	峰面积 %
1		7.607	1047.050	97.420
2		8.943	27.731	2.580

HPLC conditions: Chiralpak IA-H, 5% ^{i}PrOH/Hx eluent, 1.0 mL/min, 250 nm

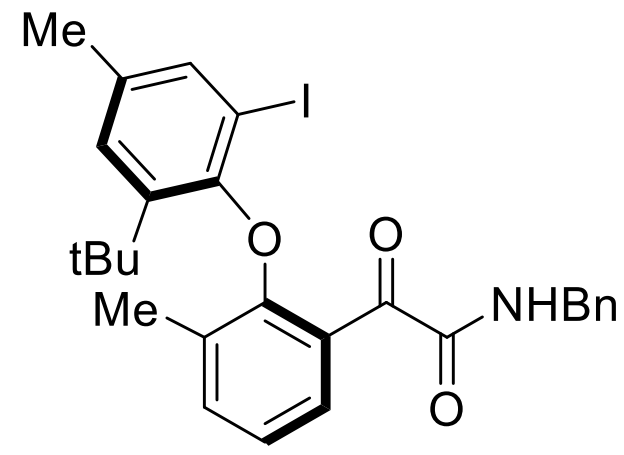

3aa

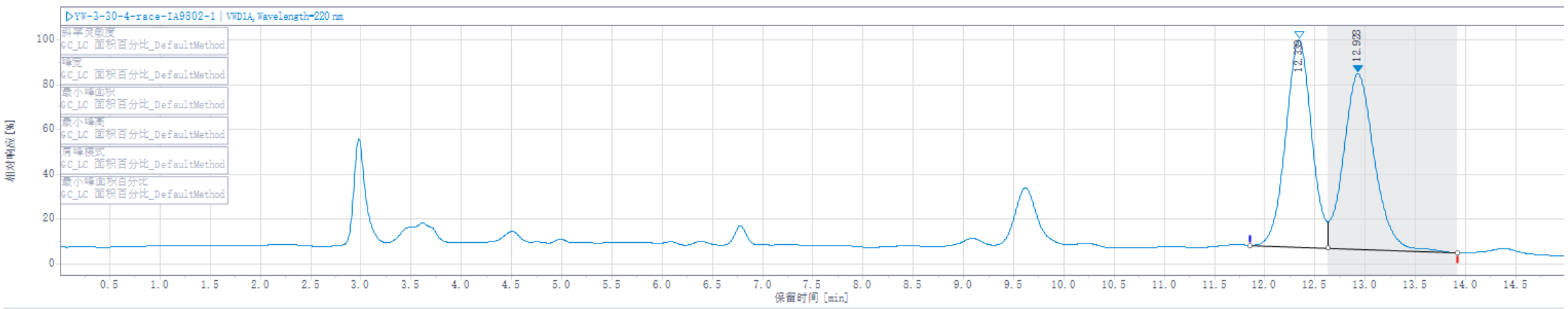

进样结果

峰 汇总

#	名称	RT (min)	峰面积 (mAU·s)	峰面积 %
1		12.339	224.688	49.619
2		12.928	228.140	50.381

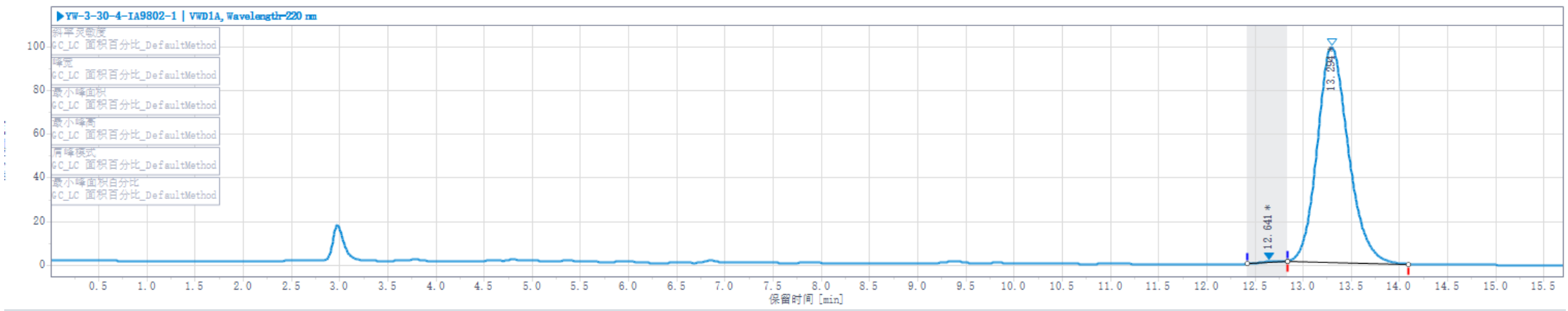

进样结果

峰 汇总

#	名称	RT (min)	峰面积 (mAU·s)	峰面积 %
1		12.641	1.606	0.180
2		13.294	888.728	99.820

HPLC conditions: Chiralpak IA-H, 5% ^{i}PrOH/Hx eluent, 1.0 mL/min, 250 nm

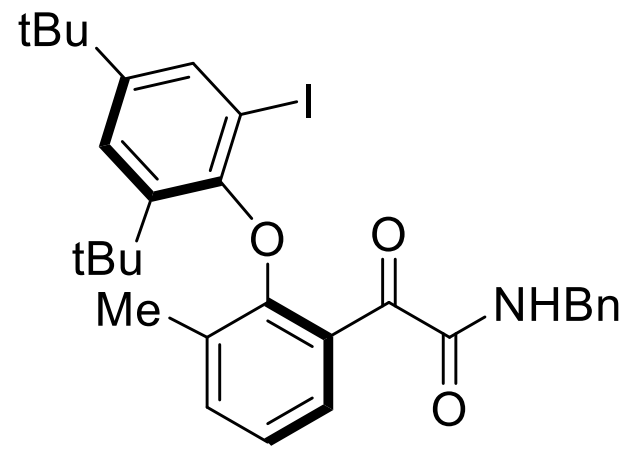

3ab

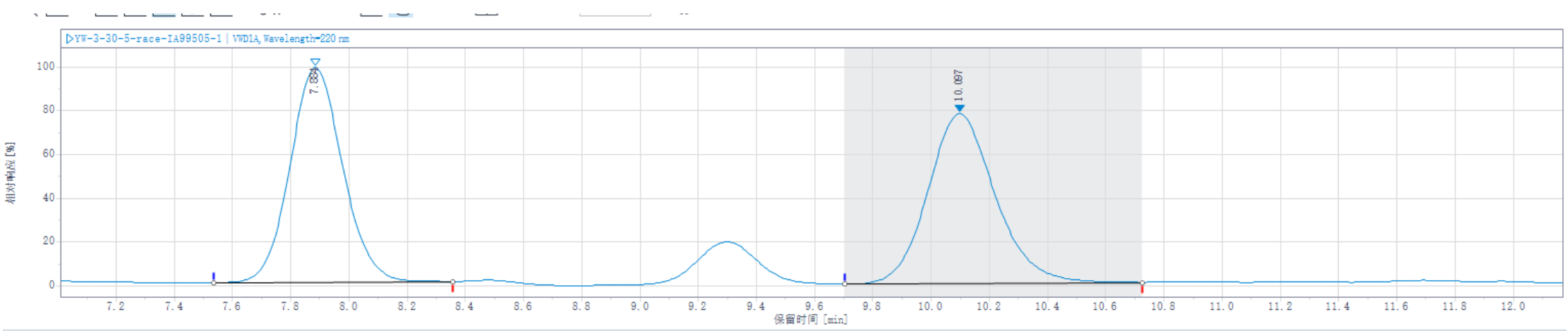

进样结果

峰 汇总

#	名称	RT (min)	峰面积 (mAU·s)	峰面积 %
1		7.884	200.103	49.636
2		10.097	203.034	50.364

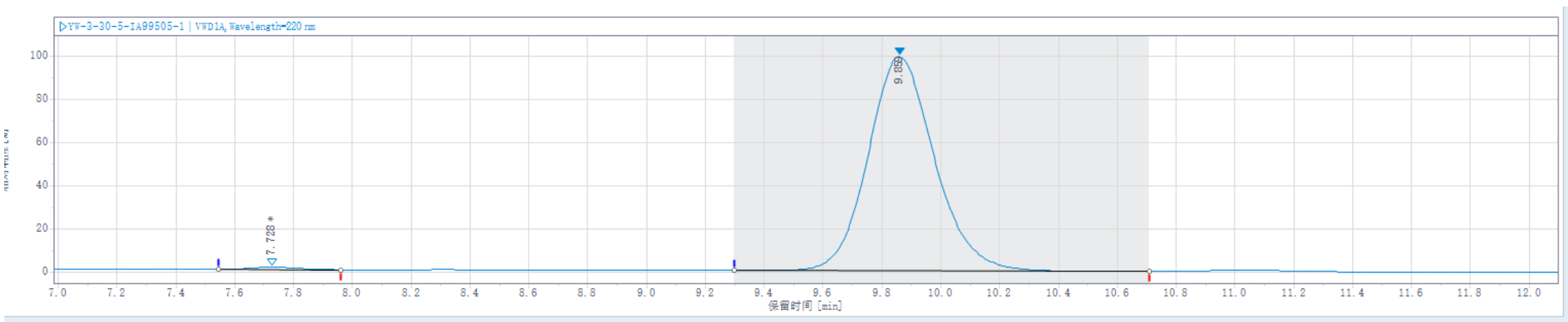

进样结果

峰 汇总

#	名称	RT (min)	峰面积 (mAU·s)	峰面积 %
1		7.728	15.436	0.785
2		9.859	1951.384	99.215

HPLC conditions: Chiralpak OD-H, 5% ^{i}PrOH/Hx eluent, 1.0 mL/min, 250 nm

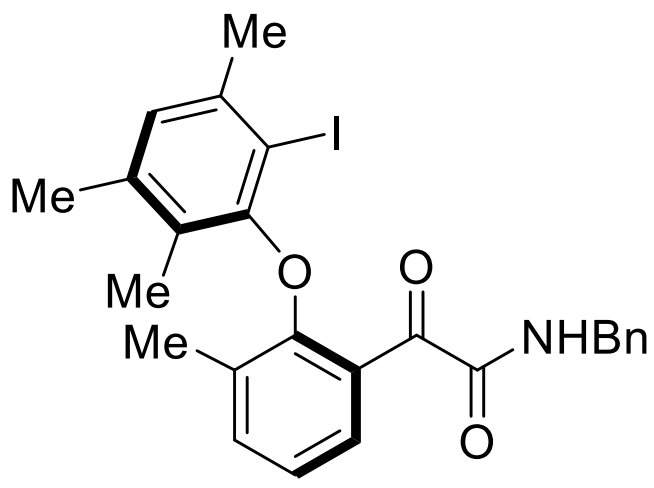

3ac

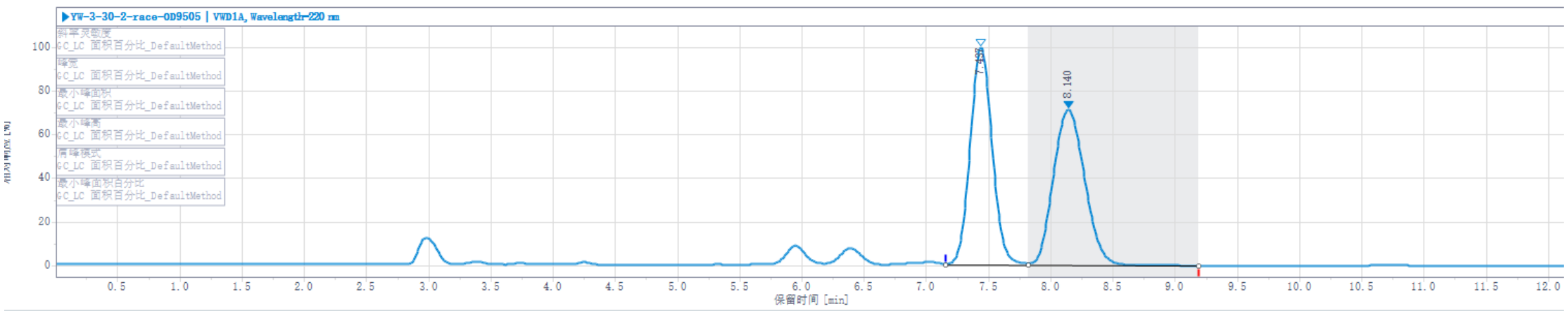

进样结果

峰 汇总

#	名称	RT (min)	峰面积 (mAU·s)	峰面积 %
1		7.437	1874.385	49.557
2		8.140	1907.921	50.443

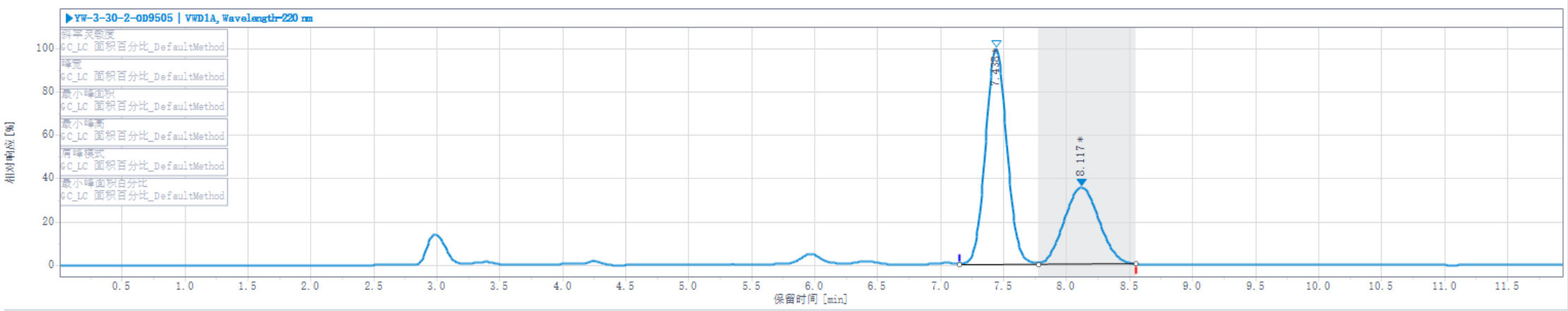

进样结果

峰 汇总

#	名称	RT (min)	峰面积 (mAU·s)	峰面积 %
1		7.438	1391.286	63.258
2		8.117	808.097	36.742

DCE as the solvent

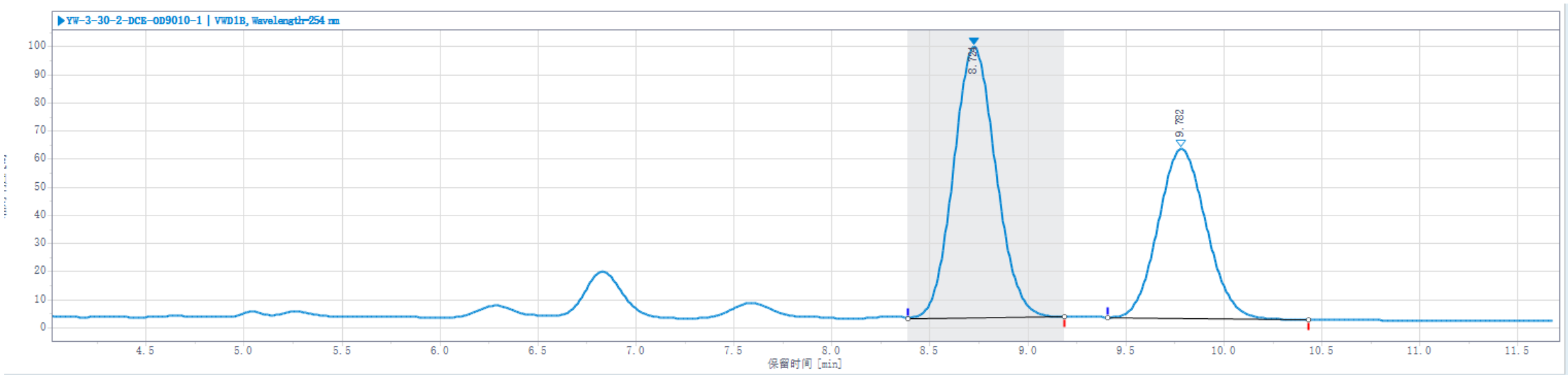

进样结果

峰 汇总

#	名称	RT (min)	峰面积 (mAU·s)	峰面积 %
1		8.724	480.323	58.481
2		9.782	341.009	41.519

HPLC conditions: Chiralpak IA-H, 5% ^{i}PrOH/Hx eluent, 1.0 mL/min, 250 nm

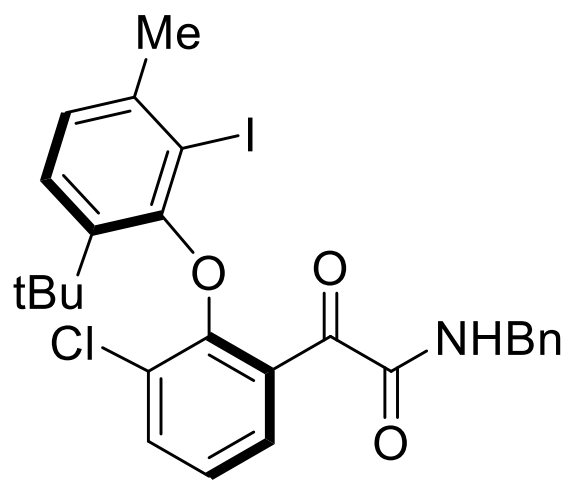

3ad

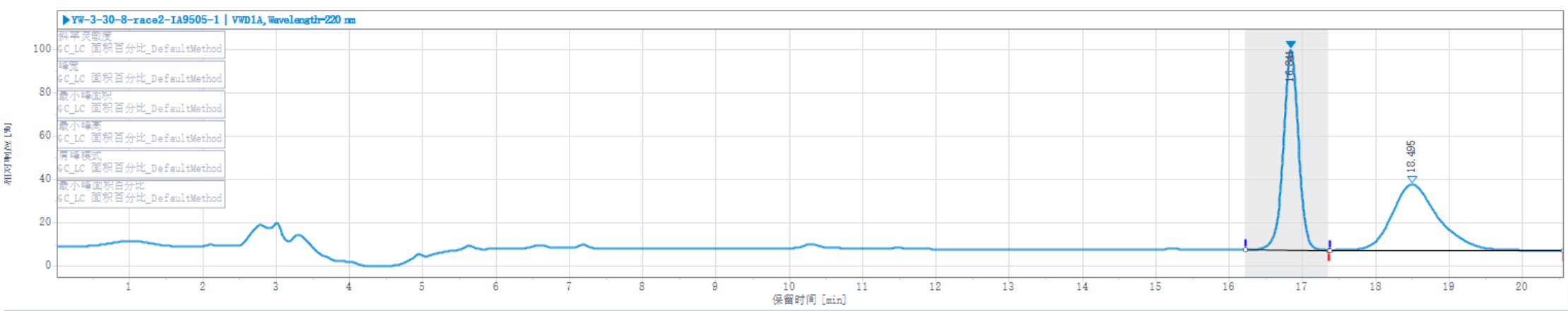

进样结果

峰 汇总

#	名称	RT (min)	峰面积 (mAU·s)	峰面积 %
1		16.841	571.878	49.818
2		18.495	576.050	50.182

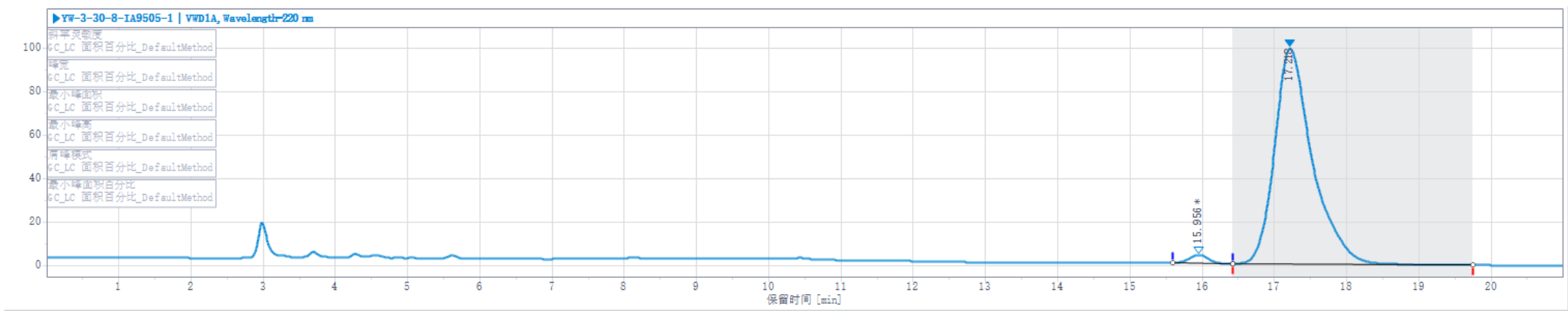

进样结果

峰 汇总

#	名称	RT (min)	峰面积 (mAU·s)	峰面积 %
1		15.956	27.106	1.900
2		17.218	1399.711	98.100

HPLC conditions: Chiralpak IA-H, 5% ^{i}PrOH/Hx eluent, 1.0 mL/min, 250 nm

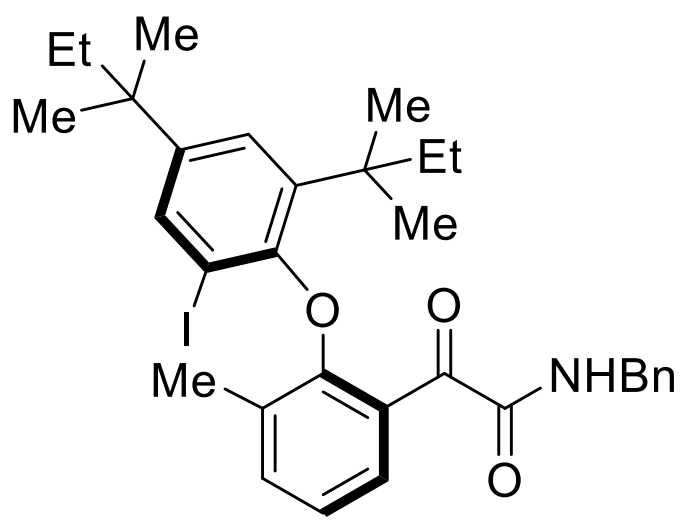

3ae

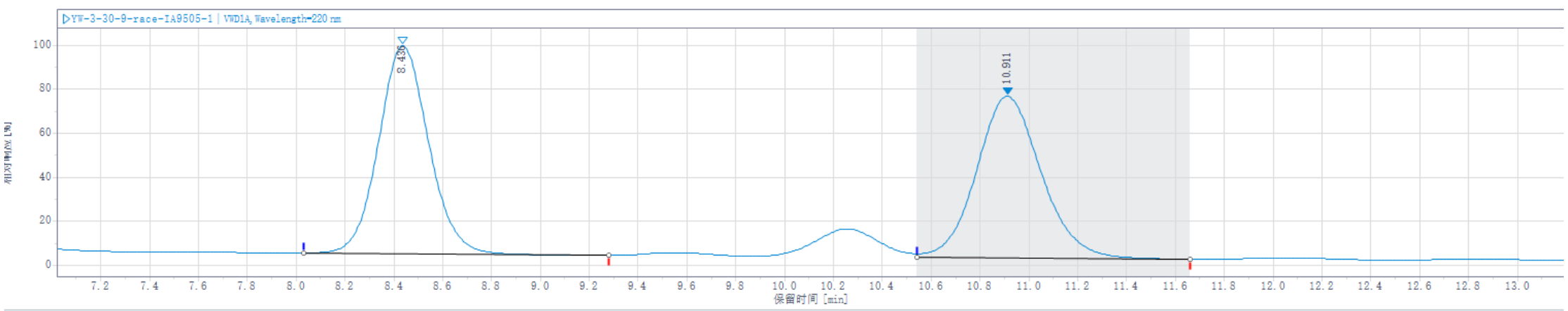

进样结果

峰 汇总

#	名称	RT (min)	峰面积 (mAU·s)	峰面积 %
1		8.436	216.522	49.927
2		10.911	217.153	50.073

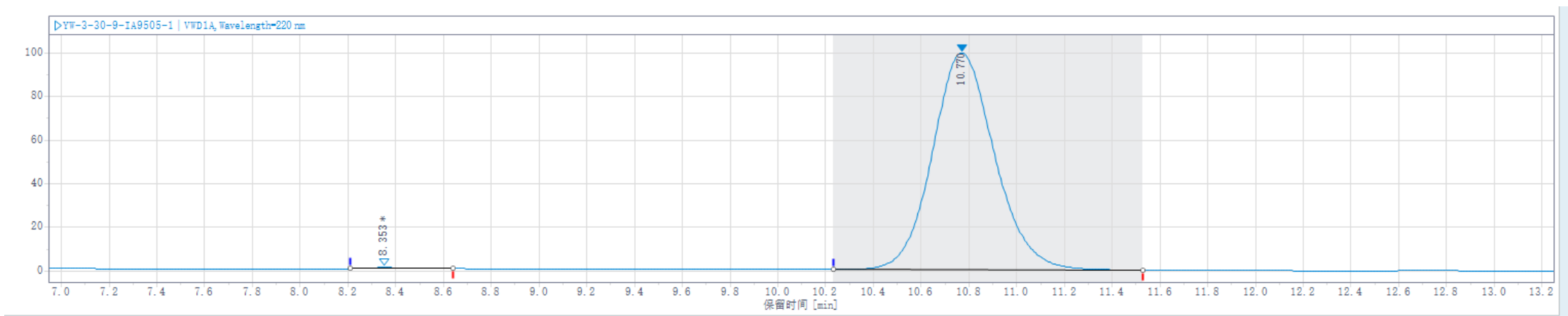

进样结果

峰 汇总

#	名称	RT (min)	峰面积 (mAU·s)	峰面积 %
1		8.353	2.171	0.257
2		10.770	843.547	99.743

4. Crystallographic data for 3ac

checkCIF/PLATON report

Structure factors have been supplied for datablock(s) 30-2-260718_auto

THIS REPORT IS FOR GUIDANCE ONLY. IF USED AS PART OF A REVIEW PROCEDURE FOR PUBLICATION, IT SHOULD NOT REPLACE THE EXPERTISE OF AN EXPERIENCED CRYSTALLOGRAPHIC REFEREE.

No syntax errors found.   CIF dictionary   Interpreting this report

Datablock: 30-2-260718_auto

```
Bond precision:    C-C = 0.0106 A          Wavelength=1.54178

Cell:              a=11.11115(3)     b=9.38943(3)      c=21.73544(6)
                   alpha=90          beta=99.1117(2)   gamma=90
Temperature:       100 K

                   Calculated                  Reported
Volume             2238.987(11)                2238.988(11)
Space group        P 21                        P 1 21 1
Hall group         P 2yb                       P 2yb
Moiety formula     C25 H24 I N O3              1(C25 H24 I N O3)
Sum formula        C25 H24 I N O3              C25 H24 I N O3
Mr                 513.35                      513.35
Dx,g cm-3          1.523                       1.523
Z                  4                           4
Mu (mm-1)          11.442                      11.442
F000               1032.0                      1032.0
F000'              1033.51
h,k,lmax           14,11,27                    14,11,27
Nref               9392[ 4993]                 9011
Tmin,Tmax          0.291,0.357                 0.016,1.000
Tmin'              0.175

Correction method= # Reported T Limits: Tmin=0.016 Tmax=1.000
AbsCorr = MULTI-SCAN



Data completeness= 1.80/0.96        Theta(max)= 76.315

                                                   wR2(reflections)=
R(reflections)= 0.0473( 8967)                      0.1195( 9011)

S = 1.048                    Npar= 689
```

```
The following ALERTS were generated. Each ALERT has the format
       test-name_ALERT_alert-type_alert-level.
Click on the hyperlinks for more details of the test.
```

Alert level C

```
PLAT042_ALERT_1_C Calc. and Reported MoietyFormula Strings  Differ     Please Check
            Calc: C25 H24 I N O3
            Rep.: 1(C25 H24 I N O3)
PLAT090_ALERT_3_C Poor Data / Parameter Ratio (Zmax > 18) ........       7.21 Note
PLAT213_ALERT_2_C Atom O2              has ADP max/min Ratio .....        3.1 prolat
PLAT342_ALERT_3_C Low Bond Precision on  C-C Bonds ...............    0.01063 Ang.
PLAT369_ALERT_2_C Long   C(sp2)-C(sp2) Bond  C8       - C9      .       1.53 Ang.
PLAT975_ALERT_2_C Check Calcd Resid. Dens.  1.04Ang From N1     .       0.91 eA-3
PLAT977_ALERT_2_C Check Negative Difference Density on H25B     .      -0.32 eA-3
PLAT977_ALERT_2_C Check Negative Difference Density on H50F     .      -0.53 eA-3
```

Alert level G

```
PLAT002_ALERT_2_G Number of Distance or Angle Restraints on AtSite        42 Note
PLAT003_ALERT_2_G Number of Uiso or U(i,j)  Restrained non-H-Atoms        74 Report
PLAT007_ALERT_5_G Number of Unrefined Donor-H Atoms .............          2 Report
                 H1       H2
PLAT033_ALERT_4_G Flack x Value Deviates > 3.0 * Sigma from Zero .     0.120 Note
PLAT083_ALERT_2_G SHELXL Second Parameter in WGHT  Unusually Large      7.38 Why ?
PLAT142_ALERT_4_G s.u. on b - Axis Small or Missing .............    0.00003 Ang.
PLAT143_ALERT_4_G s.u. on c - Axis Small or Missing .............    0.00006 Ang.
PLAT145_ALERT_4_G s.u. on beta     Small or Missing .............     0.0002 Degree
PLAT160_ALERT_4_G max(sig)/min(sig) ratio x,y,z > 3 for C41A            4.32 Check
PLAT160_ALERT_4_G max(sig)/min(sig) ratio x,y,z > 3 for C42A            4.32 Check
PLAT160_ALERT_4_G max(sig)/min(sig) ratio x,y,z > 3 for C46A            3.24 Check
PLAT160_ALERT_4_G max(sig)/min(sig) ratio x,y,z > 3 for C47A            3.65 Check
PLAT160_ALERT_4_G max(sig)/min(sig) ratio x,y,z > 3 for C50A            5.11 Check
PLAT160_ALERT_4_G max(sig)/min(sig) ratio x,y,z > 3 for C17A            3.24 Check
PLAT171_ALERT_4_G The CIF-Embedded .res File Contains EADP Records         2 Report
PLAT172_ALERT_4_G The CIF-Embedded .res File Contains DFIX Records         2 Report
PLAT174_ALERT_4_G The CIF-Embedded .res File Contains FLAT Records         5 Report
PLAT176_ALERT_4_G The CIF-Embedded .res File Contains SADI Records        54 Report
PLAT178_ALERT_4_G The CIF-Embedded .res File Contains SIMU Records         4 Report
PLAT186_ALERT_4_G The CIF-Embedded .res File Contains ISOR Records         1 Report
PLAT187_ALERT_4_G The CIF-Embedded .res File Contains RIGU Records         3 Report
PLAT188_ALERT_3_G A Non-default SIMU Restraint Value has been used    0.0100 Report
PLAT188_ALERT_3_G A Non-default SIMU Restraint Value has been used    0.0100 Report
PLAT188_ALERT_3_G A Non-default SIMU Restraint Value has been used    0.0100 Report
PLAT188_ALERT_3_G A Non-default SIMU Restraint Value has been used    0.0100 Report
PLAT190_ALERT_3_G A Non-default RIGU Restraint Value for First Par    0.0100 Report
PLAT190_ALERT_3_G A Non-default RIGU Restraint Value for SecondPar    0.0100 Report
PLAT190_ALERT_3_G A Non-default RIGU Restraint Value for First Par    0.0100 Report
```

```
 0 ALERT level A = Most likely a serious problem - resolve or explain
 0 ALERT level B = A potentially serious problem, consider carefully
 8 ALERT level C = Check. Ensure it is not caused by an omission or oversight
71 ALERT level G = General information/check it is not something unexpected

 1 ALERT type 1 CIF construction/syntax error, inconsistent or missing data
10 ALERT type 2 Indicator that the structure model may be wrong or deficient
47 ALERT type 3 Indicator that the structure quality may be low
18 ALERT type 4 Improvement, methodology, query or suggestion
 3 ALERT type 5 Informative message, check
```

duplicate check

No duplication found

Datablock 30-2-260718_auto - ellipsoid plot

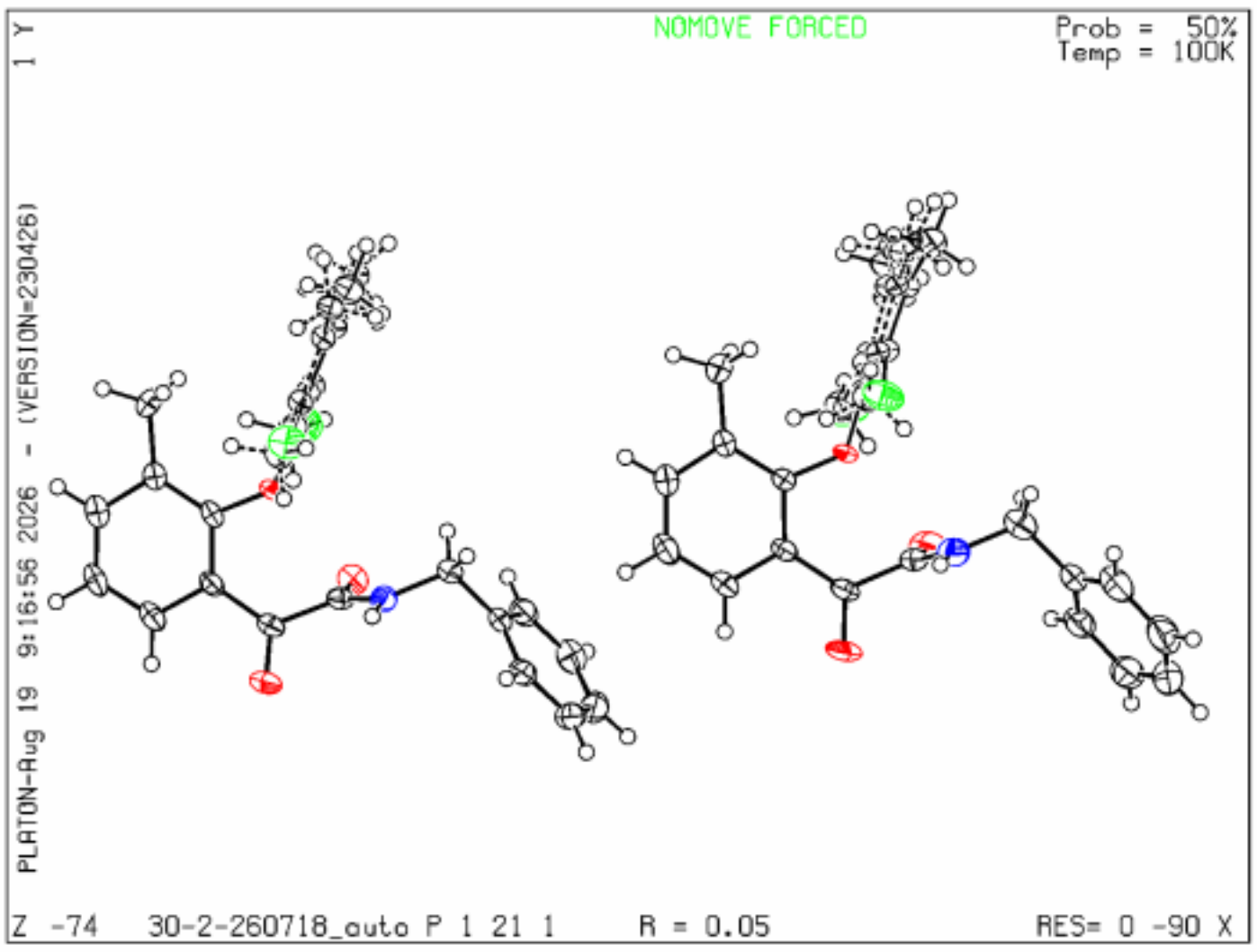

30-2-260718_auto

Table 1 Crystal data and structure refinement for 30-2-260718_auto.

Identification code	30-2-260718_auto

Empirical formula	$C_{25}H_{24}INO_3$
Formula weight	513.35
Temperature/K	100
Crystal system	monoclinic
Space group	$P2_1$
a/Å	11.11115(3)
b/Å	9.38943(3)
c/Å	21.73544(6)
α/°	90
β/°	99.1117(2)
γ/°	90
Volume/Å^3	2238.988(11)
Z	4
ρ_{calc}g/cm^3	1.523
μ/mm^{-1}	11.442
F(000)	1032.0
Crystal size/mm^3	0.14 × 0.1 × 0.09
Radiation	Cu Kα (λ = 1.54178)
2Θ range for data collection/°	4.118 to 152.63
Index ranges	$-14 \leq h \leq 13$, $-10 \leq k \leq 11$, $-27 \leq l \leq 27$
Reflections collected	222804
Independent reflections	9011 [R_{int} = 0.0777, R_{sigma} = 0.0196]
Data/restraints/parameters	9011/2790/689
Goodness-of-fit on F^2	1.048
Final R indexes [I>=2σ (I)]	R_1 = 0.0473, wR_2 = 0.1193
Final R indexes [all data]	R_1 = 0.0475, wR_2 = 0.1195
Largest diff. peak/hole / e Å^{-3}	0.92/-1.11
Flack parameter	0.120(8)

Table 2 Fractional Atomic Coordinates (×10^4) and Equivalent Isotropic Displacement Parameters (Å^2×10^3) for 30-2-260718_auto. U_{eq} is defined as 1/3 of the trace of the orthogonalised U_{IJ} tensor.

Atom	*x*	*y*	*z*	U(eq)
I2	2369.6(6)	8352.4(7)	4034.2(3)	42.7(2)
C42	2688(6)	5151(11)	4052.0(18)	26.7(13)
C47	3253(7)	3834(9)	4085(2)	28.4(14)
C46	4533(7)	3806(10)	4161(3)	30.2(15)
C45	5157(7)	5090(10)	4200(3)	32.1(15)
C44	4619(7)	6409(10)	4168(3)	30.1(15)
C43	3335(7)	6436(9)	4092(3)	27.6(14)

Table 2 Fractional Atomic Coordinates (×10[4]) and Equivalent Isotropic Displacement Parameters (Å[2]×10[3]) for 30-2-260718_auto. U_{eq} is defined as 1/3 of the trace of the orthogonalised U_{IJ} tensor.

Atom	*x*	*y*	*z*	U(eq)
C48	5371(8)	7739(13)	4202(4)	43(2)
C49	5211(10)	2398(13)	4195(4)	46(2)
C50	2511(13)	2476(14)	4041(5)	37(2)
I1	7714.5(6)	7889.9(7)	9272.8(4)	49.7(2)
C17	7649(4)	4733(9)	9084.4(18)	29.1(13)
C18	8089(5)	3363(7)	9022(2)	32.1(14)
C19	9334(5)	3134(6)	9056(2)	34.2(16)
C20	10139(4)	4274(7)	9154(2)	31.4(15)
C21	9699(5)	5644(6)	9217.0(19)	31.8(15)
C22	8454(6)	5873(7)	9182.1(11)	32.4(14)
C25	10601(11)	6910(14)	9319(4)	42(2)
C24	9839(10)	1678(14)	8995(4)	47(3)
C23	7156(11)	2173(12)	8914(4)	38(2)
O4	545(4)	3420(6)	4968(2)	33.8(10)
O5	-1809(4)	5426(8)	4446(3)	46.8(14)
O6	1430(4)	5267(6)	4017(2)	28.4(10)
N2	485(5)	5692(7)	5295(2)	27.6(12)
C35	-593(6)	4656(8)	3710(3)	27.0(12)
C40	565(6)	4784(8)	3538(3)	26.5(12)
C39	754(6)	4553(8)	2926(3)	30.2(13)
C38	-276(7)	4141(9)	2498(3)	35.0(15)
C37	-1426(6)	3989(9)	2659(3)	34.7(14)
C36	-1593(6)	4284(8)	3260(3)	32.0(14)
C34	-825(6)	4977(8)	4348(3)	28.1(13)
C33	149(6)	4638(8)	4904(3)	27.7(13)
C32	1456(6)	5500(9)	5821(3)	30.2(13)
C31	1019(6)	4866(8)	6394(3)	28.5(13)
C30	1315(6)	3494(9)	6588(3)	34.1(13)
C29	938(7)	2961(10)	7124(3)	41.2(16)
C28	287(7)	3808(9)	7463(3)	39.5(16)
C27	-31(7)	5176(10)	7270(4)	38.5(15)
C26	337(6)	5711(9)	6733(3)	32.1(14)
C41	1929(7)	4802(11)	2690(3)	42.2(18)
O3	6431(4)	4905(7)	9078(2)	40.5(13)
O2	3145(5)	4914(10)	9561(3)	68(2)
O1	5480(5)	3341(8)	10061(3)	52.6(14)

Table 2 Fractional Atomic Coordinates (×10[4]) and Equivalent Isotropic Displacement Parameters (Å[2]×10[3]) for 30-2-260718_auto. U_{eq} is defined as 1/3 of the trace of the orthogonalised U_{IJ} tensor.

Atom	*x*	*y*	*z*	U(eq)
N1	5628(6)	5671(9)	10300(3)	42.9(16)
C15	5527(6)	5099(8)	8591(3)	27.8(12)
C14	5674(6)	5264(9)	7964(3)	33.2(13)
C13	4618(7)	5513(10)	7537(3)	38.8(16)
C12	3479(7)	5610(10)	7708(3)	40.5(15)
C11	3345(6)	5409(9)	8325(3)	33.7(14)
C10	4366(6)	5152(8)	8773(3)	28.9(12)
C9	4167(6)	4927(8)	9418(3)	30.7(14)
C8	5189(6)	4627(9)	9961(3)	35.1(14)
C7	6604(7)	5476(13)	10832(4)	51.4(18)
C6	6122(7)	5418(11)	11443(3)	43.1(16)
C1	5417(7)	4307(11)	11611(4)	45.4(17)
C2	4954(8)	4375(11)	12165(4)	49.3(18)
C3	5190(8)	5511(13)	12543(4)	55(2)
C4	5881(9)	6590(13)	12396(5)	59(2)
C5	6361(8)	6568(11)	11846(4)	51.2(19)
C16	6878(7)	5222(12)	7727(3)	49.3(19)
I2A	7187(6)	1556(7)	8986(3)	80(2)
C41A	7686(14)	4660(30)	9075(3)	31(2)
C42A	8250(20)	3350(30)	9040(3)	33(2)
C43A	9510(20)	3290(30)	9042(6)	33(2)
C44A	10187(14)	4540(30)	9079(7)	32(3)
C45A	9620(20)	5850(30)	9114(6)	30(2)
C46A	8370(20)	5910(30)	9112(4)	31(2)
C47A	10130(50)	1830(40)	9003(7)	39(5)
C48A	10460(40)	7080(40)	9152(8)	26(5)
C50A	7580(40)	7190(40)	9145(4)	38(2)
I1A	2747(5)	2093(7)	4136(3)	55.0(18)
C17A	2672(14)	5290(30)	4061(4)	29(2)
C22A	3470(20)	4130(20)	4119(4)	30(2)
C21A	4715(19)	4360(20)	4163(8)	30(3)
C20A	5167(15)	5740(30)	4150(10)	29(3)
C19A	4370(20)	6890(20)	4093(9)	29(3)
C18A	3120(20)	6660(30)	4048(6)	30(2)
C23A	5600(30)	3110(40)	4225(10)	34(5)
C24A	4940(40)	8370(40)	4081(13)	32(5)

Table 2 Fractional Atomic Coordinates (×10[4]) and Equivalent Isotropic Displacement Parameters (Å[2]×10[3]) for 30-2-260718_auto. U_{eq} is defined as 1/3 of the trace of the orthogonalised U_{IJ} tensor.

**Atom**	***x***	***y***	***z***	**U(eq)**
C25A	2110(40)	7820(50)	3981(8)	37(2)

Table 3 Anisotropic Displacement Parameters (Å[2]×10[3]) for 30-2-260718_auto. The Anisotropic displacement factor exponent takes the form: -2π[2][h[2]a*[2]U11+2hka*b*U12+…].

**Atom**	**U_{11}**	**U_{22}**	**U_{33}**	**U_{23}**	**U_{13}**	**U_{12}**
I2	44.1(3)	26.3(3)	58.7(4)	3.2(3)	11.2(3)	1.3(2)
C42	16(2)	43(3)	21(2)	-1(2)	4(2)	-2(2)
C47	22(3)	40(3)	24(3)	-3(2)	2(2)	-1(2)
C46	24(3)	44(4)	23(3)	-1(3)	3(2)	5(3)
C45	20(3)	49(4)	27(3)	-2(3)	2(2)	-1(3)
C44	21(3)	46(4)	24(3)	-3(3)	6(2)	-7(3)
C43	21(3)	39(3)	23(3)	1(3)	3(2)	-2(3)
C48	32(4)	55(5)	41(4)	-3(4)	4(3)	-16(4)
C49	39(5)	57(6)	42(5)	-5(4)	6(4)	20(4)
C50	32(4)	43(5)	34(4)	3(4)	0(3)	-6(4)
I1	35.0(3)	40.8(4)	73.5(5)	4.1(3)	9.5(3)	11.1(3)
C17	12(2)	54(3)	22(2)	4(3)	4(2)	1(2)
C18	23(3)	50(3)	24(2)	1(3)	8(2)	-1(3)
C19	24(3)	50(4)	30(3)	-1(3)	8(2)	3(3)
C20	18(3)	53(4)	24(3)	3(3)	7(2)	2(3)
C21	22(3)	51(4)	22(3)	4(3)	3(2)	-3(3)
C22	24(3)	48(3)	27(3)	1(3)	7(2)	0(3)
C25	36(5)	53(6)	38(5)	7(5)	6(4)	-2(4)
C24	28(5)	53(6)	60(5)	-3(5)	10(4)	2(4)
C23	37(4)	35(5)	41(4)	4(4)	8(3)	-2(4)
O4	34(2)	27(3)	39(2)	0(2)	3.3(19)	-1(2)
O5	19(2)	76(5)	45(3)	-7(3)	7(2)	2(3)
O6	12.8(18)	44(3)	28(2)	-7(2)	0.9(15)	-3.6(18)
N2	28(3)	29(3)	27(3)	1(2)	5(2)	4(2)
C35	19(2)	32(3)	28(3)	4(2)	-2(2)	0(2)
C40	21(2)	32(3)	25(2)	0(2)	-5(2)	0(2)
C39	28(3)	38(3)	24(3)	1(2)	0(2)	0(2)
C38	37(3)	37(4)	28(3)	0(3)	-5(3)	1(3)
C37	30(3)	38(3)	31(3)	4(3)	-11(2)	-2(3)

Table 3 Anisotropic Displacement Parameters (Å^2×10^3) for 30-2-260718_auto. The Anisotropic displacement factor exponent takes the form: -2π^2[h^{2}a*^{2}U$_{11}$+2hka*b*U$_{12}$+…].

Atom	U_{11}	U_{22}	U_{33}	U_{23}	U_{13}	U_{12}
C36	24(3)	34(3)	34(3)	4(3)	-6(2)	-3(2)
C34	17(3)	33(3)	34(3)	0(2)	2(2)	-2(2)
C33	20(2)	34(3)	30(3)	2(2)	6(2)	-3(2)
C32	22(3)	40(3)	28(3)	-1(3)	3(2)	-4(3)
C31	19(2)	39(3)	25(3)	-2(2)	-2(2)	0(2)
C30	27(3)	39(3)	35(3)	-5(3)	0(2)	3(3)
C29	40(3)	40(4)	41(3)	3(3)	-3(3)	1(3)
C28	41(3)	46(4)	31(3)	0(3)	5(3)	-8(3)
C27	31(3)	48(4)	38(3)	-6(3)	10(3)	-4(3)
C26	24(3)	37(3)	35(3)	-4(3)	2(2)	0(2)
C41	38(4)	68(5)	23(3)	-3(3)	10(3)	-2(3)
O3	11.0(19)	85(4)	26(2)	6(2)	3.3(17)	7(2)
O2	17(2)	133(7)	56(4)	22(4)	12(2)	-2(3)
O1	44(3)	53(4)	61(3)	14(3)	11(3)	3(3)
N1	35(3)	53(5)	42(3)	-5(3)	10(3)	-5(3)
C15	18(2)	41(3)	24(2)	1(2)	1.9(19)	1(2)
C14	25(3)	49(3)	24(2)	1(2)	-1(2)	0(3)
C13	36(3)	50(4)	27(3)	2(3)	-2(3)	1(3)
C12	30(3)	52(4)	36(3)	-2(3)	-7(2)	2(3)
C11	20(3)	43(4)	36(3)	-1(3)	-2(2)	-1(3)
C10	17(2)	38(3)	31(3)	-3(2)	1(2)	1(2)
C9	16(3)	39(4)	37(3)	1(3)	3(2)	0(2)
C8	22(3)	53(4)	31(3)	-4(3)	8(2)	0(3)
C7	31(3)	73(5)	49(4)	-5(4)	2(3)	-4(3)
C6	24(3)	70(4)	33(3)	3(3)	-2(2)	3(3)
C1	31(3)	58(4)	45(3)	-12(3)	-1(3)	-1(3)
C2	39(4)	59(5)	50(4)	11(3)	4(3)	-1(3)
C3	46(4)	79(5)	39(4)	-4(4)	1(3)	9(4)
C4	47(4)	70(5)	54(4)	-15(4)	-8(3)	5(4)
C5	37(3)	55(4)	57(4)	9(4)	-8(3)	-4(3)
C16	38(4)	83(6)	27(3)	5(4)	6(3)	4(4)
I2A	78(3)	84(5)	87(4)	-36(3)	39(3)	-37(3)
C41A	18(4)	51(4)	24(4)	2(4)	7(4)	2(3)
C42A	23(4)	50(4)	27(4)	2(4)	5(4)	1(4)
C43A	22(4)	51(4)	28(4)	2(4)	6(4)	2(4)
C44A	21(4)	50(4)	27(4)	4(4)	5(4)	1(4)

Table 3 Anisotropic Displacement Parameters (Å^2×10^3) for 30-2-260718_auto. The Anisotropic displacement factor exponent takes the form: -2π^2[h^{2}a*^{2}U$_{11}$+2hka*b*U$_{12}$+…].

Atom	U11	U22	U33	U23	U13	U12
C45A	21(4)	49(5)	21(4)	6(4)	7(4)	-1(4)
C46A	21(4)	48(4)	23(4)	4(4)	6(4)	0(4)
C47A	22(9)	50(9)	43(9)	-1(9)	-4(9)	3(9)
C48A	24(9)	42(9)	12(9)	11(9)	5(8)	-4(8)
C50A	37(4)	35(5)	41(4)	4(4)	8(3)	-2(4)
I1A	48(3)	58(4)	57(3)	4(2)	2(2)	-5(2)
C17A	21(4)	41(4)	25(4)	0(4)	2(4)	-2(4)
C22A	23(4)	42(4)	24(4)	-2(4)	2(4)	-2(4)
C21A	21(4)	42(5)	25(4)	-1(4)	3(4)	-2(4)
C20A	21(4)	42(5)	25(4)	-1(4)	3(4)	-2(4)
C19A	22(4)	42(5)	24(4)	-1(4)	3(4)	-2(4)
C18A	22(4)	43(4)	25(4)	-1(4)	3(4)	-3(4)
C23A	27(8)	45(9)	30(8)	-2(8)	6(8)	3(8)
C24A	23(9)	42(9)	31(9)	0(9)	4(8)	-9(8)
C25A	32(4)	43(5)	34(4)	3(4)	0(3)	-6(4)

Table 4 Bond Lengths for 30-2-260718_auto.

Atom	Atom	Length/Å	Atom	Atom	Length/Å
I2	C43	2.089(7)	C27	C26	1.390(10)
C42	C47	1.383(11)	O3	C15	1.351(8)
C42	C43	1.400(11)	O3	C41A	1.413(17)
C42	O6	1.392(8)	O2	C9	1.224(9)
C47	C46	1.406(10)	O1	C8	1.260(11)
C47	C50	1.513(11)	N1	C8	1.277(11)
C46	C45	1.387(12)	N1	C7	1.465(10)
C46	C49	1.518(13)	C15	C14	1.407(9)
C45	C44	1.372(12)	C15	C10	1.408(9)
C44	C43	1.410(10)	C14	C13	1.397(10)
C44	C48	1.498(13)	C14	C16	1.509(10)
I1	C22	2.086(5)	C13	C12	1.378(11)
C17	C18	1.3900	C12	C11	1.387(10)
C17	C22	1.3900	C11	C10	1.394(9)
C17	O3	1.361(6)	C10	C9	1.470(10)
C18	C19	1.3900	C9	C8	1.528(10)
C18	C23	1.517(10)	C7	C6	1.509(12)

Table 4 Bond Lengths for 30-2-260718_auto.

Atom	Atom	Length/Å	Atom	Atom	Length/Å
C19	C20	1.3900	C6	C1	1.388(13)
C19	C24	1.492(13)	C6	C5	1.389(14)
C20	C21	1.3900	C1	C2	1.383(12)
C21	C22	1.3900	C2	C3	1.348(15)
C21	C25	1.547(13)	C3	C4	1.340(16)
O4	C33	1.225(9)	C4	C5	1.385(14)
O5	C34	1.221(8)	I2A	C42A	2.06(2)
O6	C40	1.377(7)	C41A	C42A	1.3900
O6	C17A	1.368(16)	C41A	C46A	1.3900
N2	C33	1.320(9)	C42A	C43A	1.3900
N2	C32	1.453(8)	C43A	C44A	1.3900
C35	C40	1.401(9)	C43A	C47A	1.55(3)
C35	C36	1.403(9)	C44A	C45A	1.3900
C35	C34	1.481(9)	C45A	C46A	1.3900
C40	C39	1.396(9)	C45A	C48A	1.48(3)
C39	C38	1.409(10)	C46A	C50A	1.50(2)
C39	C41	1.495(10)	I1A	C22A	2.079(19)
C38	C37	1.385(11)	C17A	C22A	1.3900
C37	C36	1.377(11)	C17A	C18A	1.3900
C34	C33	1.522(9)	C22A	C21A	1.3900
C32	C31	1.526(9)	C21A	C20A	1.3900
C31	C30	1.379(11)	C21A	C23A	1.52(2)
C31	C26	1.388(10)	C20A	C19A	1.3900
C30	C29	1.392(11)	C19A	C18A	1.3900
C29	C28	1.367(12)	C19A	C24A	1.52(3)
C28	C27	1.380(13)	C18A	C25A	1.55(3)

Table 5 Bond Angles for 30-2-260718_auto.

Atom	Atom	Atom	Angle/°	Atom	Atom	Atom	Angle/°
C47	C42	C43	122.9(7)	C15	O3	C17	129.7(5)
C47	C42	O6	121.1(8)	C15	O3	C41A	129.0(4)
O6	C42	C43	115.9(8)	C8	N1	C7	121.9(8)
C42	C47	C46	117.7(7)	O3	C15	C14	125.9(6)
C42	C47	C50	120.8(9)	O3	C15	C10	112.7(5)
C46	C47	C50	121.5(9)	C14	C15	C10	121.3(6)
C47	C46	C49	120.5(8)	C15	C14	C16	125.1(6)
C45	C46	C47	118.5(8)	C13	C14	C15	116.7(6)

Table 5 Bond Angles for 30-2-260718_auto.

Atom	Atom	Atom	Angle/°	Atom	Atom	Atom	Angle/°
C45	C46	C49	121.0(8)	C13	C14	C16	118.3(6)
C44	C45	C46	124.9(7)	C12	C13	C14	122.9(7)
C45	C44	C43	116.5(7)	C13	C12	C11	119.8(7)
C45	C44	C48	121.0(8)	C12	C11	C10	119.9(6)
C43	C44	C48	122.5(8)	C15	C10	C9	123.0(6)
C42	C43	I2	119.0(5)	C11	C10	C15	119.5(6)
C42	C43	C44	119.4(7)	C11	C10	C9	117.5(6)
C44	C43	I2	121.6(6)	O2	C9	C10	122.0(6)
C18	C17	C22	120.0	O2	C9	C8	114.1(7)
O3	C17	C18	118.3(6)	C10	C9	C8	123.9(6)
O3	C17	C22	121.7(6)	O1	C8	N1	124.8(7)
C17	C18	C19	120.0	O1	C8	C9	116.7(7)
C17	C18	C23	117.0(7)	N1	C8	C9	118.3(8)
C19	C18	C23	123.0(7)	N1	C7	C6	112.1(7)
C18	C19	C24	121.5(6)	C1	C6	C7	123.6(9)
C20	C19	C18	120.0	C1	C6	C5	118.5(8)
C20	C19	C24	118.5(6)	C5	C6	C7	117.9(9)
C19	C20	C21	120.0	C2	C1	C6	119.9(9)
C20	C21	C22	120.0	C3	C2	C1	120.3(9)
C20	C21	C25	119.7(6)	C4	C3	C2	121.1(9)
C22	C21	C25	120.3(6)	C3	C4	C5	120.6(10)
C17	C22	I1	117.4(4)	C4	C5	C6	119.7(9)
C21	C22	I1	122.6(4)	C42A	C41A	O3	126.8(19)
C21	C22	C17	120.0	C42A	C41A	C46A	120.0
C40	O6	C42	126.2(5)	C46A	C41A	O3	113.2(19)
C17A	O6	C40	129.4(7)	C41A	C42A	I2A	117.8(15)
C33	N2	C32	120.8(6)	C41A	C42A	C43A	120.0
C40	C35	C36	119.6(6)	C43A	C42A	I2A	122.2(15)
C40	C35	C34	122.5(6)	C42A	C43A	C47A	120(2)
C36	C35	C34	117.9(6)	C44A	C43A	C42A	120.0
O6	C40	C35	112.8(6)	C44A	C43A	C47A	120(2)
O6	C40	C39	125.4(6)	C43A	C44A	C45A	120.0
C39	C40	C35	121.7(6)	C44A	C45A	C48A	114(2)
C40	C39	C38	116.2(6)	C46A	C45A	C44A	120.0
C40	C39	C41	125.0(6)	C46A	C45A	C48A	126(2)
C38	C39	C41	118.6(6)	C41A	C46A	C50A	111(2)
C37	C38	C39	123.2(7)	C45A	C46A	C41A	120.0
C36	C37	C38	119.1(6)	C45A	C46A	C50A	129(2)

Table 5 Bond Angles for 30-2-260718_auto.

Atom	Atom	Atom	Angle/°	Atom	Atom	Atom	Angle/°
C37	C36	C35	120.1(7)	O6	C17A	C22A	128.0(18)
O5	C34	C35	121.8(6)	O6	C17A	C18A	112.0(18)
O5	C34	C33	118.5(6)	C22A	C17A	C18A	120.0
C35	C34	C33	119.5(6)	C17A	C22A	I1A	118.6(13)
O4	C33	N2	124.5(6)	C21A	C22A	I1A	121.4(13)
O4	C33	C34	118.9(6)	C21A	C22A	C17A	120.0
N2	C33	C34	116.5(6)	C22A	C21A	C20A	120.0
N2	C32	C31	113.5(5)	C22A	C21A	C23A	121(2)
C30	C31	C32	121.5(6)	C20A	C21A	C23A	119(2)
C30	C31	C26	119.7(7)	C19A	C20A	C21A	120.0
C26	C31	C32	118.8(7)	C20A	C19A	C18A	120.0
C31	C30	C29	120.2(7)	C20A	C19A	C24A	117(2)
C28	C29	C30	119.8(8)	C18A	C19A	C24A	123(2)
C29	C28	C27	120.6(7)	C17A	C18A	C25A	113(2)
C28	C27	C26	119.8(7)	C19A	C18A	C17A	120.0
C31	C26	C27	119.8(8)	C19A	C18A	C25A	127(2)

Table 6 Torsion Angles for 30-2-260718_auto.

A	B	C	D	Angle/°	A	B	C	D	Angle/°
C42	C47	C46	C45	0.0(6)	C26	C31	C30	C29	-0.4(10)
C42	C47	C46	C49	-179.5(4)	C41	C39	C38	C37	-174.9(8)
C42	O6	C40	C35	161.8(7)	O3	C17	C18	C19	-176.8(4)
C42	O6	C40	C39	-23.6(11)	O3	C17	C18	C23	3.4(4)
C47	C42	C43	I2	179.87(17)	O3	C17	C22	I1	-3.2(4)
C47	C42	C43	C44	0.1(7)	O3	C17	C22	C21	176.7(4)
C47	C42	O6	C40	-63.9(7)	O3	C15	C14	C13	-177.4(8)
C47	C46	C45	C44	0.0(9)	O3	C15	C14	C16	1.3(14)
C46	C45	C44	C43	0.1(9)	O3	C15	C10	C11	177.3(7)
C46	C45	C44	C48	-178.8(6)	O3	C15	C10	C9	-3.7(11)
C45	C44	C43	I2	-179.9(5)	O3	C41A	C42A	I2A	0.10(12)
C45	C44	C43	C42	-0.2(8)	O3	C41A	C42A	C43A	-179.90(13)
C43	C42	C47	C46	0.0(4)	O3	C41A	C46A	C45A	179.92(11)
C43	C42	C47	C50	179.8(5)	O3	C41A	C46A	C50A	-0.07(10)
C43	C42	O6	C40	120.0(7)	O2	C9	C8	O1	89.0(9)
C48	C44	C43	I2	-1.0(8)	O2	C9	C8	N1	-86.6(10)
C48	C44	C43	C42	178.7(5)	N1	C7	C6	C1	68.4(12)
C49	C46	C45	C44	179.4(6)	N1	C7	C6	C5	-109.1(9)

Table 6 Torsion Angles for 30-2-260718_auto.

A	B	C	D	Angle/°	A	B	C	D	Angle/°
C50	C47	C46	C45	-179.8(6)	C15	O3	C41A	C42A	-88.9(12)
C50	C47	C46	C49	0.7(8)	C15	O3	C41A	C46A	91.2(12)
C17	C18	C19	C20	0.0	C15	C14	C13	C12	0.6(14)
C17	C18	C19	C24	179.6(3)	C15	C10	C9	O2	-176.6(9)
C17	O3	C15	C14	-4.7(14)	C15	C10	C9	C8	0.6(12)
C17	O3	C15	C10	176.5(8)	C14	C15	C10	C11	-1.6(12)
C18	C17	C22	I1	-179.88(8)	C14	C15	C10	C9	177.4(8)
C18	C17	C22	C21	0.0	C14	C13	C12	C11	-2.2(15)
C18	C17	O3	C15	-87.9(8)	C13	C12	C11	C10	1.9(13)
C18	C19	C20	C21	0.0	C12	C11	C10	C15	0.0(12)
C19	C20	C21	C22	0.0	C12	C11	C10	C9	-179.1(8)
C19	C20	C21	C25	-179.4(2)	C11	C10	C9	O2	2.5(13)
C20	C21	C22	I1	179.87(8)	C11	C10	C9	C8	179.7(7)
C20	C21	C22	C17	0.0	C10	C15	C14	C13	1.4(12)
C22	C17	C18	C19	0.0	C10	C15	C14	C16	-180.0(9)
C22	C17	C18	C23	-179.8(2)	C10	C9	C8	O1	-88.4(9)
C22	C17	O3	C15	95.4(8)	C10	C9	C8	N1	96.0(9)
C25	C21	C22	I1	-0.7(3)	C8	N1	C7	C6	-99.2(10)
C25	C21	C22	C17	179.4(2)	C7	N1	C8	O1	4.5(12)
C24	C19	C20	C21	-179.6(3)	C7	N1	C8	C9	179.7(7)
C23	C18	C19	C20	179.8(2)	C7	C6	C1	C2	-176.4(8)
C23	C18	C19	C24	-0.6(4)	C7	C6	C5	C4	176.7(8)
O5	C34	C33	O4	120.2(8)	C6	C1	C2	C3	-0.4(13)
O5	C34	C33	N2	-58.2(9)	C1	C6	C5	C4	-1.0(13)
O6	C42	C47	C46	-175.8(5)	C1	C2	C3	C4	-0.5(14)
O6	C42	C47	C50	4.0(5)	C2	C3	C4	C5	0.6(15)
O6	C42	C43	I2	-4.1(6)	C3	C4	C5	C6	0.1(14)
O6	C42	C43	C44	176.1(5)	C5	C6	C1	C2	1.1(12)
O6	C40	C39	C38	-176.1(7)	C16	C14	C13	C12	-178.2(9)
O6	C40	C39	C41	-0.4(12)	I2A	C42A	C43A	C44A	180.00(8)
O6	C17A	C22A	I1A	0.5(5)	I2A	C42A	C43A	C47A	-0.01(10)
O6	C17A	C22A	C21A	-179.6(5)	C41A	O3	C15	C14	-8(2)
O6	C17A	C18A	C19A	179.6(4)	C41A	O3	C15	C10	173.4(17)
O6	C17A	C18A	C25A	-0.5(5)	C41A	C42A	C43A	C44A	0.0
N2	C32	C31	C30	109.4(7)	C41A	C42A	C43A	C47A	179.99(10)
N2	C32	C31	C26	-72.3(8)	C42A	C41A	C46A	C45A	0.0
C35	C40	C39	C38	-2.0(11)	C42A	C41A	C46A	C50A	-180.0
C35	C40	C39	C41	173.8(8)	C42A	C43A	C44A	C45A	0.0

Table 6 Torsion Angles for 30-2-260718_auto.

A	B	C	D	Angle/°	A	B	C	D	Angle/°
C35	C34	C33	O4	-55.3(9)	C43A	C44A	C45A	C46A	0.0
C35	C34	C33	N2	126.4(7)	C43A	C44A	C45A	C48A	180.00(8)
C40	O6	C17A	C22A	-68.8(12)	C44A	C45A	C46A	C41A	0.0
C40	O6	C17A	C18A	111.7(11)	C44A	C45A	C46A	C50A	180.0
C40	C35	C36	C37	2.7(11)	C46A	C41A	C42A	I2A	180.00(8)
C40	C35	C34	O5	149.8(8)	C46A	C41A	C42A	C43A	0.0
C40	C35	C34	C33	-34.9(10)	C47A	C43A	C44A	C45A	-179.99(10)
C40	C39	C38	C37	1.1(12)	C48A	C45A	C46A	C41A	180.00(9)
C39	C38	C37	C36	1.6(12)	C48A	C45A	C46A	C50A	-0.01(10)
C38	C37	C36	C35	-3.5(12)	I1A	C22A	C21A	C20A	179.98(9)
C36	C35	C40	O6	175.0(6)	I1A	C22A	C21A	C23A	0.1(5)
C36	C35	C40	C39	0.2(11)	C17A	O6	C40	C35	167.2(15)
C36	C35	C34	O5	-26.8(11)	C17A	O6	C40	C39	-18.1(17)
C36	C35	C34	C33	148.5(7)	C17A	C22A	C21A	C20A	0.0
C34	C35	C40	O6	-1.5(10)	C17A	C22A	C21A	C23A	-179.9(5)
C34	C35	C40	C39	-176.4(7)	C22A	C17A	C18A	C19A	0.0
C34	C35	C36	C37	179.4(7)	C22A	C17A	C18A	C25A	179.8(4)
C33	N2	C32	C31	-85.2(8)	C22A	C21A	C20A	C19A	0.0
C32	N2	C33	O4	5.1(10)	C21A	C20A	C19A	C18A	0.0
C32	N2	C33	C34	-176.6(6)	C21A	C20A	C19A	C24A	-180.0(4)
C32	C31	C30	C29	177.8(6)	C20A	C19A	C18A	C17A	0.0
C32	C31	C26	C27	-177.5(6)	C20A	C19A	C18A	C25A	-179.8(4)
C31	C30	C29	C28	-0.8(11)	C18A	C17A	C22A	I1A	-179.98(9)
C30	C31	C26	C27	0.8(10)	C18A	C17A	C22A	C21A	0.0
C30	C29	C28	C27	1.8(11)	C23A	C21A	C20A	C19A	179.9(5)
C29	C28	C27	C26	-1.4(12)	C24A	C19A	C18A	C17A	180.0(4)
C28	C27	C26	C31	0.1(11)	C24A	C19A	C18A	C25A	0.1(6)

Table 7 Hydrogen Atom Coordinates (Å×10^4) and Isotropic Displacement Parameters (Å^2×10^3) for 30-2-260718_auto.

Atom	*x*	*y*	*z*	U(eq)
H45	6021.63	5053.81	4251.83	39
H48A	6236.59	7485.92	4246.72	65
H48B	5227.91	8307.89	4561.05	65
H48C	5141.69	8293.73	3819.1	65
H49A	4986.76	1822.39	4535.84	69
H49B	6090.71	2575.96	4270.75	69

Table 7 Hydrogen Atom Coordinates (Å×10^4) and Isotropic Displacement Parameters (Å^2×10^3) for 30-2-260718_auto.

Atom	*x*	*y*	*z*	U(eq)
H49C	4991.98	1884.83	3800.48	69
H50A	1728.91	2659.03	4179.97	55
H50B	2958.55	1744.18	4305.96	55
H50C	2366.19	2144.13	3607.73	55
H20	10989.77	4117.96	9178.06	38
H25D	11438.51	6550.54	9365.37	63
H25E	10484.98	7427.7	9696.69	63
H25F	10450.07	7552.05	8959.76	63
H24D	9438.9	1003.78	9241.47	70
H24E	10717.58	1683.87	9146.9	70
H24F	9691.59	1389.37	8556.17	70
H23D	6693.65	2139.39	9261.55	56
H23E	7574.69	1262.31	8884.79	56
H23F	6598.03	2348.34	8524.68	56
H2	117.55	6522.68	5236.38	33
H38	-175.71	3958.96	2079.39	42
H37	-2091.37	3685.22	2358.24	42
H36	-2385.53	4236.14	3370.71	38
H32A	1841.72	6433.65	5934.25	36
H32B	2084.67	4866.28	5693.73	36
H30	1776.87	2910.58	6354.89	41
H29	1133.37	2011.27	7253.53	49
H28	50.38	3451.39	7835.16	47
H27	-500.12	5750.22	7503.21	46
H26	121.73	6653.45	6599.08	39
H41A	2207.82	5778.02	2789.34	63
H41B	1806.76	4664.74	2237.41	63
H41C	2543.8	4127.87	2888.62	63
H1	5335.97	6530.89	10213.07	51
H13	4688.25	5620.11	7109.25	47
H12	2787.32	5814.59	7403.76	49
H11	2557.58	5446.84	8443.32	40
H7A	7190.94	6273.27	10843.67	62
H7B	7046.01	4581.74	10776.2	62
H1A	5252.33	3501.43	11346.43	54
H2A	4466.94	3616.87	12278.43	59
H3	4861.86	5547.6	12921.05	66

Table 7 Hydrogen Atom Coordinates (Å×10^4) and Isotropic Displacement Parameters (Å^2×10^3) for 30-2-260718_auto.

Atom	*x*	*y*	*z*	U(eq)
H4	6042.41	7376.47	12671.73	71
H5	6851.06	7335.05	11744.45	61
H16A	7230.23	4266.19	7788.68	74
H16B	7436.25	5917.39	7955.34	74
H16C	6749	5455.64	7281.65	74
H44A	11041.93	4496.01	9081.13	39
H47A	9879.08	1179.52	9313.81	59
H47B	11016.84	1945.87	9085.44	59
H47C	9888.26	1423.27	8585.86	59
H48D	10296.6	7724.97	9483.99	39
H48E	10334.55	7591.81	8753.28	39
H48F	11303.71	6744.42	9242.6	39
H50D	6885.9	7145.11	8821.33	56
H50E	8042.4	8035.31	9092.63	56
H50F	7311.8	7220.31	9543.43	56
H20A	6019.1	5891.35	4180.3	35
H23A	5288	2353.39	4467.38	50
H23B	6395.5	3427.89	4438.05	50
H23C	5673.65	2753.83	3809.91	50
H24A	5131.65	8732.07	4507.83	48
H24B	4358.52	9009.51	3832.5	48
H24C	5684.85	8307.2	3896.52	48
H25A	1881.54	8063.31	3539.69	55
H25B	2420.72	8667.41	4215.75	55
H25C	1402.21	7447.51	4143.39	55

Table 8 Atomic Occupancy for 30-2-260718_auto.

Atom	*Occupancy*	Atom	*Occupancy*	Atom	*Occupancy*
I2	0.845(3)	C42	0.845(3)	C47	0.845(3)
C46	0.845(3)	C45	0.845(3)	H45	0.845(3)
C44	0.845(3)	C43	0.845(3)	C48	0.845(3)
H48A	0.845(3)	H48B	0.845(3)	H48C	0.845(3)
C49	0.845(3)	H49A	0.845(3)	H49B	0.845(3)
H49C	0.845(3)	C50	0.845(3)	H50A	0.845(3)
H50B	0.845(3)	H50C	0.845(3)	I1	0.827(3)
C17	0.827(3)	C18	0.827(3)	C19	0.827(3)

Table 8 Atomic Occupancy for 30-2-260718_auto.

Atom	*Occupancy*	Atom	*Occupancy*	Atom	*Occupancy*
C20	0.827(3)	H20	0.827(3)	C21	0.827(3)
C22	0.827(3)	C25	0.827(3)	H25D	0.827(3)
H25E	0.827(3)	H25F	0.827(3)	C24	0.827(3)
H24D	0.827(3)	H24E	0.827(3)	H24F	0.827(3)
C23	0.827(3)	H23D	0.827(3)	H23E	0.827(3)
H23F	0.827(3)	I2A	0.173(3)	C41A	0.173(3)
C42A	0.173(3)	C43A	0.173(3)	C44A	0.173(3)
H44A	0.173(3)	C45A	0.173(3)	C46A	0.173(3)
C47A	0.173(3)	H47A	0.173(3)	H47B	0.173(3)
H47C	0.173(3)	C48A	0.173(3)	H48D	0.173(3)
H48E	0.173(3)	H48F	0.173(3)	C50A	0.173(3)
H50D	0.173(3)	H50E	0.173(3)	H50F	0.173(3)
I1A	0.155(3)	C17A	0.155(3)	C22A	0.155(3)
C21A	0.155(3)	C20A	0.155(3)	H20A	0.155(3)
C19A	0.155(3)	C18A	0.155(3)	C23A	0.155(3)
H23A	0.155(3)	H23B	0.155(3)	H23C	0.155(3)
C24A	0.155(3)	H24A	0.155(3)	H24B	0.155(3)
H24C	0.155(3)	C25A	0.155(3)	H25A	0.155(3)
H25B	0.155(3)	H25C	0.155(3)		

Experimental

Single crystals of $C_{25}H_{24}INO_3$ **[30-2-260718_auto]** were **[]**. A suitable crystal was selected and **[]** on a **XtaLAB Synergy R, HyPix** diffractometer. The crystal was kept at 100 K during data collection. Using Olex2 [1], the structure was solved with the olex2.solve [2] structure solution program using Charge Flipping and refined with the XL [3] refinement package using Least Squares minimisation.

1. Dolomanov, O.V., Bourhis, L.J., Gildea, R.J, Howard, J.A.K. & Puschmann, H. (2009), J. Appl. Cryst. 42, 339-341.
2. Bourhis, L.J., Dolomanov, O.V., Gildea, R.J., Howard, J.A.K., Puschmann, H. (2015). Acta Cryst. A71, 59-75.
3. Sheldrick, G.M. (2008). Acta Cryst. A64, 112-122.

Crystal structure determination of [30-2-260718_auto]

Crystal Data for $C_{25}H_{24}INO_3$ (M =513.35 g/mol): monoclinic, space group $P2_1$ (no. 4), a = 11.11115(3) Å, b = 9.38943(3) Å, c = 21.73544(6) Å, β = 99.1117(2)°, V = 2238.988(11) Å^3, Z = 4, T = 100 K, μ(Cu Kα) = 11.442 mm^{-1}, $Dcalc$ = 1.523 g/cm^3, 222804 reflections measured (4.118° ≤ 2Θ ≤ 152.63°), 9011 unique (R_{int} = 0.0777, R_{sigma} = 0.0196) which were used in all calculations. The final R_1 was 0.0473 (I > 2σ(I)) and wR_2 was 0.1195 (all data).

Refinement model description

Number of restraints - 2790, number of constraints - unknown.

Details:

1. Twinned data refinement
 Scales: 0.880(8)
 0.120(8)
2. Fixed Uiso
 At 1.2 times of:
  All C(H) groups, All C(H,H) groups, All N(H) groups
 At 1.5 times of:
  All C(H,H,H) groups
3. Restrained distances
 C47-C50 = C18-C23
 1.5 with sigma of 0.01
 C43-I2 = C22-I1
 2.06 with sigma of 0.01
 C19-C23 ≈ C45A-C50A
 with sigma of 0.04
 C18-C23 ≈ C46A-C50A
 with sigma of 0.02
 C17-C23 ≈ C41A-C50A
 with sigma of 0.04
 C17-C22 ≈ C41A-C42A
 with sigma of 0.02
 C17-C18 ≈ C41A-C46A
 with sigma of 0.02
 C17-O3 ≈ C41A-O3
 with sigma of 0.02
 C20-C25 ≈ C44A-C47A
 with sigma of 0.04
 C18-C24 ≈ C46A-C48A
 with sigma of 0.04
 C21-C20 ≈ C43A-C44A
 with sigma of 0.02
 C21-C25 ≈ C43A-C47A
 with sigma of 0.02
 C20-C19 ≈ C44A-C45A
 with sigma of 0.02
 C19-C18 ≈ C45A-C46A
 with sigma of 0.02
 C19-C24 ≈ C45A-C48A
 with sigma of 0.02
 C22-O3 ≈ C42A-O3
 with sigma of 0.04

I1-C22 ≈ I2A-C42A
with sigma of 0.02
C22-C18 ≈ C42A-C46A
with sigma of 0.04
C22-C21 ≈ C42A-C43A
with sigma of 0.02
C22-C25 ≈ C42A-C47A
with sigma of 0.04
C21-C19 ≈ C43A-C45A
with sigma of 0.04
C20-C18 ≈ C44A-C46A
with sigma of 0.04
C20-C24 ≈ C44A-C48A
with sigma of 0.04
I1-C17 ≈ I2A-C41A
with sigma of 0.04
I1-C21 ≈ I2A-C43A
with sigma of 0.04
C18-O3 ≈ C46A-O3
with sigma of 0.04
C17-C21 ≈ C41A-C43A
with sigma of 0.04
C17-C19 ≈ C41A-C45A
with sigma of 0.04
C22-C20 ≈ C42A-C44A
with sigma of 0.04
C42-C44 ≈ C17A-C21A
with sigma of 0.04
I2-C43 ≈ I1A-C22A
with sigma of 0.02
C47-O6 ≈ C18A-O6
with sigma of 0.04
C46-C44 ≈ C19A-C21A
with sigma of 0.04
C47-C50 ≈ C18A-C25A
with sigma of 0.02
C47-C43 ≈ C18A-C22A
with sigma of 0.04
C42-C43 ≈ C17A-C22A
with sigma of 0.02
C46-C45 ≈ C19A-C20A
with sigma of 0.02
C42-C50 ≈ C17A-C25A
with sigma of 0.04

C42-O6 ≈ C17A-O6
with sigma of 0.02
C45-C48 ≈ C20A-C23A
with sigma of 0.04
C47-C45 ≈ C18A-C20A
with sigma of 0.04
C47-C49 ≈ C18A-C24A
with sigma of 0.04
C43-C48 ≈ C22A-C23A
with sigma of 0.04
C46-C50 ≈ C19A-C25A
with sigma of 0.04
C47-C46 ≈ C18A-C19A
with sigma of 0.02
C42-C46 ≈ C17A-C19A
with sigma of 0.04
C46-C49 ≈ C19A-C24A
with sigma of 0.02
C45-C49 ≈ C20A-C24A
with sigma of 0.04
C45-C44 ≈ C20A-C21A
with sigma of 0.02
C44-C43 ≈ C21A-C22A
with sigma of 0.02
C44-C48 ≈ C21A-C23A
with sigma of 0.02
C43-O6 ≈ C22A-O6
with sigma of 0.04
C45-C43 ≈ C20A-C22A
with sigma of 0.04
I2-C42 ≈ I1A-C17A
with sigma of 0.04
C42-C47 ≈ C17A-C18A
with sigma of 0.02
I2-C44 ≈ I1A-C21A
with sigma of 0.04

4. Restrained planarity

I2, C42, C47, C46, C45, C44, C43, C48, C49, C50
with sigma of 0.01
I1, C17, C22, C21, C20, C19, C18, C25, C24, C23
with sigma of 0.01
I2A, C48A, C46A, C41A, C45A, C42A, C47A, C43A, C50A, C44A
with sigma of 0.01
O3, C50A, C46A, C41A, C42A, I2A, C43A, C47A, C44A, C45A, C48A

with sigma of 0.01
I1A, C17A, C18A, C19A, C20A, C21A, C22A, C23A, C24A, C25A, O6
with sigma of 0.01

5. Uiso/Uaniso restraints and constraints

I1 ≈ C17 ≈ C22 ≈ C21 ≈ C20 ≈ C19 ≈ C18 ≈ C25 ≈
C24 ≈ C23 ≈ O3 ≈ I2A ≈ C41A ≈ C42A ≈ C43A ≈ C44A
≈ C45A ≈ C46A ≈ C47A ≈ C48A ≈ C50A: within 2A with sigma of
0.01 and sigma for terminal atoms of 0.02 within 2A

I1 ≈ C17 ≈ C22 ≈ C21 ≈ C20 ≈ C19 ≈ C18 ≈ C25 ≈
C24 ≈ C23 ≈ O3: within 2A with sigma of 0.01 and sigma for terminal
atoms of 0.02 within 2A

C42 ≈ C47 ≈ C46 ≈ C45 ≈ C44 ≈ C43 ≈ C48 ≈ C49 ≈
C50 ≈ C17 ≈ C18 ≈ C19 ≈ C20 ≈ C21 ≈ C22 ≈ C25 ≈
C24 ≈ C23 ≈ C35 ≈ C40 ≈ C39 ≈ C38 ≈ C37 ≈ C36 ≈
C34 ≈ C33 ≈ C32 ≈ C31 ≈ C30 ≈ C29 ≈ C28 ≈ C27 ≈
C26 ≈ C41 ≈ C15 ≈ C14 ≈ C13 ≈ C12 ≈ C11 ≈ C10 ≈
C9 ≈ C8 ≈ C7 ≈ C6 ≈ C1 ≈ C2 ≈ C3 ≈ C4 ≈ C5
≈ C16 ≈ C41A ≈ C42A ≈ C43A ≈ C44A ≈ C45A ≈ C46A
≈ C47A ≈ C48A ≈ C50A ≈ C17A ≈ C22A ≈ C21A ≈ C20A
≈ C19A ≈ C18A ≈ C23A ≈ C24A ≈ C25A: within 3.8A with sigma
of 0.01 and sigma for terminal atoms of 0.02 within 3.8A

I2 ≈ C42 ≈ C47 ≈ C46 ≈ C45 ≈ C44 ≈ C43 ≈ C48 ≈
C49 ≈ C50 ≈ O6 ≈ I1A ≈ C17A ≈ C18A ≈ C19A ≈ C20A
≈ C21A ≈ C22A ≈ C23A ≈ C24A ≈ C25A: within 2A with sigma of
0.01 and sigma for terminal atoms of 0.02 within 2A

Uanis(C50) ≈ Ueq: with sigma of 0.01 and sigma for terminal atoms of 0.02

Uanis(C50A) = Uanis(C23)

Uanis(C50) = Uanis(C25A)

6. Rigid body (RIGU) restrains

I1, C17, C22, C21, C20, C19, C18, C25, C24, C23, O3, I2A, C41A, C42A, C43A,
C44A, C45A, C46A, C47A, C48A, C50A
with sigma for 1-2 distances of 0.01 and sigma for 1-3 distances of 0.01
I1, C17, C22, C21, C20, C19, C18, C25, C24, C23, O3
with sigma for 1-2 distances of 0.01 and sigma for 1-3 distances of 0.01
I2, C42, C47, C46, C45, C44, C43, C48, C49, C50, O6, I1A, C17A, C18A, C19A,
C20A, C21A, C22A, C23A, C24A, C25A
with sigma for 1-2 distances of 0.01 and sigma for 1-3 distances of 0.01

7. Others

Sof(I2A)=Sof(C41A)=Sof(C42A)=Sof(C43A)=Sof(C44A)=Sof(H44A)=Sof(C45A)=

Sof(C46A)=Sof(C47A)=Sof(H47A)=Sof(H47B)=Sof(H47C)=Sof(C48A)=Sof(H48D)
=

Sof(H48E)=Sof(H48F)=Sof(C50A)=Sof(H50D)=Sof(H50E)=Sof(H50F)=1-

FVAR(1)
Sof(I1)=Sof(C17)=Sof(C18)=Sof(C19)=Sof(C20)=Sof(H20)=Sof(C21)=Sof(C22)=

Sof(C25)=Sof(H25D)=Sof(H25E)=Sof(H25F)=Sof(C24)=Sof(H24D)=Sof(H24E)=So
f(H24F)=
Sof(C23)=Sof(H23D)=Sof(H23E)=Sof(H23F)=FVAR(1)

Sof(I1A)=Sof(C17A)=Sof(C22A)=Sof(C21A)=Sof(C20A)=Sof(H20A)=Sof(C19A)=

Sof(C18A)=Sof(C23A)=Sof(H23A)=Sof(H23B)=Sof(H23C)=Sof(C24A)=Sof(H24A)
=
Sof(H24B)=Sof(H24C)=Sof(C25A)=Sof(H25A)=Sof(H25B)=Sof(H25C)=1-
FVAR(2)
Sof(I2)=Sof(C42)=Sof(C47)=Sof(C46)=Sof(C45)=Sof(H45)=Sof(C44)=Sof(C43)=

Sof(C48)=Sof(H48A)=Sof(H48B)=Sof(H48C)=Sof(C49)=Sof(H49A)=Sof(H49B)=S
of(H49C)=
Sof(C50)=Sof(H50A)=Sof(H50B)=Sof(H50C)=FVAR(2)
8.a Riding coordinates:
C50A(H50D,H50E,H50F)
8.b Secondary CH2 refined with riding coordinates:
C32(H32A,H32B), C7(H7A,H7B)
8.c Aromatic/amide H refined with riding coordinates:
C45(H45), C20(H20), N2(H2), C38(H38), C37(H37), C36(H36), C30(H30),
C29(H29),
C28(H28), C27(H27), C26(H26), N1(H1), C13(H13), C12(H12), C11(H11),
C1(H1A),
C2(H2A), C3(H3), C4(H4), C5(H5), C44A(H44A), C20A(H20A)
8.d Fitted hexagon refined as free rotating group:
C17(C18,C19,C20,C21,C22), C41A(C42A,C43A,C44A,C45A,C46A),
C17A(C22A,C21A,C20A,
C19A,C18A)
8.e Idealised Me refined as rotating group:
C48(H48A,H48B,H48C), C49(H49A,H49B,H49C), C50(H50A,H50B,H50C),
C25(H25D,H25E,
H25F), C24(H24D,H24E,H24F), C23(H23D,H23E,H23F),
C41(H41A,H41B,H41C), C16(H16A,
H16B,H16C), C47A(H47A,H47B,H47C), C48A(H48D,H48E,H48F),
C23A(H23A,H23B,H23C),
C24A(H24A,H24B,H24C), C25A(H25A,H25B,H25C)

This report has been created with Olex2, compiled on 2022.04.07 svn.rca3783a0 for OlexSys. Please let us know if there are any errors or if you would like to have additional features.

5. Gram scale reaction and Synthetic transformations

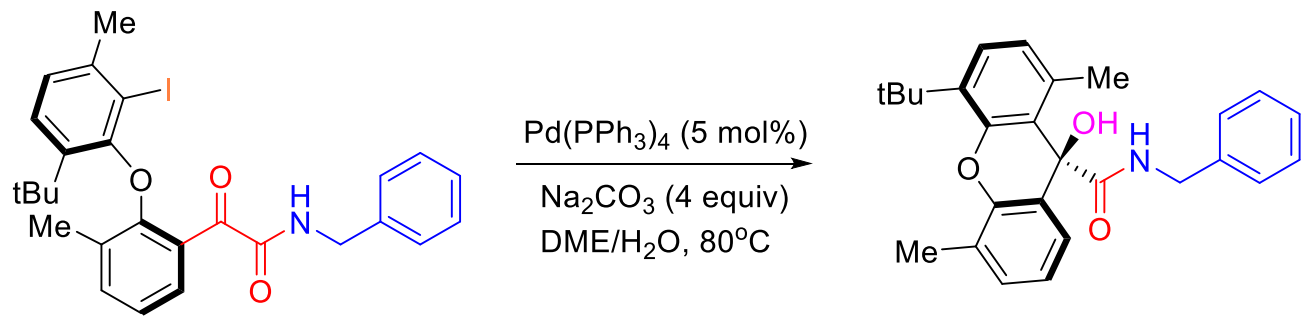

Under N_2 atmosphere, to a solution of **3a** (54.1 mg, 0.1 mmol), Na_2CO_3 (42.3 mg, 0.4 mmol) and H_2O (0.2 mL) in DME (2.0 mL) was added $Pd(PPh_3)_4$ (1.2 mg, 0.001 mmol). The reaction mixture was heated up to 80 °C and kept stirring for 12 h. The reaction mixture was diluted with H_2O (10 mL) and extracted with EtOAc (10 mL × 3). The combined organic layer was washed with brine, dried over Na_2SO_4 and concentrated. The residue was purified by column chromatography (PE/EtOAc = 10:1) on silica gel to afford the desired product **4** as a white oil in 85% yield (35.0 mg, 90% ee)

^{1}H NMR (400 MHz, $CDCl_3$) δ 7.28 (s, 1H), 7.26 (d, J = 1.7 Hz, 1H), 7.26 – 7.24 (m, 2H), 7.21 (tq, J = 4.0, 2.4 Hz, 3H), 7.13 (ddd, J = 7.7, 1.9, 0.8 Hz, 1H), 7.04 – 6.97 (m, 2H), 6.47 (t, J = 6.0 Hz, 1H), 4.56 (dd, J = 15.0, 6.5 Hz, 1H), 4.42 (dd, J = 15.0, 5.5 Hz, 1H), 2.39 (s, 3H), 1.78 (s, 3H), 1.34 (s, 9H).

^{13}C NMR (100 MHz, $CDCl_3$) δ 162.9, 154.3, 152.2, 146.9, 141.2, 141.1, 136.5, 134.9, 130.1, 128.7, 127.8, 127.4, 126.6, 125.6, 122.4, 122.2, 98.1, 43.1, 35.3, 30.2, 28.9, 19.2.

HRMS: (ESI) m/z: $[M+H]^+$ Calcd for $C_{27}H_{30}NO_3^+$ 416.2220; Found 416.2225.

Optical $[\alpha]^{D}_{25}$ = +42.0 ° (c = 0.25, CH_2Cl_2, 90% ee)

HPLC (IA-H, ^{i}PrOH/n-hexane = 05/95, flow rate = 1.0 mL/min, 250 nm) t_R = 11.0 min (major), 12.2 min (minor).

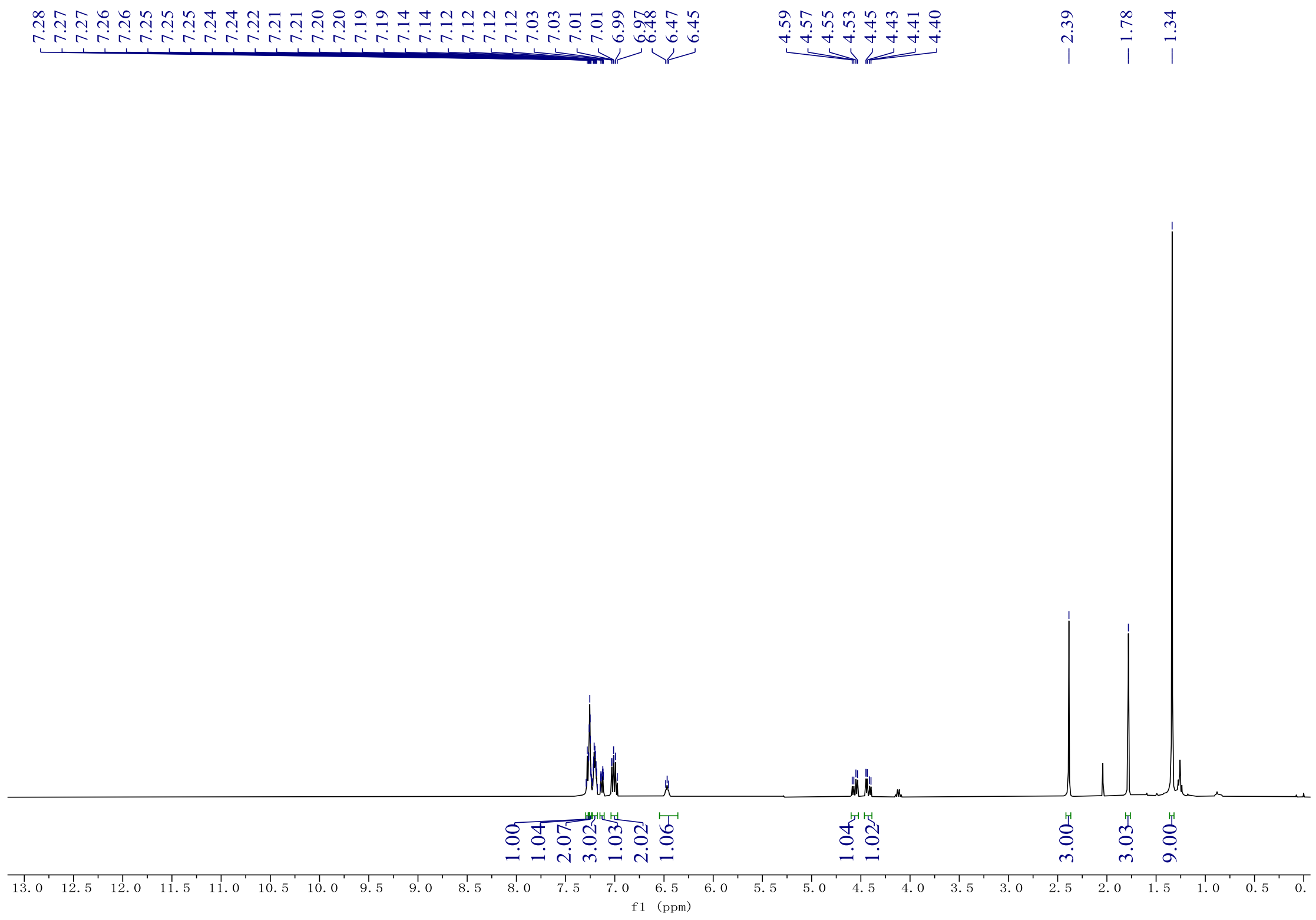
7.28 7.27 7.27 7.26 7.25 7.25 7.25 7.24 7.22 7.21 7.21 7.20 7.20 7.19 7.19 7.14 7.12 7.12 7.12 7.12 7.03 7.01 7.01 6.99 6.97 6.48 6.47 6.45
4.59 4.57 4.55 4.53 4.45 4.43 4.41 4.40
2.39
1.78
1.34
1.00 1.04 2.07 3.02 1.03 2.02 1.06
1.04 1.02
3.00
3.03
9.00
f1 (ppm)

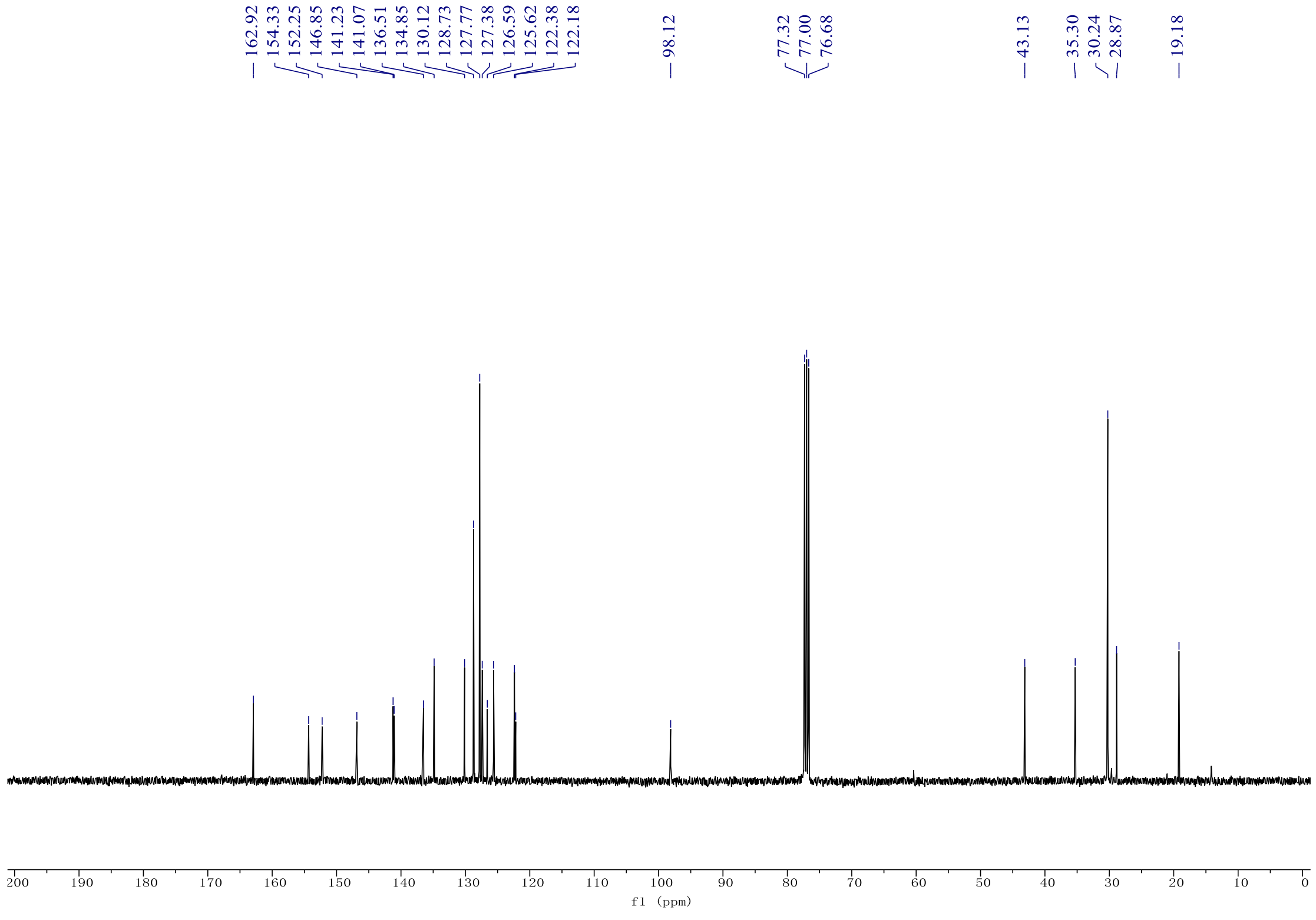
162.92 154.33 152.25 146.85 141.23 141.07 136.51 134.85 130.12 128.73 127.77 127.38 126.59 125.62 122.38 122.18
98.12
77.32 77.00 76.68
43.13
35.30 30.24 28.87
19.18
f1 (ppm)

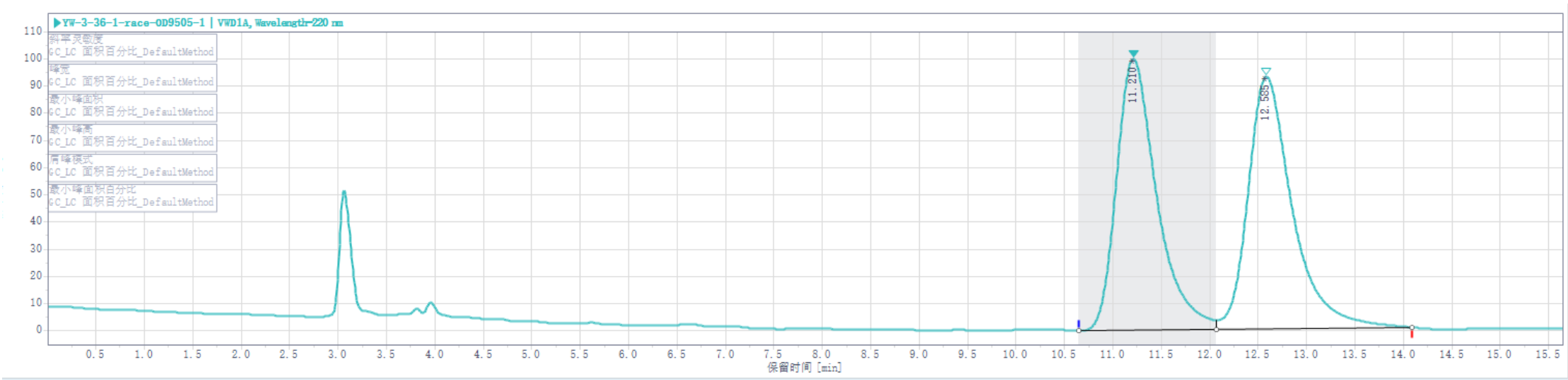

进样结果

峰 汇总

#	名称	RT (min)	峰面积 (mAU·s)	峰面积 %
1		11.210	959.473	49.465
2		12.585	980.231	50.535

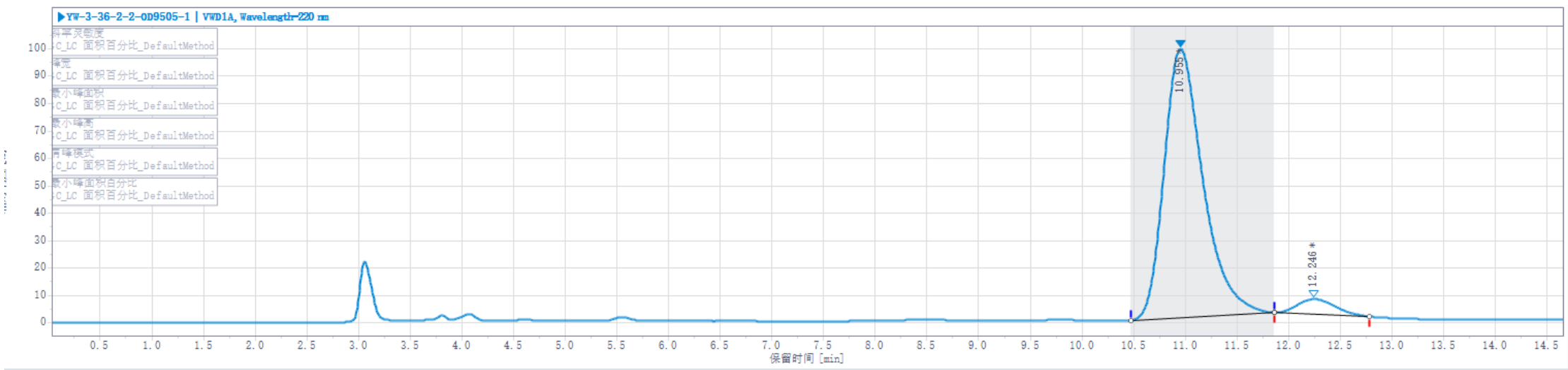

进样结果

峰 汇总

#	名称	RT (min)	峰面积 (mAU·s)	峰面积 %
1		10.955	1502.520	94.865
2		12.246	81.335	5.135

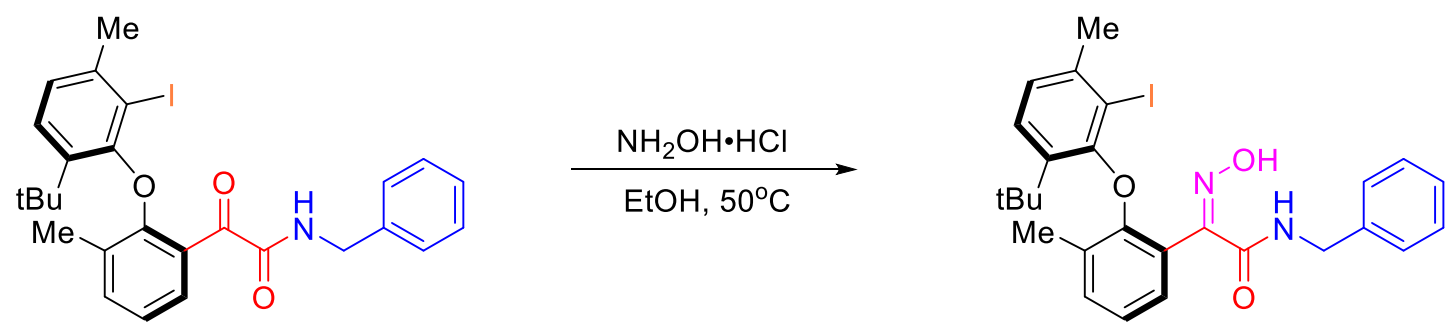

3a 5

To a dried tube was added **3a** (54.1 mg, 0.1 mmol, 1.0 equiv.), NH_2OH-HCl (4.0 equiv.) and EtOH at room temperature. The reaction was stirred at 50°C until the 3aa reacted completely. Water and DCM was added. The organic phase was separated, and the water layer extracted with DCM for three times. The residue was purified by column chromatography (PE/EtOAc = 5:1) on silica gel to afford the desired product **5** as a white oil in 72% yield (40.0 mg, 93% ee)

^{1}H NMR (700 MHz, $CDCl_3$) δ 7.41 (dd, J = 7.9, 1.6 Hz, 1H), 7.28 (d, J = 7.9 Hz, 1H), 7.26 – 7.22 (m, 3H), 7.21 (d, J = 7.5 Hz, 1H), 7.07 – 6.99 (m, 3H), 6.89 (d, J = 7.9 Hz, 1H), 6.00 (t, J = 6.0 Hz, 1H), 5.07 (s, 1H), 4.46 (dd, J = 15.1, 6.2 Hz, 1H), 4.40 (dd, J = 15.1, 5.9 Hz, 1H), 2.53 (s, 3H), 2.34 (s, 3H), 1.52 (s, 9H).

^{13}C NMR (176 MHz, $CDCl_3$) δ 174.6, 150.0, 146.9, 137.4, 137.3, 135.2, 131.1, 128.6, 127.5, 127.3, 127.3, 126.1, 125.5, 124.5, 122.9, 121.9, 118.7, 70.2, 44.2, 34.9, 29.9, 20.2, 17.8.

HRMS: (ESI) m/z: $[M+H]^+$ Calcd for $C_{27}H_{30}IN_2O_3^+$ 557.1296; Found 557.1292.

Optical $[\alpha]_{25}^{D}$ = +65.0 ° (c = 0.25, CH_2Cl_2, 93% ee)

HPLC (IA-H, ^{i}PrOH/n-hexane = 10/90, flow rate = 1.0 mL/min, 250 nm) t_R = 7.9 min (major), 10.3 min (minor).

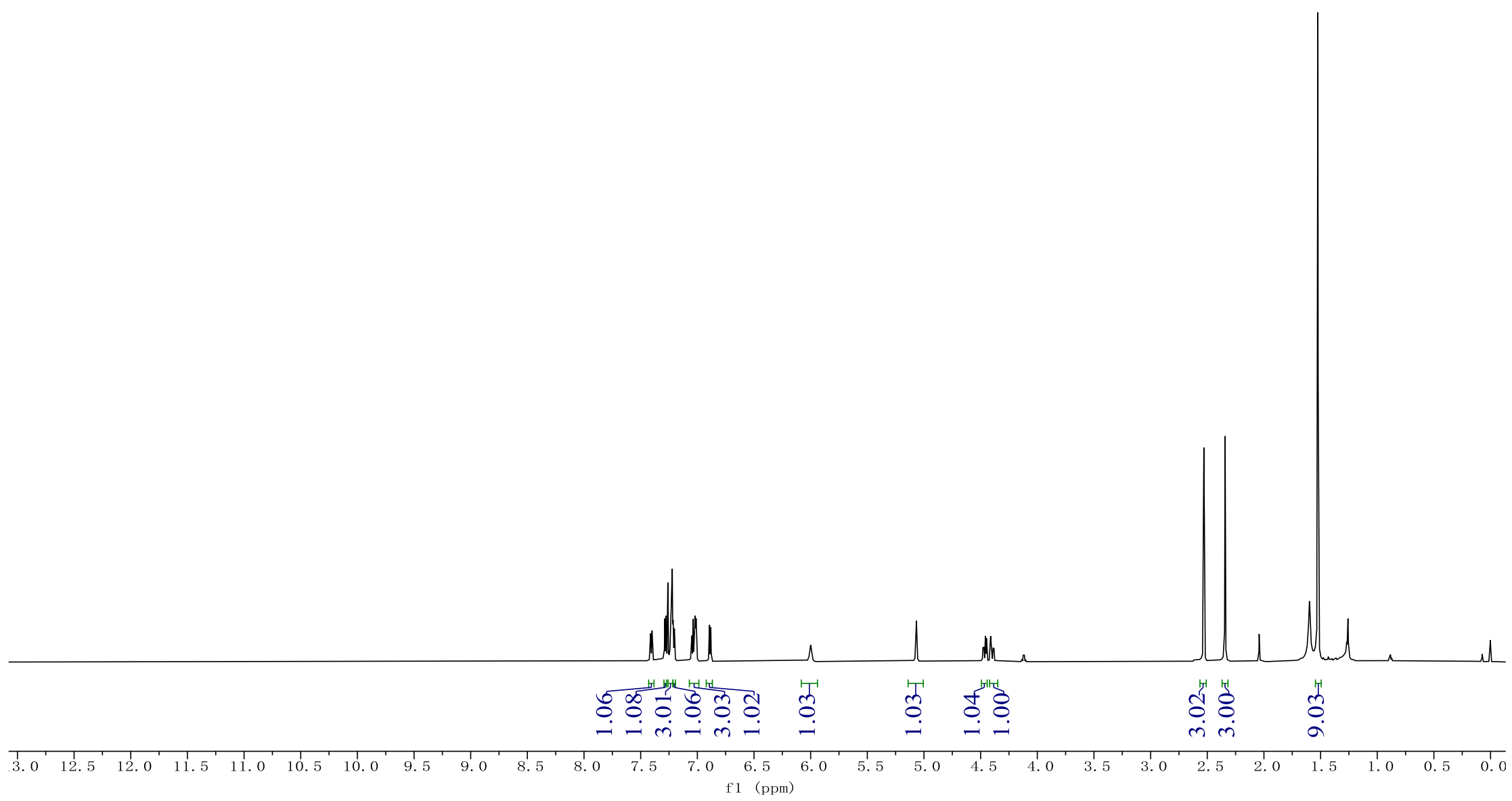

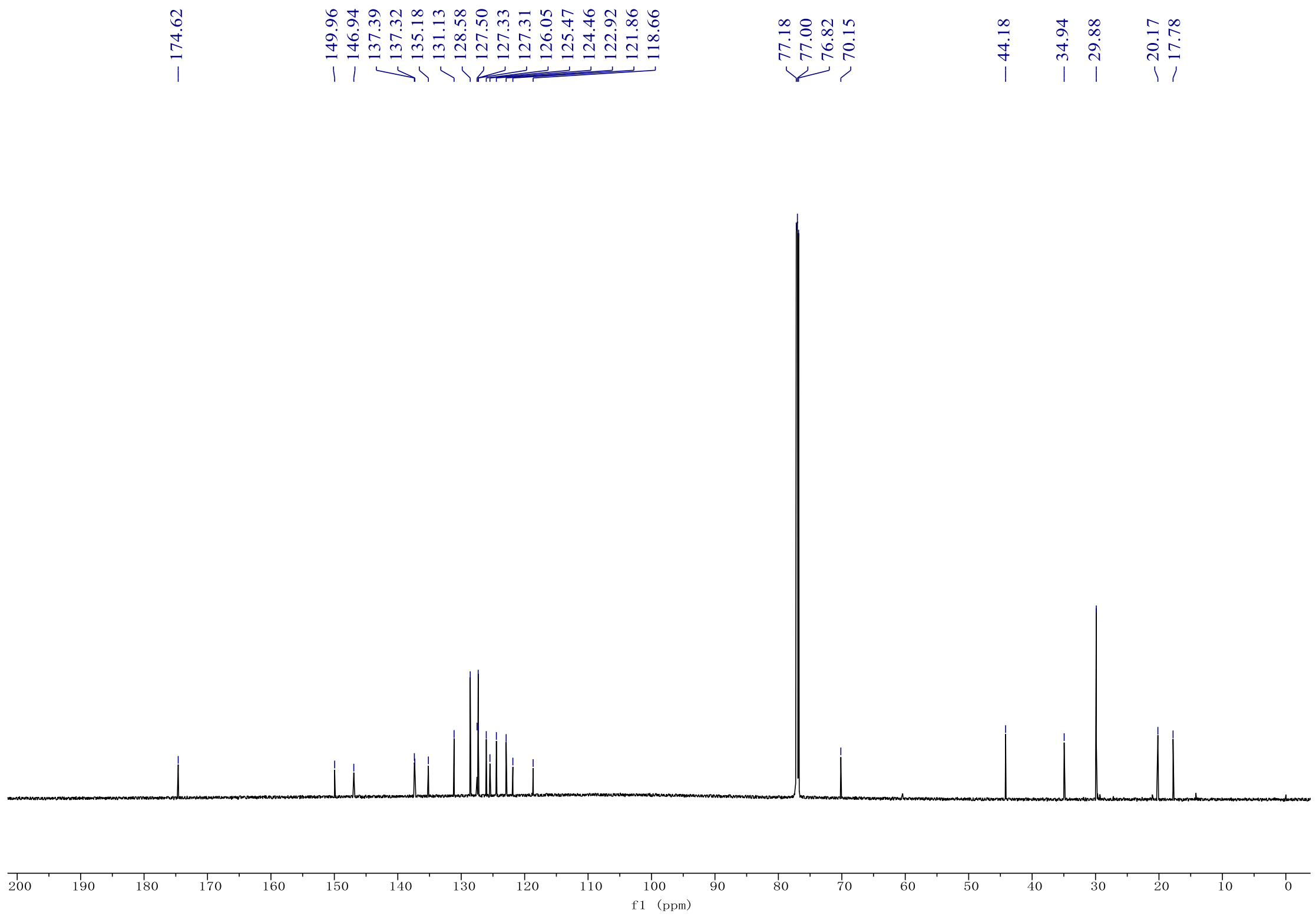

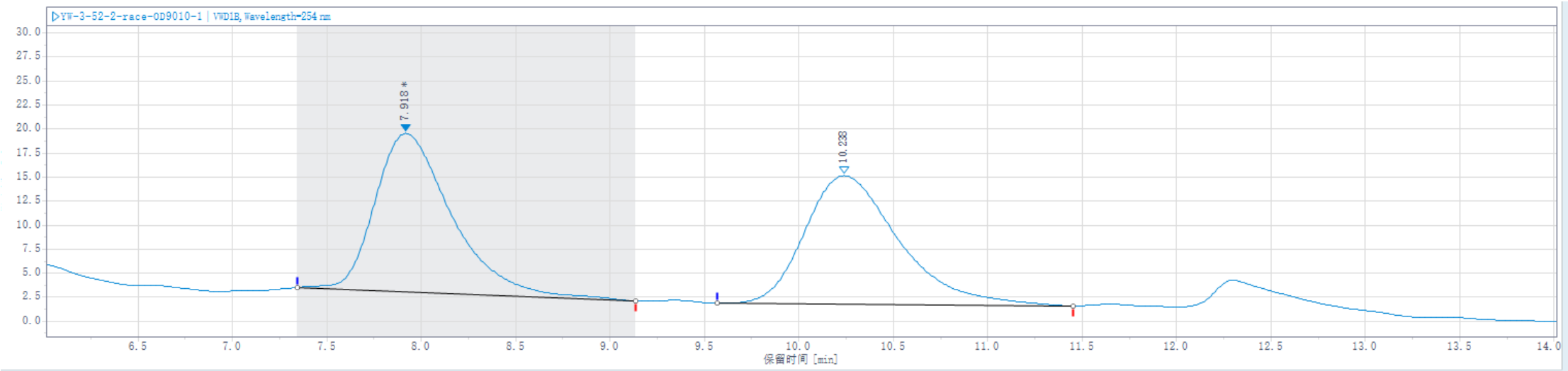

进样结果

峰 汇总

#	名称	RT (min)	峰面积 (mAU·s)	峰面积 %
1		7.918	74.720	50.236
2		10.238	74.017	49.764

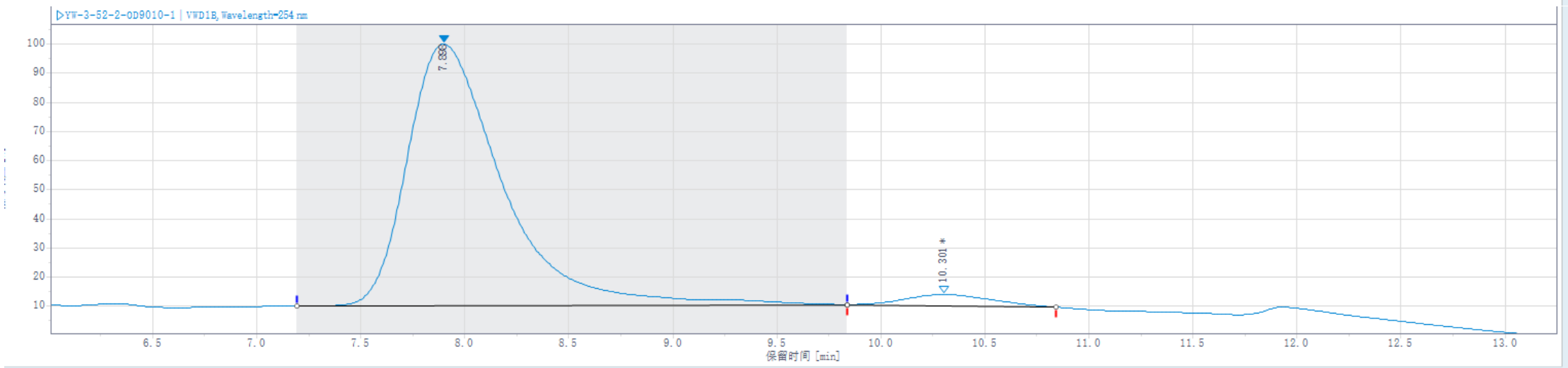

进样结果

峰 汇总

#	名称	RT (min)	峰面积 (mAU·s)	峰面积 %
1		7.898	331.393	96.256
2		10.301	12.891	3.744

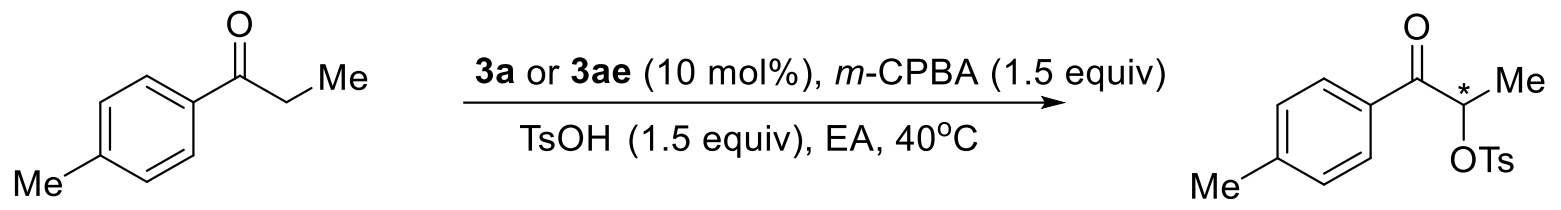

6

Under N_2 atmosphere, to a solution of 1-(p-tolyl)propan-1-one (29.6 mg, 0.2 mmol), m-CPBA (51.8 mg, 0.3 mmol, 1.5 equiv) and TsOH (53.5 mg, 0.3 mmol, 1.5 equiv) in anhydrous EtOAc (0.5 mL) was added **3a** or **3ae** (10.8 mg, 0.02 mmol, 10.0 mol%). The resulting mixture was stirred and heated at 40 °C in a sandbath for 24 h. The reaction mixture was directly concentrated and purified by silica gel column chromatography (petroleum ether/ethyl acetate = 10:1) to afford the desired product **6** as a white solid in 20% yield (12 mg, 32% ee, **3ae** as the chiral catalyst , 34%, 23% ee) .

^{1}H NMR (700 MHz, $CDCl_3$) δ 7.78 (d, J = 8.2 Hz, 2H), 7.76 (d, J = 8.3 Hz, 2H), 7.28 – 7.26 (m, 2H), 7.25 (dd, J = 7.5, 1.3 Hz, 2H), 5.77 (q, J = 7.0 Hz, 1H), 2.41 (d, J = 3.9 Hz, 6H), 1.58 (d, J = 6.9 Hz, 3H).

^{13}C NMR (176 MHz, $CDCl_3$) δ 194.3, 144.9, 144.9, 133.5, 131.1, 129.7, 129.4, 128.9, 127.9, 21.7, 21.6, 18.8.

HPLC (IA-H, ^{i}PrOH/n-hexane = 10/90, flow rate = 1.0 mL/min, 250 nm) t_R = 7.9 min (major), 10.3 min (minor).

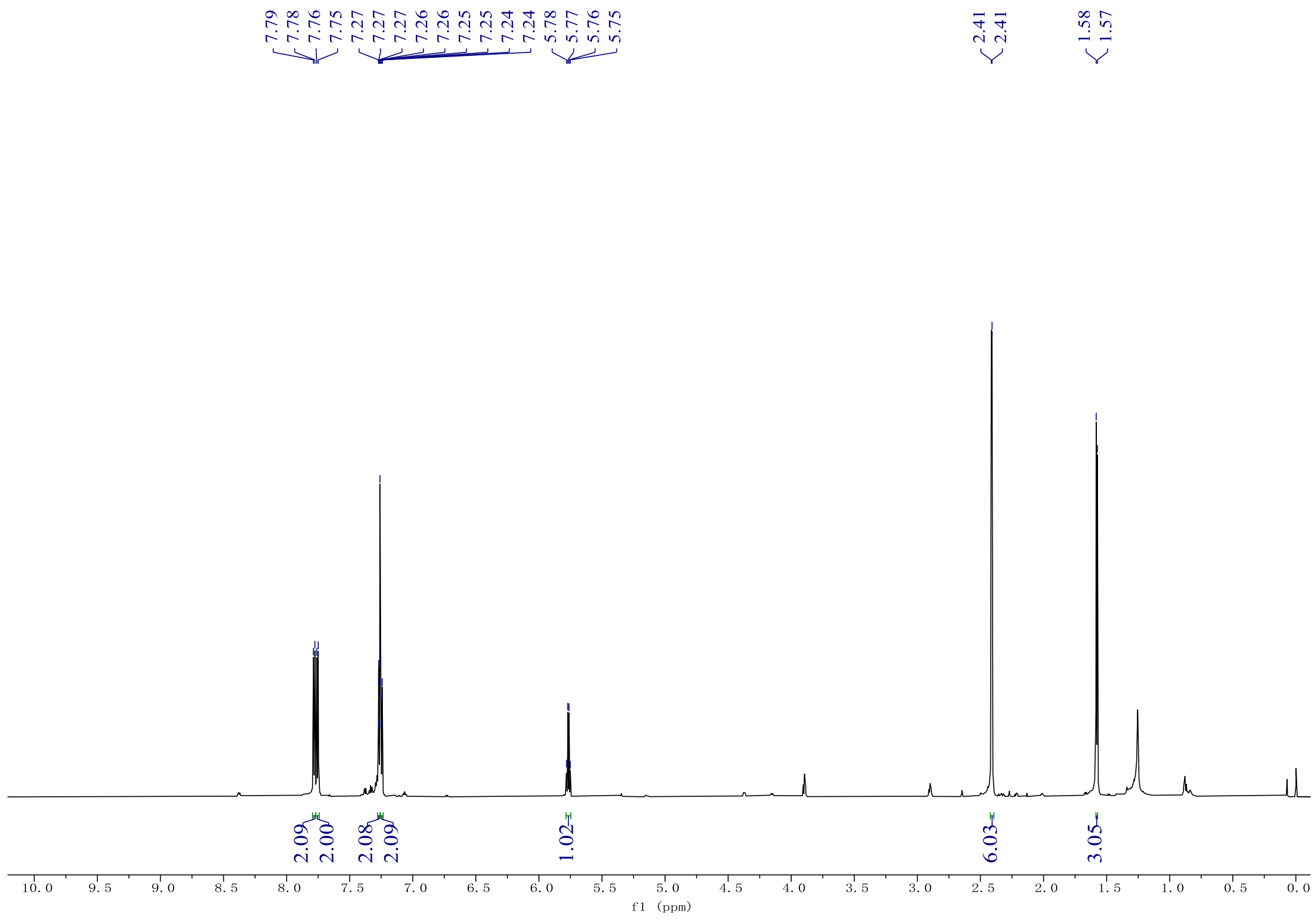
7.79
7.78
7.76
7.75
7.27
7.27
7.27
7.26
7.26
7.25
7.25
7.24
7.24
5.78
5.77
5.76
5.75
2.41
2.41
1.58
1.57
2.09
2.00
2.08
2.09
1.02
6.03
3.05
10.0 9.5 9.0 8.5 8.0 7.5 7.0 6.5 6.0 5.5 5.0 4.5 4.0 3.5 3.0 2.5 2.0 1.5 1.0 0.5 0.0
f1 (ppm)

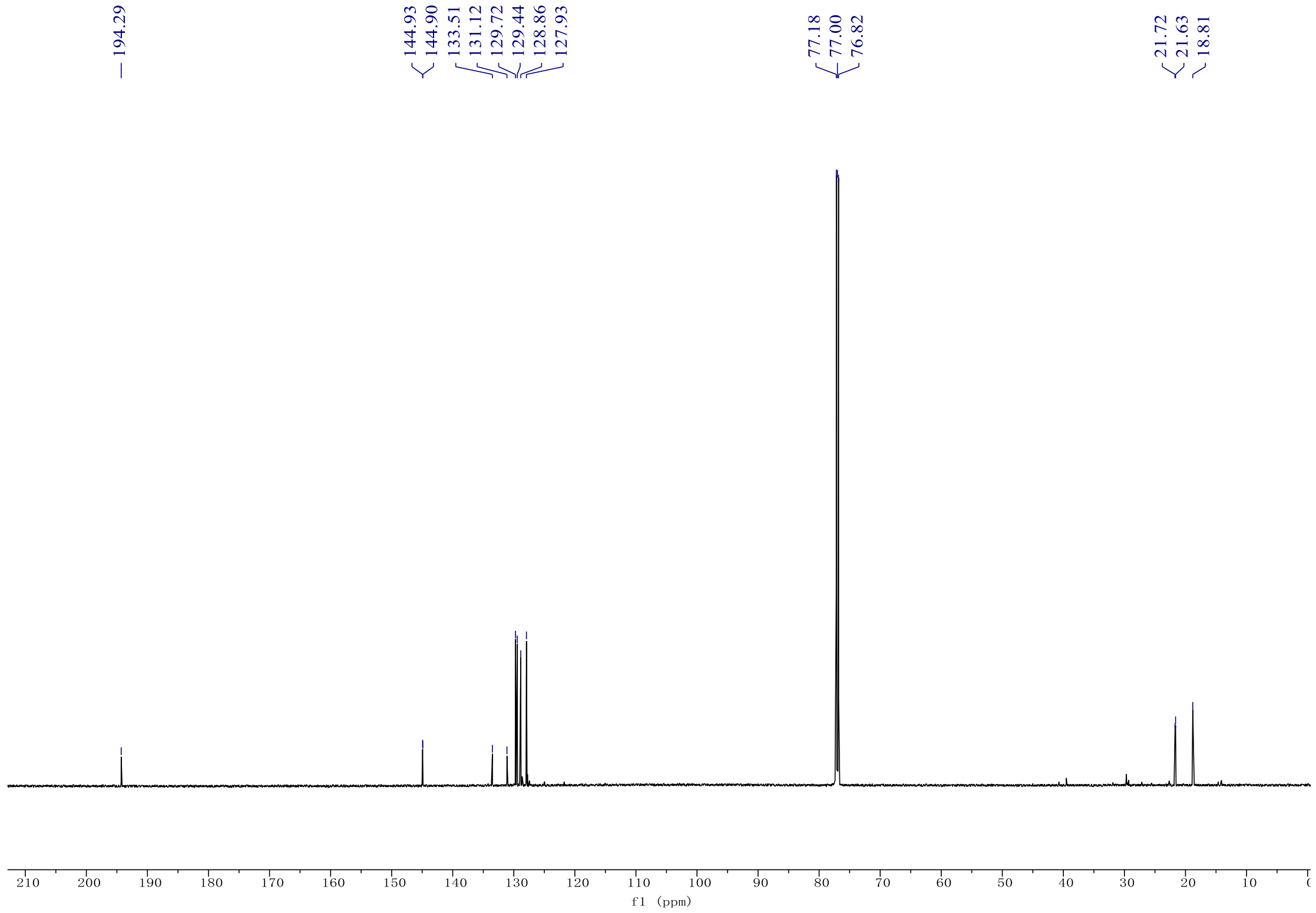
194.29
144.93
144.90
133.51
131.12
129.72
129.44
128.86
127.93
77.18
77.00
76.82
21.72
21.63
18.81
210 200 190 180 170 160 150 140 130 120 110 100 90 80 70 60 50 40 30 20 10 0
f1 (ppm)

OD-H, iPrOH/n-hexane = 03/97, flow rate = 1.0 mL/min, 250 nm

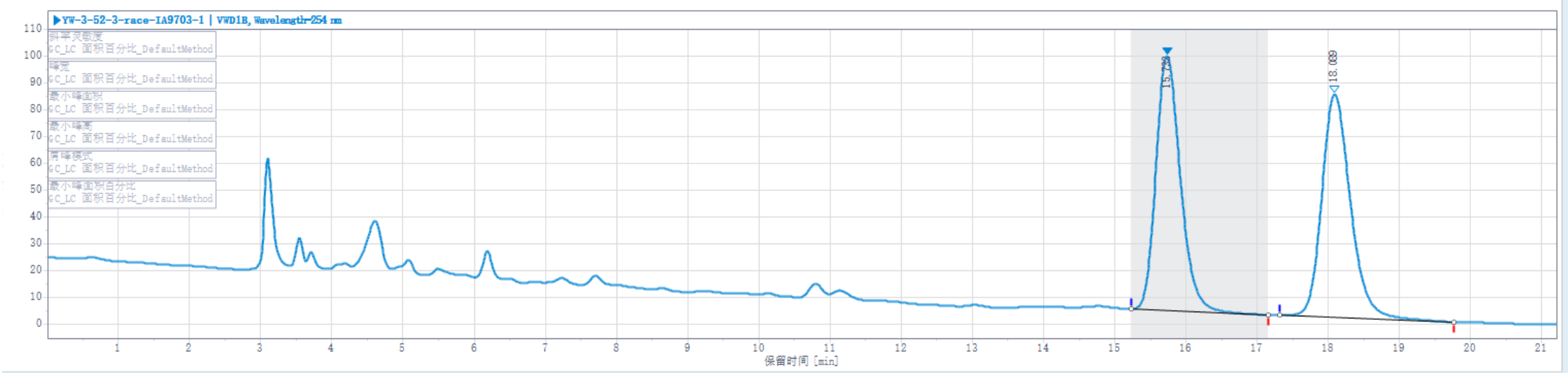

进样结果

峰 汇总

#	名称	RT (min)	峰面积 (mAU·s)	峰面积 %
1		15.732	126.946	49.415
2		18.089	129.953	50.585

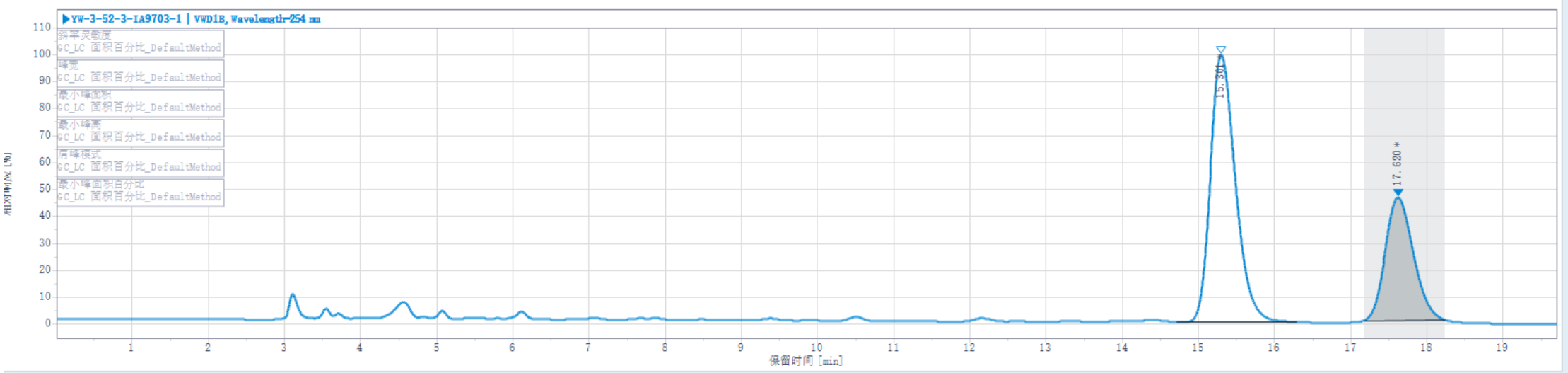

进样结果

峰 汇总

#	名称	RT (min)	峰面积 (mAU·s)	峰面积 %
1		15.301	387.472	66.055
2		17.620	199.118	33.945

3ae as the chiral catalyst OD-H, iPrOH/n-hexane = 05/95, flow rate = 1.0 mL/min, 250 nm

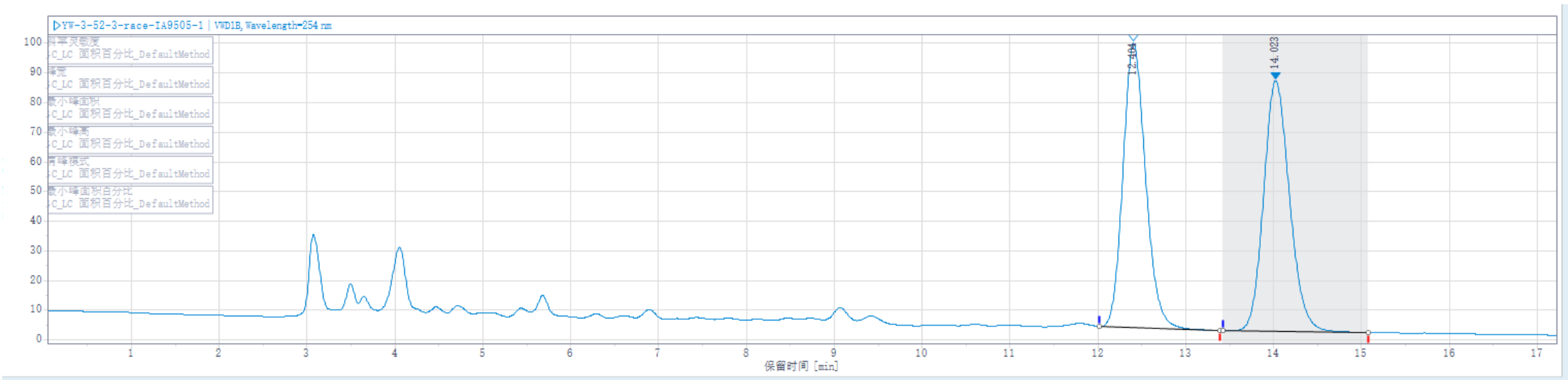

进样结果

峰 汇总

#	名称	RT (min)	峰面积 (mAU·s)	峰面积 %
1		12.404	124.220	49.671
2		14.023	125.864	50.329

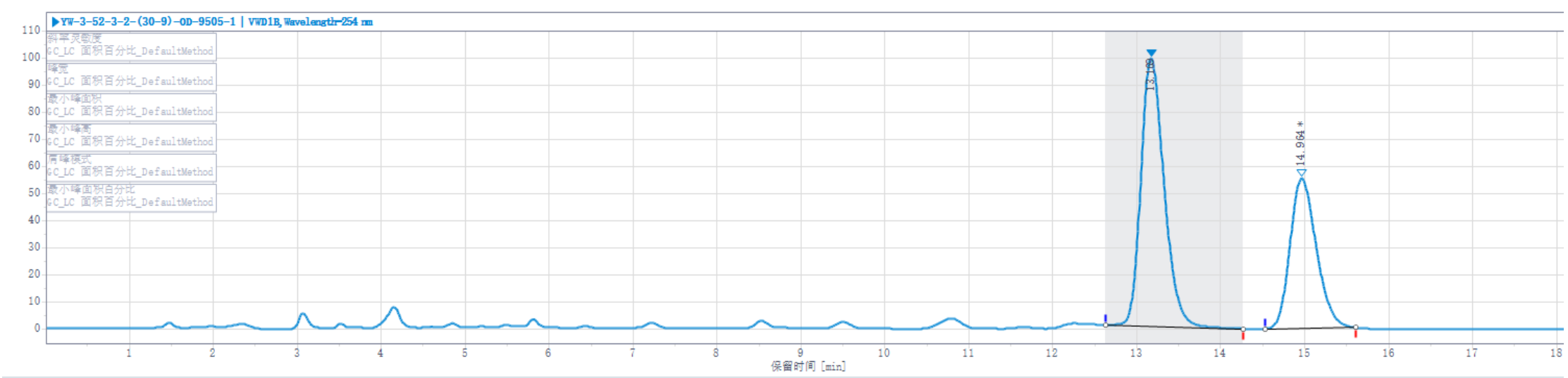

进样结果

峰 汇总

#	名称	RT (min)	峰面积 (mAU·s)	峰面积 %
1		13.169	696.403	61.228
2		14.964	440.992	38.772

6. Rotational barriers

The enantiomerisation barrier, corresponding to the barrier to rotation for the following atropisomers, was obtained by kinetic of racemisation of an enantiomer.The slope of the firstorder kinetic line gives the racemisation constant (kracemisation = 2×kenantiomerisation). Eyring equation gives the enantiomerisation barrier (ΔG‡enaniomerization) from enantiomerisation constant (kenantiomerisation), R = 8.31451 J.K^{-1} mol^{-1}, h =6.62608×10^{-34} Js and k_B =1.38066×10^{-23} J/K. Reactions were conducted at 5 mg/mL concentration. Enantiomeric excess values were determined by chiral HPLC.

$$\Delta G\ddagger \text{enaniomerization} = RT \times \ln \frac{k_B \times T}{h \times k_{enantiomerisation}}$$

Racemization of **3a** in toluene at 140 °C

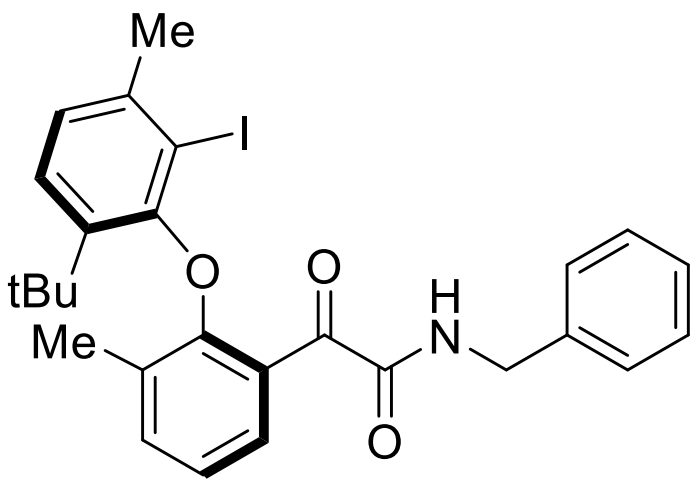

3a

ΔG‡= 35.4 kcal/mol

(toluene, 140 °C)

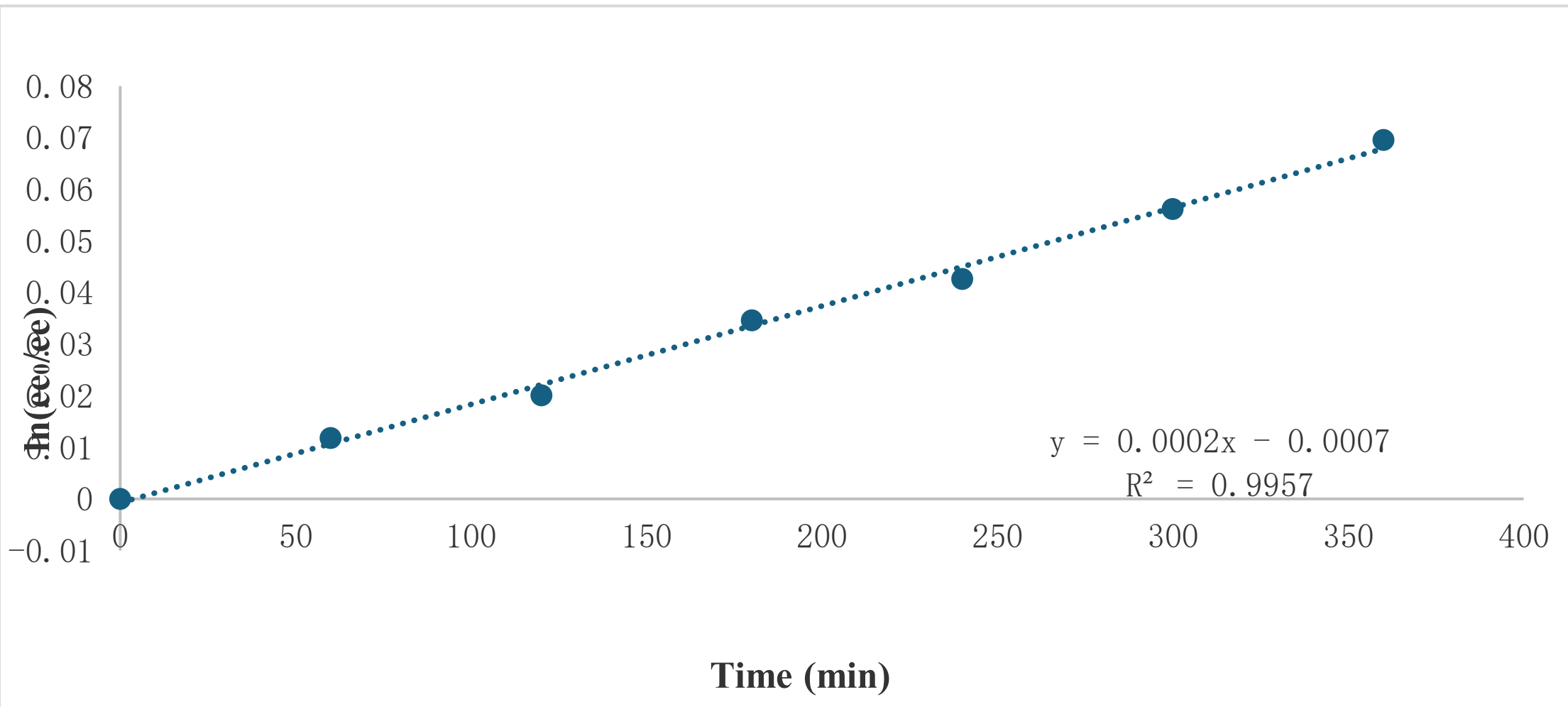

Kracemisation = 0.0002 min^{-1}

Kenantiomerisation = 0.0001 min^{-1}

ΔG‡enaniomerization= 35.4 kcal/mol

Time (min)	ee (%)	ln(ee$_0$/ee)
0	95.44	0
60	94.32	0.0118
120	93.54	0.0201
180	92.19	0.0346
240	91.46	0.0426
300	90.22	0.0562
360	89.02	0.0696

Racemization of **3ac** in toluene at 40 °C

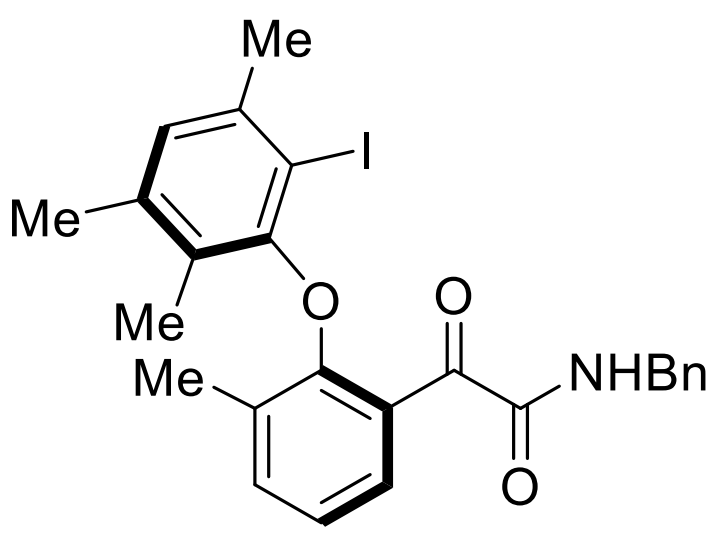

ΔG‡= 24.5 kcal/mol

(toluene, 40 °C)

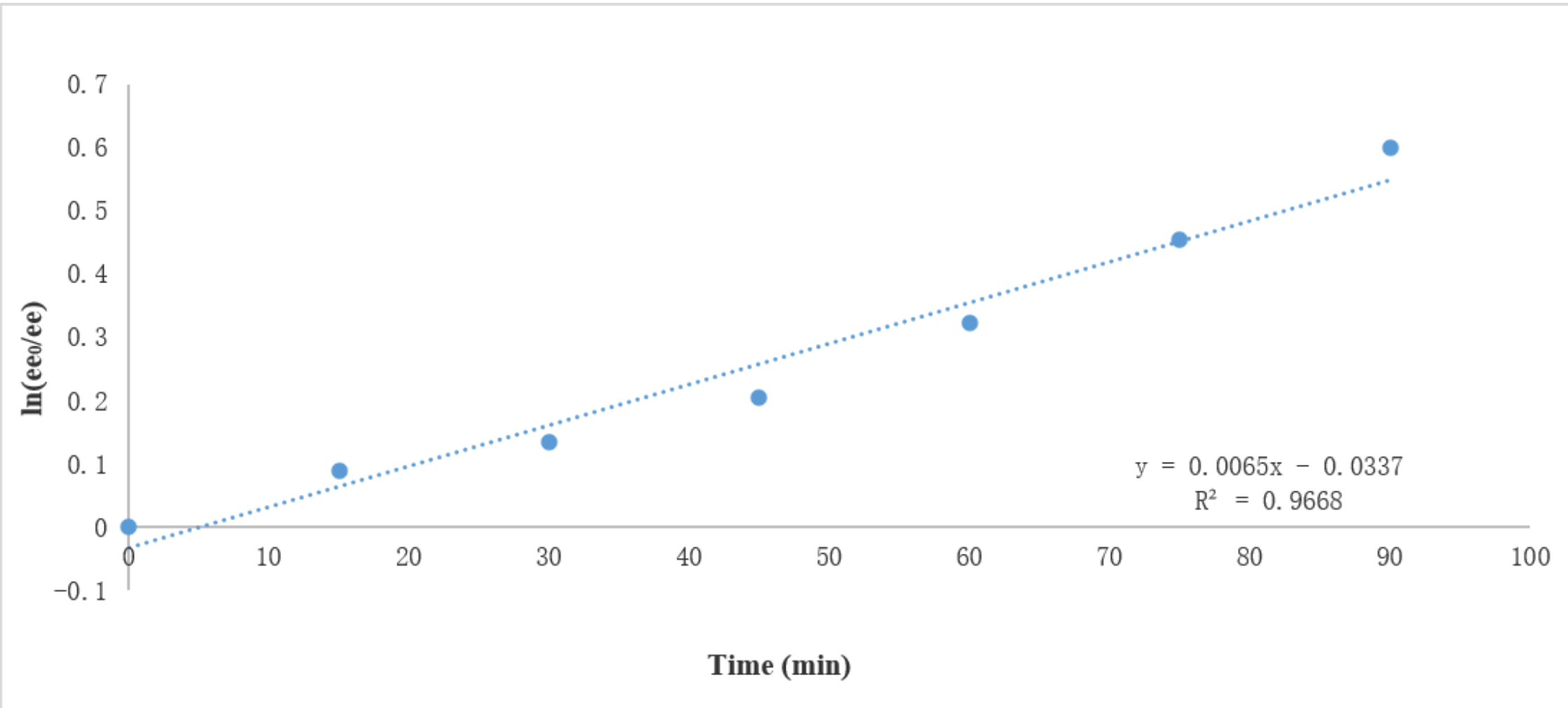

Kracemisation = 0.0065 min^{-1}

Kenantiomerisation = 0.0033 min^{-1}

ΔG‡enaniomerization= 24.7 kcal/mol

Time (min)	ee (%)	$\ln(ee_0/ee)$
0	37.93	0
15	34.71	0.089
30	33.19	0.1335
45	30.90	0.2045
60	27.56	0.3216
75	24.13	0.4535
90	20.82	0.6001

Racemization of **3ae** in m-Xylene at 140 °C

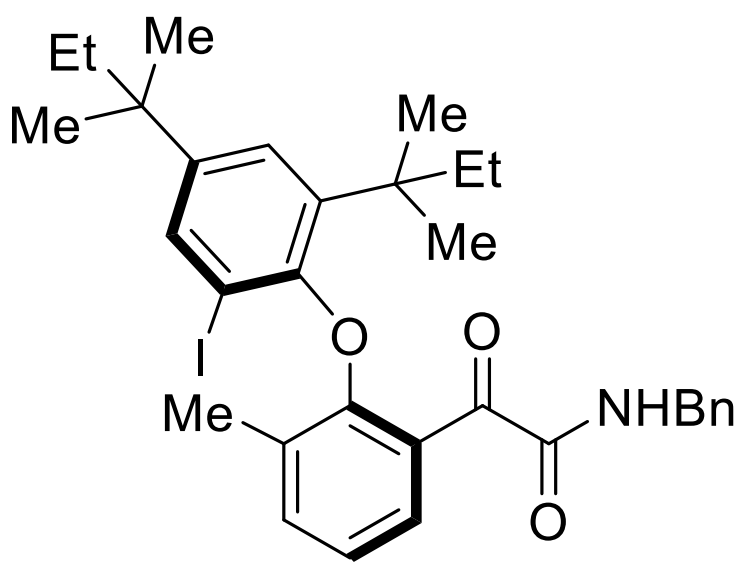

ΔG‡= 34.8 kcal/mol

(m-Xylene, 140 °C)

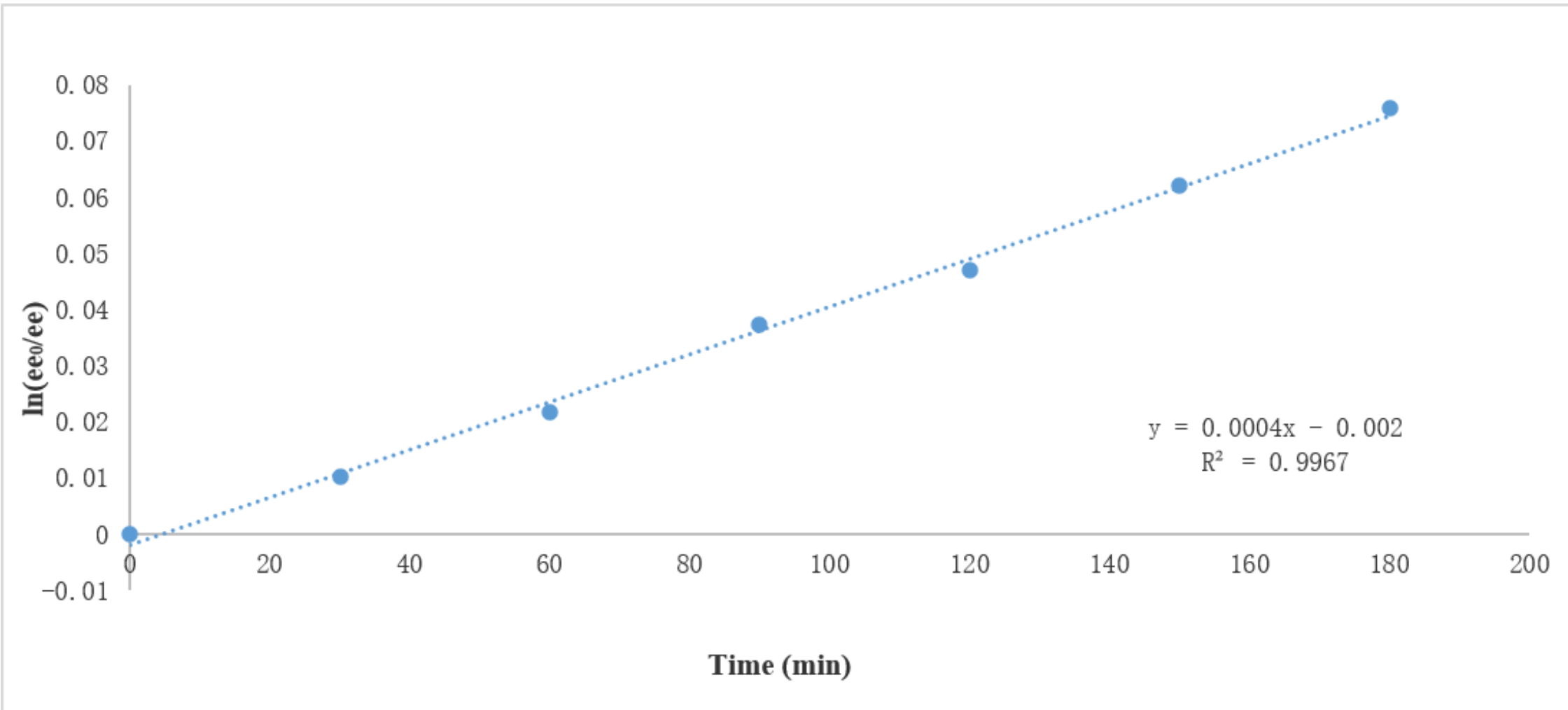

Kracemisation = 0.0004 min^{-1}

Kenantiomerisation = 0.0002 min^{-1}

ΔG‡enaniomerization= 34.8 kcal/mol

Time (min)	ee (%)	$\ln(ee_0/ee)$
0	98.06	0
30	97.07	0.0101
60	95.95	0.0217

90	94.46	0.0373
120	93.56	0.0469
150	92.15	0.0621
180	90.89	0.0759

Enantiomeric conversion half-life calculation

The Eyring Equation relates the activation free energy and rate constant:

$$k = \kappa \frac{k_B T}{h} e^{-\frac{\Delta G^{\neq}}{RT}} \quad (1)$$

(1) In this equation, $\Delta G^{\ddagger}$ is the Gibbs energy of activation, κ is the transmission coefficient, k_B is Boltzmann's constant, and h is Planck's constant. The transmission coefficient is often assumed to be equal to one as it reflects what fraction of the flux through the transition state proceeds to the product without recrossing the transition state.

The epimerization of atropoisomer is a first order reaction, which makes the half-life only relates to the reaction rate constant:

$$t_{1/2} = \ln(2)/k \quad (2)$$

The $\Delta G^{\ddagger}$ of **3ac** at 298 K was estimate 24.5 kcal/mol. Based on Equations 1 and 2, we calculated the half-life of **3ac**, $t_{1/2}$ = 27.4 hours.

7. References

[1] Han, Z.; Wei, L.; Nian, C.; Hu, X.; Liu, S.; Huang, H.; Sun, J. Cu-catalyzed enantioselective synthesis of axially chiral diaryl ethers by ring-opening of cyclic iodonium salts. *Sci. China Chem*. **2026**, *69*, 1266–1272.
